# An integrative genomics approach identifies novel pathways that influence candidaemia susceptibility

**DOI:** 10.1371/journal.pone.0180824

**Published:** 2017-07-20

**Authors:** Vasiliki Matzaraki, Mark S. Gresnigt, Martin Jaeger, Isis Ricaño-Ponce, Melissa D. Johnson, Marije Oosting, Lude Franke, Sebo Withoff, John R. Perfect, Leo A. B. Joosten, Bart Jan Kullberg, Frank L. van de Veerdonk, Iris Jonkers, Yang Li, Cisca Wijmenga, Mihai G. Netea, Vinod Kumar

**Affiliations:** 1 Department of Genetics, University Medical Center Groningen, Groningen, The Netherlands; 2 Department of Internal Medicine and Radboud Center for Infectious Diseases, Radboud University Medical Center, Nijmegen, The Netherlands; 3 Duke University Medical Center, Durham, North Carolina, United States of America; 4 K.G. Jebsen Coeliac Disease Research Centre, Department of Immunology, University of Oslo, Oslo, Norway; 5 Human Genomics Laboratory, Craiova University of Medicine and Pharmacy, Craiova, Romania; Louisiana State University, UNITED STATES

## Abstract

Candidaemia is a bloodstream infection caused by *Candida* species that primarily affects specific groups of at-risk patients. Because only small candidaemia patient cohorts are available, classical genome wide association cannot be used to identify *Candida* susceptibility genes. Therefore, we have applied an integrative genomics approach to identify novel susceptibility genes and pathways for candidaemia. *Candida*-induced transcriptome changes in human primary leukocytes were assessed by RNA sequencing. Genetic susceptibility to candidaemia was assessed using the Illumina immunochip platform for genotyping of a cohort of 217 patients. We then integrated genetics data with gene-expression profiles, *Candida-*induced cytokine production capacity, and circulating concentrations of cytokines. Based on the intersection of transcriptome pathways and genomic data, we prioritized 31 candidate genes for candidaemia susceptibility. This group of genes was enriched with genes involved in inflammation, innate immunity, complement, and hemostasis. We then validated the role of *MAP3K8* in cytokine regulation in response to *Candida* stimulation. Here, we present a new framework for the identification of susceptibility genes for infectious diseases that uses an unbiased, hypothesis-free, systems genetics approach. By applying this approach to candidaemia, we identified novel susceptibility genes and pathways for candidaemia, and future studies should assess their potential as therapeutic targets.

## Introduction

Genome-wide association studies (GWAS) have greatly contributed to the identification of susceptibility genes for human complex diseases. However, the need for large cohorts precludes the use of GWAS to identify susceptibility genes for infectious diseases for which only relatively small patient cohorts can be recruited. So far, relatively few GWAS studies have been performed in patients with infections, including studies of viral and bacterial infections [1]. Compared to the hundreds of genetic loci that have been associated to human complex diseases, the number of susceptibility genes identified as associated to infectious diseases remains low. Given that the risk of death due to infectious diseases has a large genetic heritability [2], methods other than GWAS that can be applied to smaller cohorts are crucial to make progress in understanding and treating these diseases.

Candidaemia is the fourth most common systemic bloodstream infection in the United States (US) and is associated with mortality rates of up to 40% [3,4]. It is caused by opportunistic fungal pathogens belonging to the *Candida* species, particularly *Candida albicans* (*C*. *albicans)*, and primarily affects patients with a compromised immune system [5]. However, not all at-risk patients develop candidaemia, indicating that individual differences—including genetic background—influence their susceptibility to the infection. Despite the availability of potent antifungal drugs, the mortality rate of systemic *Candida* infections remains unacceptably high [6]. In addition to current treatment strategies, only adjuvant immunotherapy such as the administration of recombinant cytokines is believed to improve outcome [7]. Therefore, an understanding of the molecular pathways involved in the human host defence and identification of susceptibility genes is crucial for the design of appropriate prophylactic and immunotherapeutic strategies.

To identify genes underlying susceptibility to *Candida* infection, we have applied a systems genomics approach that integrates genetic data from candidaemia patients genotyped with the Immunochip platform [8] with *Candida*-induced gene-expression profiles in human leukocytes, cytokine production from *Candida*-stimulated peripheral blood mononuclear cells (PBMCs), and circulating cytokine concentrations in candidaemia patients. Using this approach, we demonstrate that genetic susceptibility loci with suggestive associations (P < 9.99 x 10^−5^) could play a major role in candidaemia susceptibility and we identify genes involved in inflammation, innate immunity, complement and hemostasis as having an important role in determining susceptibility to candidaemia.

## Materials and methods

### Study populations

To identify genetic variants associated with candidaemia, we performed a two-stage Immunochip-wide analysis of a candidaemia cohort using two control groups in 2014: a population-based healthy cohort and disease-matched cohort (European ancestry), as previously described [9]. Briefly, for Immunochip-wide association analysis, we first used a cohort consisting of 217 candidaemia patients of European ancestry and 11,920 population-based healthy controls (Discovery stage). The demographic and clinical characteristics of the candidaemia cohort have been previously described [9]. Re-analysis of the data and prioritization of genes from susceptibility loci with suggestive associations (P < 9.99 x 10^−5^) was performed in 2017.

After the discovery of single nucleotide polymorphisms (SNPs) for susceptibility to candidaemia using population-based healthy controls, we then validated our findings using a validation control consisting of 146 disease-matched but candidiasis-free controls. These candidiasis-free control patients were recruited from the same hospital wards as the candidaemia patients so that co-morbidities and clinical risk factors for infection were as similar as possible between patients and controls.

### Ethics statement

The study was approved by the institutional review boards at each study centre, and enrollment occurred between January 2003 and January 2009. The study centers are the Duke University Hospital (DUMC, Durham, North Carolina, USA) and Radboud University Nijmegen Medical Centre (Nijmegen, The Netherlands). All adult subjects provided informed written consent.

### Genotyping and case-control analysis of candidaemia cohort

DNA was isolated using the Gentra Pure Gene Blood kit (Qiagen, Venlo, the Netherlands) according to the protocol of the manufacturer. Genotyping of candidaemia patients and both control groups was performed using Immunochip according to Illumina’s protocol [10]. Genotype data analysis and quality control of this cohort has been previously reported [9]. Briefly, in the discovery stage, the associations of Immunochip SNPs and susceptibility with candidaemia were tested by logistic regression using the first four components from the multidimensional scaling analysis as covariates. We considered a P value < 9.99 x 10^−5^ as the threshold for suggestive association to select 268 SNPs in 77 independent loci for validation. For validation analysis using candidaemia case-matched controls, the association between SNPs and candidaemia was tested by logistic regression using the first four multidimensional scaling analysis components as covariates. A validation P value < 0.05 was considered significant.

### PBMC isolation and stimulation with *Candida albicans*

Isolation of PBMCs from eight healthy volunteers and stimulation of PBMCs was performed as described previously [11]. After cell counting with a hemocytometer, the cell number was adjusted to 5 x 10^6^/mL. To identify the transcriptome upon *Candida* stimulation, 5 x 10^5^ isolated PBMCs were incubated with 1 x 10^6^/mL heat-killed *C*. *albicans* (UC 820) (*C*. *albicans*:PBMC ratio of 1:2.5) and RPMI culture medium as a control for 4 and 24 hours. *C*. *albicans* UC 820 is a well-described strain regarding its immune responses in PBMCs [12].

### Analysis of RNA sequencing reads

The RNA sequencing analysis of this dataset was described previously [13]. Briefly, sequencing reads were mapped to the human genome using STAR (version 2.3.0). The aligner was provided with a file containing junctions from Ensembl GRCh37.71. The Htseq-count of the Python package HTSeq (version 0.5.4p3) was used (http://www-huber.embl.de/users/anders/HTSeq/doc/overview.html) to quantify the read counts per gene based on annotation version GRCh37.71, using the default union-counting mode. Differentially expressed genes were identified by statistical analysis using the DESeq2 package. The statistically significant threshold (False Discovery Rate P < 0.05 and Fold Change ≥ 2) was applied.

### Pathway enrichment analysis

We performed gene enrichment analysis using ConsensusPathDB-human database (CPDB; http://cpdb.molgen.mpg.de/) [14]. The over-representation analysis is done using the default setting in which the database compares the predefined lists of functionally associated genes (pathways and Gene Ontology categories) to the list of differentially expressed genes and generates P values based on the hypergeometric test. The hypergeometric test P values are further corrected for multiple testing using the false discovery rate method.

### MAP3K8 inhibition of cytokines in *Candida*-stimulated PBMCs

PBMCs (5 x 10^5^) were placed in 96-well round-bottom plates in a final volume of 200μL. PBMCs were stimulated with (1 x 10^6^/mL) heat-inactivated conidia of *C*. *albicans* in the presence or absence of variable concentrations (10μM, 50μM, and 200μM) of a MAP3K8 inhibitor (#5240, Tocris Cookson Ltd., Bristol, UK) or a vehicle (DMSO) control. PBMCs were stimulated for 24 or 48 hours at 37°C and 5% CO_2_. After stimulation, culture supernatants were collected and stored at -20°C until cytokine assays were performed. Cytokine levels from *Candida*-stimulated PBMCs were measured in cell culture supernatants using enzyme-linked immunosorbent assay (ELISA) according to the manufacturer’s instructions (R&D Systems, MN, USA). Differences in cytokine levels were compared for statistical significance using the Wilcoxon rank test.

## Results

### *Candida albicans* induces transcription of genes involved in inflammation and immune-hemostasis interaction

In our experiments, we have used heat-killed *C*. *albicans* instead of live *C*. *albicans* for two main reasons. The first one is that long incubation periods of more than 48 hours to obtain the T cell derived cytokines IL-17, IL-22 and IFNγ result in overgrowth of the fungal cells and, ultimately, in cell lysis. Therefore, to achieve cell viability, we opted for heat-killed *C*. *albicans*. Secondly, the immune response is enhanced when using heat-killed *C*. *albicans* instead of live *C*. *albicans*. Heat-killing enhances the accessibility of β-glucans, whereas in live *C*. *albicans* yeast cells, β-glucans are shielded from immune recognition by a mannan layer. The β-glucans, which can be found in the inner layer of the *C*. *albicans* cell wall represent the main conserved pathogen-associated molecular patterns (PAMPs) that are recognized by one of the most important innate receptors for the recognition of *Candida* species, known as C-type lectin receptors (CLRs) [15]. The recognitions of β-glucans by CLRs constitutes the first step in the development of an immune response to *C*. *albicans*. Therefore, enhanced accessibility of β-glucans results in a pronounced immune response when using heat-killed *C*. *albicans* instead of live cells.

**Fig 1:**
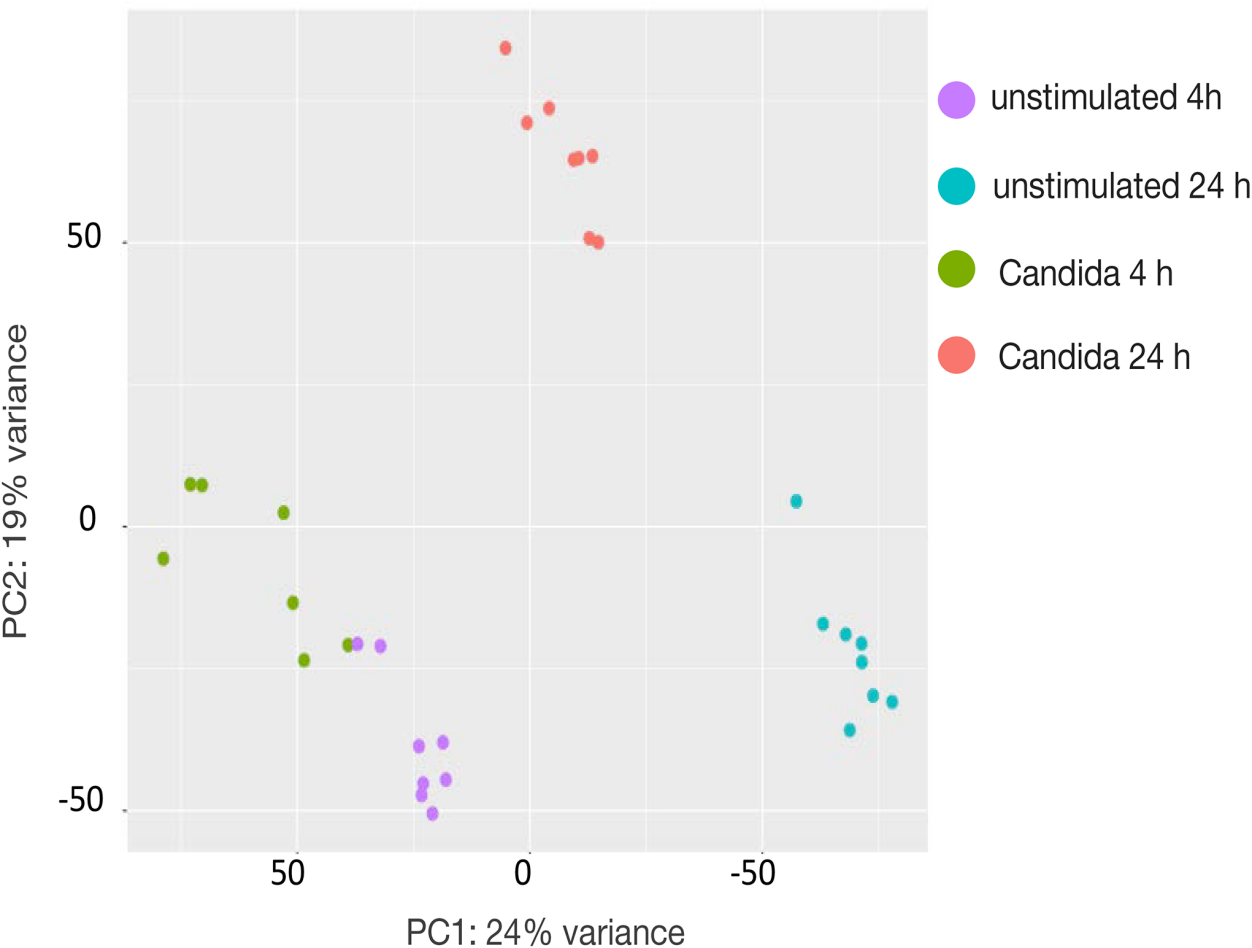
Transcriptome of PBMCs upon *Candida* stimulation. (A) Principal component analysis of RNA sequencing data obtained from eight healthy volunteers upon stimulation of their PBMCs with *C*. *albicans* for 4 or 24 hours or unstimulated. Four distinct groups were observed based on time-dependent exposure to *C*. *albicans* or culture medium alone using the first two principal components. Principal Component 1 (PC1, x-axis) represents 24% and PC2 (y-axis) represents 19% of total variation in the data. Green and red circles represent the stimulated samples with heat-killed *C*. *albicans* for 4 and 24 hours respectively and purple and blue circles represent the samples incubated with RPMI for 4 and 24 hours (used as controls) respectively.

The transcriptome of PBMCs upon *Candida* stimulation was profiled by next-generation RNA sequencing (Fig 1). A total of 312 (4 hours) and 1,476 (24 hours) protein-coding genes were identified that showed >1.5-fold higher expression in cells stimulated with *C*. *albicans* compared to unstimulated cells (adjusted P < 0.05) (S1 Table, S1A and S1B Fig). A total of 246 protein-coding genes were found to be more strongly induced both 4 h and 24 h after stimulation (S2 Table), while 66 genes showed increased expression after 4 h only and 1,230 genes were more strongly expressed after 24 h only.

**Fig 2:**
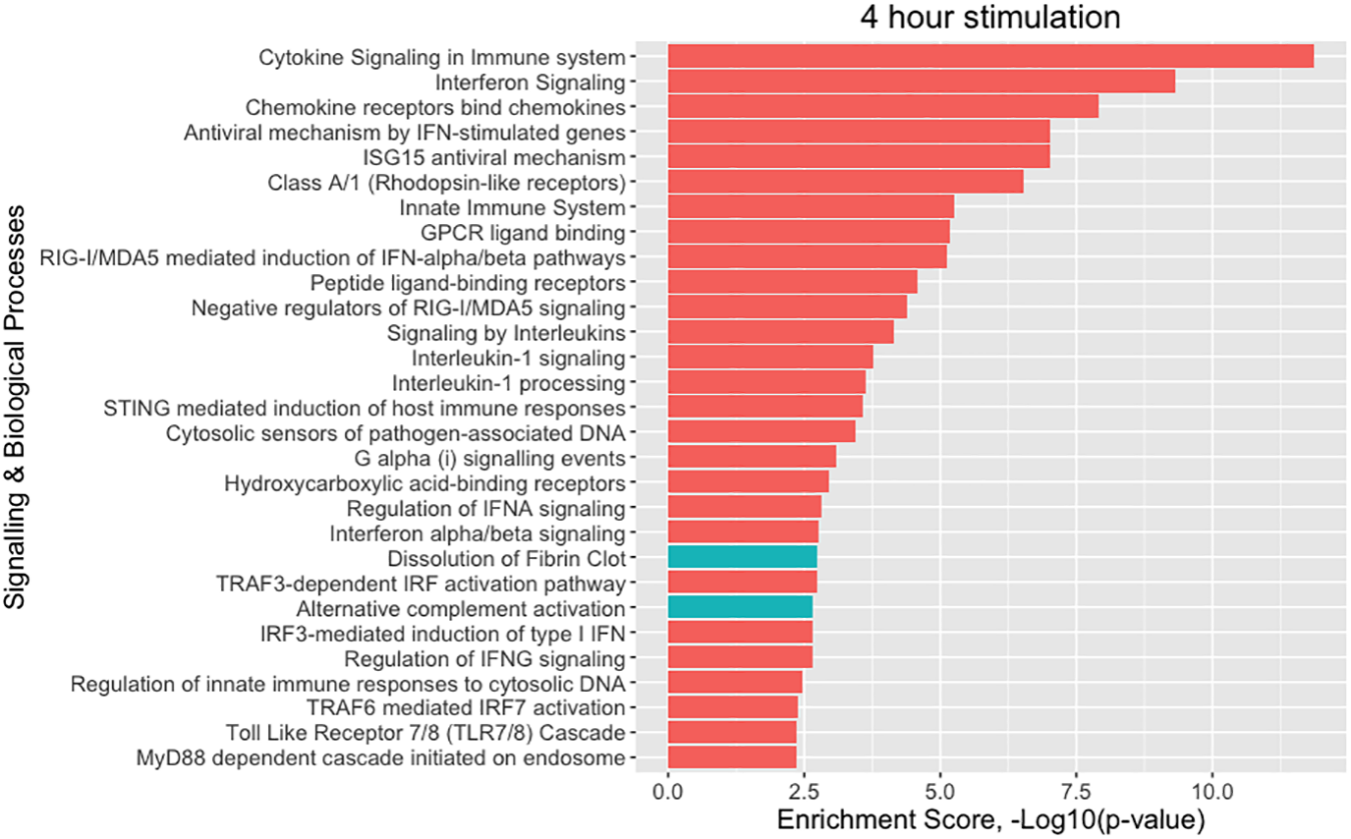
Pathway enrichment analysis of the *Candida*-induced transcripts at 4 hours. S3 Table displays all the signalling and biological processes as well as their enrichment p-values.

**Fig 3:**
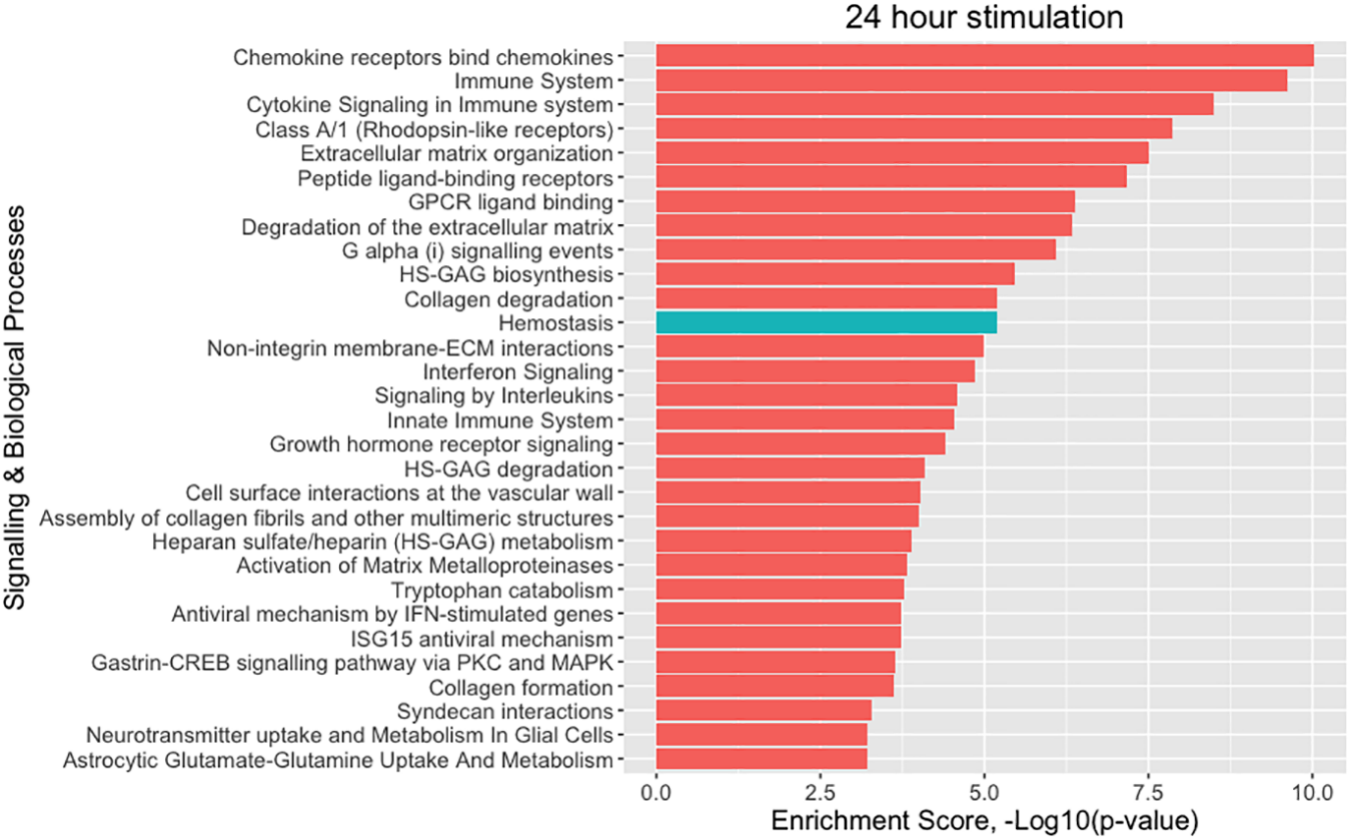
Pathway enrichment analysis of the *Candida*-induced transcripts at 24 hours reveals a stronger effect for hemostasis at 24 hours than 4 hours. S4 Table displays all the signalling and biological processes as well as their enrichment p-values.

Among the significantly differentially expressed genes, pathway enrichment analysis showed that there is an overrepresentation of genes involved in cytokine signalling and of chemokine genes at both 4 h and 24 h after stimulation. Pathway enrichment analysis on differentially expressed protein-coding genes confirmed previously described *Candida*-response pathways including cytokine signalling (4 h: P = 1.36 x 10^−12^, 24 h: P = 3.19 x 10^−9^), Toll-like-receptor-mediated signalling [16] at 4 h (P = 0.00553), interferon signalling [17] (4 h: P = 4.90 x 10^−10^, 24 h: P = 1.41 x 10^−5^) and RIG-I/MDA5 mediated induction of IFN-alpha/beta pathways (4 h: P = 7.55 x 10^−6^, 24 h: P = 0.00753) (S3 and S4 Tables) [18]. The enrichment analysis also uncovered an overrepresentation of differentially expressed genes involved in dissolution of the fibrin clot at 4 h (P = 0.0018) and 24 h (P = 0.00422), alternative complement activation at 4h (P = 0.0022), platelet activation, signalling and aggregation at 24 h (P = 0.0016) and activation of C3 and C5 -a central step of complement activation- at 24h (P = 0.00479). An even stronger effect for hemostasis (P = 6.51 x 10^−6^) was observed at 24h (Figs 2 and 3). These results suggest that, along with genes involved in cytokine and interferon signalling, genes involved in the integration of immunity and hemostasis as biological processes may also be involved in the pathogenesis of systemic *Candida* infections.

### Immunochip-based genetic study identifies 18 candidaemia susceptibility loci

After identification of the transcriptome profile induced by *Candida* in human PBMCs, we explored whether these pathways could be validated through genetic association data from a clinical candidaemia cohort of 217 patients. We had previously reported three loci to be associated to *Candida* infection at genome-wide significance (P < 5 x 10^−8^) by performing a case-control study on the Immunochip platform [9]. In addition to these three loci, during the discovery stage, we identified 77 independent loci showing suggestive associations with P values lower than 9.99 x 10^−5^. It is these independent loci that we have followed up in this study by validating them against 146 disease-matched controls to exclude confounding effects of the patient’s clinical background. Following screening of the disease-matched controls, associations at 18 independent loci could be successfully validated (P < 0.05; S2 Fig and Table 1).

**Table 1:** Prioritization of putative causal genes of candidaemia using an Immunochip-based association study.

			Discovery cohort		Candidaemia cohort				
Chr	position	SNP	P value	OR	P value	OR	Risk allele	Frequency	Gene(s)
1	19199400	rs6699706	1.01 x10^-5^	1.76	3.85 x10^-2^	1.58	C	0.11	*ALDH4A1* ^a^
1	113793315	rs11102637	5.86 x10^-6^	1.69	2.57 x10^-3^	1.84	A	0.15	*MAGI3*^b^
1	167833451	rs3766122	3.01 x10^-5^	2.02	5.03 x10^-3^	3.22	C	0.04	*F5*^a^^,^ ^c^^,^ ^d^
									*SELL*^a^^,^ ^d^
									*SELP*^d^
1	199183753	rs296537	1.57 x10^-6^	3.67	4.48 x10^-2^	4.74	A	0.01	*IGFN1*^*e*^
									*LAD1*^*e*^
2	127943178	rs6748999	8.05 x10^-6^	2.21	6.23 x10^-3^	2.78	G	0.04	*PROC*^c^^,^ ^d^^,^ ^*e*^
3	50531585	rs12491812	1.67 x10^-5^	3.24	2.48 x10^-2^	10.62	T	0.01	*CISH*^*e*^^,^^f^
5	33987450	rs16891982	2.95 x10^-5^	2.14	1.48 x10^-2^	2.66	C	0.04	*SLC45A2*^b^
6	30078406	rs11760176	9.76 x10^-5^	1.99	1.20 x10^-3^	3.74	T	0.05	*ZNRD1*^a^
									*PPP1R11*^a^
6	126875776	rs1490387	9.80 x10^-5^	0.68	9.15 x10^-3^	0.66	A	0.46	*CENPW*^b^
9	116656377	rs7022618	3.74 x10^-5^	1.64	3.74 x10^-2^	1.55	C	0.14	*TNFSF15*^*e*^
									*TNFSF8*^a^^,^ ^*e*^
9	122664970	rs72758135	6.70 x10^-5^	0.45	5.77 x10^-4^	0.401	C	0.13	*C5*^d^
									*PSMD5-AS1*^a^
									*STOM*^*e*^
10	30763712	rs1360119	3.64 x10^-7^	3.62	3.42 x10^-2^	5.13	T	0.01	*MAP3K8*^*e*^
10	54523962	rs7092540	7.76 x10^-5^	2.50	3.01 x10^-2^	3.36	A	0.02	*MBL2*^d^
12	127841061	rs59665078	7.03 x10^-5^	1.73	1.63 x10^-2^	1.85	C	0.09	*GLT1D1*^*e*^
14	94003143	rs7149309	6.55 x10^-5^	2.84	3.34 x10^-2^	5.22	T	0.01	*SERPINA1*^c^^,^ ^d^^,^ ^*e*^
									*IFI27*^*e*^
16	28909510	rs1802141	9.33 x10^-7^	3.06	2.26 x10^-2^	4.25	G	0.01	*CD19*^d^
									*LAT*^c^
									*SPNS1*^f^
									*IL27*^*e*^
17	74626807	rs3848405	1.00 x10^-6^	3.32	1.28 x10^-2^	6.62	C	0.01	*C1QTNF1*^*e*^
									*LGALS3BP*^*e*^
19	50102284	rs769450	6.63 x10^-6^	0.62	7.96 x10^-4^	0.57	G	0.4	*TOMM40*^a^
									*BCL3*^*e*^

Abbreviations: Chr, chromosome; OR, odds ratio.

^a^ Candidaemia-associated SNPs showed an eQTL effect based on publicly available eQTL datasets.

^b^ Genes in close proximity to candidaemia-associated SNP.

^c^ These genes are involved in the blood coagulation pathway.

^d^ These genes are involved in the complement system.

^e^ Differentially expressed genes in response to *Candida* stimulation.

^f^ Coding variants in the *CISH* and *SPNS1* genes were correlated with the candidaemia SNPs rs12491812 and rs1802141, respectively.

### eQTL and differential expression prioritize candidaemia causal genes enriched for inflammation and hemostasis

**Fig 4:**
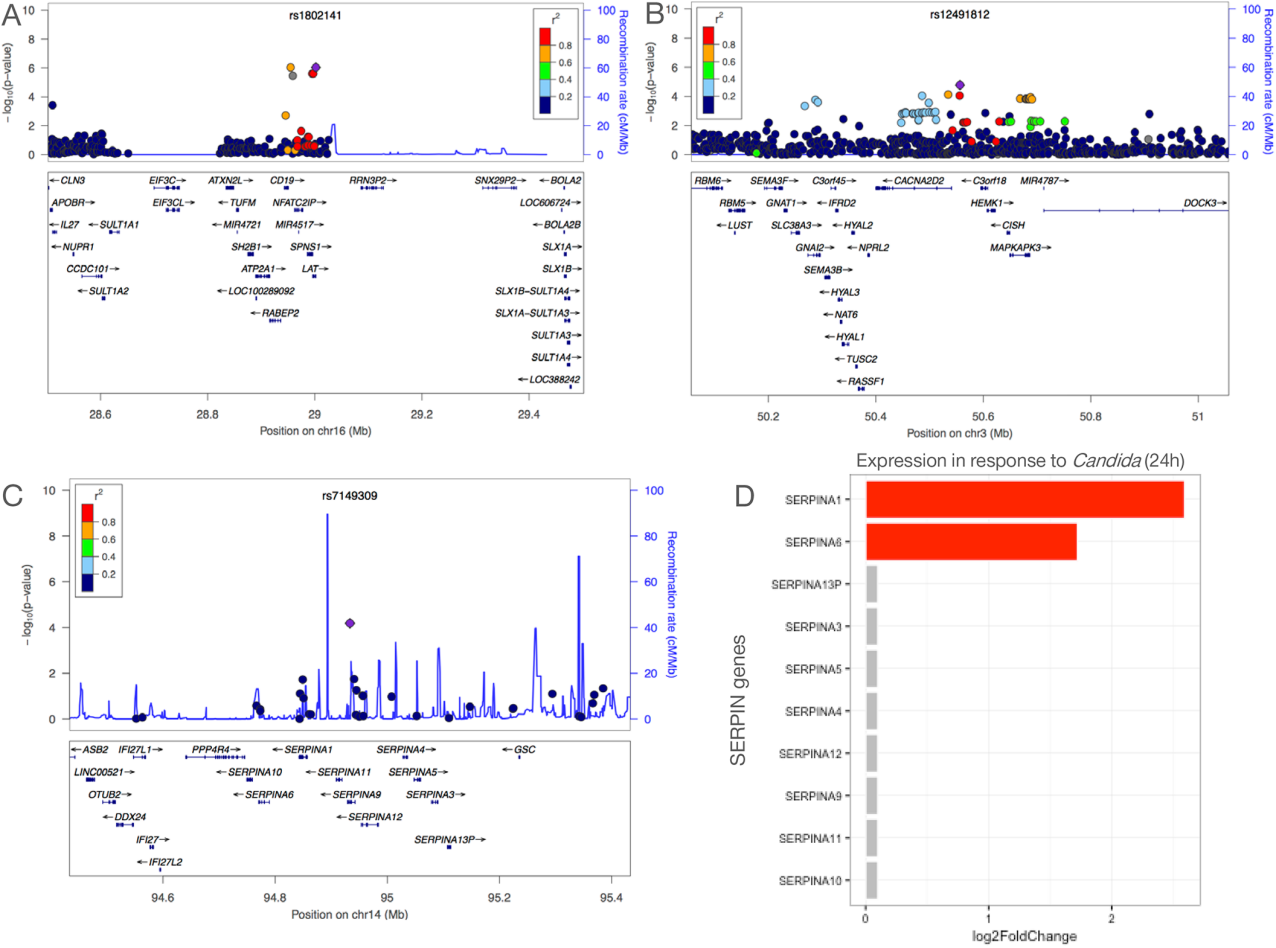
Prioritization of candidaemia genes enriched for blood coagulation based on eQTL and differential expression data. Regional association plots are shown around candidaemia-associated SNPs identified in the Discovery cohort. (A) rs1802141 (P = 9.325 x 10^−7^) on chromosome 16 is in strong LD with the synonymous variant rs61747536 (R^2^ = 0.82, D’ = 1) in the *SPNS1* gene and (B) rs12491812 (P = 1.67 x 10^−5^) on chromosome 3 is in strong LD (R^2^ = 0.89, D’ = 1) with two synonymous variants, rs2239753 and rs2239752, in the *CISH* gene. SNPs are plotted as the–log10 of the p-value. Local LD structure is reflected by the plotted estimated recombination rates (from HapMap) in the region around the associated SNP (purple diamond) and its correlated proxies. The correlation of the lead SNP to other SNPs at the locus is indicated by colour. (C) Regional association plot for rs7149309, which is located in a locus on chromosome 14, that harbors a cluster of serine protease inhibitor genes of the serpin family, with *SERPINA1* being the most likely causal gene at this locus based on our expression data upon *Candida* stimulation. (D) Log2 fold change expression levels of all *SERPIN* genes upon *Candida* stimulation for 24 hours at a locus on chromosome 14 marked by the candidaemia-associated SNP rs7149309. *SERPINA1* showed a log2 fold change of ~2.6.

To identify causal genes from these 18 susceptibility loci, three different approaches were applied. In our first approach, we tested whether the 18 candidaemia SNPs were in linkage disequilibrium (LD) with variants that alter the protein-coding sequence of the gene (both synonymous and non-synonymous). Two candidaemia-associated SNPs were identified, rs1802141 and rs12491812, that were in strong LD with synonymous variants (S5 Table) and therefore may point to putative causal genes. SNP rs1802141 on chromosome 16 is in strong LD (R^2^ = 0.82, D’ = 1) with a synonymous variant, rs61747536, in the *SPNS1* gene (Fig 4A). The *SPNS1* gene, also known as *HSpin1*, has been described to induce a caspase-independent autophagic cell death in cultured human cells [19]. SNP rs12491812 on chromosome 3 is in strong LD (R^2^ = 0.89, D’ = 1) with two synonymous variants, rs2239753 and rs2239752, in the *CISH* gene (Fig 4B). The *CISH* locus has been associated with major infectious diseases such as bacteraemia, tuberculosis, malaria [20], and viral infections such as hepatitis B [21,22], suggesting shared susceptibility genes among different infections.

We should mention that the majority of candidaemia-associated SNPs and proxies fall within intergenic/intronic regions, suggesting that they could have a functional potential by affecting gene expression (S5 Table). Therefore, in our second approach, the 18 candidaemia-associated SNPs were mapped for *cis*-eQTLs using publicly available eQTL datasets from healthy blood donor samples [23–26]. *Cis*-eQTL mapping pinpointed 8 potential causal genes at 6 loci (Table 1).

In our third approach, genes that were located within a 500 kb window around the 18 candidaemia-associated SNPs were extracted and tested as to whether they are differentially expressed after *Candida* stimulation for 4 and 24 hours (S6 and S7 Tables). Within these ‘susceptibility regions’, we found 18 and 28 genes that are significantly induced upon *Candida*-stimulation for 4 and 24 hours respectively in human PBMCs, raising the possibility that some of these genes could be regulated by the candidaemia-associated SNPs in the context of *Candida* infection. For example, one of the 18 candidaemia-associated SNPs, rs7149309, is located within a locus on chromosome 14 that harbours a cluster of ten serine protease inhibitor genes of the serpin family (Fig 4C). Serpin Family A Member 1 (*SERPINA1*) was significantly expressed in PBMCs in response to *Candida* stimulation for 24 hours compared to the other *SERPIN* genes in the same locus (Fig 4D). *SERPINA1* encodes human alpha-1 antitrypsin (hAAT). A protective role of AAT has been demonstrated against different types of infectious pathogens, such as in bacterial peritonitis [27] and pulmonary *Pseudomonas aeruginosa* infection in cystic fibrosis patients [28].

Together, our three approaches prioritized 31 putative candidate genes for candidaemia susceptibility located in 18 loci (Table 1). Intriguingly, nine out of these 31 prioritized genes (*LAT*, CD19, *F5*, *PROC*, *C5*, *SERPINA1*, *SELP*, *SELL* and *SELE*) are enriched in the processes of complement and blood coagulation (Table 1, S3 Fig and S8 Table). As expected, pathway enrichment analysis for all 31 candidaemia genes showed enrichment for cytokine- and immune-related signalling pathways, which is in agreement with our pathway enrichment analysis of the *Candida*-induced transcripts at 4 and 24 hours (Figs 2 and 3, S3 and S4 Tables). In three out of 18 loci, genes that are located in close proximity to the top SNP were shown (Table 1), as we did not find any evidence from the above three approaches to prioritize causal genes.

### *MAP3K8* modulates cytokine production in patients and in experimental models

**Fig 5:**
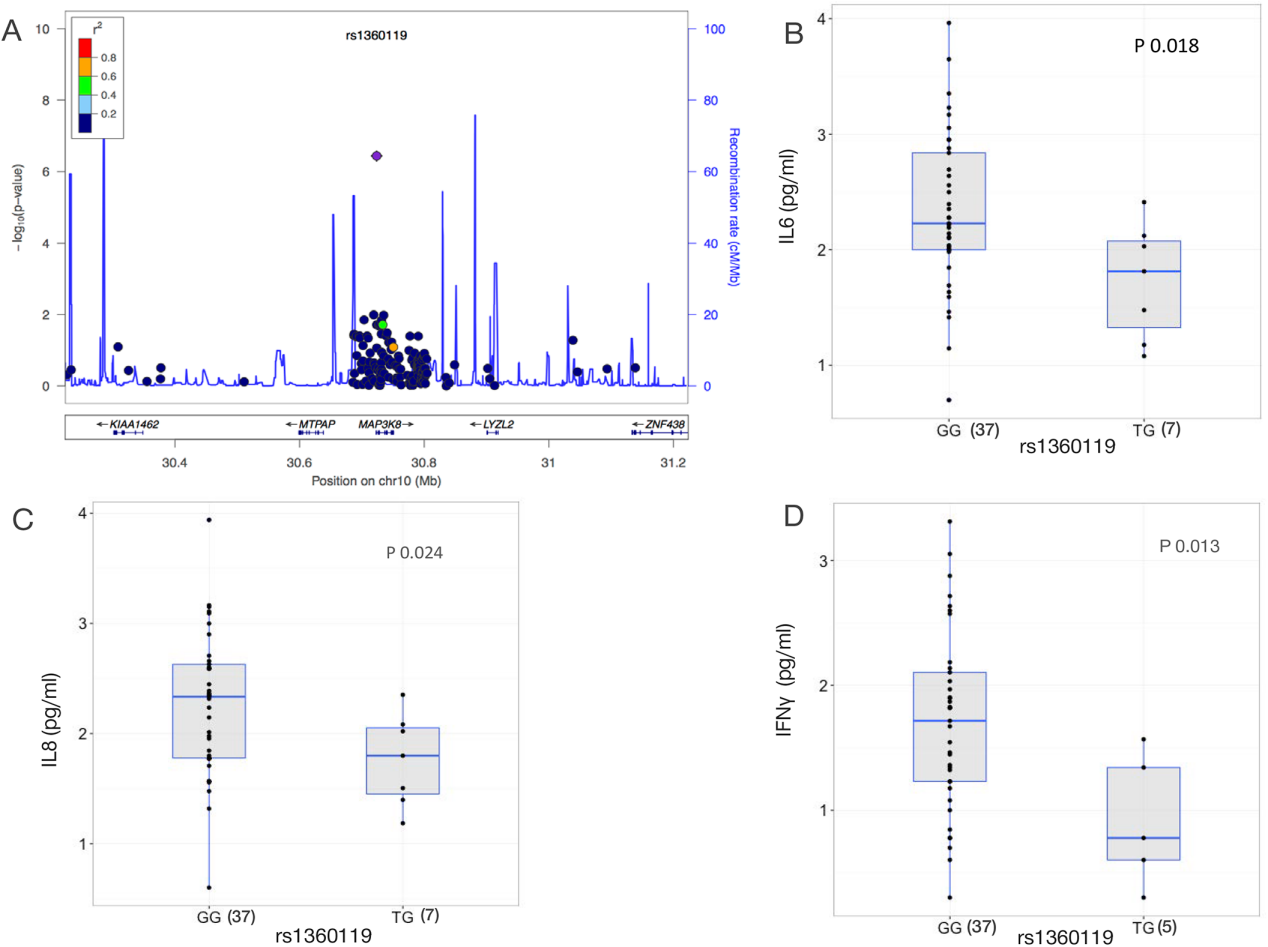
Candidaemia-associated SNP rs1360119 affects the cytokine expression in serum from patients. (A) Regional association plot for candidaemia-associated SNP rs1360119, which was mapped to *MAP3K8* locus on chromosome 10. (B) Genotypes of candidaemia-associated SNP rs1360119 are consistently associated with expression levels of (B) IL6 (P = 0.018), (C) IL8 (P = 0.024) and (D) IFNɣ (P = 0.013) as measured in serum of candidaemia patients. The numbers of individuals per genotype are shown in parentheses. Cytokine levels were log transformed and P values were obtained using Kruskal Wallis test, and 0.05 was considered statistically significant (minor allele frequency of A = 2% in Europeans from 1000 Genomes Project Phase 3, http://www.internationalgenome.org).

One of the candidaemia-associated SNPs, rs1360119, was mapped to the Mitogen-Activated Protein Kinase Kinase 8 (*MAP3K8*) locus (Fig 5A). In particular, rs1360119 is an intronic variant, suggesting that it may affect gene expression. However, rs1360119 does not show *cis*-eQTL effect using publicly available eQTL datasets from healthy blood donor samples (Table 1). That does not exclude the fact that this SNP may show an eQTL effect under context-specific conditions, for instance, upon *Candida* stimulation. In addition, an intronic variant can have functional effects on splicing and, therefore, we can speculate that this SNP may affect splicing.

Of note, MAP kinases are notably attractive therapeutic targets [29]. Therefore, considering the regulatory role of MAP3K8 protein in the production of cytokines in response to lipopolysaccharide [30], bacterial [31,32], and viral pathogens [33], we tested whether the rs1360119 SNP correlates with levels of pro-inflammatory cytokines in serum of candidaemia patients. The T allele of the SNP rs1360119 is associated with decreased IL-6 (P = 0.018), IL-8 (P = 0.024), and interferon (IFNγ) (P = 0.013) cytokine levels (Figs 5B–5D). Five other candidaemia-associated SNPs showed a moderate association (P < 0.05) with circulating cytokine levels (S9 Table), suggesting that some of the candidaemia-associated variants could determine susceptibility to candidaemia by influencing cytokine production capacity.

**Fig 6:**
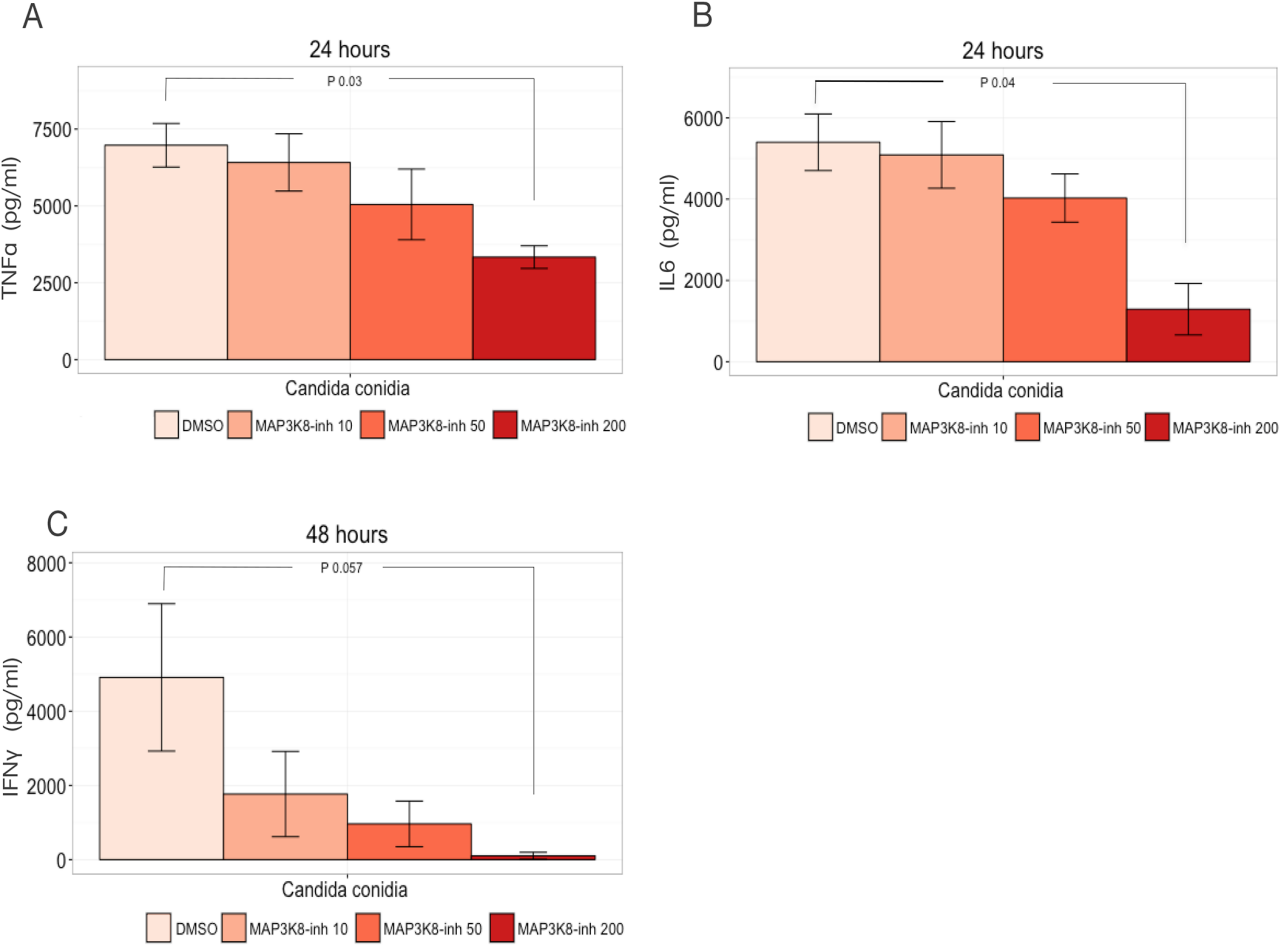
*MAP3K8* modulates cytokine production in PBMCs. Median expression levels of (A) TNFα (B) IL6 and (C) IFNγ in *Candida*-stimulated PBMCs upon inhibition of MAP3K8 at three different concentrations (10 μM, 50 μM, and 200 μM). *Candida conidia* was added at a concentration of 1 x 10^6^/ml. P values were obtained using Wilcoxon rank test (*P < 0.05). Data shown are from two independent experiments of PBMC stimulation for 24 and 48 hours with conidia of *C*. *albicans*.

To test whether *MAP3K8* is involved in regulating *Candida*-induced cytokine production, we measured cytokine levels upon stimulation of PBMCs with *C*. *albicans* in the presence or absence of a MAP3K8 chemical inhibitor. Three different cytokines (TNFα, IL-6, and IFNγ) known to be involved in host defence against *C*. *albicans* were measured [15]. MAP3K8 activity was blocked using three different concentrations of the MAP3K8 inhibitor (10 μM, 50 μM, and 200 μM). Overall, we observed a dose-dependent reduction in all three cytokine levels upon increasing concentration of MAP3K8 inhibitor. A significant decrease in *Candida-*induced TNFα production was observed compared to the DMSO-control at 200 μM of *MAP3K8* inhibitor (P = 0.03) upon 24-hour stimulation (Fig 6A). In addition, a significant decrease in IL-6 was observed upon stimulation with *Candida* conidia for 24 hours with 200 μM *MAP3K8* inhibitor compared to DMSO (P = 0.04) (Fig 6B). We should note that IFNγ, one of the most crucial cytokines for efficient host defence for systemic candidiasis, decreased compared to DMSO after stimulation with *Candida* conidia (P = 0.057) for 48 hours at 200 μM of *MAP3K8* inhibitor (Fig 6C). Overall, these experiments demonstrate that *MAP3K8* regulates cytokine levels upon *Candida* infection.

## Discussion

Although *Candida spp* are the fourth most common cause of sepsis in the US, and the most common cause of fungal sepsis in Europe, the difficulty of recruiting sufficient numbers of patients has prevented the use of large GWAS studies to reveal the genetic factors involved in the pathophysiology of *Candida* infections. Our study demonstrates the potential of using a systems genomics approach to obtain novel insights into the genetic basis and host defence mechanisms involved in relatively rare infections in which classical GWAS studies are not applicable. By integrating transcriptomic, genetic and immunological studies, we have identified novel susceptibility genes for candidaemia and new potential therapeutic targets.

The first major group of pathways that are induced during stimulation of human PBMCs with *C*. *albicans* are genes important for inflammation and innate immunity, cytokine and chemokine synthesis, interferon and inflammasome signalling. This is not surprising as these pathways have been previously shown to have a crucial role for antifungal host defence [15]. The importance of inflammation and innate immunity was also validated by genetic studies that confirmed that the genes determining susceptibility to candidaemia were enriched for inflammatory genes. However, our analysis has also provided new insight in additional processes that apparently have an important impact on candidaemia. One of these processes is complement activation, which has been shown to modulate cytokine production induced by *Candida* stimulation [34].

Hemostasis is another important biological process suggested by our study to be involved in susceptibility to candidaemia. Hemostasis is known to be strongly activated during sepsis and to interact with the inflammatory responses [35–38]. Strong interactions between immune defence and hemostasis are well documented for bacterial infections, and hemostasis has been previously associated with an increased susceptibility for bacterial sepsis [37,39,40]. Some of the hemostasis genes that we identified as candidaemia susceptibility genes (*SERPINA1*, *LAT* and *F5*) have also been shown to be important for bacterial sepsis (S4A Fig) [41]. Future studies should examine the involvement of these pathways and mechanisms in systemic *Candida* infection, especially as platelets contain several anti-fungal defensins [42], indicating their potential involvement in candidaemia. For example, an examination of protein-protein interaction data using the Plateletweb database found that 14 of our candidaemia susceptibility proteins were found in platelets (S4B Fig).

Another novel observation of the present study is the association of *SERPINA1* gene with susceptibility to candidaemia. Mutations in this gene lead to AAT deficiency and predispose individuals to chronic obstructive pulmonary disease and liver diseases [43,44]. Considering the important role of AAT in modulation of inflammation, as well as the recent beneficial effect of human AAT against bacterial infections [45], hAAT could represent a potential novel adjuvant immunotherapy against systemic *Candida* infection.

*MAP3K8* is known as a critical gene in innate immune responses linking pattern recognition receptors such as Toll-like receptors (TLRs) to TNF production through activation of extracellular signal-regulated kinase [30,46]. In this study we also demonstrate that *MAP3K8* plays an important role in *Candida* infection by modulating cytokine production. The link of *MAP3K8* with TLR-signalling suggests that the innate immune response is important in the context of *Candida* infection. In particular, non-synonymous SNPs in TLR1 have previously been associated with increased susceptibility to candidaemia, highlighting the role of TLRs in the recognition of *Candida* species [47]. Furthermore, the critical role of *MAP3K8* in host immune defence has been demonstrated for other infectious pathogens including influenza virus [33] and *Listeria monocytogenes* [32]. Most importantly, MAP3K8 is an important and novel therapeutic target for inflammatory diseases [48]. A recent computational approach using publicly available transcriptome datasets for the discovery of common immunomodulators in fungal infections also pinpointed *MAP3K8* and *SERPINE1* in the top ten consistently perturbed gene sets [49]. However, further functional studies are needed to shed light on the role of these genes in host defence against *Candida* infection and to understand their potential as therapeutic targets.

Several limitations also apply to this study. First, the patient cohort study is relatively small, which limited our power to identify many of the genetic factors influencing susceptibility to candidaemia. Second, we lack genetic validation in an independent cohort of patients. This is because no such cohort is currently available: the cohort studied here is the largest candidaemia cohort currently available. In addition, a potential limitation of the present study is that the Immunochip platform covers only 5% of the human genome, i.e. we still lack genome-wide information. This means that further critical genetic variations for candidaemia remain to be discovered on a genome-wide scale. Furthermore, it should be mentioned that *C*.*albicans* is a polymorphic fungus and is encountered either as yeast or hyphal forms. The transition between conidia and hyphae is a virulence trait of *C*. *albicans* and mutants that are locked in the yeast form are less virulent in experimental models of disseminated candidiasis [50,51]. Differential cytokine expression and excretion by immune cells may explain the increased invasiveness of hyphae. For instance, hyphae form has been shown to be unable to induce IFNγ in either human PBMCs or murine splenic lymphocytes and conidia induced a much higher TNFα production than hyphae did [52]. This differential cytokine expression may be attributed to structural differences in the cell wall between yeast and hyphal forms and therefore, the human innate immune system can discriminate between yeast and hyphae [53]. In addition, the accessibility of different pathogen associated molecular patterns (PAMPs) between hyphae and conidia of *C*. *albicans* as well as the potential of only hyphae to activate the inflammasome can explain the induction of immune responses that discriminate between conidia and hyphae forms [53,54]. Thus, by using only heat-killed *C*. *albicans* conidia in the present study may not represent the full physiological conditions in humans, and further studies should address the differential effect of both forms in PBMCs. Last, *C*. *albicans* strains may vary in pathogenicity and, therefore, may elicit different host immune responses making it interesting to consider more clinical strains (in addition to UC 820) for validation in the future.

To conclude, the application of an unbiased, hypothesis-free, systems integrative genomics approach has the power to identify novel susceptibility genes for infectious diseases such as systemic candidiasis. In this study, this approach highlighted genes in inflammation, innate immunity and hemostasis pathways that contribute to the genetic susceptibility against candidaemia. We therefore believe that such integrative approaches are an important tool for future identification of genetic susceptibility to rare infectious diseases.

## Supporting information

S1 Fig Heatmaps showing the expression of protein-coding genes, which showed >1.5-fold higher expression, upon (A) 4 and (B) 24-hour stimulation with *C*. *albicans* in PBMCs from healthy volunteers. RPMI medium was used as control. (adjusted P < 0.05).
(TIF)

S2 Fig Immunochip-wide association analysis with candidaemia. Manhattan plot highlighting the 18 independent loci showing suggestive association with candidaemia (P < 9.99 x 10^−5^) using a second set of case-matched controls. The y-axis represents the–log_10_P values of 122,779 SNPs. Their chromosomal positions are shown on the x axis. The dotted line represents the suggestive threshold for association (P < 9.99 X 10^−5^). P values were not corrected for multiple testing when testing for association with candidaemia susceptibility at 18 independent loci identified in the discovery stage.
(TIF)

S3 Fig Pathway enrichment analysis based on KEGG and Reactome sources of all 31 candidaemia genes prioritized based on eQTL, differential expression upon *Candida* stimulation and proximity to top SNP. Candidaemia genes showed an expected enrichment for cytokine signalling pathways and showed a strong enrichment for complement and coagulation pathways. Each node represents a separate pathway whose number of genes and P-value are encoded as node size and node colour, respectively. Two nodes are connected by an edge if they share members. The edge width reflects the relative overlap (corresponding to the Fowlkes-Mallows index) between the nodes, while the edge colour encodes the number of shared gene members. (see.tif image)
(TIF)

S4 Fig (A) Heatmap depicts the false discovery rate (FDR) of differentially expressed genes in sepsis as identified by *Davenport E et al* in their discovery and validation cohort. These genes were differentially expressed in response to *Candida* stimulation as well. (B) Proteins encoded by 14 candidaemia susceptibility genes detected in platelets using plateletWeb (http://plateletweb.bioapps.biozentrum.uni-wuerzburg.de/plateletweb.php). (see.tiff image)
(TIF)

S1 Table Differentially expressed protein-coding genes in response to 4 and 24 hour-*Candida* stimulation that showed >1.5-fold higher expression compared to RPMI medium used as control. (DOCX)

S2 Table A total of 246 protein-coding genes were differentially expressed at both 4 and 24 hour-*Candida* stimulation, showing >1.5-fold higher expression compared to RPMI medium used as control. (DOCX)

S3 Table Pathway enrichment analysis on differentially expressed protein-coding genes that showed >1.5 higher expression compared to RPMI medium in response to 4 hour-*Candida* stimulation. (DOCX)

S4 Table Pathway enrichment analysis on differentially expressed protein-coding genes that showed >1.5 higher expression compared to RPMI medium in response to 24 hour-*Candida* stimulation. (DOCX)

S5 Table Candidaemia-associated SNPs and variants with r^2^> = 0.8. SNPs rs12491812 and rs1802141 were in strong linkage disequilibrium (LD) with synonymous variants located in *CISH* and *SNPS1* genes respectively. (source: Haploreg http://archive.broadinstitute.org/mammals/haploreg/haploreg.php).
(DOCX)

S6 Table Differential expression of genes that are located within a 500 kilobase (kb) window around the candidaemia-associated SNPs upon *Candida* stimulation at 4 hours. Bolded genes show a log2 fold change > 1.5.
(DOCX)

S7 Table Differential expression of genes that are located within a 500 kilobase (kb) window around the candidaemia-associated SNPs upon *Candida* stimulation at 24 hours. Bolded genes show a log2 fold change > 1.5.
(DOCX)

S8 Table All thirty-one prioritized susceptibility genes for candidaemia showed a strong enrichment for complement and coagulation pathways along with cytokine- and immune- related pathways based on KEGG and Reactome sources. (DOCX)

S9 Table Five additional candidaemia SNPs showed a moderate association with circulating cytokine levels as measured in serum from candidaemia patients. Cytokine levels were log transformed and statistical significance was tested with Kruskal Wallis test.
(DOCX)
