# Supplementary figures and images for: An integrative genomics approach identifies novel pathways that influence candidaemia susceptibility
Source: PLoS One. 2017 Jul 20;12(7):e0180824. doi: 10.1371/journal.pone.0180824 (PMC5519064; doi:10.1371/journal.pone.0180824)

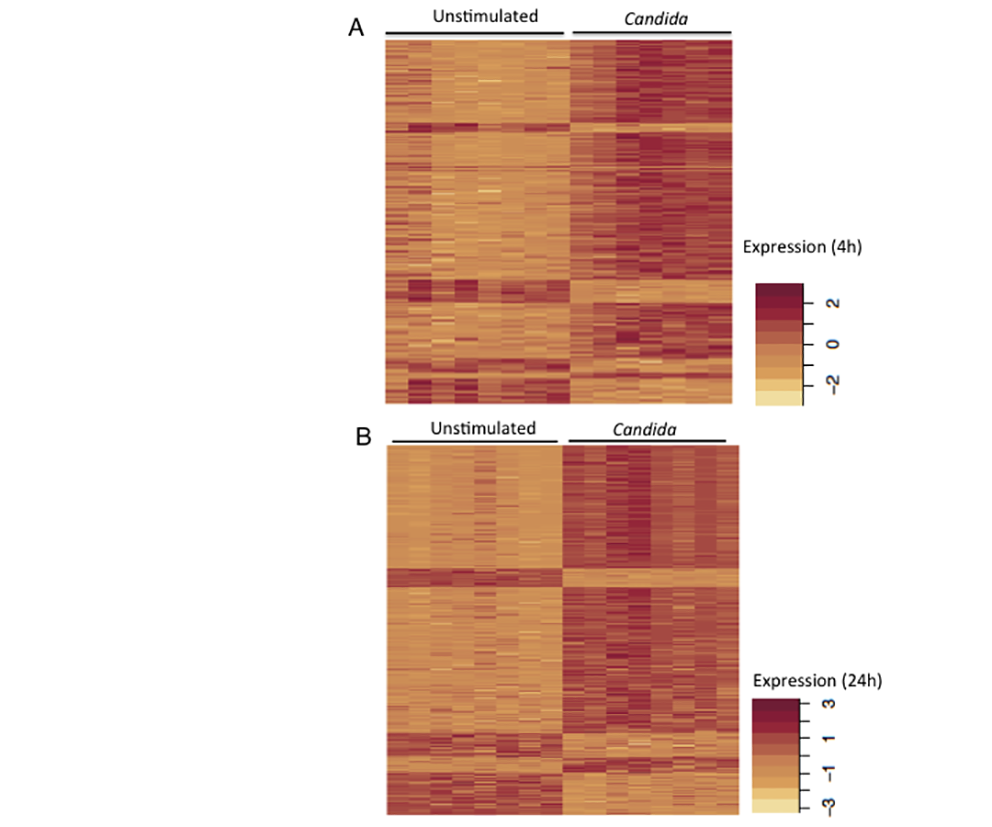

Supplement: S1 Fig — Heatmaps showing the expression of protein-coding genes, which showed >1.5-fold higher expression, upon (A) 4 and (B) 24-hour stimulation with C. albicans in PBMCs from healthy volunteers. RPMI medium was used as control. (adjusted P < 0.05). (TIF) [file pone.0180824.s001.tif]

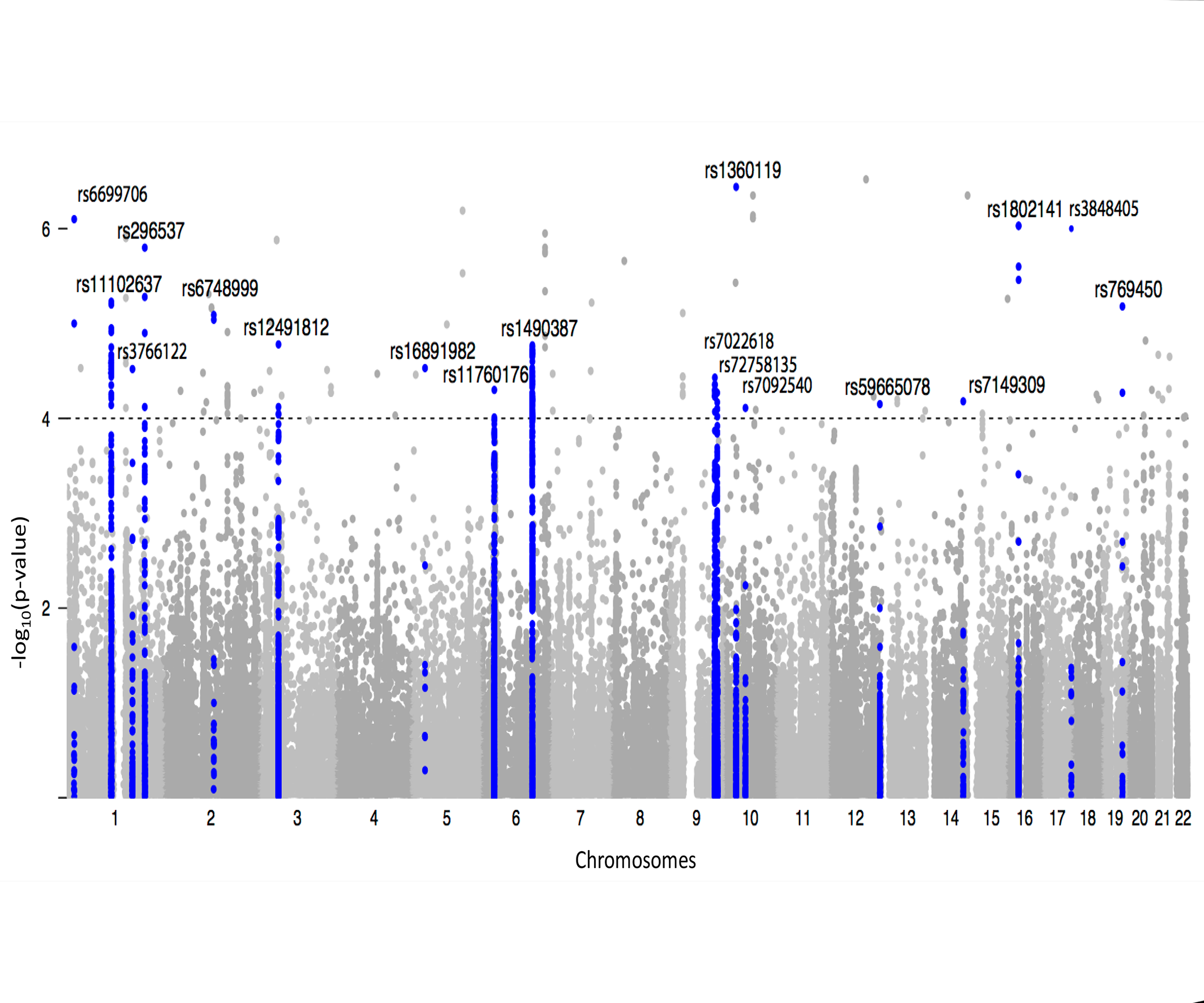

Supplement: S2 Fig — Manhattan plot highlighting the 18 independent loci showing suggestive association with candidaemia (P < 9.99 x 10^−5^) using a second set of case-matched controls. The y-axis represents the–log10P values of 122,779 SNPs. Their chromosomal positions are shown on the x axis. The dotted line represents the suggestive threshold for association (P < 9.99 X 10−5). P values were not corrected for multiple testing when testing for association with candidaemia susceptibility at 18 independent loci identified in the discovery stage. (TIF) [file pone.0180824.s002.tif]

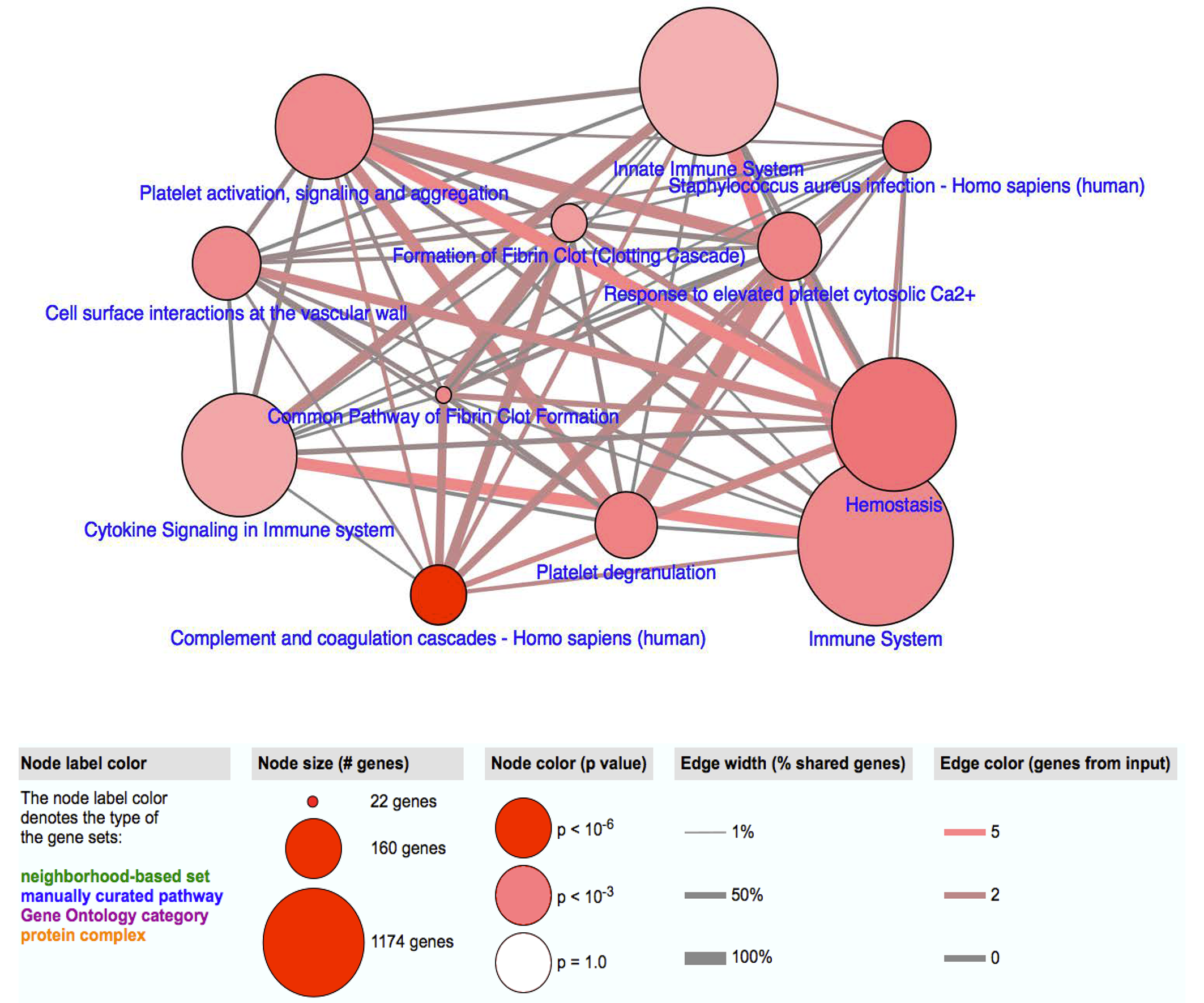

Supplement: S3 Fig — Candidaemia genes showed an expected enrichment for cytokine signalling pathways and showed a strong enrichment for complement and coagulation pathways. Each node represents a separate pathway whose number of genes and P-value are encoded as node size and node colour, respectively. Two nodes are connected by an edge if they share members. The edge width reflects the relative overlap (corresponding to the Fowlkes-Mallows index) between the nodes, while the edge colour encodes the number of shared gene members. (see.tif image) (TIF) [file pone.0180824.s003.tif]

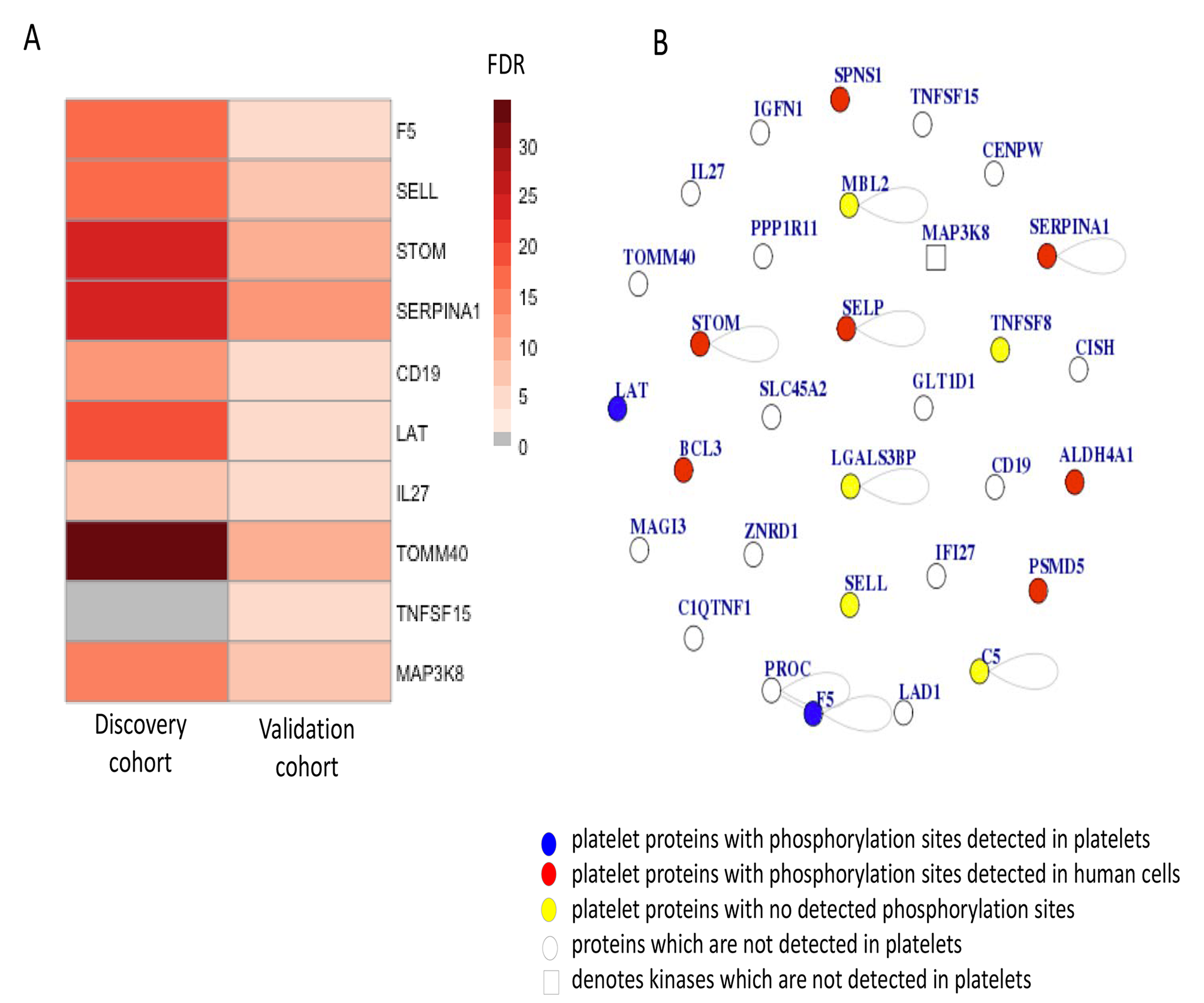

Supplement: S4 Fig — (A) Heatmap depicts the false discovery rate (FDR) of differentially expressed genes in sepsis as identified by Davenport E et al in their discovery and validation cohort. These genes were differentially expressed in response to Candida stimulation as well. (B) Proteins encoded by 14 candidaemia susceptibility genes detected in platelets using plateletWeb (http://plateletweb.bioapps.biozentrum.uni-wuerzburg.de/plateletweb.php). (see.tiff image) (TIF) [file pone.0180824.s004.tif]
