# Supplementary material for: An integrative genomics approach identifies novel pathways that influence candidaemia susceptibility
Source: PLoS One. 2017 Jul 20;12(7):e0180824. doi: 10.1371/journal.pone.0180824 (PMC5519064; doi:10.1371/journal.pone.0180824)
Supplement: S1 Table — (DOCX) [file pone.0180824.s005.docx]

Table S1. Differentially-expressed protein-coding genes in response to 4 and 24 hour-*Candida* stimulation that showed >1.5-fold higher expression compared to RPMI medium used as control.

| Ensembl ID | Log2FoldChange | P adjusted | Gene | Biotype | Stimulation time (hours) |
| --- | --- | --- | --- | --- | --- |
| ENSG00000114737 | 1.62 | 7.92E-14 | CISH | Protein coding | 4 |
| ENSG00000197272 | 2.21 | 1.11E-11 | IL27 | Protein coding | 4 |
| ENSG00000159166 | 2.32 | 2.02E-11 | LAD1 | Protein coding | 4 |
| ENSG00000107968 | 1.68 | 1.67E-23 | MAP3K8 | Protein coding | 4 |
| ENSG00000136244 | 7.22 | 0 | IL6 | Protein coding | 4 |
| ENSG00000113302 | 6.88 | 4.91E-88 | IL12B | Protein coding | 4 |
| ENSG00000115009 | 6.25 | 2.01E-233 | CCL20 | Protein coding | 4 |
| ENSG00000117525 | 6.19 | 0 | F3 | Protein coding | 4 |
| ENSG00000006075 | 6.16 | 5.22E-124 | CCL3 | Protein coding | 4 |
| ENSG00000232810 | 5.95 | 2.66E-110 | TNF | Protein coding | 4 |
| ENSG00000115008 | 5.77 | 5.34E-78 | IL1A | Protein coding | 4 |
| ENSG00000136688 | 5.21 | 7.96E-51 | IL36G | Protein coding | 4 |
| ENSG00000108342 | 5.15 | 2.56E-46 | CSF3 | Protein coding | 4 |
| ENSG00000129277 | 4.96 | 8.86E-64 | CCL4 | Protein coding | 4 |
| ENSG00000205021 | 4.77 | 2.68E-39 | CCL3L1 | Protein coding | 4 |
| ENSG00000125538 | 4.73 | 2.72E-150 | IL1B | Protein coding | 4 |
| ENSG00000137869 | 4.73 | 2.22E-43 | CYP19A1 | Protein coding | 4 |
| ENSG00000205020 | 4.71 | 4.73E-53 | CCL4L1 | Protein coding | 4 |
| ENSG00000073756 | 4.68 | 9.19E-137 | PTGS2 | Protein coding | 4 |
| ENSG00000184979 | 4.66 | 1.89E-135 | USP18 | Protein coding | 4 |
| ENSG00000165474 | 4.56 | 1.47E-40 | GJB2 | Protein coding | 4 |
| ENSG00000163739 | 4.51 | 1.98E-45 | CXCL1 | Protein coding | 4 |
| ENSG00000132669 | 4.44 | 7.11E-156 | RIN2 | Protein coding | 4 |
| ENSG00000050730 | 4.34 | 1.37E-129 | TNIP3 | Protein coding | 4 |
| ENSG00000197262 | 4.31 | 6.06E-42 | CCL4L2 | Protein coding | 4 |
| ENSG00000185745 | 4.24 | 1.69E-51 | IFIT1 | Protein coding | 4 |
| ENSG00000140519 | 4.21 | 1.22E-28 | RHCG | Protein coding | 4 |
| ENSG00000134321 | 4.13 | 1.04E-46 | RSAD2 | Protein coding | 4 |
| ENSG00000123689 | 4.12 | 8.23E-102 | G0S2 | Protein coding | 4 |
| ENSG00000081041 | 3.90 | 5.76E-37 | CXCL2 | Protein coding | 4 |
| ENSG00000181634 | 3.89 | 7.72E-72 | TNFSF15 | Protein coding | 4 |
| ENSG00000177535 | 3.83 | 2.09E-41 | OR2B11 | Protein coding | 4 |
| ENSG00000108691 | 3.81 | 3.04E-26 | CCL2 | Protein coding | 4 |
| ENSG00000122641 | 3.81 | 5.40E-29 | INHBA | Protein coding | 4 |
| ENSG00000134326 | 3.79 | 3.69E-46 | CMPK2 | Protein coding | 4 |
| ENSG00000157601 | 3.79 | 3.27E-90 | MX1 | Protein coding | 4 |
| ENSG00000111537 | 3.68 | 8.05E-21 | IFNG | Protein coding | 4 |
| ENSG00000119917 | 3.68 | 6.29E-38 | IFIT3 | Protein coding | 4 |
| ENSG00000111331 | 3.64 | 6.76E-137 | OAS3 | Protein coding | 4 |
| ENSG00000137959 | 3.61 | 3.69E-148 | IFI44L | Protein coding | 4 |
| ENSG00000154099 | 3.60 | 4.19E-44 | DNAAF1 | Protein coding | 4 |
| ENSG00000108700 | 3.59 | 4.48E-19 | CCL8 | Protein coding | 4 |
| ENSG00000169429 | 3.59 | 1.95E-104 | IL8 | Protein coding | 4 |
| ENSG00000163734 | 3.59 | 3.13E-32 | CXCL3 | Protein coding | 4 |
| ENSG00000088827 | 3.53 | 6.48E-58 | SIGLEC1 | Protein coding | 4 |
| ENSG00000078401 | 3.52 | 2.81E-65 | EDN1 | Protein coding | 4 |
| ENSG00000164400 | 3.48 | 2.09E-24 | CSF2 | Protein coding | 4 |
| ENSG00000163661 | 3.44 | 4.48E-34 | PTX3 | Protein coding | 4 |
| ENSG00000163735 | 3.42 | 5.16E-36 | CXCL5 | Protein coding | 4 |
| ENSG00000162711 | 3.41 | 1.02E-293 | NLRP3 | Protein coding | 4 |
| ENSG00000187608 | 3.40 | 3.22E-30 | ISG15 | Protein coding | 4 |
| ENSG00000126709 | 3.37 | 6.74E-32 | IFI6 | Protein coding | 4 |
| ENSG00000123610 | 3.35 | 6.07E-22 | TNFAIP6 | Protein coding | 4 |
| ENSG00000126262 | 3.34 | 2.85E-25 | FFAR2 | Protein coding | 4 |
| ENSG00000105855 | 3.32 | 2.42E-55 | ITGB8 | Protein coding | 4 |
| ENSG00000105707 | 3.29 | 1.01E-23 | HPN | Protein coding | 4 |
| ENSG00000167236 | 3.28 | 4.47E-18 | CCL23 | Protein coding | 4 |
| ENSG00000197632 | 3.26 | 4.45E-90 | SERPINB2 | Protein coding | 4 |
| ENSG00000157227 | 3.22 | 4.60E-163 | MMP14 | Protein coding | 4 |
| ENSG00000137965 | 3.19 | 4.36E-66 | IFI44 | Protein coding | 4 |
| ENSG00000184221 | 3.19 | 1.69E-50 | OLIG1 | Protein coding | 4 |
| ENSG00000089127 | 3.17 | 2.90E-79 | OAS1 | Protein coding | 4 |
| ENSG00000123700 | 3.17 | 7.91E-25 | KCNJ2 | Protein coding | 4 |
| ENSG00000164181 | 3.15 | 3.24E-57 | ELOVL7 | Protein coding | 4 |
| ENSG00000149289 | 3.08 | 2.73E-65 | ZC3H12C | Protein coding | 4 |
| ENSG00000106701 | 3.08 | 6.45E-46 | FSD1L | Protein coding | 4 |
| ENSG00000108688 | 3.06 | 3.68E-15 | CCL7 | Protein coding | 4 |
| ENSG00000078081 | 3.06 | 4.32E-63 | LAMP3 | Protein coding | 4 |
| ENSG00000125148 | 3.01 | 1.66E-44 | MT2A | Protein coding | 4 |
| ENSG00000119922 | 3.00 | 6.85E-22 | IFIT2 | Protein coding | 4 |
| ENSG00000107104 | 2.99 | 1.01E-96 | KANK1 | Protein coding | 4 |
| ENSG00000137393 | 2.99 | 5.11E-75 | RNF144B | Protein coding | 4 |
| ENSG00000164683 | 2.96 | 2.63E-32 | HEY1 | Protein coding | 4 |
| ENSG00000136689 | 2.91 | 4.00E-15 | IL1RN | Protein coding | 4 |
| ENSG00000138642 | 2.91 | 4.98E-52 | HERC6 | Protein coding | 4 |
| ENSG00000111335 | 2.91 | 2.31E-66 | OAS2 | Protein coding | 4 |
| ENSG00000184557 | 2.90 | 2.43E-165 | SOCS3 | Protein coding | 4 |
| ENSG00000138646 | 2.89 | 2.42E-22 | HERC5 | Protein coding | 4 |
| ENSG00000102794 | 2.88 | 4.76E-12 | IRG1 | Protein coding | 4 |
| ENSG00000253831 | 2.81 | 1.08E-13 | ETV3L | Protein coding | 4 |
| ENSG00000183486 | 2.79 | 4.07E-55 | MX2 | Protein coding | 4 |
| ENSG00000121380 | 2.79 | 1.81E-11 | BCL2L14 | Protein coding | 4 |
| ENSG00000180616 | 2.79 | 6.18E-11 | SSTR2 | Protein coding | 4 |
| ENSG00000163666 | 2.79 | 1.21E-12 | HESX1 | Protein coding | 4 |
| ENSG00000162891 | 2.77 | 4.53E-13 | IL20 | Protein coding | 4 |
| ENSG00000138135 | 2.77 | 2.01E-11 | CH25H | Protein coding | 4 |
| ENSG00000136695 | 2.76 | 4.30E-10 | IL36RN | Protein coding | 4 |
| ENSG00000105711 | 2.76 | 1.55E-39 | SCN1B | Protein coding | 4 |
| ENSG00000114019 | 2.75 | 2.75E-10 | AMOTL2 | Protein coding | 4 |
| ENSG00000160932 | 2.74 | 9.03E-44 | LY6E | Protein coding | 4 |
| ENSG00000132530 | 2.72 | 3.45E-64 | XAF1 | Protein coding | 4 |
| ENSG00000196141 | 2.69 | 1.97E-35 | SPATS2L | Protein coding | 4 |
| ENSG00000144802 | 2.67 | 2.33E-125 | NFKBIZ | Protein coding | 4 |
| ENSG00000130589 | 2.66 | 8.17E-68 | HELZ2 | Protein coding | 4 |
| ENSG00000156127 | 2.64 | 2.69E-44 | BATF | Protein coding | 4 |
| ENSG00000104213 | 2.63 | 3.46E-10 | PDGFRL | Protein coding | 4 |
| ENSG00000162433 | 2.62 | 7.07E-11 | AK4 | Protein coding | 4 |
| ENSG00000124875 | 2.62 | 5.85E-14 | CXCL6 | Protein coding | 4 |
| ENSG00000150782 | 2.60 | 2.80E-42 | IL18 | Protein coding | 4 |
| ENSG00000173918 | 2.59 | 4.20E-09 | C1QTNF1 | Protein coding | 4 |
| ENSG00000125462 | 2.59 | 3.66E-30 | C1orf61 | Protein coding | 4 |
| ENSG00000117009 | 2.57 | 3.41E-36 | KMO | Protein coding | 4 |
| ENSG00000107201 | 2.56 | 7.48E-19 | DDX58 | Protein coding | 4 |
| ENSG00000115267 | 2.56 | 8.76E-44 | IFIH1 | Protein coding | 4 |
| ENSG00000124102 | 2.55 | 3.85E-37 | PI3 | Protein coding | 4 |
| ENSG00000055332 | 2.55 | 8.28E-56 | EIF2AK2 | Protein coding | 4 |
| ENSG00000013588 | 2.54 | 5.09E-24 | GPRC5A | Protein coding | 4 |
| ENSG00000135373 | 2.54 | 6.60E-17 | EHF | Protein coding | 4 |
| ENSG00000171855 | 2.53 | 3.45E-09 | IFNB1 | Protein coding | 4 |
| ENSG00000177409 | 2.52 | 4.11E-46 | SAMD9L | Protein coding | 4 |
| ENSG00000079215 | 2.51 | 3.68E-10 | SLC1A3 | Protein coding | 4 |
| ENSG00000121900 | 2.51 | 9.54E-11 | TMEM54 | Protein coding | 4 |
| ENSG00000108753 | 2.50 | 2.70E-09 | HNF1B | Protein coding | 4 |
| ENSG00000183657 | 2.50 | 1.10E-09 | PP13439 | Protein coding | 4 |
| ENSG00000151726 | 2.47 | 1.93E-47 | ACSL1 | Protein coding | 4 |
| ENSG00000152778 | 2.45 | 9.32E-34 | IFIT5 | Protein coding | 4 |
| ENSG00000172403 | 2.45 | 2.71E-17 | SYNPO2 | Protein coding | 4 |
| ENSG00000205927 | 2.41 | 1.36E-14 | OLIG2 | Protein coding | 4 |
| ENSG00000008118 | 2.39 | 7.85E-11 | CAMK1G | Protein coding | 4 |
| ENSG00000163082 | 2.38 | 4.11E-57 | SGPP2 | Protein coding | 4 |
| ENSG00000128271 | 2.38 | 3.80E-21 | ADORA2A | Protein coding | 4 |
| ENSG00000165949 | 2.37 | 1.04E-09 | IFI27 | Protein coding | 4 |
| ENSG00000140955 | 2.37 | 1.05E-07 | ADAD2 | Protein coding | 4 |
| ENSG00000187479 | 2.37 | 1.35E-11 | C11orf96 | Protein coding | 4 |
| ENSG00000172183 | 2.37 | 9.14E-42 | ISG20 | Protein coding | 4 |
| ENSG00000136048 | 2.36 | 3.60E-33 | DRAM1 | Protein coding | 4 |
| ENSG00000256515 | 2.36 | 2.43E-07 | CCL3L3 | Protein coding | 4 |
| ENSG00000133106 | 2.34 | 1.84E-37 | EPSTI1 | Protein coding | 4 |
| ENSG00000108771 | 2.34 | 2.97E-45 | DHX58 | Protein coding | 4 |
| ENSG00000068079 | 2.34 | 1.28E-30 | IFI35 | Protein coding | 4 |
| ENSG00000141837 | 2.34 | 4.49E-19 | CACNA1A | Protein coding | 4 |
| ENSG00000134716 | 2.33 | 8.38E-09 | CYP2J2 | Protein coding | 4 |
| ENSG00000198848 | 2.33 | 3.10E-30 | CES1 | Protein coding | 4 |
| ENSG00000121743 | 2.31 | 3.10E-16 | GJA3 | Protein coding | 4 |
| ENSG00000164266 | 2.30 | 2.63E-12 | SPINK1 | Protein coding | 4 |
| ENSG00000136634 | 2.29 | 2.03E-22 | IL10 | Protein coding | 4 |
| ENSG00000041982 | 2.28 | 5.04E-08 | TNC | Protein coding | 4 |
| ENSG00000136514 | 2.28 | 5.12E-11 | RTP4 | Protein coding | 4 |
| ENSG00000138821 | 2.27 | 8.87E-15 | SLC39A8 | Protein coding | 4 |
| ENSG00000112096 | 2.26 | 1.94E-45 | SOD2 | Protein coding | 4 |
| ENSG00000255398 | 2.26 | 3.27E-24 | HCAR3 | Protein coding | 4 |
| ENSG00000140379 | 2.26 | 2.16E-32 | BCL2A1 | Protein coding | 4 |
| ENSG00000196878 | 2.25 | 6.73E-59 | LAMB3 | Protein coding | 4 |
| ENSG00000196664 | 2.25 | 1.91E-40 | TLR7 | Protein coding | 4 |
| ENSG00000170312 | 2.24 | 6.28E-19 | CDK1 | Protein coding | 4 |
| ENSG00000134070 | 2.24 | 3.29E-50 | IRAK2 | Protein coding | 4 |
| ENSG00000149557 | 2.23 | 8.41E-27 | FEZ1 | Protein coding | 4 |
| ENSG00000171714 | 2.22 | 1.22E-17 | ANO5 | Protein coding | 4 |
| ENSG00000105928 | 2.21 | 1.52E-18 | DFNA5 | Protein coding | 4 |
| ENSG00000139572 | 2.21 | 3.69E-38 | GPR84 | Protein coding | 4 |
| ENSG00000204960 | 2.21 | 2.45E-07 | BLACE | Protein coding | 4 |
| ENSG00000137331 | 2.19 | 4.81E-44 | IER3 | Protein coding | 4 |
| ENSG00000180875 | 2.18 | 4.98E-12 | GREM2 | Protein coding | 4 |
| ENSG00000185507 | 2.17 | 7.64E-41 | IRF7 | Protein coding | 4 |
| ENSG00000102755 | 2.16 | 6.88E-34 | FLT1 | Protein coding | 4 |
| ENSG00000168389 | 2.16 | 1.15E-41 | MFSD2A | Protein coding | 4 |
| ENSG00000122861 | 2.16 | 1.66E-26 | PLAU | Protein coding | 4 |
| ENSG00000134470 | 2.15 | 5.61E-25 | IL15RA | Protein coding | 4 |
| ENSG00000104312 | 2.15 | 2.98E-35 | RIPK2 | Protein coding | 4 |
| ENSG00000180316 | 2.15 | 1.49E-22 | PNPLA1 | Protein coding | 4 |
| ENSG00000121858 | 2.15 | 2.37E-11 | TNFSF10 | Protein coding | 4 |
| ENSG00000157557 | 2.14 | 2.51E-50 | ETS2 | Protein coding | 4 |
| ENSG00000116690 | 2.13 | 8.94E-21 | PRG4 | Protein coding | 4 |
| ENSG00000090339 | 2.13 | 1.07E-41 | ICAM1 | Protein coding | 4 |
| ENSG00000165891 | 2.12 | 1.85E-12 | E2F7 | Protein coding | 4 |
| ENSG00000138771 | 2.12 | 7.89E-07 | SHROOM3 | Protein coding | 4 |
| ENSG00000132274 | 2.11 | 7.47E-36 | TRIM22 | Protein coding | 4 |
| ENSG00000137757 | 2.11 | 6.64E-21 | CASP5 | Protein coding | 4 |
| ENSG00000137726 | 2.11 | 1.02E-25 | FXYD6 | Protein coding | 4 |
| ENSG00000155130 | 2.10 | 3.25E-13 | MARCKS | Protein coding | 4 |
| ENSG00000143344 | 2.10 | 4.67E-11 | RGL1 | Protein coding | 4 |
| ENSG00000105825 | 2.09 | 8.31E-15 | TFPI2 | Protein coding | 4 |
| ENSG00000162614 | 2.09 | 2.37E-14 | NEXN | Protein coding | 4 |
| ENSG00000138035 | 2.09 | 7.31E-27 | PNPT1 | Protein coding | 4 |
| ENSG00000124107 | 2.07 | 2.40E-13 | SLPI | Protein coding | 4 |
| ENSG00000010030 | 2.06 | 1.10E-07 | ETV7 | Protein coding | 4 |
| ENSG00000137628 | 2.05 | 8.04E-28 | DDX60 | Protein coding | 4 |
| ENSG00000162772 | 2.05 | 3.27E-24 | ATF3 | Protein coding | 4 |
| ENSG00000182782 | 2.04 | 1.50E-11 | HCAR2 | Protein coding | 4 |
| ENSG00000110848 | 2.03 | 2.57E-27 | CD69 | Protein coding | 4 |
| ENSG00000100906 | 2.02 | 6.15E-38 | NFKBIA | Protein coding | 4 |
| ENSG00000120217 | 2.01 | 6.95E-10 | CD274 | Protein coding | 4 |
| ENSG00000114251 | 2.01 | 2.66E-18 | WNT5A | Protein coding | 4 |
| ENSG00000178860 | 2.01 | 1.42E-08 | MSC | Protein coding | 4 |
| ENSG00000138496 | 2.00 | 5.01E-27 | PARP9 | Protein coding | 4 |
| ENSG00000115956 | 2.00 | 1.58E-30 | PLEK | Protein coding | 4 |
| ENSG00000160326 | 1.99 | 7.69E-33 | SLC2A6 | Protein coding | 4 |
| ENSG00000185885 | 1.98 | 1.65E-22 | IFITM1 | Protein coding | 4 |
| ENSG00000131979 | 1.98 | 3.24E-23 | GCH1 | Protein coding | 4 |
| ENSG00000243649 | 1.98 | 2.32E-07 | CFB | Protein coding | 4 |
| ENSG00000142089 | 1.96 | 4.88E-09 | IFITM3 | Protein coding | 4 |
| ENSG00000121797 | 1.96 | 6.93E-55 | CCRL2 | Protein coding | 4 |
| ENSG00000166920 | 1.95 | 7.77E-13 | C15orf48 | Protein coding | 4 |
| ENSG00000026751 | 1.95 | 3.98E-12 | SLAMF7 | Protein coding | 4 |
| ENSG00000038210 | 1.95 | 1.59E-31 | PI4K2B | Protein coding | 4 |
| ENSG00000135114 | 1.93 | 2.13E-23 | OASL | Protein coding | 4 |
| ENSG00000196569 | 1.93 | 4.65E-08 | LAMA2 | Protein coding | 4 |
| ENSG00000166016 | 1.92 | 8.41E-28 | ABTB2 | Protein coding | 4 |
| ENSG00000104432 | 1.92 | 5.28E-14 | IL7 | Protein coding | 4 |
| ENSG00000116833 | 1.92 | 8.71E-06 | NR5A2 | Protein coding | 4 |
| ENSG00000102096 | 1.90 | 3.77E-44 | PIM2 | Protein coding | 4 |
| ENSG00000205413 | 1.90 | 3.62E-27 | SAMD9 | Protein coding | 4 |
| ENSG00000075651 | 1.90 | 2.30E-32 | PLD1 | Protein coding | 4 |
| ENSG00000140464 | 1.90 | 1.47E-34 | PML | Protein coding | 4 |
| ENSG00000148926 | 1.89 | 1.28E-39 | ADM | Protein coding | 4 |
| ENSG00000185338 | 1.88 | 4.82E-27 | SOCS1 | Protein coding | 4 |
| ENSG00000163840 | 1.88 | 2.05E-27 | DTX3L | Protein coding | 4 |
| ENSG00000116663 | 1.88 | 1.13E-19 | FBXO6 | Protein coding | 4 |
| ENSG00000179431 | 1.87 | 7.80E-07 | FJX1 | Protein coding | 4 |
| ENSG00000117594 | 1.87 | 8.77E-05 | HSD11B1 | Protein coding | 4 |
| ENSG00000135899 | 1.86 | 9.27E-33 | SP110 | Protein coding | 4 |
| ENSG00000166394 | 1.86 | 1.05E-12 | CYB5R2 | Protein coding | 4 |
| ENSG00000168961 | 1.86 | 3.85E-37 | LGALS9 | Protein coding | 4 |
| ENSG00000104320 | 1.86 | 2.34E-34 | NBN | Protein coding | 4 |
| ENSG00000168062 | 1.85 | 1.12E-05 | BATF2 | Protein coding | 4 |
| ENSG00000164825 | 1.85 | 2.67E-08 | DEFB1 | Protein coding | 4 |
| ENSG00000104951 | 1.85 | 2.28E-25 | IL4I1 | Protein coding | 4 |
| ENSG00000178965 | 1.85 | 6.39E-05 | C1orf173 | Protein coding | 4 |
| ENSG00000166523 | 1.85 | 4.52E-23 | CLEC4E | Protein coding | 4 |
| ENSG00000122643 | 1.84 | 3.72E-20 | NT5C3 | Protein coding | 4 |
| ENSG00000089692 | 1.83 | 1.09E-22 | LAG3 | Protein coding | 4 |
| ENSG00000196116 | 1.82 | 2.08E-22 | TDRD7 | Protein coding | 4 |
| ENSG00000152503 | 1.82 | 1.06E-42 | TRIM36 | Protein coding | 4 |
| ENSG00000213689 | 1.82 | 2.26E-33 | TREX1 | Protein coding | 4 |
| ENSG00000059378 | 1.82 | 1.53E-28 | PARP12 | Protein coding | 4 |
| ENSG00000169194 | 1.80 | 3.90E-06 | IL13 | Protein coding | 4 |
| ENSG00000142224 | 1.80 | 2.55E-09 | IL19 | Protein coding | 4 |
| ENSG00000153132 | 1.79 | 1.52E-08 | CLGN | Protein coding | 4 |
| ENSG00000197982 | 1.79 | 5.59E-21 | C1orf122 | Protein coding | 4 |
| ENSG00000164761 | 1.79 | 0.000203301 | TNFRSF11B | Protein coding | 4 |
| ENSG00000124256 | 1.78 | 1.27E-22 | ZBP1 | Protein coding | 4 |
| ENSG00000049130 | 1.78 | 3.69E-06 | KITLG | Protein coding | 4 |
| ENSG00000140961 | 1.78 | 7.92E-12 | OSGIN1 | Protein coding | 4 |
| ENSG00000011422 | 1.77 | 1.99E-31 | PLAUR | Protein coding | 4 |
| ENSG00000138166 | 1.76 | 3.30E-24 | DUSP5 | Protein coding | 4 |
| ENSG00000140450 | 1.76 | 2.34E-19 | ARRDC4 | Protein coding | 4 |
| ENSG00000183473 | 1.76 | 3.58E-16 | SSTR3 | Protein coding | 4 |
| ENSG00000128383 | 1.75 | 1.13E-07 | APOBEC3A | Protein coding | 4 |
| ENSG00000214872 | 1.75 | 3.87E-15 | SMTNL1 | Protein coding | 4 |
| ENSG00000182168 | 1.75 | 0.000290152 | UNC5C | Protein coding | 4 |
| ENSG00000169504 | 1.75 | 5.05E-30 | CLIC4 | Protein coding | 4 |
| ENSG00000188313 | 1.74 | 7.93E-30 | PLSCR1 | Protein coding | 4 |
| ENSG00000185022 | 1.74 | 4.76E-29 | MAFF | Protein coding | 4 |
| ENSG00000166670 | 1.74 | 2.40E-05 | MMP10 | Protein coding | 4 |
| ENSG00000056558 | 1.73 | 6.74E-58 | TRAF1 | Protein coding | 4 |
| ENSG00000163565 | 1.72 | 8.34E-24 | IFI16 | Protein coding | 4 |
| ENSG00000087074 | 1.72 | 7.32E-40 | PPP1R15A | Protein coding | 4 |
| ENSG00000183742 | 1.72 | 1.72E-15 | MACC1 | Protein coding | 4 |
| ENSG00000105246 | 1.71 | 4.87E-17 | EBI3 | Protein coding | 4 |
| ENSG00000149798 | 1.71 | 1.05E-23 | CDC42EP2 | Protein coding | 4 |
| ENSG00000136810 | 1.71 | 1.08E-17 | TXN | Protein coding | 4 |
| ENSG00000186583 | 1.70 | 2.49E-09 | SPATC1 | Protein coding | 4 |
| ENSG00000215306 | 1.70 | 0.000207753 | AL135998.1 | Protein coding | 4 |
| ENSG00000221949 | 1.70 | 2.01E-07 | C12orf61 | Protein coding | 4 |
| ENSG00000125384 | 1.70 | 3.21E-16 | PTGER2 | Protein coding | 4 |
| ENSG00000130303 | 1.70 | 3.55E-23 | BST2 | Protein coding | 4 |
| ENSG00000134460 | 1.69 | 3.73E-20 | IL2RA | Protein coding | 4 |
| ENSG00000185215 | 1.67 | 6.66E-22 | TNFAIP2 | Protein coding | 4 |
| ENSG00000075618 | 1.67 | 4.01E-23 | FSCN1 | Protein coding | 4 |
| ENSG00000123685 | 1.66 | 3.51E-07 | BATF3 | Protein coding | 4 |
| ENSG00000100024 | 1.65 | 1.15E-11 | UPB1 | Protein coding | 4 |
| ENSG00000103569 | 1.65 | 2.01E-36 | AQP9 | Protein coding | 4 |
| ENSG00000118194 | 1.65 | 0.000250971 | TNNT2 | Protein coding | 4 |
| ENSG00000154262 | 1.64 | 1.44E-07 | ABCA6 | Protein coding | 4 |
| ENSG00000173786 | 1.64 | 1.10E-26 | CNP | Protein coding | 4 |
| ENSG00000196684 | 1.64 | 1.10E-20 | HSH2D | Protein coding | 4 |
| ENSG00000217825 | 1.64 | 6.04E-05 | AC099552.4 | Protein coding | 4 |
| ENSG00000143061 | 1.64 | 1.39E-07 | IGSF3 | Protein coding | 4 |
| ENSG00000101438 | 1.64 | 0.000803253 | SLC32A1 | Protein coding | 4 |
| ENSG00000132256 | 1.63 | 4.82E-27 | TRIM5 | Protein coding | 4 |
| ENSG00000138670 | 1.62 | 5.94E-24 | RASGEF1B | Protein coding | 4 |
| ENSG00000029559 | 1.62 | 0.000892394 | IBSP | Protein coding | 4 |
| ENSG00000181649 | 1.61 | 4.80E-07 | PHLDA2 | Protein coding | 4 |
| ENSG00000147434 | 1.60 | 1.00E-05 | CHRNA6 | Protein coding | 4 |
| ENSG00000170525 | 1.60 | 4.70E-35 | PFKFB3 | Protein coding | 4 |
| ENSG00000112773 | 1.59 | 1.35E-20 | FAM46A | Protein coding | 4 |
| ENSG00000137462 | 1.59 | 5.75E-26 | TLR2 | Protein coding | 4 |
| ENSG00000198673 | 1.59 | 7.92E-14 | FAM19A2 | Protein coding | 4 |
| ENSG00000156587 | 1.59 | 1.21E-15 | UBE2L6 | Protein coding | 4 |
| ENSG00000180730 | 1.59 | 4.73E-06 | SHISA2 | Protein coding | 4 |
| ENSG00000130222 | 1.58 | 2.67E-07 | GADD45G | Protein coding | 4 |
| ENSG00000152229 | 1.58 | 1.67E-07 | PSTPIP2 | Protein coding | 4 |
| ENSG00000171631 | 1.58 | 4.10E-23 | P2RY6 | Protein coding | 4 |
| ENSG00000196639 | 1.58 | 5.85E-16 | HRH1 | Protein coding | 4 |
| ENSG00000132109 | 1.58 | 1.75E-15 | TRIM21 | Protein coding | 4 |
| ENSG00000198121 | 1.56 | 6.69E-08 | LPAR1 | Protein coding | 4 |
| ENSG00000235531 | 1.56 | 9.37E-08 | RP11-383H13.1 | Protein coding | 4 |
| ENSG00000169245 | 1.56 | 0.001337552 | CXCL10 | Protein coding | 4 |
| ENSG00000121594 | 1.55 | 3.10E-10 | CD80 | Protein coding | 4 |
| ENSG00000124882 | 1.55 | 9.77E-24 | EREG | Protein coding | 4 |
| ENSG00000148737 | 1.55 | 5.90E-25 | TCF7L2 | Protein coding | 4 |
| ENSG00000125733 | 1.54 | 7.36E-31 | TRIP10 | Protein coding | 4 |
| ENSG00000102554 | 1.54 | 4.25E-16 | KLF5 | Protein coding | 4 |
| ENSG00000125730 | 1.54 | 3.07E-17 | C3 | Protein coding | 4 |
| ENSG00000135678 | 1.54 | 1.09E-12 | CPM | Protein coding | 4 |
| ENSG00000026103 | 1.54 | 1.28E-19 | FAS | Protein coding | 4 |
| ENSG00000064787 | 1.54 | 1.03E-06 | BCAS1 | Protein coding | 4 |
| ENSG00000204099 | 1.53 | 5.39E-16 | NEU4 | Protein coding | 4 |
| ENSG00000162493 | 1.53 | 1.66E-07 | PDPN | Protein coding | 4 |
| ENSG00000130164 | 1.53 | 2.25E-31 | LDLR | Protein coding | 4 |
| ENSG00000163644 | 1.53 | 3.36E-23 | PPM1K | Protein coding | 4 |
| ENSG00000175471 | 1.53 | 6.86E-18 | MCTP1 | Protein coding | 4 |
| ENSG00000168334 | 1.53 | 0.001468582 | XIRP1 | Protein coding | 4 |
| ENSG00000167191 | 1.52 | 0.002168268 | GPRC5B | Protein coding | 4 |
| ENSG00000173801 | 1.52 | 3.42E-19 | JUP | Protein coding | 4 |
| ENSG00000182175 | 1.52 | 2.57E-06 | RGMA | Protein coding | 4 |
| ENSG00000101331 | 1.52 | 0.000945222 | CCM2L | Protein coding | 4 |
| ENSG00000188211 | 1.52 | 1.04E-11 | NCR3LG1 | Protein coding | 4 |
| ENSG00000158470 | 1.52 | 5.45E-30 | B4GALT5 | Protein coding | 4 |
| ENSG00000074416 | 1.51 | 4.83E-22 | MGLL | Protein coding | 4 |
| ENSG00000131669 | 1.51 | 2.90E-30 | NINJ1 | Protein coding | 4 |
| ENSG00000110047 | 1.50 | 9.54E-49 | EHD1 | Protein coding | 4 |
| ENSG00000102794 | 8.92 | 0 | IRG1 | Protein coding | 24 |
| ENSG00000196932 | 7.81 | 1.07E-63 | TMEM26 | Protein coding | 24 |
| ENSG00000108691 | 7.76 | 3.21E-102 | CCL2 | Protein coding | 24 |
| ENSG00000163739 | 7.70 | 0 | CXCL1 | Protein coding | 24 |
| ENSG00000108688 | 7.52 | 4.40E-49 | CCL7 | Protein coding | 24 |
| ENSG00000163735 | 7.50 | 1.59E-119 | CXCL5 | Protein coding | 24 |
| ENSG00000165474 | 7.36 | 2.86E-69 | GJB2 | Protein coding | 24 |
| ENSG00000172379 | 6.86 | 1.38E-57 | ARNT2 | Protein coding | 24 |
| ENSG00000108700 | 6.86 | 2.04E-30 | CCL8 | Protein coding | 24 |
| ENSG00000108342 | 6.74 | 1.82E-47 | CSF3 | Protein coding | 24 |
| ENSG00000172724 | 6.68 | 8.32E-34 | CCL19 | Protein coding | 24 |
| ENSG00000125538 | 6.67 | 4.90E-245 | IL1B | Protein coding | 24 |
| ENSG00000166396 | 6.61 | 6.77E-38 | SERPINB7 | Protein coding | 24 |
| ENSG00000124102 | 6.55 | 2.60E-72 | PI3 | Protein coding | 24 |
| ENSG00000163734 | 6.49 | 2.07E-273 | CXCL3 | Protein coding | 24 |
| ENSG00000172551 | 6.44 | 2.21E-44 | MUCL1 | Protein coding | 24 |
| ENSG00000136244 | 6.40 | 2.40E-60 | IL6 | Protein coding | 24 |
| ENSG00000173918 | 6.37 | 1.07E-35 | C1QTNF1 | Protein coding | 24 |
| ENSG00000115414 | -6.36 | 1.57E-77 | FN1 | Protein coding | 24 |
| ENSG00000136688 | 6.31 | 5.92E-47 | IL36G | Protein coding | 24 |
| ENSG00000169429 | 6.13 | 0 | IL8 | Protein coding | 24 |
| ENSG00000006074 | 6.11 | 4.43E-29 | CCL18 | Protein coding | 24 |
| ENSG00000128917 | 6.02 | 2.23E-32 | DLL4 | Protein coding | 24 |
| ENSG00000121743 | 6.00 | 3.20E-87 | GJA3 | Protein coding | 24 |
| ENSG00000197632 | 5.93 | 9.64E-225 | SERPINB2 | Protein coding | 24 |
| ENSG00000170866 | 5.85 | 2.25E-51 | LILRA3 | Protein coding | 24 |
| ENSG00000074410 | 5.83 | 3.41E-39 | CA12 | Protein coding | 24 |
| ENSG00000081041 | 5.82 | 4.56E-234 | CXCL2 | Protein coding | 24 |
| ENSG00000157227 | 5.81 | 1.91E-243 | MMP14 | Protein coding | 24 |
| ENSG00000129538 | -5.76 | 1.90E-36 | RNASE1 | Protein coding | 24 |
| ENSG00000103888 | 5.74 | 1.39E-65 | KIAA1199 | Protein coding | 24 |
| ENSG00000162493 | 5.73 | 8.09E-29 | PDPN | Protein coding | 24 |
| ENSG00000105855 | 5.73 | 1.29E-55 | ITGB8 | Protein coding | 24 |
| ENSG00000148344 | 5.68 | 6.61E-27 | PTGES | Protein coding | 24 |
| ENSG00000099998 | 5.68 | 2.26E-30 | GGT5 | Protein coding | 24 |
| ENSG00000253831 | 5.61 | 9.55E-34 | ETV3L | Protein coding | 24 |
| ENSG00000183762 | 5.59 | 3.27E-55 | KREMEN1 | Protein coding | 24 |
| ENSG00000006075 | 5.56 | 7.64E-197 | CCL3 | Protein coding | 24 |
| ENSG00000124875 | 5.50 | 2.60E-46 | CXCL6 | Protein coding | 24 |
| ENSG00000123610 | 5.38 | 1.58E-74 | TNFAIP6 | Protein coding | 24 |
| ENSG00000150510 | 5.38 | 1.36E-38 | FAM124A | Protein coding | 24 |
| ENSG00000174705 | 5.33 | 3.37E-32 | SH3PXD2B | Protein coding | 24 |
| ENSG00000170323 | -5.32 | 4.53E-26 | FABP4 | Protein coding | 24 |
| ENSG00000105825 | 5.27 | 3.61E-118 | TFPI2 | Protein coding | 24 |
| ENSG00000123689 | 5.26 | 2.21E-47 | G0S2 | Protein coding | 24 |
| ENSG00000135047 | 5.21 | 6.92E-81 | CTSL1 | Protein coding | 24 |
| ENSG00000164181 | 5.21 | 9.38E-80 | ELOVL7 | Protein coding | 24 |
| ENSG00000101331 | 5.21 | 1.22E-36 | CCM2L | Protein coding | 24 |
| ENSG00000154065 | 5.21 | 2.15E-26 | ANKRD29 | Protein coding | 24 |
| ENSG00000111537 | 5.18 | 6.10E-25 | IFNG | Protein coding | 24 |
| ENSG00000163395 | 5.14 | 1.75E-20 | IGFN1 | Protein coding | 24 |
| ENSG00000110436 | 5.11 | 7.38E-143 | SLC1A2 | Protein coding | 24 |
| ENSG00000205927 | 5.10 | 9.90E-35 | OLIG2 | Protein coding | 24 |
| ENSG00000114251 | 5.10 | 3.44E-24 | WNT5A | Protein coding | 24 |
| ENSG00000053747 | 5.06 | 2.20E-22 | LAMA3 | Protein coding | 24 |
| ENSG00000105509 | 5.05 | 2.55E-26 | HAS1 | Protein coding | 24 |
| ENSG00000073756 | 5.03 | 1.17E-134 | PTGS2 | Protein coding | 24 |
| ENSG00000105976 | 4.97 | 8.50E-38 | MET | Protein coding | 24 |
| ENSG00000050730 | 4.95 | 2.76E-194 | TNIP3 | Protein coding | 24 |
| ENSG00000164400 | 4.93 | 1.25E-22 | CSF2 | Protein coding | 24 |
| ENSG00000122641 | 4.93 | 3.41E-41 | INHBA | Protein coding | 24 |
| ENSG00000152766 | 4.92 | 1.13E-21 | ANKRD22 | Protein coding | 24 |
| ENSG00000131203 | 4.89 | 7.16E-64 | IDO1 | Protein coding | 24 |
| ENSG00000136634 | 4.87 | 2.31E-88 | IL10 | Protein coding | 24 |
| ENSG00000162433 | 4.87 | 1.09E-63 | AK4 | Protein coding | 24 |
| ENSG00000019169 | 4.86 | 5.71E-41 | MARCO | Protein coding | 24 |
| ENSG00000156234 | 4.80 | 3.88E-31 | CXCL13 | Protein coding | 24 |
| ENSG00000196611 | 4.79 | 9.97E-23 | MMP1 | Protein coding | 24 |
| ENSG00000162407 | 4.72 | 1.77E-104 | PPAP2B | Protein coding | 24 |
| ENSG00000115008 | 4.70 | 6.63E-31 | IL1A | Protein coding | 24 |
| ENSG00000126262 | 4.70 | 1.01E-30 | FFAR2 | Protein coding | 24 |
| ENSG00000139572 | 4.70 | 5.68E-118 | GPR84 | Protein coding | 24 |
| ENSG00000008118 | 4.69 | 1.69E-15 | CAMK1G | Protein coding | 24 |
| ENSG00000125730 | 4.69 | 4.24E-47 | C3 | Protein coding | 24 |
| ENSG00000138685 | 4.62 | 5.42E-19 | FGF2 | Protein coding | 24 |
| ENSG00000243649 | 4.60 | 9.33E-20 | CFB | Protein coding | 24 |
| ENSG00000142224 | 4.58 | 1.12E-25 | IL19 | Protein coding | 24 |
| ENSG00000121380 | 4.57 | 9.45E-21 | BCL2L14 | Protein coding | 24 |
| ENSG00000180616 | 4.55 | 2.78E-18 | SSTR2 | Protein coding | 24 |
| ENSG00000236939 | 4.47 | 1.45E-16 | C8orf56 | Protein coding | 24 |
| ENSG00000166920 | 4.43 | 1.55E-43 | C15orf48 | Protein coding | 24 |
| ENSG00000171049 | 4.42 | 1.47E-148 | FPR2 | Protein coding | 24 |
| ENSG00000164509 | 4.42 | 5.79E-24 | IL31RA | Protein coding | 24 |
| ENSG00000213694 | 4.39 | 3.72E-31 | S1PR3 | Protein coding | 24 |
| ENSG00000137757 | 4.38 | 1.60E-44 | CASP5 | Protein coding | 24 |
| ENSG00000183019 | 4.33 | 5.15E-75 | C19orf59 | Protein coding | 24 |
| ENSG00000138135 | 4.33 | 7.79E-22 | CH25H | Protein coding | 24 |
| ENSG00000018280 | 4.32 | 2.28E-112 | SLC11A1 | Protein coding | 24 |
| ENSG00000136695 | 4.31 | 7.88E-15 | IL36RN | Protein coding | 24 |
| ENSG00000154188 | -4.25 | 3.30E-27 | ANGPT1 | Protein coding | 24 |
| ENSG00000124731 | 4.24 | 4.70E-71 | TREM1 | Protein coding | 24 |
| ENSG00000138316 | 4.24 | 1.92E-54 | ADAMTS14 | Protein coding | 24 |
| ENSG00000120217 | 4.20 | 4.54E-134 | CD274 | Protein coding | 24 |
| ENSG00000135111 | 4.18 | 7.62E-21 | TBX3 | Protein coding | 24 |
| ENSG00000117594 | 4.18 | 1.16E-14 | HSD11B1 | Protein coding | 24 |
| ENSG00000137673 | 4.18 | 2.91E-36 | MMP7 | Protein coding | 24 |
| ENSG00000107249 | 4.18 | 2.68E-81 | GLIS3 | Protein coding | 24 |
| ENSG00000115009 | 4.14 | 8.41E-28 | CCL20 | Protein coding | 24 |
| ENSG00000105835 | 4.11 | 2.40E-172 | NAMPT | Protein coding | 24 |
| ENSG00000184557 | 4.11 | 1.25E-147 | SOCS3 | Protein coding | 24 |
| ENSG00000229644 | 4.08 | 8.43E-124 | NAMPTL | Protein coding | 24 |
| ENSG00000173239 | 4.08 | 7.45E-12 | LIPM | Protein coding | 24 |
| ENSG00000136379 | 4.06 | 3.23E-93 | FAM108C1 | Protein coding | 24 |
| ENSG00000196878 | 4.06 | 3.77E-60 | LAMB3 | Protein coding | 24 |
| ENSG00000244405 | 4.04 | 3.42E-29 | ETV5 | Protein coding | 24 |
| ENSG00000198019 | 4.03 | 2.55E-31 | FCGR1B | Protein coding | 24 |
| ENSG00000111012 | 4.02 | 6.73E-39 | CYP27B1 | Protein coding | 24 |
| ENSG00000099250 | 4.01 | 2.31E-115 | NRP1 | Protein coding | 24 |
| ENSG00000162494 | 4.01 | 1.10E-09 | LRRC38 | Protein coding | 24 |
| ENSG00000113302 | 4.00 | 1.57E-14 | IL12B | Protein coding | 24 |
| ENSG00000187957 | 3.98 | 1.27E-20 | DNER | Protein coding | 24 |
| ENSG00000100191 | 3.98 | 2.71E-11 | SLC5A4 | Protein coding | 24 |
| ENSG00000172594 | 3.97 | 1.53E-152 | SMPDL3A | Protein coding | 24 |
| ENSG00000134247 | -3.96 | 4.06E-23 | PTGFRN | Protein coding | 24 |
| ENSG00000167236 | 3.95 | 1.20E-25 | CCL23 | Protein coding | 24 |
| ENSG00000121797 | 3.92 | 1.26E-94 | CCRL2 | Protein coding | 24 |
| ENSG00000197646 | 3.92 | 2.75E-24 | PDCD1LG2 | Protein coding | 24 |
| ENSG00000164692 | 3.92 | 1.90E-21 | COL1A2 | Protein coding | 24 |
| ENSG00000165029 | 3.91 | 9.29E-126 | ABCA1 | Protein coding | 24 |
| ENSG00000112096 | 3.90 | 1.78E-208 | SOD2 | Protein coding | 24 |
| ENSG00000133048 | 3.89 | 7.12E-27 | CHI3L1 | Protein coding | 24 |
| ENSG00000136048 | 3.89 | 8.49E-98 | DRAM1 | Protein coding | 24 |
| ENSG00000205021 | 3.88 | 3.46E-24 | CCL3L1 | Protein coding | 24 |
| ENSG00000196639 | 3.88 | 2.17E-52 | HRH1 | Protein coding | 24 |
| ENSG00000186818 | 3.87 | 3.82E-20 | LILRB4 | Protein coding | 24 |
| ENSG00000075651 | 3.84 | 1.10E-115 | PLD1 | Protein coding | 24 |
| ENSG00000125780 | 3.83 | 5.06E-11 | TGM3 | Protein coding | 24 |
| ENSG00000138821 | 3.82 | 3.86E-128 | SLC39A8 | Protein coding | 24 |
| ENSG00000105707 | 3.81 | 1.97E-17 | HPN | Protein coding | 24 |
| ENSG00000108702 | 3.81 | 1.44E-08 | CCL1 | Protein coding | 24 |
| ENSG00000168062 | 3.79 | 9.58E-15 | BATF2 | Protein coding | 24 |
| ENSG00000145244 | -3.79 | 4.85E-68 | CORIN | Protein coding | 24 |
| ENSG00000170525 | 3.79 | 6.75E-145 | PFKFB3 | Protein coding | 24 |
| ENSG00000157168 | -3.78 | 2.56E-17 | NRG1 | Protein coding | 24 |
| ENSG00000139567 | 3.78 | 2.50E-17 | ACVRL1 | Protein coding | 24 |
| ENSG00000148926 | 3.78 | 2.30E-25 | ADM | Protein coding | 24 |
| ENSG00000138755 | 3.78 | 2.00E-09 | CXCL9 | Protein coding | 24 |
| ENSG00000169715 | 3.76 | 2.47E-13 | MT1E | Protein coding | 24 |
| ENSG00000100985 | 3.74 | 5.23E-18 | MMP9 | Protein coding | 24 |
| ENSG00000099985 | 3.74 | 2.55E-63 | OSM | Protein coding | 24 |
| ENSG00000103569 | 3.73 | 3.20E-280 | AQP9 | Protein coding | 24 |
| ENSG00000117228 | 3.72 | 9.18E-30 | GBP1 | Protein coding | 24 |
| ENSG00000067798 | 3.71 | 2.56E-10 | NAV3 | Protein coding | 24 |
| ENSG00000169413 | -3.71 | 8.16E-23 | RNASE6 | Protein coding | 24 |
| ENSG00000164821 | -3.70 | 1.01E-10 | DEFA4 | Protein coding | 24 |
| ENSG00000253958 | 3.69 | 7.51E-16 | CLDN23 | Protein coding | 24 |
| ENSG00000150337 | 3.68 | 6.88E-11 | FCGR1A | Protein coding | 24 |
| ENSG00000163736 | 3.68 | 4.22E-83 | PPBP | Protein coding | 24 |
| ENSG00000169946 | 3.67 | 1.02E-11 | ZFPM2 | Protein coding | 24 |
| ENSG00000165124 | 3.66 | 5.12E-12 | SVEP1 | Protein coding | 24 |
| ENSG00000106034 | -3.65 | 3.20E-42 | CPED1 | Protein coding | 24 |
| ENSG00000133687 | 3.65 | 7.00E-19 | TMTC1 | Protein coding | 24 |
| ENSG00000128578 | 3.65 | 3.29E-49 | STRIP2 | Protein coding | 24 |
| ENSG00000010030 | 3.64 | 1.15E-13 | ETV7 | Protein coding | 24 |
| ENSG00000173369 | 3.63 | 3.79E-14 | C1QB | Protein coding | 24 |
| ENSG00000134765 | -3.63 | 3.32E-29 | DSC1 | Protein coding | 24 |
| ENSG00000105711 | 3.61 | 3.29E-48 | SCN1B | Protein coding | 24 |
| ENSG00000122861 | 3.60 | 4.71E-29 | PLAU | Protein coding | 24 |
| ENSG00000151726 | 3.60 | 2.57E-219 | ACSL1 | Protein coding | 24 |
| ENSG00000090659 | 3.60 | 9.77E-29 | CD209 | Protein coding | 24 |
| ENSG00000197272 | 3.60 | 3.54E-10 | IL27 | Protein coding | 24 |
| ENSG00000198959 | 3.58 | 4.80E-78 | TGM2 | Protein coding | 24 |
| ENSG00000140022 | 3.58 | 6.45E-17 | STON2 | Protein coding | 24 |
| ENSG00000180316 | 3.56 | 5.32E-22 | PNPLA1 | Protein coding | 24 |
| ENSG00000153208 | 3.56 | 5.94E-20 | MERTK | Protein coding | 24 |
| ENSG00000183657 | 3.56 | 2.41E-09 | PP13439 | Protein coding | 24 |
| ENSG00000134028 | 3.55 | 1.40E-18 | ADAMDEC1 | Protein coding | 24 |
| ENSG00000198848 | 3.55 | 3.37E-15 | CES1 | Protein coding | 24 |
| ENSG00000085265 | -3.54 | 1.19E-28 | FCN1 | Protein coding | 24 |
| ENSG00000164929 | 3.54 | 2.42E-30 | BAALC | Protein coding | 24 |
| ENSG00000164266 | 3.54 | 8.39E-17 | SPINK1 | Protein coding | 24 |
| ENSG00000182580 | -3.54 | 7.82E-10 | EPHB3 | Protein coding | 24 |
| ENSG00000138944 | 3.53 | 2.80E-10 | KIAA1644 | Protein coding | 24 |
| ENSG00000149289 | 3.53 | 3.80E-107 | ZC3H12C | Protein coding | 24 |
| ENSG00000163518 | 3.53 | 8.66E-21 | FCRL4 | Protein coding | 24 |
| ENSG00000125144 | 3.52 | 7.82E-08 | MT1G | Protein coding | 24 |
| ENSG00000163661 | 3.52 | 3.13E-46 | PTX3 | Protein coding | 24 |
| ENSG00000064042 | 3.52 | 1.49E-16 | LIMCH1 | Protein coding | 24 |
| ENSG00000079215 | 3.51 | 1.05E-13 | SLC1A3 | Protein coding | 24 |
| ENSG00000189221 | 3.51 | 4.27E-13 | MAOA | Protein coding | 24 |
| ENSG00000125148 | 3.50 | 1.93E-21 | MT2A | Protein coding | 24 |
| ENSG00000121594 | 3.50 | 2.13E-43 | CD80 | Protein coding | 24 |
| ENSG00000087076 | 3.49 | 5.59E-20 | HSD17B14 | Protein coding | 24 |
| ENSG00000164047 | -3.48 | 2.91E-10 | CAMP | Protein coding | 24 |
| ENSG00000084734 | 3.48 | 3.23E-08 | GCKR | Protein coding | 24 |
| ENSG00000011422 | 3.47 | 3.53E-65 | PLAUR | Protein coding | 24 |
| ENSG00000123700 | 3.47 | 2.62E-76 | KCNJ2 | Protein coding | 24 |
| ENSG00000086300 | 3.46 | 9.51E-138 | SNX10 | Protein coding | 24 |
| ENSG00000006118 | 3.46 | 2.47E-53 | TMEM132A | Protein coding | 24 |
| ENSG00000145685 | 3.45 | 8.19E-69 | LHFPL2 | Protein coding | 24 |
| ENSG00000105928 | 3.44 | 6.31E-20 | DFNA5 | Protein coding | 24 |
| ENSG00000134531 | 3.42 | 8.81E-21 | EMP1 | Protein coding | 24 |
| ENSG00000188820 | 3.40 | 2.88E-12 | FAM26F | Protein coding | 24 |
| ENSG00000173372 | 3.36 | 3.63E-14 | C1QA | Protein coding | 24 |
| ENSG00000100285 | 3.36 | 1.25E-45 | NEFH | Protein coding | 24 |
| ENSG00000112115 | 3.35 | 1.74E-06 | IL17A | Protein coding | 24 |
| ENSG00000205856 | 3.35 | 1.68E-06 | C22orf42 | Protein coding | 24 |
| ENSG00000181634 | 3.34 | 9.00E-34 | TNFSF15 | Protein coding | 24 |
| ENSG00000161944 | -3.34 | 8.73E-30 | ASGR2 | Protein coding | 24 |
| ENSG00000174837 | 3.33 | 6.72E-62 | EMR1 | Protein coding | 24 |
| ENSG00000047936 | 3.33 | 2.09E-06 | ROS1 | Protein coding | 24 |
| ENSG00000155659 | -3.33 | 6.29E-30 | VSIG4 | Protein coding | 24 |
| ENSG00000100767 | 3.30 | 1.72E-58 | PAPLN | Protein coding | 24 |
| ENSG00000135549 | -3.30 | 4.24E-46 | PKIB | Protein coding | 24 |
| ENSG00000100336 | 3.30 | 4.41E-09 | APOL4 | Protein coding | 24 |
| ENSG00000026751 | 3.30 | 8.28E-62 | SLAMF7 | Protein coding | 24 |
| ENSG00000130558 | -3.29 | 7.49E-20 | OLFM1 | Protein coding | 24 |
| ENSG00000149798 | 3.29 | 7.15E-58 | CDC42EP2 | Protein coding | 24 |
| ENSG00000125810 | 3.29 | 9.66E-59 | CD93 | Protein coding | 24 |
| ENSG00000172927 | 3.29 | 1.58E-11 | MYEOV | Protein coding | 24 |
| ENSG00000172817 | 3.29 | 7.85E-17 | CYP7B1 | Protein coding | 24 |
| ENSG00000116574 | 3.28 | 2.14E-110 | RHOU | Protein coding | 24 |
| ENSG00000127318 | 3.27 | 2.13E-08 | IL22 | Protein coding | 24 |
| ENSG00000183748 | 3.27 | 2.17E-16 | MRC1L1 | Protein coding | 24 |
| ENSG00000169908 | 3.26 | 5.95E-09 | TM4SF1 | Protein coding | 24 |
| ENSG00000178789 | -3.25 | 5.89E-71 | CD300LB | Protein coding | 24 |
| ENSG00000181374 | 3.24 | 1.95E-06 | CCL13 | Protein coding | 24 |
| ENSG00000159450 | 3.24 | 1.24E-09 | TCHH | Protein coding | 24 |
| ENSG00000204099 | 3.24 | 3.76E-13 | NEU4 | Protein coding | 24 |
| ENSG00000132514 | -3.23 | 3.13E-10 | CLEC10A | Protein coding | 24 |
| ENSG00000129277 | 3.23 | 3.95E-64 | CCL4 | Protein coding | 24 |
| ENSG00000128512 | 3.22 | 1.13E-121 | DOCK4 | Protein coding | 24 |
| ENSG00000172752 | 3.22 | 2.01E-06 | COL6A5 | Protein coding | 24 |
| ENSG00000124479 | 3.22 | 2.12E-06 | NDP | Protein coding | 24 |
| ENSG00000112818 | 3.21 | 5.28E-16 | MEP1A | Protein coding | 24 |
| ENSG00000111199 | 3.21 | 2.65E-08 | TRPV4 | Protein coding | 24 |
| ENSG00000134460 | 3.20 | 3.53E-65 | IL2RA | Protein coding | 24 |
| ENSG00000074416 | 3.19 | 7.43E-139 | MGLL | Protein coding | 24 |
| ENSG00000101000 | 3.18 | 1.35E-16 | PROCR | Protein coding | 24 |
| ENSG00000143344 | 3.18 | 8.58E-15 | RGL1 | Protein coding | 24 |
| ENSG00000106701 | 3.18 | 9.82E-139 | FSD1L | Protein coding | 24 |
| ENSG00000096006 | -3.18 | 1.31E-11 | CRISP3 | Protein coding | 24 |
| ENSG00000159189 | 3.17 | 9.07E-12 | C1QC | Protein coding | 24 |
| ENSG00000198121 | 3.17 | 3.22E-37 | LPAR1 | Protein coding | 24 |
| ENSG00000074660 | 3.17 | 2.67E-65 | SCARF1 | Protein coding | 24 |
| ENSG00000136052 | 3.16 | 1.11E-100 | SLC41A2 | Protein coding | 24 |
| ENSG00000165238 | 3.16 | 1.74E-14 | WNK2 | Protein coding | 24 |
| ENSG00000244482 | 3.15 | 2.60E-25 | LILRA6 | Protein coding | 24 |
| ENSG00000198178 | -3.14 | 2.50E-07 | CLEC4C | Protein coding | 24 |
| ENSG00000163823 | 3.14 | 3.93E-20 | CCR1 | Protein coding | 24 |
| ENSG00000151693 | 3.13 | 3.93E-27 | ASAP2 | Protein coding | 24 |
| ENSG00000013588 | 3.13 | 1.17E-05 | GPRC5A | Protein coding | 24 |
| ENSG00000121900 | 3.13 | 5.88E-09 | TMEM54 | Protein coding | 24 |
| ENSG00000198829 | 3.12 | 1.22E-10 | SUCNR1 | Protein coding | 24 |
| ENSG00000180061 | 3.12 | 7.17E-35 | TMEM150B | Protein coding | 24 |
| ENSG00000198814 | 3.12 | 4.10E-158 | GK | Protein coding | 24 |
| ENSG00000112116 | 3.11 | 1.39E-05 | IL17F | Protein coding | 24 |
| ENSG00000060982 | 3.10 | 2.15E-65 | BCAT1 | Protein coding | 24 |
| ENSG00000175899 | -3.09 | 5.08E-34 | A2M | Protein coding | 24 |
| ENSG00000137869 | 3.09 | 2.93E-08 | CYP19A1 | Protein coding | 24 |
| ENSG00000144681 | 3.08 | 6.11E-10 | STAC | Protein coding | 24 |
| ENSG00000104055 | -3.07 | 6.51E-11 | TGM5 | Protein coding | 24 |
| ENSG00000128342 | 3.07 | 4.20E-22 | LIF | Protein coding | 24 |
| ENSG00000154451 | 3.06 | 1.82E-48 | GBP5 | Protein coding | 24 |
| ENSG00000124490 | -3.06 | 3.60E-06 | CRISP2 | Protein coding | 24 |
| ENSG00000177575 | 3.06 | 1.03E-17 | CD163 | Protein coding | 24 |
| ENSG00000052795 | 3.06 | 9.14E-73 | FNIP2 | Protein coding | 24 |
| ENSG00000151790 | 3.05 | 2.27E-06 | TDO2 | Protein coding | 24 |
| ENSG00000198535 | 3.05 | 4.84E-08 | C2CD4A | Protein coding | 24 |
| ENSG00000165105 | 3.04 | 2.41E-05 | RASEF | Protein coding | 24 |
| ENSG00000185338 | 3.04 | 2.83E-42 | SOCS1 | Protein coding | 24 |
| ENSG00000144837 | 3.03 | 8.83E-09 | PLA1A | Protein coding | 24 |
| ENSG00000104951 | 3.03 | 1.39E-10 | IL4I1 | Protein coding | 24 |
| ENSG00000134256 | -3.03 | 1.33E-46 | CD101 | Protein coding | 24 |
| ENSG00000134321 | 3.01 | 1.04E-07 | RSAD2 | Protein coding | 24 |
| ENSG00000205502 | 3.01 | 2.34E-06 | C2CD4B | Protein coding | 24 |
| ENSG00000077063 | -3.01 | 9.15E-11 | CTTNBP2 | Protein coding | 24 |
| ENSG00000180871 | -3.00 | 1.09E-36 | CXCR2 | Protein coding | 24 |
| ENSG00000178860 | 2.99 | 8.32E-44 | MSC | Protein coding | 24 |
| ENSG00000104140 | 2.99 | 2.39E-08 | RHOV | Protein coding | 24 |
| ENSG00000147145 | 2.99 | 9.05E-07 | LPAR4 | Protein coding | 24 |
| ENSG00000111344 | -2.99 | 8.16E-37 | RASAL1 | Protein coding | 24 |
| ENSG00000198719 | 2.99 | 4.52E-44 | DLL1 | Protein coding | 24 |
| ENSG00000158428 | 2.98 | 4.96E-10 | C2orf62 | Protein coding | 24 |
| ENSG00000075420 | 2.98 | 4.75E-128 | FNDC3B | Protein coding | 24 |
| ENSG00000121807 | -2.98 | 3.57E-49 | CCR2 | Protein coding | 24 |
| ENSG00000134532 | 2.97 | 2.91E-29 | SOX5 | Protein coding | 24 |
| ENSG00000164683 | 2.97 | 3.15E-08 | HEY1 | Protein coding | 24 |
| ENSG00000259207 | 2.96 | 2.73E-20 | ITGB3 | Protein coding | 24 |
| ENSG00000143333 | 2.96 | 1.25E-47 | RGS16 | Protein coding | 24 |
| ENSG00000130052 | 2.95 | 1.95E-48 | STARD8 | Protein coding | 24 |
| ENSG00000118257 | 2.95 | 8.18E-74 | NRP2 | Protein coding | 24 |
| ENSG00000134871 | 2.95 | 8.48E-11 | COL4A2 | Protein coding | 24 |
| ENSG00000104321 | 2.95 | 7.26E-08 | TRPA1 | Protein coding | 24 |
| ENSG00000109471 | 2.94 | 1.42E-08 | IL2 | Protein coding | 24 |
| ENSG00000165949 | 2.94 | 1.31E-08 | IFI27 | Protein coding | 24 |
| ENSG00000136689 | 2.94 | 7.86E-12 | IL1RN | Protein coding | 24 |
| ENSG00000108950 | 2.94 | 4.14E-45 | FAM20A | Protein coding | 24 |
| ENSG00000171860 | 2.94 | 7.16E-62 | C3AR1 | Protein coding | 24 |
| ENSG00000204103 | 2.92 | 3.24E-25 | MAFB | Protein coding | 24 |
| ENSG00000243440 | 2.92 | 2.97E-28 | AF165138.7 | Protein coding | 24 |
| ENSG00000124107 | 2.92 | 5.32E-20 | SLPI | Protein coding | 24 |
| ENSG00000095970 | -2.91 | 3.45E-14 | TREM2 | Protein coding | 24 |
| ENSG00000123342 | 2.90 | 1.30E-18 | MMP19 | Protein coding | 24 |
| ENSG00000174348 | -2.90 | 1.17E-08 | PODN | Protein coding | 24 |
| ENSG00000168952 | -2.88 | 2.69E-07 | STXBP6 | Protein coding | 24 |
| ENSG00000176170 | 2.88 | 1.67E-41 | SPHK1 | Protein coding | 24 |
| ENSG00000147650 | 2.88 | 1.80E-51 | LRP12 | Protein coding | 24 |
| ENSG00000101333 | 2.88 | 4.07E-07 | PLCB4 | Protein coding | 24 |
| ENSG00000111961 | 2.87 | 5.99E-65 | SASH1 | Protein coding | 24 |
| ENSG00000133657 | 2.87 | 1.04E-83 | ATP13A3 | Protein coding | 24 |
| ENSG00000134470 | 2.87 | 1.35E-41 | IL15RA | Protein coding | 24 |
| ENSG00000149948 | 2.87 | 4.13E-07 | HMGA2 | Protein coding | 24 |
| ENSG00000158488 | -2.87 | 1.13E-05 | CD1E | Protein coding | 24 |
| ENSG00000139832 | 2.87 | 3.12E-46 | RAB20 | Protein coding | 24 |
| ENSG00000029153 | 2.87 | 3.77E-60 | ARNTL2 | Protein coding | 24 |
| ENSG00000174145 | 2.86 | 9.31E-05 | KIAA1239 | Protein coding | 24 |
| ENSG00000136205 | 2.86 | 3.38E-40 | TNS3 | Protein coding | 24 |
| ENSG00000148734 | 2.86 | 5.55E-05 | NPFFR1 | Protein coding | 24 |
| ENSG00000100448 | -2.85 | 0.000103489 | CTSG | Protein coding | 24 |
| ENSG00000146070 | 2.85 | 2.18E-20 | PLA2G7 | Protein coding | 24 |
| ENSG00000134326 | 2.85 | 6.78E-08 | CMPK2 | Protein coding | 24 |
| ENSG00000152503 | 2.84 | 1.84E-26 | TRIM36 | Protein coding | 24 |
| ENSG00000162512 | 2.84 | 4.60E-14 | SDC3 | Protein coding | 24 |
| ENSG00000135678 | 2.84 | 4.61E-57 | CPM | Protein coding | 24 |
| ENSG00000224821 | 2.84 | 6.70E-06 | COL4A2-AS2 | Protein coding | 24 |
| ENSG00000115107 | 2.84 | 8.66E-21 | STEAP3 | Protein coding | 24 |
| ENSG00000166278 | 2.84 | 1.68E-10 | C2 | Protein coding | 24 |
| ENSG00000175262 | -2.84 | 2.91E-07 | C1orf127 | Protein coding | 24 |
| ENSG00000137809 | -2.84 | 1.33E-06 | ITGA11 | Protein coding | 24 |
| ENSG00000105889 | 2.83 | 6.68E-11 | STEAP1B | Protein coding | 24 |
| ENSG00000177098 | -2.83 | 2.33E-05 | SCN4B | Protein coding | 24 |
| ENSG00000154252 | 2.83 | 9.60E-08 | GAL3ST2 | Protein coding | 24 |
| ENSG00000129009 | 2.82 | 0.000123752 | ISLR | Protein coding | 24 |
| ENSG00000186583 | 2.82 | 1.32E-20 | SPATC1 | Protein coding | 24 |
| ENSG00000188056 | 2.82 | 3.33E-18 | TREML4 | Protein coding | 24 |
| ENSG00000078098 | 2.81 | 2.96E-07 | FAP | Protein coding | 24 |
| ENSG00000101187 | 2.81 | 6.31E-124 | SLCO4A1 | Protein coding | 24 |
| ENSG00000090339 | 2.80 | 3.16E-45 | ICAM1 | Protein coding | 24 |
| ENSG00000124491 | -2.80 | 2.00E-23 | F13A1 | Protein coding | 24 |
| ENSG00000105246 | 2.79 | 1.37E-57 | EBI3 | Protein coding | 24 |
| ENSG00000176076 | 2.79 | 2.34E-13 | KCNE1L | Protein coding | 24 |
| ENSG00000164825 | 2.79 | 8.55E-05 | DEFB1 | Protein coding | 24 |
| ENSG00000179630 | 2.79 | 1.64E-60 | LACC1 | Protein coding | 24 |
| ENSG00000184221 | 2.78 | 1.13E-06 | OLIG1 | Protein coding | 24 |
| ENSG00000151012 | 2.78 | 1.76E-60 | SLC7A11 | Protein coding | 24 |
| ENSG00000140105 | 2.77 | 1.36E-19 | WARS | Protein coding | 24 |
| ENSG00000138061 | 2.77 | 1.30E-63 | CYP1B1 | Protein coding | 24 |
| ENSG00000197301 | 2.77 | 2.07E-08 | RP11-366L20.2 | Protein coding | 24 |
| ENSG00000177294 | 2.77 | 3.00E-09 | FBXO39 | Protein coding | 24 |
| ENSG00000113070 | 2.77 | 2.09E-29 | HBEGF | Protein coding | 24 |
| ENSG00000179331 | 2.76 | 4.55E-28 | RAB39A | Protein coding | 24 |
| ENSG00000106341 | 2.76 | 1.79E-18 | PPP1R17 | Protein coding | 24 |
| ENSG00000169184 | 2.76 | 5.59E-51 | MN1 | Protein coding | 24 |
| ENSG00000173334 | 2.76 | 8.52E-107 | TRIB1 | Protein coding | 24 |
| ENSG00000107130 | 2.75 | 1.48E-28 | NCS1 | Protein coding | 24 |
| ENSG00000116990 | -2.75 | 3.44E-32 | MYCL1 | Protein coding | 24 |
| ENSG00000119917 | 2.75 | 1.97E-06 | IFIT3 | Protein coding | 24 |
| ENSG00000140379 | 2.75 | 2.90E-121 | BCL2A1 | Protein coding | 24 |
| ENSG00000137959 | 2.74 | 2.02E-07 | IFI44L | Protein coding | 24 |
| ENSG00000136810 | 2.73 | 2.36E-68 | TXN | Protein coding | 24 |
| ENSG00000135424 | 2.73 | 4.37E-14 | ITGA7 | Protein coding | 24 |
| ENSG00000004846 | 2.73 | 0.000151362 | ABCB5 | Protein coding | 24 |
| ENSG00000158714 | 2.72 | 3.90E-11 | SLAMF8 | Protein coding | 24 |
| ENSG00000004468 | 2.72 | 3.60E-51 | CD38 | Protein coding | 24 |
| ENSG00000118322 | 2.72 | 7.21E-14 | ATP10B | Protein coding | 24 |
| ENSG00000109099 | 2.72 | 6.38E-14 | PMP22 | Protein coding | 24 |
| ENSG00000036530 | -2.71 | 9.78E-06 | CYP46A1 | Protein coding | 24 |
| ENSG00000092200 | -2.71 | 4.18E-13 | RPGRIP1 | Protein coding | 24 |
| ENSG00000001617 | 2.70 | 1.49E-07 | SEMA3F | Protein coding | 24 |
| ENSG00000135821 | 2.70 | 1.03E-40 | GLUL | Protein coding | 24 |
| ENSG00000135218 | -2.70 | 6.74E-35 | CD36 | Protein coding | 24 |
| ENSG00000205364 | 2.69 | 0.000301407 | MT1M | Protein coding | 24 |
| ENSG00000137462 | 2.69 | 3.92E-51 | TLR2 | Protein coding | 24 |
| ENSG00000132669 | 2.68 | 4.33E-43 | RIN2 | Protein coding | 24 |
| ENSG00000073712 | 2.68 | 3.17E-22 | FERMT2 | Protein coding | 24 |
| ENSG00000108684 | 2.68 | 0.000329646 | ASIC2 | Protein coding | 24 |
| ENSG00000155254 | -2.68 | 1.88E-31 | MARVELD1 | Protein coding | 24 |
| ENSG00000163814 | 2.67 | 4.69E-42 | CDCP1 | Protein coding | 24 |
| ENSG00000135636 | 2.67 | 1.96E-11 | DYSF | Protein coding | 24 |
| ENSG00000114248 | 2.67 | 2.30E-06 | LRRC31 | Protein coding | 24 |
| ENSG00000089041 | 2.67 | 1.08E-15 | P2RX7 | Protein coding | 24 |
| ENSG00000138356 | 2.67 | 0.000221832 | AOX1 | Protein coding | 24 |
| ENSG00000185052 | 2.67 | 1.72E-18 | SLC24A3 | Protein coding | 24 |
| ENSG00000169245 | 2.67 | 3.21E-05 | CXCL10 | Protein coding | 24 |
| ENSG00000146374 | 2.67 | 0.000357433 | RSPO3 | Protein coding | 24 |
| ENSG00000155629 | 2.66 | 4.26E-88 | PIK3AP1 | Protein coding | 24 |
| ENSG00000157827 | 2.65 | 1.47E-69 | FMNL2 | Protein coding | 24 |
| ENSG00000064787 | 2.65 | 7.68E-23 | BCAS1 | Protein coding | 24 |
| ENSG00000131979 | 2.65 | 5.59E-91 | GCH1 | Protein coding | 24 |
| ENSG00000165935 | 2.64 | 3.41E-05 | C12orf70 | Protein coding | 24 |
| ENSG00000104918 | 2.64 | 3.55E-16 | RETN | Protein coding | 24 |
| ENSG00000158473 | -2.64 | 6.85E-24 | CD1D | Protein coding | 24 |
| ENSG00000117013 | 2.64 | 5.36E-15 | KCNQ4 | Protein coding | 24 |
| ENSG00000180113 | 2.64 | 1.28E-57 | TDRD6 | Protein coding | 24 |
| ENSG00000059728 | 2.64 | 4.41E-68 | MXD1 | Protein coding | 24 |
| ENSG00000134070 | 2.63 | 7.47E-65 | IRAK2 | Protein coding | 24 |
| ENSG00000101188 | 2.63 | 4.02E-11 | NTSR1 | Protein coding | 24 |
| ENSG00000158869 | 2.63 | 2.68E-53 | FCER1G | Protein coding | 24 |
| ENSG00000140859 | 2.61 | 1.04E-16 | KIFC3 | Protein coding | 24 |
| ENSG00000075618 | 2.60 | 6.27E-83 | FSCN1 | Protein coding | 24 |
| ENSG00000101336 | 2.60 | 2.04E-51 | HCK | Protein coding | 24 |
| ENSG00000116663 | 2.60 | 7.68E-12 | FBXO6 | Protein coding | 24 |
| ENSG00000165621 | -2.60 | 0.000550813 | OXGR1 | Protein coding | 24 |
| ENSG00000171659 | -2.60 | 1.74E-15 | GPR34 | Protein coding | 24 |
| ENSG00000215306 | 2.59 | 8.20E-07 | AL135998.1 | Protein coding | 24 |
| ENSG00000188290 | 2.59 | 0.000224591 | HES4 | Protein coding | 24 |
| ENSG00000197249 | 2.59 | 4.87E-18 | SERPINA1 | Protein coding | 24 |
| ENSG00000182782 | 2.59 | 1.90E-25 | HCAR2 | Protein coding | 24 |
| ENSG00000106853 | 2.59 | 1.20E-19 | PTGR1 | Protein coding | 24 |
| ENSG00000254087 | 2.58 | 2.36E-81 | LYN | Protein coding | 24 |
| ENSG00000109265 | 2.58 | 1.62E-12 | KIAA1211 | Protein coding | 24 |
| ENSG00000075223 | 2.58 | 6.62E-56 | SEMA3C | Protein coding | 24 |
| ENSG00000025434 | 2.57 | 7.77E-14 | NR1H3 | Protein coding | 24 |
| ENSG00000174502 | 2.57 | 6.76E-05 | SLC26A9 | Protein coding | 24 |
| ENSG00000185897 | 2.57 | 0.000668788 | FFAR3 | Protein coding | 24 |
| ENSG00000197046 | 2.57 | 1.77E-15 | SIGLEC15 | Protein coding | 24 |
| ENSG00000104972 | 2.56 | 2.07E-37 | LILRB1 | Protein coding | 24 |
| ENSG00000140279 | 2.56 | 5.68E-20 | DUOX2 | Protein coding | 24 |
| ENSG00000188313 | 2.56 | 9.63E-19 | PLSCR1 | Protein coding | 24 |
| ENSG00000100024 | 2.56 | 2.49E-28 | UPB1 | Protein coding | 24 |
| ENSG00000175352 | 2.56 | 3.08E-25 | NRIP3 | Protein coding | 24 |
| ENSG00000205020 | 2.55 | 3.61E-07 | CCL4L1 | Protein coding | 24 |
| ENSG00000148677 | 2.55 | 0.000107234 | ANKRD1 | Protein coding | 24 |
| ENSG00000141161 | -2.55 | 1.22E-05 | UNC45B | Protein coding | 24 |
| ENSG00000179431 | 2.55 | 4.78E-06 | FJX1 | Protein coding | 24 |
| ENSG00000007350 | -2.54 | 2.73E-14 | TKTL1 | Protein coding | 24 |
| ENSG00000124780 | -2.54 | 0.000772853 | KCNK17 | Protein coding | 24 |
| ENSG00000161381 | -2.54 | 5.76E-22 | PLXDC1 | Protein coding | 24 |
| ENSG00000123685 | 2.53 | 1.52E-23 | BATF3 | Protein coding | 24 |
| ENSG00000002587 | 2.53 | 8.85E-07 | HS3ST1 | Protein coding | 24 |
| ENSG00000268865 | 2.52 | 1.69E-05 | AC026310.1 | Protein coding | 24 |
| ENSG00000255398 | 2.52 | 1.36E-15 | HCAR3 | Protein coding | 24 |
| ENSG00000166165 | 2.52 | 4.66E-20 | CKB | Protein coding | 24 |
| ENSG00000182566 | 2.52 | 2.73E-06 | CLEC4G | Protein coding | 24 |
| ENSG00000147454 | 2.52 | 1.69E-38 | SLC25A37 | Protein coding | 24 |
| ENSG00000176049 | 2.51 | 4.40E-38 | JAKMIP2 | Protein coding | 24 |
| ENSG00000163694 | 2.51 | 7.20E-45 | RBM47 | Protein coding | 24 |
| ENSG00000159399 | 2.51 | 8.62E-41 | HK2 | Protein coding | 24 |
| ENSG00000235531 | 2.51 | 2.64E-20 | RP11-383H13.1 | Protein coding | 24 |
| ENSG00000155465 | 2.51 | 2.21E-31 | SLC7A7 | Protein coding | 24 |
| ENSG00000184979 | 2.51 | 2.30E-06 | USP18 | Protein coding | 24 |
| ENSG00000130202 | 2.51 | 3.06E-17 | PVRL2 | Protein coding | 24 |
| ENSG00000152580 | -2.50 | 9.73E-07 | IGSF10 | Protein coding | 24 |
| ENSG00000077585 | 2.50 | 6.41E-94 | GPR137B | Protein coding | 24 |
| ENSG00000112394 | 2.49 | 2.48E-08 | SLC16A10 | Protein coding | 24 |
| ENSG00000137393 | 2.49 | 7.52E-111 | RNF144B | Protein coding | 24 |
| ENSG00000205436 | 2.48 | 0.000247701 | EXOC3L4 | Protein coding | 24 |
| ENSG00000137331 | 2.48 | 7.53E-47 | IER3 | Protein coding | 24 |
| ENSG00000187116 | 2.48 | 9.38E-11 | LILRA5 | Protein coding | 24 |
| ENSG00000135094 | 2.48 | 9.63E-07 | SDS | Protein coding | 24 |
| ENSG00000002549 | 2.48 | 7.34E-11 | LAP3 | Protein coding | 24 |
| ENSG00000104093 | 2.47 | 1.76E-61 | DMXL2 | Protein coding | 24 |
| ENSG00000188676 | 2.47 | 4.30E-24 | IDO2 | Protein coding | 24 |
| ENSG00000131042 | 2.47 | 1.41E-30 | LILRB2 | Protein coding | 24 |
| ENSG00000251139 | 2.47 | 0.001184228 | RP11-701P16.2 | Protein coding | 24 |
| ENSG00000228835 | -2.46 | 8.72E-07 | AC012123.1 | Protein coding | 24 |
| ENSG00000120586 | 2.46 | 2.03E-06 | MRC1 | Protein coding | 24 |
| ENSG00000163293 | -2.46 | 2.68E-12 | NIPAL1 | Protein coding | 24 |
| ENSG00000180509 | 2.46 | 2.55E-24 | KCNE1 | Protein coding | 24 |
| ENSG00000149573 | -2.46 | 3.02E-19 | MPZL2 | Protein coding | 24 |
| ENSG00000105419 | 2.45 | 2.88E-05 | MEIS3 | Protein coding | 24 |
| ENSG00000110079 | 2.45 | 1.15E-09 | MS4A4A | Protein coding | 24 |
| ENSG00000131669 | 2.45 | 1.97E-43 | NINJ1 | Protein coding | 24 |
| ENSG00000178445 | 2.45 | 2.91E-09 | GLDC | Protein coding | 24 |
| ENSG00000213512 | 2.45 | 1.07E-17 | GBP7 | Protein coding | 24 |
| ENSG00000133116 | 2.45 | 3.87E-16 | KL | Protein coding | 24 |
| ENSG00000114737 | 2.45 | 2.51E-26 | CISH | Protein coding | 24 |
| ENSG00000133106 | 2.44 | 3.12E-09 | EPSTI1 | Protein coding | 24 |
| ENSG00000122043 | -2.44 | 1.08E-06 | LINC00544 | Protein coding | 24 |
| ENSG00000120129 | 2.44 | 4.94E-56 | DUSP1 | Protein coding | 24 |
| ENSG00000157064 | 2.44 | 0.000362326 | NMNAT2 | Protein coding | 24 |
| ENSG00000188786 | 2.44 | 5.91E-71 | MTF1 | Protein coding | 24 |
| ENSG00000175183 | 2.44 | 0.000644281 | CSRP2 | Protein coding | 24 |
| ENSG00000155130 | 2.43 | 3.27E-64 | MARCKS | Protein coding | 24 |
| ENSG00000178726 | 2.43 | 1.10E-21 | THBD | Protein coding | 24 |
| ENSG00000115415 | 2.43 | 3.56E-10 | STAT1 | Protein coding | 24 |
| ENSG00000197122 | 2.43 | 5.18E-33 | SRC | Protein coding | 24 |
| ENSG00000104432 | 2.43 | 6.73E-42 | IL7 | Protein coding | 24 |
| ENSG00000172216 | 2.43 | 3.39E-35 | CEBPB | Protein coding | 24 |
| ENSG00000103196 | 2.42 | 3.38E-38 | CRISPLD2 | Protein coding | 24 |
| ENSG00000123095 | 2.42 | 4.13E-35 | BHLHE41 | Protein coding | 24 |
| ENSG00000180251 | 2.42 | 0.000133584 | SLC9A4 | Protein coding | 24 |
| ENSG00000162897 | 2.42 | 0.000393791 | FCAMR | Protein coding | 24 |
| ENSG00000110077 | -2.40 | 8.53E-24 | MS4A6A | Protein coding | 24 |
| ENSG00000058085 | 2.40 | 9.64E-06 | LAMC2 | Protein coding | 24 |
| ENSG00000160593 | -2.40 | 3.55E-58 | AMICA1 | Protein coding | 24 |
| ENSG00000170448 | -2.39 | 1.05E-111 | NFXL1 | Protein coding | 24 |
| ENSG00000103313 | 2.39 | 1.09E-28 | MEFV | Protein coding | 24 |
| ENSG00000185215 | 2.38 | 9.50E-39 | TNFAIP2 | Protein coding | 24 |
| ENSG00000100906 | 2.37 | 8.18E-74 | NFKBIA | Protein coding | 24 |
| ENSG00000169403 | 2.36 | 1.94E-29 | PTAFR | Protein coding | 24 |
| ENSG00000179813 | 2.36 | 0.001255759 | FAM216B | Protein coding | 24 |
| ENSG00000130066 | 2.36 | 2.54E-59 | SAT1 | Protein coding | 24 |
| ENSG00000077327 | 2.36 | 0.002189924 | SPAG6 | Protein coding | 24 |
| ENSG00000101460 | 2.35 | 3.60E-17 | MAP1LC3A | Protein coding | 24 |
| ENSG00000078114 | -2.35 | 7.03E-05 | NEBL | Protein coding | 24 |
| ENSG00000184500 | 2.34 | 1.29E-05 | PROS1 | Protein coding | 24 |
| ENSG00000113916 | 2.34 | 3.08E-131 | BCL6 | Protein coding | 24 |
| ENSG00000185499 | 2.34 | 3.10E-15 | MUC1 | Protein coding | 24 |
| ENSG00000162654 | 2.34 | 2.82E-37 | GBP4 | Protein coding | 24 |
| ENSG00000088826 | 2.33 | 2.64E-23 | SMOX | Protein coding | 24 |
| ENSG00000139318 | 2.33 | 5.15E-33 | DUSP6 | Protein coding | 24 |
| ENSG00000170961 | 2.33 | 0.002546268 | HAS2 | Protein coding | 24 |
| ENSG00000187796 | -2.33 | 3.38E-39 | CARD9 | Protein coding | 24 |
| ENSG00000158470 | 2.33 | 2.83E-52 | B4GALT5 | Protein coding | 24 |
| ENSG00000153246 | 2.32 | 0.000255929 | PLA2R1 | Protein coding | 24 |
| ENSG00000176788 | 2.32 | 3.11E-65 | BASP1 | Protein coding | 24 |
| ENSG00000163220 | 2.32 | 6.47E-10 | S100A9 | Protein coding | 24 |
| ENSG00000186907 | 2.31 | 1.10E-06 | RTN4RL2 | Protein coding | 24 |
| ENSG00000095203 | -2.31 | 0.000152566 | EPB41L4B | Protein coding | 24 |
| ENSG00000171596 | -2.31 | 2.16E-17 | NMUR1 | Protein coding | 24 |
| ENSG00000173805 | -2.31 | 6.24E-05 | HAP1 | Protein coding | 24 |
| ENSG00000143067 | 2.31 | 4.66E-46 | ZNF697 | Protein coding | 24 |
| ENSG00000254521 | 2.30 | 2.67E-07 | SIGLEC12 | Protein coding | 24 |
| ENSG00000159713 | -2.30 | 0.001308233 | TPPP3 | Protein coding | 24 |
| ENSG00000078401 | 2.30 | 2.73E-24 | EDN1 | Protein coding | 24 |
| ENSG00000164733 | 2.30 | 1.76E-29 | CTSB | Protein coding | 24 |
| ENSG00000186431 | 2.30 | 1.52E-25 | FCAR | Protein coding | 24 |
| ENSG00000107562 | 2.29 | 0.001316133 | CXCL12 | Protein coding | 24 |
| ENSG00000135373 | 2.29 | 1.15E-16 | EHF | Protein coding | 24 |
| ENSG00000168615 | 2.29 | 8.33E-67 | ADAM9 | Protein coding | 24 |
| ENSG00000134802 | 2.29 | 1.64E-44 | SLC43A3 | Protein coding | 24 |
| ENSG00000197208 | 2.29 | 2.89E-22 | SLC22A4 | Protein coding | 24 |
| ENSG00000042493 | 2.29 | 3.98E-16 | CAPG | Protein coding | 24 |
| ENSG00000196839 | 2.29 | 2.01E-58 | ADA | Protein coding | 24 |
| ENSG00000120833 | 2.28 | 1.21E-17 | SOCS2 | Protein coding | 24 |
| ENSG00000177409 | 2.28 | 1.63E-12 | SAMD9L | Protein coding | 24 |
| ENSG00000196935 | 2.28 | 4.37E-18 | SRGAP1 | Protein coding | 24 |
| ENSG00000181577 | 2.28 | 1.28E-12 | C6orf223 | Protein coding | 24 |
| ENSG00000198417 | 2.28 | 8.81E-33 | MT1F | Protein coding | 24 |
| ENSG00000134853 | 2.27 | 0.000398803 | PDGFRA | Protein coding | 24 |
| ENSG00000187678 | 2.27 | 0.001127899 | SPRY4 | Protein coding | 24 |
| ENSG00000008394 | 2.26 | 2.25E-24 | MGST1 | Protein coding | 24 |
| ENSG00000138496 | 2.26 | 2.32E-11 | PARP9 | Protein coding | 24 |
| ENSG00000024422 | 2.26 | 9.46E-05 | EHD2 | Protein coding | 24 |
| ENSG00000114315 | 2.25 | 1.25E-07 | HES1 | Protein coding | 24 |
| ENSG00000171989 | 2.25 | 1.16E-05 | LDHAL6B | Protein coding | 24 |
| ENSG00000154856 | 2.25 | 2.79E-06 | APCDD1 | Protein coding | 24 |
| ENSG00000137965 | 2.25 | 9.13E-07 | IFI44 | Protein coding | 24 |
| ENSG00000138080 | 2.25 | 2.57E-16 | EMILIN1 | Protein coding | 24 |
| ENSG00000183347 | 2.25 | 4.52E-11 | GBP6 | Protein coding | 24 |
| ENSG00000168334 | 2.24 | 7.97E-06 | XIRP1 | Protein coding | 24 |
| ENSG00000170681 | 2.24 | 4.87E-08 | MURC | Protein coding | 24 |
| ENSG00000128383 | 2.24 | 2.72E-05 | APOBEC3A | Protein coding | 24 |
| ENSG00000171657 | -2.24 | 1.19E-08 | GPR82 | Protein coding | 24 |
| ENSG00000153132 | 2.24 | 1.88E-05 | CLGN | Protein coding | 24 |
| ENSG00000185686 | 2.24 | 0.001527004 | PRAME | Protein coding | 24 |
| ENSG00000108244 | 2.23 | 4.32E-06 | KRT23 | Protein coding | 24 |
| ENSG00000128641 | 2.23 | 5.43E-18 | MYO1B | Protein coding | 24 |
| ENSG00000078081 | 2.23 | 6.88E-48 | LAMP3 | Protein coding | 24 |
| ENSG00000136002 | -2.23 | 2.91E-07 | ARHGEF4 | Protein coding | 24 |
| ENSG00000115919 | 2.23 | 2.65E-76 | KYNU | Protein coding | 24 |
| ENSG00000152229 | 2.23 | 2.00E-37 | PSTPIP2 | Protein coding | 24 |
| ENSG00000186354 | 2.23 | 0.001785847 | C9orf47 | Protein coding | 24 |
| ENSG00000111331 | 2.22 | 1.17E-05 | OAS3 | Protein coding | 24 |
| ENSG00000106066 | -2.22 | 3.54E-21 | CPVL | Protein coding | 24 |
| ENSG00000113763 | 2.22 | 0.000894676 | UNC5A | Protein coding | 24 |
| ENSG00000124145 | 2.22 | 9.04E-37 | SDC4 | Protein coding | 24 |
| ENSG00000157766 | 2.22 | 0.000407066 | ACAN | Protein coding | 24 |
| ENSG00000169862 | 2.22 | 0.001052495 | CTNND2 | Protein coding | 24 |
| ENSG00000136026 | 2.22 | 2.98E-37 | CKAP4 | Protein coding | 24 |
| ENSG00000189325 | 2.21 | 0.000742465 | C6orf222 | Protein coding | 24 |
| ENSG00000265096 | 2.21 | 0.004481273 | AC073624.1 | Protein coding | 24 |
| ENSG00000138119 | 2.21 | 1.26E-08 | MYOF | Protein coding | 24 |
| ENSG00000169248 | 2.21 | 0.000159689 | CXCL11 | Protein coding | 24 |
| ENSG00000254827 | 2.21 | 1.47E-11 | SLC22A18AS | Protein coding | 24 |
| ENSG00000254415 | 2.20 | 7.44E-27 | SIGLEC14 | Protein coding | 24 |
| ENSG00000156219 | 2.20 | 0.004036546 | ART3 | Protein coding | 24 |
| ENSG00000198682 | 2.20 | 1.75E-33 | PAPSS2 | Protein coding | 24 |
| ENSG00000221887 | 2.20 | 5.19E-05 | HMSD | Protein coding | 24 |
| ENSG00000175857 | -2.19 | 1.44E-29 | GAPT | Protein coding | 24 |
| ENSG00000165914 | 2.19 | 2.37E-17 | TTC7B | Protein coding | 24 |
| ENSG00000124762 | 2.19 | 2.97E-30 | CDKN1A | Protein coding | 24 |
| ENSG00000176046 | 2.19 | 0.003046373 | NUPR1 | Protein coding | 24 |
| ENSG00000104368 | 2.19 | 4.30E-19 | PLAT | Protein coding | 24 |
| ENSG00000136514 | 2.18 | 7.09E-17 | RTP4 | Protein coding | 24 |
| ENSG00000170486 | -2.18 | 3.53E-45 | KRT72 | Protein coding | 24 |
| ENSG00000214274 | -2.18 | 1.53E-08 | ANG | Protein coding | 24 |
| ENSG00000104312 | 2.18 | 7.30E-45 | RIPK2 | Protein coding | 24 |
| ENSG00000213886 | 2.18 | 0.005312953 | UBD | Protein coding | 24 |
| ENSG00000162745 | 2.18 | 1.47E-06 | OLFML2B | Protein coding | 24 |
| ENSG00000221963 | 2.17 | 8.10E-41 | APOL6 | Protein coding | 24 |
| ENSG00000149635 | 2.17 | 2.33E-08 | OCSTAMP | Protein coding | 24 |
| ENSG00000095383 | 2.17 | 8.46E-22 | TBC1D2 | Protein coding | 24 |
| ENSG00000130368 | 2.17 | 0.005513906 | MAS1 | Protein coding | 24 |
| ENSG00000116514 | 2.17 | 1.64E-43 | RNF19B | Protein coding | 24 |
| ENSG00000149927 | -2.17 | 0.000750156 | DOC2A | Protein coding | 24 |
| ENSG00000225485 | 2.16 | 4.92E-09 | ARHGAP23 | Protein coding | 24 |
| ENSG00000132530 | 2.16 | 9.92E-08 | XAF1 | Protein coding | 24 |
| ENSG00000143545 | 2.16 | 1.18E-26 | RAB13 | Protein coding | 24 |
| ENSG00000117009 | 2.16 | 2.93E-39 | KMO | Protein coding | 24 |
| ENSG00000042062 | 2.16 | 1.23E-20 | FAM65C | Protein coding | 24 |
| ENSG00000136960 | 2.16 | 2.44E-22 | ENPP2 | Protein coding | 24 |
| ENSG00000167996 | 2.16 | 1.32E-28 | FTH1 | Protein coding | 24 |
| ENSG00000166068 | 2.15 | 4.71E-23 | SPRED1 | Protein coding | 24 |
| ENSG00000154027 | -2.15 | 2.57E-14 | AK5 | Protein coding | 24 |
| ENSG00000149557 | 2.15 | 1.82E-23 | FEZ1 | Protein coding | 24 |
| ENSG00000198369 | 2.15 | 1.05E-25 | SPRED2 | Protein coding | 24 |
| ENSG00000111452 | -2.15 | 1.37E-14 | GPR133 | Protein coding | 24 |
| ENSG00000120306 | 2.14 | 1.51E-26 | CYSTM1 | Protein coding | 24 |
| ENSG00000149131 | 2.14 | 0.001446176 | SERPING1 | Protein coding | 24 |
| ENSG00000142185 | 2.13 | 7.82E-12 | TRPM2 | Protein coding | 24 |
| ENSG00000163464 | -2.13 | 2.58E-12 | CXCR1 | Protein coding | 24 |
| ENSG00000232810 | 2.13 | 6.46E-43 | TNF | Protein coding | 24 |
| ENSG00000138166 | 2.13 | 2.87E-59 | DUSP5 | Protein coding | 24 |
| ENSG00000185022 | 2.13 | 3.07E-34 | MAFF | Protein coding | 24 |
| ENSG00000143546 | 2.13 | 9.86E-12 | S100A8 | Protein coding | 24 |
| ENSG00000147570 | 2.12 | 2.82E-11 | DNAJC5B | Protein coding | 24 |
| ENSG00000100368 | 2.12 | 3.30E-31 | CSF2RB | Protein coding | 24 |
| ENSG00000087116 | 2.12 | 0.001509628 | ADAMTS2 | Protein coding | 24 |
| ENSG00000187848 | 2.12 | 0.003433758 | P2RX2 | Protein coding | 24 |
| ENSG00000196141 | 2.12 | 7.59E-08 | SPATS2L | Protein coding | 24 |
| ENSG00000127838 | 2.11 | 1.60E-37 | PNKD | Protein coding | 24 |
| ENSG00000160326 | 2.11 | 1.13E-24 | SLC2A6 | Protein coding | 24 |
| ENSG00000184988 | 2.11 | 4.68E-38 | TMEM106A | Protein coding | 24 |
| ENSG00000124216 | 2.11 | 1.81E-05 | SNAI1 | Protein coding | 24 |
| ENSG00000134575 | 2.10 | 2.66E-23 | ACP2 | Protein coding | 24 |
| ENSG00000135124 | 2.10 | 1.98E-34 | P2RX4 | Protein coding | 24 |
| ENSG00000095209 | 2.10 | 1.86E-35 | TMEM38B | Protein coding | 24 |
| ENSG00000188215 | 2.10 | 1.09E-33 | DCUN1D3 | Protein coding | 24 |
| ENSG00000119922 | 2.09 | 6.62E-06 | IFIT2 | Protein coding | 24 |
| ENSG00000160883 | 2.09 | 1.04E-09 | HK3 | Protein coding | 24 |
| ENSG00000173535 | -2.09 | 6.51E-10 | TNFRSF10C | Protein coding | 24 |
| ENSG00000090376 | 2.09 | 4.91E-40 | IRAK3 | Protein coding | 24 |
| ENSG00000178695 | 2.08 | 9.51E-54 | KCTD12 | Protein coding | 24 |
| ENSG00000121933 | -2.08 | 7.51E-11 | ADORA3 | Protein coding | 24 |
| ENSG00000165694 | 2.08 | 0.008263804 | FRMD7 | Protein coding | 24 |
| ENSG00000100342 | 2.08 | 5.53E-23 | APOL1 | Protein coding | 24 |
| ENSG00000214872 | 2.08 | 9.41E-18 | SMTNL1 | Protein coding | 24 |
| ENSG00000175489 | 2.08 | 1.49E-31 | LRRC25 | Protein coding | 24 |
| ENSG00000113494 | 2.07 | 1.09E-11 | PRLR | Protein coding | 24 |
| ENSG00000185745 | 2.07 | 0.000638608 | IFIT1 | Protein coding | 24 |
| ENSG00000142089 | 2.07 | 0.00010907 | IFITM3 | Protein coding | 24 |
| ENSG00000185222 | 2.07 | 3.28E-18 | WBP5 | Protein coding | 24 |
| ENSG00000163625 | 2.07 | 4.44E-70 | WDFY3 | Protein coding | 24 |
| ENSG00000136867 | 2.07 | 3.20E-16 | SLC31A2 | Protein coding | 24 |
| ENSG00000137441 | -2.06 | 1.94E-39 | FGFBP2 | Protein coding | 24 |
| ENSG00000197405 | 2.06 | 3.72E-38 | C5AR1 | Protein coding | 24 |
| ENSG00000222047 | 2.06 | 0.00116362 | C10orf55 | Protein coding | 24 |
| ENSG00000064201 | -2.06 | 4.40E-74 | TSPAN32 | Protein coding | 24 |
| ENSG00000116962 | 2.06 | 6.23E-20 | NID1 | Protein coding | 24 |
| ENSG00000140274 | 2.06 | 0.001184004 | DUOXA2 | Protein coding | 24 |
| ENSG00000118785 | 2.06 | 9.41E-09 | SPP1 | Protein coding | 24 |
| ENSG00000135604 | 2.05 | 4.56E-28 | STX11 | Protein coding | 24 |
| ENSG00000255221 | 2.05 | 0.00024036 | CARD17 | Protein coding | 24 |
| ENSG00000020577 | 2.05 | 2.30E-17 | SAMD4A | Protein coding | 24 |
| ENSG00000176177 | 2.04 | 9.99E-05 | ENTHD1 | Protein coding | 24 |
| ENSG00000106258 | 2.04 | 6.79E-13 | CYP3A5 | Protein coding | 24 |
| ENSG00000248905 | 2.04 | 1.09E-10 | FMN1 | Protein coding | 24 |
| ENSG00000172867 | -2.03 | 0.000116676 | KRT2 | Protein coding | 24 |
| ENSG00000168899 | 2.03 | 2.43E-19 | VAMP5 | Protein coding | 24 |
| ENSG00000168658 | 2.03 | 0.009119662 | VWA3B | Protein coding | 24 |
| ENSG00000249242 | 2.03 | 0.000181067 | TMEM150C | Protein coding | 24 |
| ENSG00000132003 | 2.02 | 2.08E-38 | ZSWIM4 | Protein coding | 24 |
| ENSG00000130589 | 2.02 | 1.95E-06 | HELZ2 | Protein coding | 24 |
| ENSG00000181381 | 2.02 | 1.49E-29 | DDX60L | Protein coding | 24 |
| ENSG00000205300 | 2.01 | 0.008393252 | RP11-352D3.2 | Protein coding | 24 |
| ENSG00000137628 | 2.01 | 8.57E-12 | DDX60 | Protein coding | 24 |
| ENSG00000125347 | 2.01 | 5.90E-35 | IRF1 | Protein coding | 24 |
| ENSG00000105501 | 2.01 | 2.49E-21 | SIGLEC5 | Protein coding | 24 |
| ENSG00000164111 | 2.01 | 4.55E-38 | ANXA5 | Protein coding | 24 |
| ENSG00000221955 | 2.01 | 1.99E-09 | SLC12A8 | Protein coding | 24 |
| ENSG00000164530 | -2.01 | 1.68E-13 | PI16 | Protein coding | 24 |
| ENSG00000005102 | 2.01 | 4.37E-08 | MEOX1 | Protein coding | 24 |
| ENSG00000156414 | 2.00 | 1.48E-16 | TDRD9 | Protein coding | 24 |
| ENSG00000125462 | 2.00 | 8.41E-11 | C1orf61 | Protein coding | 24 |
| ENSG00000157557 | 2.00 | 2.69E-36 | ETS2 | Protein coding | 24 |
| ENSG00000141505 | -2.00 | 4.08E-13 | ASGR1 | Protein coding | 24 |
| ENSG00000180539 | -2.00 | 2.29E-16 | C9orf139 | Protein coding | 24 |
| ENSG00000187559 | -2.00 | 0.010750358 | FOXD4L3 | Protein coding | 24 |
| ENSG00000168329 | -2.00 | 3.50E-33 | CX3CR1 | Protein coding | 24 |
| ENSG00000162614 | 2.00 | 1.64E-11 | NEXN | Protein coding | 24 |
| ENSG00000153071 | 1.99 | 2.91E-24 | DAB2 | Protein coding | 24 |
| ENSG00000140749 | 1.99 | 2.69E-23 | IGSF6 | Protein coding | 24 |
| ENSG00000131095 | 1.99 | 0.005691734 | GFAP | Protein coding | 24 |
| ENSG00000135838 | 1.99 | 2.28E-24 | NPL | Protein coding | 24 |
| ENSG00000135454 | 1.99 | 0.000258386 | B4GALNT1 | Protein coding | 24 |
| ENSG00000166926 | 1.99 | 0.010016922 | MS4A6E | Protein coding | 24 |
| ENSG00000137767 | 1.99 | 3.84E-28 | SQRDL | Protein coding | 24 |
| ENSG00000187608 | 1.98 | 0.000264203 | ISG15 | Protein coding | 24 |
| ENSG00000152952 | 1.98 | 0.012173805 | PLOD2 | Protein coding | 24 |
| ENSG00000143479 | 1.98 | 2.63E-16 | DYRK3 | Protein coding | 24 |
| ENSG00000104921 | 1.98 | 1.12E-25 | FCER2 | Protein coding | 24 |
| ENSG00000186642 | 1.98 | 3.36E-10 | PDE2A | Protein coding | 24 |
| ENSG00000154734 | -1.97 | 7.26E-08 | ADAMTS1 | Protein coding | 24 |
| ENSG00000183662 | -1.97 | 0.005585141 | FAM19A1 | Protein coding | 24 |
| ENSG00000101916 | 1.97 | 2.18E-39 | TLR8 | Protein coding | 24 |
| ENSG00000173083 | 1.97 | 4.64E-31 | HPSE | Protein coding | 24 |
| ENSG00000047457 | 1.96 | 0.000377288 | CP | Protein coding | 24 |
| ENSG00000131435 | 1.96 | 0.001588978 | PDLIM4 | Protein coding | 24 |
| ENSG00000167034 | 1.96 | 2.53E-15 | NKX3-1 | Protein coding | 24 |
| ENSG00000204577 | 1.96 | 6.25E-19 | LILRB3 | Protein coding | 24 |
| ENSG00000167613 | 1.96 | 2.69E-25 | LAIR1 | Protein coding | 24 |
| ENSG00000182541 | 1.96 | 7.79E-46 | LIMK2 | Protein coding | 24 |
| ENSG00000089169 | -1.96 | 0.013749247 | RPH3A | Protein coding | 24 |
| ENSG00000137094 | 1.95 | 3.01E-28 | DNAJB5 | Protein coding | 24 |
| ENSG00000150687 | -1.95 | 1.83E-22 | PRSS23 | Protein coding | 24 |
| ENSG00000101321 | 1.95 | 0.001927677 | XKR7 | Protein coding | 24 |
| ENSG00000183960 | -1.95 | 1.74E-10 | KCNH8 | Protein coding | 24 |
| ENSG00000117525 | 1.95 | 1.10E-13 | F3 | Protein coding | 24 |
| ENSG00000068079 | 1.95 | 4.10E-07 | IFI35 | Protein coding | 24 |
| ENSG00000197721 | 1.95 | 0.000288286 | CR1L | Protein coding | 24 |
| ENSG00000135503 | 1.95 | 8.13E-28 | ACVR1B | Protein coding | 24 |
| ENSG00000163840 | 1.95 | 2.76E-25 | DTX3L | Protein coding | 24 |
| ENSG00000028137 | 1.95 | 5.37E-37 | TNFRSF1B | Protein coding | 24 |
| ENSG00000137710 | 1.95 | 3.95E-61 | RDX | Protein coding | 24 |
| ENSG00000178175 | 1.94 | 1.15E-12 | ZNF366 | Protein coding | 24 |
| ENSG00000170458 | 1.94 | 8.09E-26 | CD14 | Protein coding | 24 |
| ENSG00000111817 | 1.94 | 6.75E-39 | DSE | Protein coding | 24 |
| ENSG00000197702 | -1.94 | 0.000661811 | PARVA | Protein coding | 24 |
| ENSG00000180875 | 1.94 | 3.66E-20 | GREM2 | Protein coding | 24 |
| ENSG00000162772 | 1.94 | 5.22E-07 | ATF3 | Protein coding | 24 |
| ENSG00000205358 | 1.94 | 0.009342735 | MT1H | Protein coding | 24 |
| ENSG00000120875 | 1.93 | 7.21E-28 | DUSP4 | Protein coding | 24 |
| ENSG00000111319 | -1.93 | 1.64E-07 | SCNN1A | Protein coding | 24 |
| ENSG00000137491 | 1.93 | 8.33E-08 | SLCO2B1 | Protein coding | 24 |
| ENSG00000101384 | 1.93 | 9.09E-28 | JAG1 | Protein coding | 24 |
| ENSG00000111424 | 1.93 | 1.46E-21 | VDR | Protein coding | 24 |
| ENSG00000166002 | 1.93 | 8.23E-08 | C11orf75 | Protein coding | 24 |
| ENSG00000089692 | 1.93 | 6.98E-20 | LAG3 | Protein coding | 24 |
| ENSG00000187726 | -1.92 | 4.36E-08 | DNAJB13 | Protein coding | 24 |
| ENSG00000172794 | -1.92 | 6.19E-55 | RAB37 | Protein coding | 24 |
| ENSG00000129673 | 1.92 | 3.80E-14 | AANAT | Protein coding | 24 |
| ENSG00000122733 | 1.92 | 0.010156726 | KIAA1045 | Protein coding | 24 |
| ENSG00000086548 | -1.92 | 5.21E-06 | CEACAM6 | Protein coding | 24 |
| ENSG00000104974 | 1.92 | 9.76E-28 | LILRA1 | Protein coding | 24 |
| ENSG00000154262 | -1.92 | 5.34E-10 | ABCA6 | Protein coding | 24 |
| ENSG00000142611 | 1.91 | 0.001237402 | PRDM16 | Protein coding | 24 |
| ENSG00000157601 | 1.91 | 0.000367506 | MX1 | Protein coding | 24 |
| ENSG00000100079 | -1.91 | 0.00015412 | LGALS2 | Protein coding | 24 |
| ENSG00000183621 | 1.91 | 3.90E-40 | ZNF438 | Protein coding | 24 |
| ENSG00000179165 | 1.91 | 0.002560694 | PXT1 | Protein coding | 24 |
| ENSG00000154258 | -1.91 | 3.57E-07 | ABCA9 | Protein coding | 24 |
| ENSG00000213949 | 1.91 | 1.20E-71 | ITGA1 | Protein coding | 24 |
| ENSG00000167858 | 1.91 | 0.013561806 | TEKT1 | Protein coding | 24 |
| ENSG00000169136 | 1.91 | 1.09E-16 | ATF5 | Protein coding | 24 |
| ENSG00000087085 | 1.91 | 1.48E-09 | ACHE | Protein coding | 24 |
| ENSG00000138642 | 1.91 | 3.81E-08 | HERC6 | Protein coding | 24 |
| ENSG00000056661 | 1.90 | 0.00163333 | PCGF2 | Protein coding | 24 |
| ENSG00000163251 | 1.90 | 3.20E-13 | FZD5 | Protein coding | 24 |
| ENSG00000186049 | -1.90 | 2.82E-26 | KRT73 | Protein coding | 24 |
| ENSG00000104320 | 1.90 | 1.01E-95 | NBN | Protein coding | 24 |
| ENSG00000204001 | -1.90 | 4.54E-06 | LCN8 | Protein coding | 24 |
| ENSG00000089127 | 1.90 | 0.000121788 | OAS1 | Protein coding | 24 |
| ENSG00000145936 | 1.89 | 2.08E-11 | KCNMB1 | Protein coding | 24 |
| ENSG00000171509 | 1.89 | 0.017612448 | RXFP1 | Protein coding | 24 |
| ENSG00000180767 | -1.89 | 0.001529883 | CHST13 | Protein coding | 24 |
| ENSG00000169884 | -1.89 | 1.23E-10 | WNT10B | Protein coding | 24 |
| ENSG00000070729 | 1.89 | 0.010715505 | CNGB1 | Protein coding | 24 |
| ENSG00000217825 | 1.89 | 0.009420562 | AC099552.4 | Protein coding | 24 |
| ENSG00000135404 | 1.89 | 3.18E-22 | CD63 | Protein coding | 24 |
| ENSG00000179148 | -1.89 | 0.015002741 | ALOXE3 | Protein coding | 24 |
| ENSG00000010327 | -1.89 | 4.37E-25 | STAB1 | Protein coding | 24 |
| ENSG00000065328 | 1.89 | 0.001748971 | MCM10 | Protein coding | 24 |
| ENSG00000175471 | 1.88 | 6.38E-49 | MCTP1 | Protein coding | 24 |
| ENSG00000060558 | 1.88 | 1.50E-21 | GNA15 | Protein coding | 24 |
| ENSG00000197121 | 1.88 | 3.67E-40 | PGAP1 | Protein coding | 24 |
| ENSG00000136830 | 1.87 | 5.19E-23 | FAM129B | Protein coding | 24 |
| ENSG00000105472 | -1.87 | 3.67E-07 | CLEC11A | Protein coding | 24 |
| ENSG00000145555 | 1.87 | 9.82E-08 | MYO10 | Protein coding | 24 |
| ENSG00000136286 | 1.87 | 3.65E-32 | MYO1G | Protein coding | 24 |
| ENSG00000185885 | 1.87 | 3.59E-10 | IFITM1 | Protein coding | 24 |
| ENSG00000230062 | 1.87 | 0.009862354 | ANKRD66 | Protein coding | 24 |
| ENSG00000112715 | 1.87 | 2.28E-07 | VEGFA | Protein coding | 24 |
| ENSG00000087074 | 1.87 | 5.86E-35 | PPP1R15A | Protein coding | 24 |
| ENSG00000169116 | -1.86 | 4.86E-15 | PARM1 | Protein coding | 24 |
| ENSG00000122176 | -1.86 | 0.001129045 | FMOD | Protein coding | 24 |
| ENSG00000143162 | 1.86 | 1.65E-26 | CREG1 | Protein coding | 24 |
| ENSG00000074964 | 1.86 | 2.06E-09 | ARHGEF10L | Protein coding | 24 |
| ENSG00000156574 | -1.86 | 0.011524802 | NODAL | Protein coding | 24 |
| ENSG00000155090 | 1.86 | 2.68E-53 | KLF10 | Protein coding | 24 |
| ENSG00000205517 | -1.86 | 0.002955251 | RGL3 | Protein coding | 24 |
| ENSG00000107447 | -1.86 | 0.011590097 | DNTT | Protein coding | 24 |
| ENSG00000143847 | -1.86 | 1.24E-13 | PPFIA4 | Protein coding | 24 |
| ENSG00000160791 | 1.85 | 1.35E-20 | CCR5 | Protein coding | 24 |
| ENSG00000137193 | 1.85 | 2.09E-53 | PIM1 | Protein coding | 24 |
| ENSG00000162631 | -1.85 | 0.005303123 | NTNG1 | Protein coding | 24 |
| ENSG00000121690 | -1.85 | 4.27E-09 | DEPDC7 | Protein coding | 24 |
| ENSG00000160213 | 1.85 | 4.89E-32 | CSTB | Protein coding | 24 |
| ENSG00000076716 | -1.85 | 2.61E-18 | GPC4 | Protein coding | 24 |
| ENSG00000120949 | 1.85 | 2.79E-29 | TNFRSF8 | Protein coding | 24 |
| ENSG00000179399 | 1.85 | 0.001826087 | GPC5 | Protein coding | 24 |
| ENSG00000105609 | 1.85 | 4.18E-08 | LILRB5 | Protein coding | 24 |
| ENSG00000186529 | -1.85 | 4.03E-08 | CYP4F3 | Protein coding | 24 |
| ENSG00000162383 | -1.85 | 9.58E-12 | SLC1A7 | Protein coding | 24 |
| ENSG00000179750 | 1.85 | 4.50E-06 | APOBEC3B | Protein coding | 24 |
| ENSG00000163874 | 1.85 | 1.33E-46 | ZC3H12A | Protein coding | 24 |
| ENSG00000171621 | 1.85 | 1.37E-19 | SPSB1 | Protein coding | 24 |
| ENSG00000135114 | 1.84 | 7.74E-06 | OASL | Protein coding | 24 |
| ENSG00000169495 | 1.84 | 0.000174814 | HTRA4 | Protein coding | 24 |
| ENSG00000203685 | -1.84 | 1.26E-05 | C1orf95 | Protein coding | 24 |
| ENSG00000087245 | 1.84 | 0.000540764 | MMP2 | Protein coding | 24 |
| ENSG00000185291 | 1.84 | 4.51E-20 | IL3RA | Protein coding | 24 |
| ENSG00000166922 | 1.83 | 1.89E-06 | SCG5 | Protein coding | 24 |
| ENSG00000019991 | -1.83 | 2.18E-09 | HGF | Protein coding | 24 |
| ENSG00000102934 | -1.83 | 8.81E-09 | PLLP | Protein coding | 24 |
| ENSG00000166592 | 1.83 | 5.56E-07 | RRAD | Protein coding | 24 |
| ENSG00000173114 | -1.83 | 9.90E-19 | LRRN3 | Protein coding | 24 |
| ENSG00000115267 | 1.83 | 4.89E-19 | IFIH1 | Protein coding | 24 |
| ENSG00000087842 | 1.82 | 8.33E-10 | PIR | Protein coding | 24 |
| ENSG00000174460 | -1.82 | 0.018070154 | ZCCHC12 | Protein coding | 24 |
| ENSG00000166523 | 1.82 | 4.29E-23 | CLEC4E | Protein coding | 24 |
| ENSG00000011201 | 1.82 | 1.01E-05 | KAL1 | Protein coding | 24 |
| ENSG00000115884 | 1.82 | 0.003598033 | SDC1 | Protein coding | 24 |
| ENSG00000173193 | 1.82 | 2.24E-24 | PARP14 | Protein coding | 24 |
| ENSG00000179593 | 1.82 | 1.63E-15 | ALOX15B | Protein coding | 24 |
| ENSG00000173599 | -1.82 | 1.57E-38 | PC | Protein coding | 24 |
| ENSG00000010610 | -1.82 | 1.07E-45 | CD4 | Protein coding | 24 |
| ENSG00000158481 | -1.82 | 3.25E-19 | CD1C | Protein coding | 24 |
| ENSG00000064932 | 1.82 | 1.10E-23 | SBNO2 | Protein coding | 24 |
| ENSG00000203804 | 1.82 | 0.000139598 | C1orf138 | Protein coding | 24 |
| ENSG00000188158 | -1.82 | 1.88E-08 | NHS | Protein coding | 24 |
| ENSG00000142910 | -1.82 | 0.003437473 | TINAGL1 | Protein coding | 24 |
| ENSG00000141469 | -1.82 | 7.71E-18 | SLC14A1 | Protein coding | 24 |
| ENSG00000178719 | 1.82 | 4.27E-25 | GRINA | Protein coding | 24 |
| ENSG00000163221 | 1.81 | 3.05E-15 | S100A12 | Protein coding | 24 |
| ENSG00000180596 | 1.81 | 2.21E-09 | HIST1H2BC | Protein coding | 24 |
| ENSG00000183696 | 1.81 | 5.46E-41 | UPP1 | Protein coding | 24 |
| ENSG00000167483 | -1.81 | 5.61E-54 | FAM129C | Protein coding | 24 |
| ENSG00000141574 | 1.81 | 7.83E-05 | SECTM1 | Protein coding | 24 |
| ENSG00000241399 | -1.80 | 2.05E-33 | CD302 | Protein coding | 24 |
| ENSG00000170801 | 1.80 | 0.001490984 | HTRA3 | Protein coding | 24 |
| ENSG00000173212 | 1.80 | 0.006002503 | MAB21L3 | Protein coding | 24 |
| ENSG00000169194 | 1.80 | 0.000938064 | IL13 | Protein coding | 24 |
| ENSG00000125430 | 1.79 | 2.25E-22 | HS3ST3B1 | Protein coding | 24 |
| ENSG00000154102 | -1.79 | 1.51E-24 | C16orf74 | Protein coding | 24 |
| ENSG00000187513 | 1.79 | 0.006653835 | GJA4 | Protein coding | 24 |
| ENSG00000186187 | 1.79 | 2.07E-32 | ZNRF1 | Protein coding | 24 |
| ENSG00000158315 | 1.79 | 0.007545972 | RHBDL2 | Protein coding | 24 |
| ENSG00000007516 | -1.79 | 8.43E-36 | BAIAP3 | Protein coding | 24 |
| ENSG00000140932 | -1.78 | 8.51E-05 | CMTM2 | Protein coding | 24 |
| ENSG00000267174 | -1.78 | 0.002325925 | CTC-510F12.4 | Protein coding | 24 |
| ENSG00000133101 | 1.78 | 8.62E-08 | CCNA1 | Protein coding | 24 |
| ENSG00000167105 | 1.78 | 0.000823561 | TMEM92 | Protein coding | 24 |
| ENSG00000172232 | -1.78 | 0.001113287 | AZU1 | Protein coding | 24 |
| ENSG00000140284 | 1.78 | 5.98E-08 | SLC27A2 | Protein coding | 24 |
| ENSG00000102393 | 1.78 | 3.15E-27 | GLA | Protein coding | 24 |
| ENSG00000163082 | 1.78 | 1.91E-22 | SGPP2 | Protein coding | 24 |
| ENSG00000136694 | -1.78 | 0.023122544 | IL36A | Protein coding | 24 |
| ENSG00000148737 | 1.78 | 1.22E-20 | TCF7L2 | Protein coding | 24 |
| ENSG00000144597 | 1.78 | 3.29E-42 | EAF1 | Protein coding | 24 |
| ENSG00000165507 | 1.78 | 1.08E-15 | C10orf10 | Protein coding | 24 |
| ENSG00000239998 | 1.78 | 9.08E-15 | LILRA2 | Protein coding | 24 |
| ENSG00000134716 | 1.78 | 1.50E-05 | CYP2J2 | Protein coding | 24 |
| ENSG00000167994 | 1.78 | 0.000361622 | RAB3IL1 | Protein coding | 24 |
| ENSG00000143147 | 1.78 | 2.15E-10 | GPR161 | Protein coding | 24 |
| ENSG00000069122 | 1.77 | 0.021476819 | GPR116 | Protein coding | 24 |
| ENSG00000130222 | 1.77 | 4.36E-05 | GADD45G | Protein coding | 24 |
| ENSG00000144802 | 1.77 | 1.91E-76 | NFKBIZ | Protein coding | 24 |
| ENSG00000120457 | -1.77 | 0.029255073 | KCNJ5 | Protein coding | 24 |
| ENSG00000090530 | -1.76 | 0.006486349 | LEPREL1 | Protein coding | 24 |
| ENSG00000175779 | -1.76 | 0.021951746 | C15orf53 | Protein coding | 24 |
| ENSG00000085514 | 1.76 | 5.22E-28 | PILRA | Protein coding | 24 |
| ENSG00000115718 | -1.76 | 1.16E-05 | PROC | Protein coding | 24 |
| ENSG00000130643 | -1.76 | 0.002325925 | CALY | Protein coding | 24 |
| ENSG00000164647 | 1.76 | 0.007205617 | STEAP1 | Protein coding | 24 |
| ENSG00000082996 | 1.76 | 6.64E-50 | RNF13 | Protein coding | 24 |
| ENSG00000160471 | 1.76 | 0.001761773 | COX6B2 | Protein coding | 24 |
| ENSG00000171236 | 1.76 | 9.82E-11 | LRG1 | Protein coding | 24 |
| ENSG00000203814 | 1.76 | 0.000542518 | HIST2H2BF | Protein coding | 24 |
| ENSG00000196581 | -1.76 | 0.002964498 | AJAP1 | Protein coding | 24 |
| ENSG00000005381 | -1.76 | 6.19E-10 | MPO | Protein coding | 24 |
| ENSG00000117984 | 1.75 | 1.52E-07 | CTSD | Protein coding | 24 |
| ENSG00000167261 | -1.75 | 7.23E-47 | DPEP2 | Protein coding | 24 |
| ENSG00000138646 | 1.75 | 3.10E-05 | HERC5 | Protein coding | 24 |
| ENSG00000039560 | 1.75 | 1.14E-09 | RAI14 | Protein coding | 24 |
| ENSG00000115828 | 1.75 | 1.48E-19 | QPCT | Protein coding | 24 |
| ENSG00000101230 | -1.75 | 1.10E-06 | ISM1 | Protein coding | 24 |
| ENSG00000113361 | -1.75 | 0.028360763 | CDH6 | Protein coding | 24 |
| ENSG00000197982 | 1.75 | 2.80E-23 | C1orf122 | Protein coding | 24 |
| ENSG00000085117 | 1.75 | 7.54E-25 | CD82 | Protein coding | 24 |
| ENSG00000106025 | -1.75 | 0.031727801 | TSPAN12 | Protein coding | 24 |
| ENSG00000075035 | -1.74 | 0.013420851 | WSCD2 | Protein coding | 24 |
| ENSG00000134042 | -1.74 | 0.031107442 | MRO | Protein coding | 24 |
| ENSG00000156587 | 1.74 | 4.60E-09 | UBE2L6 | Protein coding | 24 |
| ENSG00000213937 | -1.74 | 0.004738274 | CLDN9 | Protein coding | 24 |
| ENSG00000100644 | 1.74 | 6.46E-43 | HIF1A | Protein coding | 24 |
| ENSG00000185507 | 1.74 | 2.74E-05 | IRF7 | Protein coding | 24 |
| ENSG00000135077 | 1.74 | 9.18E-23 | HAVCR2 | Protein coding | 24 |
| ENSG00000108179 | 1.74 | 3.98E-25 | PPIF | Protein coding | 24 |
| ENSG00000090382 | -1.73 | 5.16E-27 | LYZ | Protein coding | 24 |
| ENSG00000168903 | -1.73 | 0.025091169 | BTNL3 | Protein coding | 24 |
| ENSG00000062716 | 1.73 | 8.39E-52 | VMP1 | Protein coding | 24 |
| ENSG00000198053 | 1.73 | 1.36E-23 | SIRPA | Protein coding | 24 |
| ENSG00000137267 | 1.73 | 3.10E-09 | TUBB2A | Protein coding | 24 |
| ENSG00000216490 | 1.73 | 8.97E-16 | IFI30 | Protein coding | 24 |
| ENSG00000152778 | 1.73 | 3.48E-18 | IFIT5 | Protein coding | 24 |
| ENSG00000171517 | 1.73 | 0.000323574 | LPAR3 | Protein coding | 24 |
| ENSG00000065911 | 1.73 | 2.19E-33 | MTHFD2 | Protein coding | 24 |
| ENSG00000115956 | 1.72 | 2.99E-24 | PLEK | Protein coding | 24 |
| ENSG00000162692 | 1.72 | 8.60E-08 | VCAM1 | Protein coding | 24 |
| ENSG00000108582 | 1.72 | 1.03E-23 | CPD | Protein coding | 24 |
| ENSG00000182379 | 1.72 | 4.52E-05 | NXPH4 | Protein coding | 24 |
| ENSG00000101425 | -1.72 | 6.82E-15 | BPI | Protein coding | 24 |
| ENSG00000168754 | -1.72 | 0.000696 | FAM178B | Protein coding | 24 |
| ENSG00000170099 | 1.72 | 0.033023518 | SERPINA6 | Protein coding | 24 |
| ENSG00000168386 | -1.72 | 2.21E-10 | FILIP1L | Protein coding | 24 |
| ENSG00000136040 | 1.72 | 3.33E-32 | PLXNC1 | Protein coding | 24 |
| ENSG00000160200 | 1.72 | 1.34E-07 | CBS | Protein coding | 24 |
| ENSG00000158186 | 1.72 | 1.19E-21 | MRAS | Protein coding | 24 |
| ENSG00000127507 | 1.72 | 4.51E-34 | EMR2 | Protein coding | 24 |
| ENSG00000115159 | 1.72 | 7.51E-45 | GPD2 | Protein coding | 24 |
| ENSG00000133067 | -1.71 | 5.39E-20 | LGR6 | Protein coding | 24 |
| ENSG00000169442 | -1.71 | 1.03E-64 | CD52 | Protein coding | 24 |
| ENSG00000186047 | 1.71 | 7.61E-09 | DLEU7 | Protein coding | 24 |
| ENSG00000074181 | 1.71 | 3.17E-07 | NOTCH3 | Protein coding | 24 |
| ENSG00000141748 | -1.70 | 0.020248391 | ARL5C | Protein coding | 24 |
| ENSG00000168394 | 1.70 | 2.55E-19 | TAP1 | Protein coding | 24 |
| ENSG00000126709 | 1.70 | 0.005146971 | IFI6 | Protein coding | 24 |
| ENSG00000170439 | 1.70 | 6.38E-05 | METTL7B | Protein coding | 24 |
| ENSG00000176597 | 1.70 | 3.00E-24 | B3GNT5 | Protein coding | 24 |
| ENSG00000198910 | 1.70 | 3.22E-19 | L1CAM | Protein coding | 24 |
| ENSG00000136231 | 1.70 | 0.000205214 | IGF2BP3 | Protein coding | 24 |
| ENSG00000163746 | 1.70 | 0.000422717 | PLSCR2 | Protein coding | 24 |
| ENSG00000214193 | 1.70 | 4.71E-14 | SH3D21 | Protein coding | 24 |
| ENSG00000186407 | 1.70 | 7.33E-24 | CD300E | Protein coding | 24 |
| ENSG00000196542 | -1.69 | 0.007812256 | SPTSSB | Protein coding | 24 |
| ENSG00000156510 | -1.69 | 4.54E-06 | HKDC1 | Protein coding | 24 |
| ENSG00000147416 | 1.69 | 1.70E-25 | ATP6V1B2 | Protein coding | 24 |
| ENSG00000130962 | 1.69 | 0.000265984 | PRRG1 | Protein coding | 24 |
| ENSG00000152315 | 1.69 | 3.32E-07 | KCNK13 | Protein coding | 24 |
| ENSG00000187479 | 1.69 | 0.03082898 | C11orf96 | Protein coding | 24 |
| ENSG00000163393 | 1.69 | 6.95E-22 | SLC22A15 | Protein coding | 24 |
| ENSG00000173281 | 1.69 | 1.09E-48 | PPP1R3B | Protein coding | 24 |
| ENSG00000174469 | -1.69 | 3.35E-12 | CNTNAP2 | Protein coding | 24 |
| ENSG00000120162 | 1.69 | 1.94E-15 | MOB3B | Protein coding | 24 |
| ENSG00000177807 | 1.69 | 1.50E-05 | KCNJ10 | Protein coding | 24 |
| ENSG00000169122 | 1.68 | 1.46E-06 | FAM110B | Protein coding | 24 |
| ENSG00000142549 | 1.68 | 0.003266832 | IGLON5 | Protein coding | 24 |
| ENSG00000185947 | 1.68 | 1.72E-37 | ZNF267 | Protein coding | 24 |
| ENSG00000170385 | 1.68 | 1.14E-60 | SLC30A1 | Protein coding | 24 |
| ENSG00000156011 | 1.68 | 3.34E-33 | PSD3 | Protein coding | 24 |
| ENSG00000198768 | 1.68 | 0.033218907 | APCDD1L | Protein coding | 24 |
| ENSG00000153823 | 1.68 | 1.68E-26 | PID1 | Protein coding | 24 |
| ENSG00000010278 | -1.68 | 3.07E-27 | CD9 | Protein coding | 24 |
| ENSG00000163958 | 1.68 | 0.001389906 | ZDHHC19 | Protein coding | 24 |
| ENSG00000164604 | 1.68 | 0.00787023 | GPR85 | Protein coding | 24 |
| ENSG00000183722 | 1.67 | 0.000604321 | LHFP | Protein coding | 24 |
| ENSG00000110665 | -1.67 | 1.32E-55 | C11orf21 | Protein coding | 24 |
| ENSG00000141837 | 1.67 | 5.93E-10 | CACNA1A | Protein coding | 24 |
| ENSG00000026103 | 1.67 | 2.39E-25 | FAS | Protein coding | 24 |
| ENSG00000167995 | 1.67 | 1.69E-21 | BEST1 | Protein coding | 24 |
| ENSG00000188060 | 1.67 | 1.89E-06 | RAB42 | Protein coding | 24 |
| ENSG00000167987 | 1.66 | 6.63E-19 | VPS37C | Protein coding | 24 |
| ENSG00000165383 | 1.66 | 0.040217466 | LRRC18 | Protein coding | 24 |
| ENSG00000107317 | -1.66 | 7.18E-08 | PTGDS | Protein coding | 24 |
| ENSG00000185339 | 1.66 | 0.001341754 | TCN2 | Protein coding | 24 |
| ENSG00000145362 | 1.66 | 0.000756807 | ANK2 | Protein coding | 24 |
| ENSG00000182326 | 1.66 | 7.32E-06 | C1S | Protein coding | 24 |
| ENSG00000050767 | -1.66 | 0.000450096 | COL23A1 | Protein coding | 24 |
| ENSG00000168209 | 1.66 | 1.93E-17 | DDIT4 | Protein coding | 24 |
| ENSG00000145365 | 1.66 | 7.75E-29 | TIFA | Protein coding | 24 |
| ENSG00000174370 | -1.65 | 1.61E-07 | C11orf45 | Protein coding | 24 |
| ENSG00000205846 | 1.65 | 1.14E-10 | CLEC6A | Protein coding | 24 |
| ENSG00000158062 | -1.65 | 1.71E-60 | UBXN11 | Protein coding | 24 |
| ENSG00000242498 | -1.65 | 5.39E-16 | C15orf38 | Protein coding | 24 |
| ENSG00000141096 | -1.65 | 1.76E-13 | DPEP3 | Protein coding | 24 |
| ENSG00000050438 | 1.65 | 5.06E-28 | SLC4A8 | Protein coding | 24 |
| ENSG00000169855 | 1.65 | 1.67E-09 | ROBO1 | Protein coding | 24 |
| ENSG00000104375 | 1.65 | 6.29E-34 | STK3 | Protein coding | 24 |
| ENSG00000196154 | -1.65 | 2.50E-34 | S100A4 | Protein coding | 24 |
| ENSG00000050628 | 1.65 | 0.000490895 | PTGER3 | Protein coding | 24 |
| ENSG00000110446 | 1.65 | 3.52E-16 | SLC15A3 | Protein coding | 24 |
| ENSG00000076351 | -1.65 | 1.33E-11 | SLC46A1 | Protein coding | 24 |
| ENSG00000171798 | 1.64 | 0.002283104 | KNDC1 | Protein coding | 24 |
| ENSG00000167434 | -1.64 | 0.040799346 | CA4 | Protein coding | 24 |
| ENSG00000114019 | 1.64 | 0.037455013 | AMOTL2 | Protein coding | 24 |
| ENSG00000148834 | 1.64 | 1.40E-24 | GSTO1 | Protein coding | 24 |
| ENSG00000196754 | 1.64 | 0.01112021 | S100A2 | Protein coding | 24 |
| ENSG00000124469 | -1.64 | 1.89E-09 | CEACAM8 | Protein coding | 24 |
| ENSG00000198853 | 1.64 | 5.98E-21 | RUSC2 | Protein coding | 24 |
| ENSG00000114698 | 1.64 | 0.026464321 | PLSCR4 | Protein coding | 24 |
| ENSG00000123094 | 1.64 | 3.93E-07 | RASSF8 | Protein coding | 24 |
| ENSG00000185046 | -1.64 | 0.015144441 | ANKS1B | Protein coding | 24 |
| ENSG00000103942 | -1.64 | 1.19E-19 | HOMER2 | Protein coding | 24 |
| ENSG00000079385 | 1.64 | 4.64E-09 | CEACAM1 | Protein coding | 24 |
| ENSG00000171051 | 1.64 | 3.86E-31 | FPR1 | Protein coding | 24 |
| ENSG00000101327 | 1.64 | 0.04402052 | PDYN | Protein coding | 24 |
| ENSG00000198673 | 1.64 | 9.70E-12 | FAM19A2 | Protein coding | 24 |
| ENSG00000172380 | 1.63 | 0.027299013 | GNG12 | Protein coding | 24 |
| ENSG00000090104 | 1.63 | 1.14E-40 | RGS1 | Protein coding | 24 |
| ENSG00000049768 | 1.63 | 5.57E-21 | FOXP3 | Protein coding | 24 |
| ENSG00000165695 | 1.63 | 0.000331906 | AK8 | Protein coding | 24 |
| ENSG00000179348 | 1.63 | 3.22E-12 | GATA2 | Protein coding | 24 |
| ENSG00000118849 | 1.63 | 0.017952891 | RARRES1 | Protein coding | 24 |
| ENSG00000087086 | 1.63 | 5.29E-17 | FTL | Protein coding | 24 |
| ENSG00000110218 | 1.63 | 4.34E-43 | PANX1 | Protein coding | 24 |
| ENSG00000125744 | 1.63 | 2.68E-12 | RTN2 | Protein coding | 24 |
| ENSG00000100678 | 1.63 | 0.004008538 | SLC8A3 | Protein coding | 24 |
| ENSG00000166670 | 1.63 | 0.038893948 | MMP10 | Protein coding | 24 |
| ENSG00000181585 | -1.63 | 6.43E-05 | TMIE | Protein coding | 24 |
| ENSG00000255582 | -1.62 | 0.008676255 | OR10G2 | Protein coding | 24 |
| ENSG00000196189 | 1.62 | 3.73E-06 | SEMA4A | Protein coding | 24 |
| ENSG00000009694 | -1.62 | 3.49E-16 | TENM1 | Protein coding | 24 |
| ENSG00000196355 | -1.62 | 1.50E-18 | AC021860.1 | Protein coding | 24 |
| ENSG00000111335 | 1.62 | 6.71E-06 | OAS2 | Protein coding | 24 |
| ENSG00000167094 | -1.62 | 7.84E-18 | TTC16 | Protein coding | 24 |
| ENSG00000119686 | 1.62 | 1.09E-10 | FLVCR2 | Protein coding | 24 |
| ENSG00000072682 | 1.62 | 4.03E-06 | P4HA2 | Protein coding | 24 |
| ENSG00000156453 | -1.61 | 1.27E-09 | PCDH1 | Protein coding | 24 |
| ENSG00000114948 | -1.61 | 5.44E-16 | ADAM23 | Protein coding | 24 |
| ENSG00000227051 | -1.61 | 1.14E-24 | C14orf132 | Protein coding | 24 |
| ENSG00000111863 | 1.61 | 8.10E-37 | ADTRP | Protein coding | 24 |
| ENSG00000167553 | 1.61 | 1.72E-12 | TUBA1C | Protein coding | 24 |
| ENSG00000120262 | 1.61 | 3.70E-08 | CCDC170 | Protein coding | 24 |
| ENSG00000143412 | -1.61 | 1.66E-18 | ANXA9 | Protein coding | 24 |
| ENSG00000138760 | 1.61 | 1.32E-37 | SCARB2 | Protein coding | 24 |
| ENSG00000196622 | -1.61 | 0.001063106 | RIMBP3 | Protein coding | 24 |
| ENSG00000140511 | 1.61 | 4.11E-17 | HAPLN3 | Protein coding | 24 |
| ENSG00000171595 | -1.61 | 2.64E-05 | DNAI2 | Protein coding | 24 |
| ENSG00000120051 | 1.61 | 0.000153049 | CCDC147 | Protein coding | 24 |
| ENSG00000091129 | -1.60 | 0.000663771 | NRCAM | Protein coding | 24 |
| ENSG00000236320 | -1.60 | 8.22E-13 | SLFN14 | Protein coding | 24 |
| ENSG00000197561 | -1.60 | 0.026658129 | ELANE | Protein coding | 24 |
| ENSG00000121297 | 1.60 | 2.37E-13 | TSHZ3 | Protein coding | 24 |
| ENSG00000055332 | 1.60 | 7.41E-08 | EIF2AK2 | Protein coding | 24 |
| ENSG00000135148 | 1.60 | 5.47E-18 | TRAFD1 | Protein coding | 24 |
| ENSG00000145901 | 1.60 | 2.44E-30 | TNIP1 | Protein coding | 24 |
| ENSG00000144843 | 1.60 | 2.45E-24 | ADPRH | Protein coding | 24 |
| ENSG00000139354 | 1.60 | 3.31E-17 | GAS2L3 | Protein coding | 24 |
| ENSG00000244486 | -1.60 | 6.14E-06 | SCARF2 | Protein coding | 24 |
| ENSG00000196396 | 1.60 | 6.46E-63 | PTPN1 | Protein coding | 24 |
| ENSG00000136630 | 1.60 | 2.38E-13 | HLX | Protein coding | 24 |
| ENSG00000107518 | -1.60 | 0.044261708 | ATRNL1 | Protein coding | 24 |
| ENSG00000138411 | 1.59 | 6.63E-05 | HECW2 | Protein coding | 24 |
| ENSG00000139410 | 1.59 | 0.00029944 | SDSL | Protein coding | 24 |
| ENSG00000118985 | 1.59 | 2.90E-38 | ELL2 | Protein coding | 24 |
| ENSG00000101160 | 1.59 | 5.93E-16 | CTSZ | Protein coding | 24 |
| ENSG00000005108 | -1.59 | 0.008839864 | THSD7A | Protein coding | 24 |
| ENSG00000187583 | 1.59 | 3.36E-12 | PLEKHN1 | Protein coding | 24 |
| ENSG00000158104 | 1.59 | 0.002112284 | HPD | Protein coding | 24 |
| ENSG00000198515 | -1.58 | 0.027400262 | CNGA1 | Protein coding | 24 |
| ENSG00000198785 | 1.58 | 6.78E-06 | GRIN3A | Protein coding | 24 |
| ENSG00000242616 | 1.58 | 2.21E-07 | GNG10 | Protein coding | 24 |
| ENSG00000031081 | 1.58 | 1.14E-18 | ARHGAP31 | Protein coding | 24 |
| ENSG00000080854 | -1.58 | 4.93E-16 | IGSF9B | Protein coding | 24 |
| ENSG00000167895 | -1.58 | 1.02E-43 | TMC8 | Protein coding | 24 |
| ENSG00000197705 | -1.58 | 3.91E-20 | KLHL14 | Protein coding | 24 |
| ENSG00000100097 | 1.58 | 5.38E-15 | LGALS1 | Protein coding | 24 |
| ENSG00000176641 | 1.58 | 0.006098646 | RNF152 | Protein coding | 24 |
| ENSG00000130487 | 1.58 | 2.33E-10 | KLHDC7B | Protein coding | 24 |
| ENSG00000103257 | 1.58 | 1.02E-27 | SLC7A5 | Protein coding | 24 |
| ENSG00000197461 | 1.58 | 4.56E-07 | PDGFA | Protein coding | 24 |
| ENSG00000185432 | -1.58 | 9.16E-27 | METTL7A | Protein coding | 24 |
| ENSG00000178226 | -1.58 | 1.48E-09 | PRSS36 | Protein coding | 24 |
| ENSG00000073737 | -1.58 | 1.09E-06 | DHRS9 | Protein coding | 24 |
| ENSG00000164713 | 1.57 | 1.08E-21 | BRI3 | Protein coding | 24 |
| ENSG00000168461 | 1.57 | 3.48E-30 | RAB31 | Protein coding | 24 |
| ENSG00000161640 | 1.57 | 0.002051548 | SIGLEC11 | Protein coding | 24 |
| ENSG00000053108 | -1.57 | 4.32E-05 | FSTL4 | Protein coding | 24 |
| ENSG00000184060 | 1.57 | 6.28E-16 | ADAP2 | Protein coding | 24 |
| ENSG00000170956 | 1.57 | 8.25E-10 | CEACAM3 | Protein coding | 24 |
| ENSG00000115592 | 1.57 | 0.000740449 | PRKAG3 | Protein coding | 24 |
| ENSG00000179604 | 1.57 | 5.03E-08 | CDC42EP4 | Protein coding | 24 |
| ENSG00000102265 | 1.57 | 1.82E-15 | TIMP1 | Protein coding | 24 |
| ENSG00000166546 | -1.57 | 0.013300963 | BEAN1 | Protein coding | 24 |
| ENSG00000103642 | 1.57 | 1.82E-32 | LACTB | Protein coding | 24 |
| ENSG00000162645 | 1.56 | 1.37E-23 | GBP2 | Protein coding | 24 |
| ENSG00000110076 | 1.56 | 8.80E-08 | NRXN2 | Protein coding | 24 |
| ENSG00000102580 | 1.56 | 4.57E-30 | DNAJC3 | Protein coding | 24 |
| ENSG00000168389 | 1.56 | 1.54E-24 | MFSD2A | Protein coding | 24 |
| ENSG00000159374 | -1.56 | 0.008727889 | M1AP | Protein coding | 24 |
| ENSG00000139629 | 1.56 | 3.63E-23 | GALNT6 | Protein coding | 24 |
| ENSG00000196132 | -1.56 | 0.001618843 | MYT1 | Protein coding | 24 |
| ENSG00000118160 | 1.56 | 0.032157395 | SLC8A2 | Protein coding | 24 |
| ENSG00000196562 | -1.56 | 1.04E-14 | SULF2 | Protein coding | 24 |
| ENSG00000069188 | -1.56 | 6.02E-13 | SDK2 | Protein coding | 24 |
| ENSG00000164512 | -1.56 | 8.43E-09 | ANKRD55 | Protein coding | 24 |
| ENSG00000183742 | 1.56 | 8.27E-11 | MACC1 | Protein coding | 24 |
| ENSG00000148175 | 1.55 | 1.25E-29 | STOM | Protein coding | 24 |
| ENSG00000197262 | 1.55 | 0.004150659 | CCL4L2 | Protein coding | 24 |
| ENSG00000117215 | -1.55 | 0.048369792 | PLA2G2D | Protein coding | 24 |
| ENSG00000071967 | -1.55 | 2.01E-23 | CYBRD1 | Protein coding | 24 |
| ENSG00000153976 | 1.55 | 0.009814789 | HS3ST3A1 | Protein coding | 24 |
| ENSG00000214402 | -1.55 | 0.01775015 | LCNL1 | Protein coding | 24 |
| ENSG00000157470 | -1.55 | 0.003035031 | FAM81A | Protein coding | 24 |
| ENSG00000104783 | 1.55 | 7.06E-32 | KCNN4 | Protein coding | 24 |
| ENSG00000244242 | 1.55 | 2.25E-07 | IFITM10 | Protein coding | 24 |
| ENSG00000166510 | -1.55 | 3.25E-07 | CCDC68 | Protein coding | 24 |
| ENSG00000140939 | 1.55 | 6.52E-12 | NOL3 | Protein coding | 24 |
| ENSG00000128335 | 1.54 | 2.13E-26 | APOL2 | Protein coding | 24 |
| ENSG00000181649 | 1.54 | 0.000965445 | PHLDA2 | Protein coding | 24 |
| ENSG00000117226 | 1.54 | 7.59E-33 | GBP3 | Protein coding | 24 |
| ENSG00000255346 | -1.54 | 0.047655116 | NOX5 | Protein coding | 24 |
| ENSG00000171435 | -1.54 | 0.030735406 | KSR2 | Protein coding | 24 |
| ENSG00000132109 | 1.54 | 1.45E-17 | TRIM21 | Protein coding | 24 |
| ENSG00000172403 | 1.54 | 1.09E-13 | SYNPO2 | Protein coding | 24 |
| ENSG00000160145 | -1.54 | 3.81E-08 | KALRN | Protein coding | 24 |
| ENSG00000118515 | 1.54 | 1.48E-28 | SGK1 | Protein coding | 24 |
| ENSG00000227184 | -1.53 | 1.60E-15 | EPPK1 | Protein coding | 24 |
| ENSG00000140564 | 1.53 | 7.39E-25 | FURIN | Protein coding | 24 |
| ENSG00000163354 | -1.53 | 4.11E-06 | DCST2 | Protein coding | 24 |
| ENSG00000148459 | 1.53 | 1.63E-14 | PDSS1 | Protein coding | 24 |
| ENSG00000166016 | 1.53 | 1.62E-17 | ABTB2 | Protein coding | 24 |
| ENSG00000087237 | 1.53 | 2.94E-10 | CETP | Protein coding | 24 |
| ENSG00000081985 | 1.53 | 4.72E-30 | IL12RB2 | Protein coding | 24 |
| ENSG00000141655 | -1.53 | 4.64E-17 | TNFRSF11A | Protein coding | 24 |
| ENSG00000169439 | 1.53 | 2.70E-23 | SDC2 | Protein coding | 24 |
| ENSG00000174326 | -1.53 | 1.22E-05 | SLC16A11 | Protein coding | 24 |
| ENSG00000172070 | 1.53 | 3.15E-17 | SRXN1 | Protein coding | 24 |
| ENSG00000110799 | -1.53 | 9.62E-05 | VWF | Protein coding | 24 |
| ENSG00000109756 | 1.53 | 1.34E-60 | RAPGEF2 | Protein coding | 24 |
| ENSG00000099860 | 1.52 | 1.90E-21 | GADD45B | Protein coding | 24 |
| ENSG00000114812 | -1.52 | 5.76E-44 | VIPR1 | Protein coding | 24 |
| ENSG00000124743 | -1.52 | 0.008970889 | KLHL31 | Protein coding | 24 |
| ENSG00000110203 | -1.52 | 5.15E-06 | FOLR3 | Protein coding | 24 |
| ENSG00000123130 | 1.52 | 1.61E-25 | ACOT9 | Protein coding | 24 |
| ENSG00000111261 | -1.52 | 0.008141544 | MANSC1 | Protein coding | 24 |
| ENSG00000182022 | 1.52 | 1.08E-28 | CHST15 | Protein coding | 24 |
| ENSG00000070614 | 1.52 | 9.38E-21 | NDST1 | Protein coding | 24 |
| ENSG00000086288 | -1.52 | 1.40E-10 | NME8 | Protein coding | 24 |
| ENSG00000188157 | 1.52 | 2.59E-05 | AGRN | Protein coding | 24 |
| ENSG00000166927 | 1.52 | 1.74E-23 | MS4A7 | Protein coding | 24 |
| ENSG00000132386 | -1.52 | 1.55E-23 | SERPINF1 | Protein coding | 24 |
| ENSG00000167711 | -1.52 | 1.02E-09 | SERPINF2 | Protein coding | 24 |
| ENSG00000089012 | -1.52 | 2.71E-21 | SIRPG | Protein coding | 24 |
| ENSG00000164251 | 1.52 | 1.85E-13 | F2RL1 | Protein coding | 24 |
| ENSG00000186522 | 1.52 | 3.72E-18 | Sep-10 | Protein coding | 24 |
| ENSG00000129667 | 1.52 | 2.04E-17 | RHBDF2 | Protein coding | 24 |
| ENSG00000197723 | 1.51 | 0.027236878 | HSPB9 | Protein coding | 24 |
| ENSG00000113368 | 1.51 | 4.55E-21 | LMNB1 | Protein coding | 24 |
| ENSG00000170581 | 1.51 | 1.42E-14 | STAT2 | Protein coding | 24 |
| ENSG00000126003 | 1.51 | 5.51E-51 | PLAGL2 | Protein coding | 24 |
| ENSG00000107201 | 1.51 | 1.94E-16 | DDX58 | Protein coding | 24 |
| ENSG00000196167 | -1.51 | 0.008663081 | C11orf92 | Protein coding | 24 |
| ENSG00000092051 | 1.51 | 0.04393524 | JPH4 | Protein coding | 24 |
| ENSG00000151364 | 1.51 | 0.034643862 | KCTD14 | Protein coding | 24 |
| ENSG00000132274 | 1.51 | 1.77E-15 | TRIM22 | Protein coding | 24 |
| ENSG00000186204 | -1.51 | 0.01338553 | CYP4F12 | Protein coding | 24 |
| ENSG00000156273 | 1.51 | 1.58E-57 | BACH1 | Protein coding | 24 |
| ENSG00000140470 | 1.50 | 6.41E-08 | ADAMTS17 | Protein coding | 24 |
| ENSG00000169604 | -1.50 | 0.018820039 | ANTXR1 | Protein coding | 24 |
| ENSG00000179294 | 1.50 | 2.28E-09 | C17orf96 | Protein coding | 24 |
| ENSG00000119714 | 1.50 | 5.91E-14 | GPR68 | Protein coding | 24 |
| ENSG00000108679 | 1.50 | 1.05E-06 | LGALS3BP | Protein coding | 24 |
| ENSG00000110880 | 1.50 | 1.10E-23 | CORO1C | Protein coding | 24 |
| ENSG00000196743 | 1.50 | 2.47E-18 | GM2A | Protein coding | 24 |
| ENSG00000183486 | 1.49 | 0.000114538 | MX2 | Protein coding | 24 |
| ENSG00000007314 | -1.49 | 0.000421234 | SCN4A | Protein coding | 24 |
| ENSG00000177398 | -1.49 | 0.015579966 | UMODL1 | Protein coding | 24 |
| ENSG00000136868 | 1.49 | 1.14E-23 | SLC31A1 | Protein coding | 24 |
| ENSG00000184985 | 1.49 | 0.001447254 | SORCS2 | Protein coding | 24 |
| ENSG00000169435 | -1.49 | 1.67E-07 | RASSF6 | Protein coding | 24 |
| ENSG00000184838 | 1.49 | 1.06E-05 | PRR16 | Protein coding | 24 |
| ENSG00000114923 | -1.49 | 0.032070436 | SLC4A3 | Protein coding | 24 |
| ENSG00000156127 | 1.49 | 4.13E-15 | BATF | Protein coding | 24 |
| ENSG00000025708 | 1.48 | 0.000203007 | TYMP | Protein coding | 24 |
| ENSG00000197712 | 1.48 | 5.59E-10 | FAM114A1 | Protein coding | 24 |
| ENSG00000241978 | 1.48 | 0.000603342 | AKAP2 | Protein coding | 24 |
| ENSG00000187775 | 1.48 | 0.000153307 | DNAH17 | Protein coding | 24 |
| ENSG00000165272 | -1.48 | 9.49E-63 | AQP3 | Protein coding | 24 |
| ENSG00000130881 | -1.48 | 0.000360158 | LRP3 | Protein coding | 24 |
| ENSG00000198467 | -1.48 | 4.64E-54 | TPM2 | Protein coding | 24 |
| ENSG00000179057 | -1.48 | 0.000517099 | IGSF22 | Protein coding | 24 |
| ENSG00000213759 | -1.48 | 0.016034093 | UGT2B11 | Protein coding | 24 |
| ENSG00000121316 | -1.48 | 1.99E-11 | PLBD1 | Protein coding | 24 |
| ENSG00000165125 | -1.48 | 0.003243739 | TRPV6 | Protein coding | 24 |
| ENSG00000100600 | 1.48 | 4.98E-10 | LGMN | Protein coding | 24 |
| ENSG00000143322 | 1.47 | 4.13E-55 | ABL2 | Protein coding | 24 |
| ENSG00000170909 | 1.47 | 2.96E-06 | OSCAR | Protein coding | 24 |
| ENSG00000154269 | 1.47 | 0.000138304 | ENPP3 | Protein coding | 24 |
| ENSG00000070985 | -1.47 | 0.027863146 | TRPM5 | Protein coding | 24 |
| ENSG00000129450 | 1.47 | 2.33E-35 | SIGLEC9 | Protein coding | 24 |
| ENSG00000188888 | -1.47 | 0.026610871 | GPR179 | Protein coding | 24 |
| ENSG00000120057 | -1.47 | 0.002652137 | SFRP5 | Protein coding | 24 |
| ENSG00000177453 | 1.47 | 0.000522348 | NIM1 | Protein coding | 24 |
| ENSG00000204978 | -1.47 | 0.038356832 | C19orf69 | Protein coding | 24 |
| ENSG00000142621 | 1.47 | 1.36E-06 | FHAD1 | Protein coding | 24 |
| ENSG00000100346 | -1.47 | 1.37E-46 | CACNA1I | Protein coding | 24 |
| ENSG00000160233 | 1.47 | 4.24E-08 | LRRC3 | Protein coding | 24 |
| ENSG00000072401 | 1.47 | 8.15E-27 | UBE2D1 | Protein coding | 24 |
| ENSG00000102921 | 1.47 | 3.60E-43 | N4BP1 | Protein coding | 24 |
| ENSG00000163191 | 1.46 | 6.59E-18 | S100A11 | Protein coding | 24 |
| ENSG00000161509 | -1.46 | 0.000230283 | GRIN2C | Protein coding | 24 |
| ENSG00000073331 | 1.46 | 8.06E-21 | ALPK1 | Protein coding | 24 |
| ENSG00000153317 | 1.46 | 7.32E-42 | ASAP1 | Protein coding | 24 |
| ENSG00000166963 | 1.46 | 3.39E-09 | MAP1A | Protein coding | 24 |
| ENSG00000117115 | -1.46 | 2.37E-11 | PADI2 | Protein coding | 24 |
| ENSG00000135929 | 1.46 | 5.47E-13 | CYP27A1 | Protein coding | 24 |
| ENSG00000164823 | 1.46 | 1.16E-27 | OSGIN2 | Protein coding | 24 |
| ENSG00000165181 | 1.46 | 0.012893289 | C9orf84 | Protein coding | 24 |
| ENSG00000198715 | 1.46 | 4.52E-13 | C1orf85 | Protein coding | 24 |
| ENSG00000171094 | 1.46 | 0.008444543 | ALK | Protein coding | 24 |
| ENSG00000162723 | 1.46 | 0.000456112 | SLAMF9 | Protein coding | 24 |
| ENSG00000179241 | 1.45 | 2.51E-13 | LDLRAD3 | Protein coding | 24 |
| ENSG00000064886 | 1.45 | 5.61E-15 | CHI3L2 | Protein coding | 24 |
| ENSG00000254918 | -1.45 | 1.72E-15 | RP11-259P6.1 | Protein coding | 24 |
| ENSG00000131370 | 1.45 | 8.49E-19 | SH3BP5 | Protein coding | 24 |
| ENSG00000073150 | 1.45 | 1.11E-07 | PANX2 | Protein coding | 24 |
| ENSG00000185386 | 1.45 | 2.13E-14 | MAPK11 | Protein coding | 24 |
| ENSG00000168003 | 1.45 | 1.95E-18 | SLC3A2 | Protein coding | 24 |
| ENSG00000146021 | -1.45 | 5.54E-19 | KLHL3 | Protein coding | 24 |
| ENSG00000142657 | 1.45 | 3.82E-17 | PGD | Protein coding | 24 |
| ENSG00000047249 | 1.44 | 1.97E-24 | ATP6V1H | Protein coding | 24 |
| ENSG00000157551 | 1.44 | 1.29E-17 | KCNJ15 | Protein coding | 24 |
| ENSG00000154764 | -1.44 | 1.81E-16 | WNT7A | Protein coding | 24 |
| ENSG00000171631 | 1.44 | 0.000321038 | P2RY6 | Protein coding | 24 |
| ENSG00000151948 | 1.44 | 7.24E-13 | GLT1D1 | Protein coding | 24 |
| ENSG00000101017 | 1.44 | 1.03E-17 | CD40 | Protein coding | 24 |
| ENSG00000169504 | 1.44 | 1.13E-31 | CLIC4 | Protein coding | 24 |
| ENSG00000089351 | 1.44 | 2.42E-20 | GRAMD1A | Protein coding | 24 |
| ENSG00000133055 | 1.44 | 4.03E-06 | MYBPH | Protein coding | 24 |
| ENSG00000162398 | -1.43 | 0.027689904 | C1orf177 | Protein coding | 24 |
| ENSG00000177628 | 1.43 | 5.01E-18 | GBA | Protein coding | 24 |
| ENSG00000170667 | -1.43 | 2.04E-05 | RASA4B | Protein coding | 24 |
| ENSG00000124785 | 1.43 | 0.004941584 | NRN1 | Protein coding | 24 |
| ENSG00000172548 | 1.43 | 0.002532922 | NIPAL4 | Protein coding | 24 |
| ENSG00000212734 | -1.43 | 0.021783736 | C17orf100 | Protein coding | 24 |
| ENSG00000066697 | 1.43 | 7.65E-20 | MSANTD3 | Protein coding | 24 |
| ENSG00000196116 | 1.43 | 3.40E-16 | TDRD7 | Protein coding | 24 |
| ENSG00000103647 | -1.43 | 0.001052796 | CORO2B | Protein coding | 24 |
| ENSG00000088827 | 1.43 | 0.036234003 | SIGLEC1 | Protein coding | 24 |
| ENSG00000197081 | 1.42 | 1.60E-23 | IGF2R | Protein coding | 24 |
| ENSG00000143226 | 1.42 | 3.69E-24 | FCGR2A | Protein coding | 24 |
| ENSG00000183856 | -1.42 | 0.000295606 | IQGAP3 | Protein coding | 24 |
| ENSG00000114450 | 1.42 | 9.24E-29 | GNB4 | Protein coding | 24 |
| ENSG00000164136 | 1.42 | 1.68E-17 | IL15 | Protein coding | 24 |
| ENSG00000143153 | 1.42 | 1.50E-06 | ATP1B1 | Protein coding | 24 |
| ENSG00000089250 | -1.42 | 0.044338904 | NOS1 | Protein coding | 24 |
| ENSG00000130948 | -1.42 | 0.007166724 | HSD17B3 | Protein coding | 24 |
| ENSG00000163568 | 1.42 | 1.17E-18 | AIM2 | Protein coding | 24 |
| ENSG00000259030 | -1.42 | 0.002222931 | FPGT-TNNI3K | Protein coding | 24 |
| ENSG00000069399 | 1.42 | 3.32E-18 | BCL3 | Protein coding | 24 |
| ENSG00000104763 | 1.42 | 1.16E-20 | ASAH1 | Protein coding | 24 |
| ENSG00000138600 | 1.42 | 1.82E-53 | SPPL2A | Protein coding | 24 |
| ENSG00000183473 | 1.42 | 8.23E-14 | SSTR3 | Protein coding | 24 |
| ENSG00000085871 | -1.42 | 0.000109586 | MGST2 | Protein coding | 24 |
| ENSG00000114127 | 1.42 | 2.63E-57 | XRN1 | Protein coding | 24 |
| ENSG00000167281 | -1.41 | 0.004782691 | RBFOX3 | Protein coding | 24 |
| ENSG00000144118 | 1.41 | 1.05E-25 | RALB | Protein coding | 24 |
| ENSG00000161647 | 1.41 | 0.001645688 | MPP3 | Protein coding | 24 |
| ENSG00000151704 | -1.41 | 0.011602573 | KCNJ1 | Protein coding | 24 |
| ENSG00000187642 | 1.41 | 0.000269511 | C1orf170 | Protein coding | 24 |
| ENSG00000133805 | 1.41 | 1.50E-22 | AMPD3 | Protein coding | 24 |
| ENSG00000136869 | 1.41 | 4.97E-24 | TLR4 | Protein coding | 24 |
| ENSG00000143434 | -1.41 | 5.13E-15 | SEMA6C | Protein coding | 24 |
| ENSG00000170542 | 1.41 | 1.93E-33 | SERPINB9 | Protein coding | 24 |
| ENSG00000152128 | 1.41 | 0.000149789 | TMEM163 | Protein coding | 24 |
| ENSG00000145506 | 1.41 | 0.038412147 | NKD2 | Protein coding | 24 |
| ENSG00000196730 | 1.41 | 3.08E-13 | DAPK1 | Protein coding | 24 |
| ENSG00000026508 | 1.40 | 1.95E-33 | CD44 | Protein coding | 24 |
| ENSG00000173221 | 1.40 | 4.34E-28 | GLRX | Protein coding | 24 |
| ENSG00000056972 | 1.40 | 4.52E-13 | TRAF3IP2 | Protein coding | 24 |
| ENSG00000215244 | 1.40 | 8.42E-06 | AL137145.1 | Protein coding | 24 |
| ENSG00000121764 | 1.40 | 0.012279234 | HCRTR1 | Protein coding | 24 |
| ENSG00000173821 | 1.40 | 3.29E-23 | RNF213 | Protein coding | 24 |
| ENSG00000170959 | -1.40 | 0.033130083 | DCDC5 | Protein coding | 24 |
| ENSG00000102755 | 1.40 | 2.80E-07 | FLT1 | Protein coding | 24 |
| ENSG00000160932 | 1.40 | 0.003154654 | LY6E | Protein coding | 24 |
| ENSG00000196954 | 1.40 | 4.64E-54 | CASP4 | Protein coding | 24 |
| ENSG00000169507 | -1.40 | 0.000325266 | SLC38A11 | Protein coding | 24 |
| ENSG00000020181 | 1.39 | 3.30E-08 | GPR124 | Protein coding | 24 |
| ENSG00000106546 | 1.39 | 9.71E-52 | AHR | Protein coding | 24 |
| ENSG00000107968 | 1.39 | 4.14E-45 | MAP3K8 | Protein coding | 24 |
| ENSG00000119655 | 1.39 | 3.77E-13 | NPC2 | Protein coding | 24 |
| ENSG00000163235 | 1.39 | 9.27E-14 | TGFA | Protein coding | 24 |
| ENSG00000249992 | 1.39 | 0.008189849 | TMEM158 | Protein coding | 24 |
| ENSG00000205730 | 1.39 | 1.81E-17 | ITPRIPL2 | Protein coding | 24 |
| ENSG00000135677 | 1.39 | 1.78E-30 | GNS | Protein coding | 24 |
| ENSG00000174600 | 1.39 | 9.15E-05 | CMKLR1 | Protein coding | 24 |
| ENSG00000103811 | 1.39 | 1.10E-12 | CTSH | Protein coding | 24 |
| ENSG00000106976 | -1.38 | 0.000708439 | DNM1 | Protein coding | 24 |
| ENSG00000184731 | -1.38 | 5.43E-07 | FAM110C | Protein coding | 24 |
| ENSG00000183134 | -1.38 | 5.92E-06 | PTGDR2 | Protein coding | 24 |
| ENSG00000132205 | 1.38 | 4.71E-20 | EMILIN2 | Protein coding | 24 |
| ENSG00000186088 | 1.38 | 2.57E-26 | PION | Protein coding | 24 |
| ENSG00000108771 | 1.38 | 8.06E-05 | DHX58 | Protein coding | 24 |
| ENSG00000170379 | 1.38 | 3.78E-19 | FAM115C | Protein coding | 24 |
| ENSG00000135116 | -1.38 | 4.85E-06 | HRK | Protein coding | 24 |
| ENSG00000197142 | 1.38 | 2.85E-64 | ACSL5 | Protein coding | 24 |
| ENSG00000139610 | -1.38 | 0.029145218 | CELA1 | Protein coding | 24 |
| ENSG00000120896 | -1.38 | 7.93E-46 | SORBS3 | Protein coding | 24 |
| ENSG00000137098 | -1.38 | 4.31E-06 | SPAG8 | Protein coding | 24 |
| ENSG00000174939 | -1.37 | 0.038476229 | ASPHD1 | Protein coding | 24 |
| ENSG00000185909 | 1.37 | 6.02E-06 | KLHDC8B | Protein coding | 24 |
| ENSG00000258227 | 1.37 | 3.28E-05 | CLEC5A | Protein coding | 24 |
| ENSG00000102048 | -1.37 | 0.04733001 | ASB9 | Protein coding | 24 |
| ENSG00000186074 | 1.37 | 8.42E-05 | CD300LF | Protein coding | 24 |
| ENSG00000156869 | 1.37 | 0.00020871 | FRRS1 | Protein coding | 24 |
| ENSG00000110911 | 1.36 | 8.28E-46 | SLC11A2 | Protein coding | 24 |
| ENSG00000185187 | -1.36 | 4.75E-36 | SIGIRR | Protein coding | 24 |
| ENSG00000183773 | -1.36 | 7.11E-05 | AIFM3 | Protein coding | 24 |
| ENSG00000196517 | 1.36 | 4.58E-05 | SLC6A9 | Protein coding | 24 |
| ENSG00000100911 | 1.36 | 8.96E-14 | PSME2 | Protein coding | 24 |
| ENSG00000127947 | 1.36 | 2.98E-27 | PTPN12 | Protein coding | 24 |
| ENSG00000135519 | -1.36 | 4.00E-10 | KCNH3 | Protein coding | 24 |
| ENSG00000267534 | 1.36 | 5.65E-08 | S1PR2 | Protein coding | 24 |
| ENSG00000172426 | 1.36 | 0.000205882 | RSPH9 | Protein coding | 24 |
| ENSG00000111110 | 1.36 | 1.35E-06 | PPM1H | Protein coding | 24 |
| ENSG00000172183 | 1.36 | 6.54E-13 | ISG20 | Protein coding | 24 |
| ENSG00000141458 | 1.36 | 1.16E-28 | NPC1 | Protein coding | 24 |
| ENSG00000162482 | -1.36 | 0.013275836 | AKR7A3 | Protein coding | 24 |
| ENSG00000102554 | 1.36 | 2.72E-12 | KLF5 | Protein coding | 24 |
| ENSG00000129270 | -1.35 | 1.69E-06 | MMP28 | Protein coding | 24 |
| ENSG00000146592 | -1.35 | 5.42E-22 | CREB5 | Protein coding | 24 |
| ENSG00000079482 | -1.35 | 1.65E-05 | OPHN1 | Protein coding | 24 |
| ENSG00000100292 | 1.35 | 1.48E-12 | HMOX1 | Protein coding | 24 |
| ENSG00000175003 | 1.35 | 9.81E-05 | SLC22A1 | Protein coding | 24 |
| ENSG00000256574 | -1.35 | 0.005036523 | OR13A1 | Protein coding | 24 |
| ENSG00000003402 | 1.35 | 1.00E-62 | CFLAR | Protein coding | 24 |
| ENSG00000107551 | 1.35 | 6.98E-15 | RASSF4 | Protein coding | 24 |
| ENSG00000044459 | 1.35 | 8.10E-10 | CNTLN | Protein coding | 24 |
| ENSG00000176014 | 1.35 | 1.78E-10 | TUBB6 | Protein coding | 24 |
| ENSG00000114405 | 1.35 | 6.51E-05 | C3orf14 | Protein coding | 24 |
| ENSG00000134107 | 1.35 | 6.35E-26 | BHLHE40 | Protein coding | 24 |
| ENSG00000158966 | -1.35 | 0.000154581 | CACHD1 | Protein coding | 24 |
| ENSG00000100427 | -1.35 | 1.23E-08 | MLC1 | Protein coding | 24 |
| ENSG00000038427 | 1.35 | 2.35E-05 | VCAN | Protein coding | 24 |
| ENSG00000204936 | 1.35 | 4.63E-06 | CD177 | Protein coding | 24 |
| ENSG00000149489 | 1.35 | 2.33E-09 | ROM1 | Protein coding | 24 |
| ENSG00000146094 | 1.34 | 6.96E-14 | DOK3 | Protein coding | 24 |
| ENSG00000183307 | 1.34 | 8.88E-06 | CECR6 | Protein coding | 24 |
| ENSG00000127920 | 1.34 | 9.84E-08 | GNG11 | Protein coding | 24 |
| ENSG00000152760 | 1.34 | 0.007049549 | TCTEX1D1 | Protein coding | 24 |
| ENSG00000082397 | 1.34 | 4.16E-16 | EPB41L3 | Protein coding | 24 |
| ENSG00000169554 | 1.34 | 4.44E-35 | ZEB2 | Protein coding | 24 |
| ENSG00000118523 | 1.34 | 1.65E-06 | CTGF | Protein coding | 24 |
| ENSG00000169252 | -1.34 | 5.19E-22 | ADRB2 | Protein coding | 24 |
| ENSG00000088298 | 1.34 | 5.29E-17 | EDEM2 | Protein coding | 24 |
| ENSG00000102096 | 1.34 | 1.96E-34 | PIM2 | Protein coding | 24 |
| ENSG00000090924 | 1.34 | 4.61E-23 | PLEKHG2 | Protein coding | 24 |
| ENSG00000175643 | 1.34 | 3.25E-15 | RMI2 | Protein coding | 24 |
| ENSG00000143217 | 1.34 | 0.000368713 | PVRL4 | Protein coding | 24 |
| ENSG00000066468 | -1.34 | 0.000491847 | FGFR2 | Protein coding | 24 |
| ENSG00000085449 | 1.33 | 5.03E-38 | WDFY1 | Protein coding | 24 |
| ENSG00000155307 | 1.33 | 5.26E-24 | SAMSN1 | Protein coding | 24 |
| ENSG00000204169 | -1.33 | 0.025357119 | AGAP7 | Protein coding | 24 |
| ENSG00000068366 | 1.33 | 1.93E-35 | ACSL4 | Protein coding | 24 |
| ENSG00000241839 | 1.33 | 1.62E-19 | PLEKHO2 | Protein coding | 24 |
| ENSG00000173559 | 1.33 | 2.30E-49 | NABP1 | Protein coding | 24 |
| ENSG00000158517 | 1.33 | 2.37E-09 | NCF1 | Protein coding | 24 |
| ENSG00000145687 | -1.33 | 2.30E-20 | SSBP2 | Protein coding | 24 |
| ENSG00000198283 | 1.32 | 0.019028097 | OR5B21 | Protein coding | 24 |
| ENSG00000182557 | -1.32 | 0.000750342 | SPNS3 | Protein coding | 24 |
| ENSG00000198355 | 1.32 | 1.10E-11 | PIM3 | Protein coding | 24 |
| ENSG00000007866 | 1.32 | 2.38E-06 | TEAD3 | Protein coding | 24 |
| ENSG00000075399 | 1.32 | 7.39E-15 | VPS9D1 | Protein coding | 24 |
| ENSG00000085733 | 1.32 | 0.000227235 | CTTN | Protein coding | 24 |
| ENSG00000180071 | -1.32 | 2.65E-06 | ANKRD18A | Protein coding | 24 |
| ENSG00000184232 | 1.32 | 4.05E-10 | OAF | Protein coding | 24 |
| ENSG00000161888 | -1.32 | 0.010224292 | SPC24 | Protein coding | 24 |
| ENSG00000100284 | 1.32 | 1.17E-14 | TOM1 | Protein coding | 24 |
| ENSG00000147443 | -1.32 | 4.89E-25 | DOK2 | Protein coding | 24 |
| ENSG00000173706 | 1.32 | 1.10E-27 | HEG1 | Protein coding | 24 |
| ENSG00000182600 | -1.32 | 0.001095056 | C2orf82 | Protein coding | 24 |
| ENSG00000057294 | -1.32 | 0.02264475 | PKP2 | Protein coding | 24 |
| ENSG00000013619 | 1.31 | 2.37E-07 | MAMLD1 | Protein coding | 24 |
| ENSG00000116701 | 1.31 | 4.01E-14 | NCF2 | Protein coding | 24 |
| ENSG00000151117 | -1.31 | 8.65E-13 | TMEM86A | Protein coding | 24 |
| ENSG00000139193 | -1.31 | 1.34E-26 | CD27 | Protein coding | 24 |
| ENSG00000173868 | -1.31 | 2.37E-09 | PHOSPHO1 | Protein coding | 24 |
| ENSG00000146376 | 1.31 | 1.07E-16 | ARHGAP18 | Protein coding | 24 |
| ENSG00000130830 | 1.31 | 2.65E-11 | MPP1 | Protein coding | 24 |
| ENSG00000189292 | -1.31 | 0.026707174 | FAM150B | Protein coding | 24 |
| ENSG00000085491 | 1.31 | 2.30E-29 | SLC25A24 | Protein coding | 24 |
| ENSG00000132357 | 1.31 | 1.32E-20 | CARD6 | Protein coding | 24 |
| ENSG00000025039 | 1.31 | 5.76E-21 | RRAGD | Protein coding | 24 |
| ENSG00000159128 | 1.31 | 2.18E-20 | IFNGR2 | Protein coding | 24 |
| ENSG00000091436 | 1.31 | 3.21E-19 | MLTK | Protein coding | 24 |
| ENSG00000050030 | -1.31 | 1.91E-05 | KIAA2022 | Protein coding | 24 |
| ENSG00000148154 | 1.30 | 1.24E-41 | UGCG | Protein coding | 24 |
| ENSG00000123609 | 1.30 | 4.62E-17 | NMI | Protein coding | 24 |
| ENSG00000176884 | 1.30 | 0.017108544 | GRIN1 | Protein coding | 24 |
| ENSG00000142192 | 1.30 | 1.29E-18 | APP | Protein coding | 24 |
| ENSG00000116991 | 1.30 | 6.36E-14 | SIPA1L2 | Protein coding | 24 |
| ENSG00000143570 | 1.30 | 4.66E-20 | SLC39A1 | Protein coding | 24 |
| ENSG00000196923 | 1.30 | 4.62E-16 | PDLIM7 | Protein coding | 24 |
| ENSG00000135643 | -1.30 | 4.26E-05 | KCNMB4 | Protein coding | 24 |
| ENSG00000140464 | 1.30 | 5.54E-10 | PML | Protein coding | 24 |
| ENSG00000206077 | -1.30 | 4.75E-14 | ZDHHC11B | Protein coding | 24 |
| ENSG00000172159 | 1.30 | 1.45E-09 | FRMD3 | Protein coding | 24 |
| ENSG00000059378 | 1.30 | 2.45E-09 | PARP12 | Protein coding | 24 |
| ENSG00000136161 | -1.29 | 2.98E-14 | RCBTB2 | Protein coding | 24 |
| ENSG00000128040 | -1.29 | 0.038076242 | SPINK2 | Protein coding | 24 |
| ENSG00000104722 | -1.29 | 0.000209413 | NEFM | Protein coding | 24 |
| ENSG00000091428 | 1.29 | 0.01203222 | RAPGEF4 | Protein coding | 24 |
| ENSG00000196782 | -1.29 | 2.60E-19 | MAML3 | Protein coding | 24 |
| ENSG00000154640 | 1.29 | 1.37E-20 | BTG3 | Protein coding | 24 |
| ENSG00000157985 | -1.29 | 3.92E-07 | AGAP1 | Protein coding | 24 |
| ENSG00000163803 | -1.29 | 3.28E-10 | PLB1 | Protein coding | 24 |
| ENSG00000121060 | 1.29 | 1.68E-15 | TRIM25 | Protein coding | 24 |
| ENSG00000125733 | 1.29 | 9.98E-24 | TRIP10 | Protein coding | 24 |
| ENSG00000174125 | 1.29 | 5.84E-39 | TLR1 | Protein coding | 24 |
| ENSG00000137575 | 1.29 | 1.25E-31 | SDCBP | Protein coding | 24 |
| ENSG00000105479 | -1.29 | 0.00894312 | CCDC114 | Protein coding | 24 |
| ENSG00000144668 | 1.29 | 0.003159259 | ITGA9 | Protein coding | 24 |
| ENSG00000169994 | 1.28 | 9.41E-05 | MYO7B | Protein coding | 24 |
| ENSG00000180611 | 1.28 | 8.84E-15 | MB21D2 | Protein coding | 24 |
| ENSG00000150681 | -1.28 | 1.82E-10 | RGS18 | Protein coding | 24 |
| ENSG00000105967 | 1.28 | 9.67E-24 | TFEC | Protein coding | 24 |
| ENSG00000102837 | 1.28 | 8.60E-11 | OLFM4 | Protein coding | 24 |
| ENSG00000197614 | 1.28 | 0.038905589 | MFAP5 | Protein coding | 24 |
| ENSG00000127951 | -1.28 | 0.000344473 | FGL2 | Protein coding | 24 |
| ENSG00000173578 | 1.28 | 9.43E-05 | XCR1 | Protein coding | 24 |
| ENSG00000119535 | -1.28 | 6.24E-13 | CSF3R | Protein coding | 24 |
| ENSG00000085831 | 1.28 | 0.009796759 | TTC39A | Protein coding | 24 |
| ENSG00000176092 | -1.28 | 3.71E-05 | AIM1L | Protein coding | 24 |
| ENSG00000167291 | 1.28 | 5.04E-16 | TBC1D16 | Protein coding | 24 |
| ENSG00000138678 | -1.28 | 1.96E-11 | AGPAT9 | Protein coding | 24 |
| ENSG00000162894 | -1.28 | 1.52E-36 | FAIM3 | Protein coding | 24 |
| ENSG00000076067 | -1.28 | 1.58E-07 | RBMS2 | Protein coding | 24 |
| ENSG00000137731 | -1.28 | 0.030455063 | FXYD2 | Protein coding | 24 |
| ENSG00000102678 | -1.28 | 4.03E-06 | FGF9 | Protein coding | 24 |
| ENSG00000039319 | 1.28 | 1.10E-21 | ZFYVE16 | Protein coding | 24 |
| ENSG00000155096 | 1.28 | 9.24E-35 | AZIN1 | Protein coding | 24 |
| ENSG00000186815 | -1.28 | 5.39E-37 | TPCN1 | Protein coding | 24 |
| ENSG00000075426 | 1.28 | 8.13E-16 | FOSL2 | Protein coding | 24 |
| ENSG00000141968 | 1.27 | 8.47E-31 | VAV1 | Protein coding | 24 |
| ENSG00000004838 | -1.27 | 5.85E-08 | ZMYND10 | Protein coding | 24 |
| ENSG00000100647 | 1.27 | 2.19E-27 | KIAA0247 | Protein coding | 24 |
| ENSG00000107242 | -1.27 | 4.59E-08 | PIP5K1B | Protein coding | 24 |
| ENSG00000176845 | 1.27 | 3.76E-13 | METRNL | Protein coding | 24 |
| ENSG00000073792 | 1.27 | 1.16E-11 | IGF2BP2 | Protein coding | 24 |
| ENSG00000167549 | -1.27 | 2.70E-06 | CORO6 | Protein coding | 24 |
| ENSG00000169744 | -1.27 | 0.001461277 | LDB2 | Protein coding | 24 |
| ENSG00000154240 | 1.27 | 2.29E-06 | CEP112 | Protein coding | 24 |
| ENSG00000130203 | -1.27 | 0.011461643 | APOE | Protein coding | 24 |
| ENSG00000178038 | -1.27 | 5.78E-12 | ALS2CL | Protein coding | 24 |
| ENSG00000105808 | -1.27 | 0.000529432 | RASA4 | Protein coding | 24 |
| ENSG00000049449 | 1.27 | 3.16E-11 | RCN1 | Protein coding | 24 |
| ENSG00000106571 | 1.27 | 0.025244258 | GLI3 | Protein coding | 24 |
| ENSG00000043462 | 1.27 | 7.03E-30 | LCP2 | Protein coding | 24 |
| ENSG00000136274 | 1.26 | 0.01973781 | NACAD | Protein coding | 24 |
| ENSG00000184014 | 1.26 | 7.83E-42 | DENND5A | Protein coding | 24 |
| ENSG00000143409 | -1.26 | 1.58E-21 | FAM63A | Protein coding | 24 |
| ENSG00000159403 | 1.26 | 0.000157911 | C1R | Protein coding | 24 |
| ENSG00000014257 | -1.26 | 9.52E-08 | ACPP | Protein coding | 24 |
| ENSG00000064763 | 1.26 | 4.48E-14 | FAR2 | Protein coding | 24 |
| ENSG00000145103 | 1.26 | 0.005247294 | ILDR1 | Protein coding | 24 |
| ENSG00000167768 | -1.26 | 2.16E-09 | KRT1 | Protein coding | 24 |
| ENSG00000136929 | -1.26 | 7.16E-06 | HEMGN | Protein coding | 24 |
| ENSG00000108984 | -1.26 | 6.51E-10 | MAP2K6 | Protein coding | 24 |
| ENSG00000113657 | 1.26 | 0.007768619 | DPYSL3 | Protein coding | 24 |
| ENSG00000183735 | 1.26 | 4.28E-48 | TBK1 | Protein coding | 24 |
| ENSG00000168404 | 1.26 | 3.39E-14 | MLKL | Protein coding | 24 |
| ENSG00000101294 | 1.25 | 8.32E-16 | HM13 | Protein coding | 24 |
| ENSG00000113742 | 1.25 | 2.09E-41 | CPEB4 | Protein coding | 24 |
| ENSG00000165259 | 1.25 | 1.38E-05 | HDX | Protein coding | 24 |
| ENSG00000137496 | 1.25 | 2.84E-08 | IL18BP | Protein coding | 24 |
| ENSG00000165806 | 1.25 | 9.85E-15 | CASP7 | Protein coding | 24 |
| ENSG00000135378 | 1.25 | 3.49E-07 | PRRG4 | Protein coding | 24 |
| ENSG00000132141 | -1.25 | 0.004341053 | CCT6B | Protein coding | 24 |
| ENSG00000108861 | 1.25 | 1.97E-15 | DUSP3 | Protein coding | 24 |
| ENSG00000132334 | 1.25 | 3.23E-20 | PTPRE | Protein coding | 24 |
| ENSG00000145708 | -1.25 | 0.035457029 | CRHBP | Protein coding | 24 |
| ENSG00000008513 | 1.25 | 5.07E-18 | ST3GAL1 | Protein coding | 24 |
| ENSG00000168994 | 1.25 | 4.48E-08 | PXDC1 | Protein coding | 24 |
| ENSG00000103043 | 1.25 | 7.33E-25 | VAC14 | Protein coding | 24 |
| ENSG00000204267 | 1.25 | 6.53E-18 | TAP2 | Protein coding | 24 |
| ENSG00000134668 | -1.25 | 0.001007457 | SPOCD1 | Protein coding | 24 |
| ENSG00000072274 | 1.25 | 1.03E-38 | TFRC | Protein coding | 24 |
| ENSG00000164430 | 1.25 | 6.56E-16 | MB21D1 | Protein coding | 24 |
| ENSG00000146859 | 1.24 | 4.33E-15 | TMEM140 | Protein coding | 24 |
| ENSG00000141540 | -1.24 | 4.92E-19 | TTYH2 | Protein coding | 24 |
| ENSG00000116260 | 1.24 | 3.27E-11 | QSOX1 | Protein coding | 24 |
| ENSG00000090674 | 1.24 | 7.38E-12 | MCOLN1 | Protein coding | 24 |
| ENSG00000188004 | 1.24 | 5.47E-08 | C1orf204 | Protein coding | 24 |
| ENSG00000168229 | -1.24 | 4.86E-13 | PTGDR | Protein coding | 24 |
| ENSG00000152672 | -1.24 | 0.047045123 | CLEC4F | Protein coding | 24 |
| ENSG00000111321 | 1.24 | 3.81E-08 | LTBR | Protein coding | 24 |
| ENSG00000198363 | 1.24 | 3.53E-23 | ASPH | Protein coding | 24 |
| ENSG00000170293 | 1.24 | 4.06E-14 | CMTM8 | Protein coding | 24 |
| ENSG00000111224 | 1.24 | 2.82E-22 | PARP11 | Protein coding | 24 |
| ENSG00000137841 | -1.24 | 3.33E-64 | PLCB2 | Protein coding | 24 |
| ENSG00000090975 | -1.24 | 4.19E-24 | PITPNM2 | Protein coding | 24 |
| ENSG00000212743 | 1.24 | 6.58E-08 | DKFZP667F0711 | Protein coding | 24 |
| ENSG00000137507 | 1.24 | 1.44E-12 | LRRC32 | Protein coding | 24 |
| ENSG00000007062 | 1.24 | 0.010183154 | PROM1 | Protein coding | 24 |
| ENSG00000185567 | -1.24 | 0.000164145 | AHNAK2 | Protein coding | 24 |
| ENSG00000147883 | 1.23 | 1.48E-07 | CDKN2B | Protein coding | 24 |
| ENSG00000136560 | 1.23 | 4.60E-45 | TANK | Protein coding | 24 |
| ENSG00000198964 | 1.23 | 7.81E-26 | SGMS1 | Protein coding | 24 |
| ENSG00000013364 | 1.23 | 6.26E-13 | MVP | Protein coding | 24 |
| ENSG00000164342 | 1.23 | 2.96E-09 | TLR3 | Protein coding | 24 |
| ENSG00000086065 | 1.23 | 1.12E-17 | CHMP5 | Protein coding | 24 |
| ENSG00000119139 | 1.23 | 6.70E-15 | TJP2 | Protein coding | 24 |
| ENSG00000106537 | 1.23 | 2.13E-17 | TSPAN13 | Protein coding | 24 |
| ENSG00000143507 | 1.23 | 3.38E-27 | DUSP10 | Protein coding | 24 |
| ENSG00000109861 | 1.23 | 0.001427245 | CTSC | Protein coding | 24 |
| ENSG00000269089 | 1.23 | 0.01104754 | AP003733.1 | Protein coding | 24 |
| ENSG00000080573 | -1.23 | 3.89E-10 | COL5A3 | Protein coding | 24 |
| ENSG00000115112 | -1.23 | 6.76E-06 | TFCP2L1 | Protein coding | 24 |
| ENSG00000121858 | 1.23 | 0.001328153 | TNFSF10 | Protein coding | 24 |
| ENSG00000204131 | -1.23 | 8.46E-15 | NHSL2 | Protein coding | 24 |
| ENSG00000113296 | -1.23 | 3.17E-07 | THBS4 | Protein coding | 24 |
