# Supplementary material for: An integrative genomics approach identifies novel pathways that influence candidaemia susceptibility
Source: PLoS One. 2017 Jul 20;12(7):e0180824. doi: 10.1371/journal.pone.0180824 (PMC5519064; doi:10.1371/journal.pone.0180824)
Supplement: S2 Table — (DOCX) [file pone.0180824.s006.docx]

Table S2. A total of 246 protein-coding genes were differentially expressed at both 4 and 24 hour-*Candida* stimulation, showing >1.5-fold higher expression compared to RPMI medium used as control.

| Ensembl ID | Gene |
| --- | --- |
| ENSG00000154262 | ABCA6 |
| ENSG00000166016 | ABTB2 |
| ENSG00000217825 | AC099552.4 |
| ENSG00000151726 | ACSL1 |
| ENSG00000148926 | ADM |
| ENSG00000162433 | AK4 |
| ENSG00000215306 | AL135998.1 |
| ENSG00000114019 | AMOTL2 |
| ENSG00000128383 | APOBEC3A |
| ENSG00000103569 | AQP9 |
| ENSG00000162772 | ATF3 |
| ENSG00000158470 | B4GALT5 |
| ENSG00000156127 | BATF |
| ENSG00000168062 | BATF2 |
| ENSG00000123685 | BATF3 |
| ENSG00000064787 | BCAS1 |
| ENSG00000140379 | BCL2A1 |
| ENSG00000121380 | BCL2L14 |
| ENSG00000187479 | C11orf96 |
| ENSG00000166920 | C15orf48 |
| ENSG00000197982 | C1orf122 |
| ENSG00000125462 | C1orf61 |
| ENSG00000173918 | C1QTNF1 |
| ENSG00000125730 | C3 |
| ENSG00000141837 | CACNA1A |
| ENSG00000008118 | CAMK1G |
| ENSG00000137757 | CASP5 |
| ENSG00000108691 | CCL2 |
| ENSG00000115009 | CCL20 |
| ENSG00000167236 | CCL23 |
| ENSG00000006075 | CCL3 |
| ENSG00000205021 | CCL3L1 |
| ENSG00000129277 | CCL4 |
| ENSG00000205020 | CCL4L1 |
| ENSG00000197262 | CCL4L2 |
| ENSG00000108688 | CCL7 |
| ENSG00000108700 | CCL8 |
| ENSG00000101331 | CCM2L |
| ENSG00000121797 | CCRL2 |
| ENSG00000120217 | CD274 |
| ENSG00000121594 | CD80 |
| ENSG00000149798 | CDC42EP2 |
| ENSG00000198848 | CES1 |
| ENSG00000243649 | CFB |
| ENSG00000138135 | CH25H |
| ENSG00000114737 | CISH |
| ENSG00000166523 | CLEC4E |
| ENSG00000153132 | CLGN |
| ENSG00000169504 | CLIC4 |
| ENSG00000134326 | CMPK2 |
| ENSG00000135678 | CPM |
| ENSG00000164400 | CSF2 |
| ENSG00000108342 | CSF3 |
| ENSG00000163739 | CXCL1 |
| ENSG00000169245 | CXCL10 |
| ENSG00000081041 | CXCL2 |
| ENSG00000163734 | CXCL3 |
| ENSG00000163735 | CXCL5 |
| ENSG00000124875 | CXCL6 |
| ENSG00000137869 | CYP19A1 |
| ENSG00000134716 | CYP2J2 |
| ENSG00000107201 | DDX58 |
| ENSG00000137628 | DDX60 |
| ENSG00000164825 | DEFB1 |
| ENSG00000105928 | DFNA5 |
| ENSG00000108771 | DHX58 |
| ENSG00000136048 | DRAM1 |
| ENSG00000163840 | DTX3L |
| ENSG00000138166 | DUSP5 |
| ENSG00000105246 | EBI3 |
| ENSG00000078401 | EDN1 |
| ENSG00000135373 | EHF |
| ENSG00000055332 | EIF2AK2 |
| ENSG00000164181 | ELOVL7 |
| ENSG00000133106 | EPSTI1 |
| ENSG00000157557 | ETS2 |
| ENSG00000253831 | ETV3L |
| ENSG00000010030 | ETV7 |
| ENSG00000117525 | F3 |
| ENSG00000198673 | FAM19A2 |
| ENSG00000026103 | FAS |
| ENSG00000116663 | FBXO6 |
| ENSG00000149557 | FEZ1 |
| ENSG00000126262 | FFAR2 |
| ENSG00000179431 | FJX1 |
| ENSG00000102755 | FLT1 |
| ENSG00000075618 | FSCN1 |
| ENSG00000106701 | FSD1L |
| ENSG00000123689 | G0S2 |
| ENSG00000130222 | GADD45G |
| ENSG00000131979 | GCH1 |
| ENSG00000121743 | GJA3 |
| ENSG00000165474 | GJB2 |
| ENSG00000139572 | GPR84 |
| ENSG00000013588 | GPRC5A |
| ENSG00000180875 | GREM2 |
| ENSG00000182782 | HCAR2 |
| ENSG00000255398 | HCAR3 |
| ENSG00000130589 | HELZ2 |
| ENSG00000138646 | HERC5 |
| ENSG00000138642 | HERC6 |
| ENSG00000164683 | HEY1 |
| ENSG00000105707 | HPN |
| ENSG00000196639 | HRH1 |
| ENSG00000117594 | HSD11B1 |
| ENSG00000090339 | ICAM1 |
| ENSG00000137331 | IER3 |
| ENSG00000165949 | IFI27 |
| ENSG00000068079 | IFI35 |
| ENSG00000137965 | IFI44 |
| ENSG00000137959 | IFI44L |
| ENSG00000126709 | IFI6 |
| ENSG00000115267 | IFIH1 |
| ENSG00000185745 | IFIT1 |
| ENSG00000119922 | IFIT2 |
| ENSG00000119917 | IFIT3 |
| ENSG00000152778 | IFIT5 |
| ENSG00000185885 | IFITM1 |
| ENSG00000142089 | IFITM3 |
| ENSG00000111537 | IFNG |
| ENSG00000136634 | IL10 |
| ENSG00000113302 | IL12B |
| ENSG00000169194 | IL13 |
| ENSG00000134470 | IL15RA |
| ENSG00000142224 | IL19 |
| ENSG00000115008 | IL1A |
| ENSG00000125538 | IL1B |
| ENSG00000136689 | IL1RN |
| ENSG00000197272 | IL27 |
| ENSG00000134460 | IL2RA |
| ENSG00000136688 | IL36G |
| ENSG00000136695 | IL36RN |
| ENSG00000104951 | IL4I1 |
| ENSG00000136244 | IL6 |
| ENSG00000104432 | IL7 |
| ENSG00000169429 | IL8 |
| ENSG00000122641 | INHBA |
| ENSG00000134070 | IRAK2 |
| ENSG00000185507 | IRF7 |
| ENSG00000102794 | IRG1 |
| ENSG00000187608 | ISG15 |
| ENSG00000172183 | ISG20 |
| ENSG00000105855 | ITGB8 |
| ENSG00000123700 | KCNJ2 |
| ENSG00000102554 | KLF5 |
| ENSG00000117009 | KMO |
| ENSG00000089692 | LAG3 |
| ENSG00000196878 | LAMB3 |
| ENSG00000078081 | LAMP3 |
| ENSG00000198121 | LPAR1 |
| ENSG00000160932 | LY6E |
| ENSG00000183742 | MACC1 |
| ENSG00000185022 | MAFF |
| ENSG00000107968 | MAP3K8 |
| ENSG00000155130 | MARCKS |
| ENSG00000175471 | MCTP1 |
| ENSG00000168389 | MFSD2A |
| ENSG00000074416 | MGLL |
| ENSG00000166670 | MMP10 |
| ENSG00000157227 | MMP14 |
| ENSG00000178860 | MSC |
| ENSG00000125148 | MT2A |
| ENSG00000157601 | MX1 |
| ENSG00000183486 | MX2 |
| ENSG00000104320 | NBN |
| ENSG00000204099 | NEU4 |
| ENSG00000162614 | NEXN |
| ENSG00000100906 | NFKBIA |
| ENSG00000144802 | NFKBIZ |
| ENSG00000131669 | NINJ1 |
| ENSG00000089127 | OAS1 |
| ENSG00000111335 | OAS2 |
| ENSG00000111331 | OAS3 |
| ENSG00000135114 | OASL |
| ENSG00000184221 | OLIG1 |
| ENSG00000205927 | OLIG2 |
| ENSG00000171631 | P2RY6 |
| ENSG00000059378 | PARP12 |
| ENSG00000138496 | PARP9 |
| ENSG00000162493 | PDPN |
| ENSG00000170525 | PFKFB3 |
| ENSG00000181649 | PHLDA2 |
| ENSG00000124102 | PI3 |
| ENSG00000102096 | PIM2 |
| ENSG00000122861 | PLAU |
| ENSG00000011422 | PLAUR |
| ENSG00000075651 | PLD1 |
| ENSG00000115956 | PLEK |
| ENSG00000188313 | PLSCR1 |
| ENSG00000140464 | PML |
| ENSG00000180316 | PNPLA1 |
| ENSG00000183657 | PP13439 |
| ENSG00000087074 | PPP1R15A |
| ENSG00000152229 | PSTPIP2 |
| ENSG00000073756 | PTGS2 |
| ENSG00000163661 | PTX3 |
| ENSG00000143344 | RGL1 |
| ENSG00000132669 | RIN2 |
| ENSG00000104312 | RIPK2 |
| ENSG00000137393 | RNF144B |
| ENSG00000235531 | RP11-383H13.1 |
| ENSG00000134321 | RSAD2 |
| ENSG00000136514 | RTP4 |
| ENSG00000177409 | SAMD9L |
| ENSG00000105711 | SCN1B |
| ENSG00000197632 | SERPINB2 |
| ENSG00000163082 | SGPP2 |
| ENSG00000088827 | SIGLEC1 |
| ENSG00000026751 | SLAMF7 |
| ENSG00000079215 | SLC1A3 |
| ENSG00000160326 | SLC2A6 |
| ENSG00000138821 | SLC39A8 |
| ENSG00000124107 | SLPI |
| ENSG00000214872 | SMTNL1 |
| ENSG00000185338 | SOCS1 |
| ENSG00000184557 | SOCS3 |
| ENSG00000112096 | SOD2 |
| ENSG00000186583 | SPATC1 |
| ENSG00000196141 | SPATS2L |
| ENSG00000164266 | SPINK1 |
| ENSG00000180616 | SSTR2 |
| ENSG00000183473 | SSTR3 |
| ENSG00000172403 | SYNPO2 |
| ENSG00000148737 | TCF7L2 |
| ENSG00000196116 | TDRD7 |
| ENSG00000105825 | TFPI2 |
| ENSG00000137462 | TLR2 |
| ENSG00000121900 | TMEM54 |
| ENSG00000232810 | TNF |
| ENSG00000185215 | TNFAIP2 |
| ENSG00000123610 | TNFAIP6 |
| ENSG00000121858 | TNFSF10 |
| ENSG00000181634 | TNFSF15 |
| ENSG00000050730 | TNIP3 |
| ENSG00000132109 | TRIM21 |
| ENSG00000132274 | TRIM22 |
| ENSG00000152503 | TRIM36 |
| ENSG00000125733 | TRIP10 |
| ENSG00000136810 | TXN |
| ENSG00000156587 | UBE2L6 |
| ENSG00000100024 | UPB1 |
| ENSG00000184979 | USP18 |
| ENSG00000114251 | WNT5A |
| ENSG00000132530 | XAF1 |
| ENSG00000168334 | XIRP1 |
| ENSG00000149289 | ZC3H12C |
