# Supplementary material for: An integrative genomics approach identifies novel pathways that influence candidaemia susceptibility
Source: PLoS One. 2017 Jul 20;12(7):e0180824. doi: 10.1371/journal.pone.0180824 (PMC5519064; doi:10.1371/journal.pone.0180824)
Supplement: S3 Table — (DOCX) [file pone.0180824.s007.docx]

Table S3. Pathway enrichment analysis on differentially expressed protein-coding genes that showed >1.5 higher expression compared to RPMI medium in response to 4 hour-*Candida* stimulation.

| Pathway name | Set size | Candidates | p-value | q-value | Pathway source | -log_10_P |
| --- | --- | --- | --- | --- | --- | --- |
| Immune System | 1174 | 60 (5.2%) | 4.77E-13 | 5.33E-11 | Reactome | 12.32 |
| **Cytokine Signaling in Immune system** | 376 | 32 (8.5%) | 1.36E-12 | 1.06E-10 | Reactome | 11.87 |
| **Interferon Signaling** | 68 | 13 (19.1%) | 4.90E-10 | 2.74E-08 | Reactome | 9.31 |
| Chemokine receptors bind chemokines | 60 | 11 (19.0%) | 1.20E-08 | 4.09E-07 | Reactome | 7.92 |
| ISG15 antiviral mechanism | 31 | 8 (25.8%) | 9.71E-08 | 2.00E-06 | Reactome | 7.01 |
| Antiviral mechanism by IFN-stimulated genes | 31 | 8 (25.8%) | 9.71E-08 | 2.00E-06 | Reactome | 7.01 |
| Class A/1 (Rhodopsin-like receptors) | 326 | 22 (6.8%) | 3.00E-07 | 5.23E-06 | Reactome | 6.52 |
| Innate Immune System | 709 | 32 (4.6%) | 5.44E-06 | 8.20E-05 | Reactome | 5.26 |
| GPCR ligand binding | 454 | 24 (5.4%) | 6.55E-06 | 9.49E-05 | Reactome | 5.18 |
| **RIG-I/MDA5 mediated induction of IFN-alpha/beta pathways** | 53 | 8 (15.1%) | 7.55E-06 | 0.000107 | Reactome | 5.12 |
| Peptide ligand-binding receptors | 199 | 14 (7.2%) | 2.69E-05 | 0.000352 | Reactome | 4.57 |
| Negative regulators of RIG-I/MDA5 signaling | 21 | 5 (23.8%) | 4.21E-05 | 0.000515 | Reactome | 4.38 |
| Signaling by Interleukins | 270 | 16 (5.9%) | 7.20E-05 | 0.000829 | Reactome | 4.14 |
| Interleukin-1 signaling | 43 | 6 (14.0%) | 0.000169 | 0.00177 | Reactome | 3.77 |
| Interleukin-1 processing | 7 | 3 (42.9%) | 0.00024 | 0.00238 | Reactome | 3.62 |
| STING mediated induction of host immune responses | 17 | 4 (23.5%) | 0.000272 | 0.00263 | Reactome | 3.57 |
| Cytosolic sensors of pathogen-associated DNA | 49 | 6 (12.2%) | 0.000353 | 0.00325 | Reactome | 3.45 |
| G alpha (i) signalling events | 243 | 13 (5.4%) | 0.000834 | 0.00718 | Reactome | 3.08 |
| Hydroxycarboxylic acid-binding receptors | 3 | 2 (66.7%) | 0.00112 | 0.0093 | Reactome | 2.95 |
| Regulation of IFNA signaling | 26 | 4 (15.4%) | 0.00149 | 0.012 | Reactome | 2.83 |
| Interferon alpha/beta signaling | 27 | 4 (14.8%) | 0.00172 | 0.0138 | Reactome | 2.76 |
| TRAF3-dependent IRF activation pathway | 13 | 3 (23.1%) | 0.0018 | 0.0141 | Reactome | 2.74 |
| **Dissolution of Fibrin Clot** | 13 | 3 (23.1%) | 0.0018 | 0.0141 | Reactome | 2.74 |
| Alternative complement activation | 4 | 2 (50.0%) | 0.0022 | 0.0165 | Reactome | 2.66 |
| Regulation of IFNG signaling | 14 | 3 (21.4%) | 0.00226 | 0.0165 | Reactome | 2.65 |
| IRF3-mediated induction of type I IFN | 14 | 3 (21.4%) | 0.00226 | 0.0165 | Reactome | 2.65 |
| Regulation of innate immune responses to cytosolic DNA | 16 | 3 (18.8%) | 0.00338 | 0.0232 | Reactome | 2.47 |
| TRAF6 mediated IRF7 activation | 17 | 3 (17.6%) | 0.00404 | 0.0266 | Reactome | 2.39 |
| MyD88 dependent cascade initiated on endosome | 79 | 6 (7.6%) | 0.00433 | 0.0275 | Reactome | 2.36 |
| Toll Like Receptor 7/8 (TLR7/8) Cascade | 79 | 6 (7.6%) | 0.00433 | 0.0275 | Reactome | 2.36 |
| Toll Like Receptor 9 (TLR9) Cascade | 83 | 6 (7.2%) | 0.00551 | 0.0334 | Reactome | 2.26 |
| Toll-Like Receptors Cascades | 138 | 8 (5.8%) | 0.00553 | 0.0334 | Reactome | 2.26 |
| Growth hormone receptor signaling | 19 | 3 (15.8%) | 0.0056 | 0.0334 | Reactome | 2.25 |
| Interferon gamma signaling | 19 | 3 (15.8%) | 0.0056 | 0.0334 | Reactome | 2.25 |
| Activated TLR4 signalling | 110 | 7 (6.4%) | 0.00562 | 0.0334 | Reactome | 2.25 |
| Adaptive Immune System | 612 | 21 (3.5%) | 0.00635 | 0.0368 | Reactome | 2.2 |
| MyD88:Mal cascade initiated on plasma membrane | 86 | 6 (7.0%) | 0.00655 | 0.0369 | Reactome | 2.18 |
| Toll Like Receptor TLR1:TLR2 Cascade | 86 | 6 (7.0%) | 0.00655 | 0.0369 | Reactome | 2.18 |
| Toll Like Receptor TLR6:TLR2 Cascade | 86 | 6 (7.0%) | 0.00655 | 0.0369 | Reactome | 2.18 |
| Toll Like Receptor 2 (TLR2) Cascade | 86 | 6 (7.0%) | 0.00655 | 0.0369 | Reactome | 2.18 |
| Activation of C3 and C5 | 7 | 2 (28.6%) | 0.00742 | 0.0412 | Reactome | 2.13 |
| Death Receptor Signalling | 22 | 3 (13.6%) | 0.00852 | 0.0462 | Reactome | 2.07 |
| Toll Like Receptor 4 (TLR4) Cascade | 119 | 7 (5.9%) | 0.00855 | 0.0462 | Reactome | 2.07 |
| RAF-independent MAPK1/3 activation | 23 | 3 (13.0%) | 0.00966 | 0.0504 | Reactome | 2.02 |
