# Supplementary material for: An integrative genomics approach identifies novel pathways that influence candidaemia susceptibility
Source: PLoS One. 2017 Jul 20;12(7):e0180824. doi: 10.1371/journal.pone.0180824 (PMC5519064; doi:10.1371/journal.pone.0180824)
Supplement: S4 Table — (DOCX) [file pone.0180824.s008.docx]

Table S4. Pathway enrichment analysis on differentially expressed protein-coding genes that showed >1.5 higher expression compared to RPMI medium in response to 24 hour-*Candida* stimulation.

| Pathway name | Set size | | Candidates | | p-value | | q-value | | Pathway source | | | -log_10_P |
| --- | --- | --- | --- | --- | --- | --- | --- | --- | --- | --- | --- | --- |
| Chemokine receptors bind chemokines | | 60 | | 19 (32.8%) | | 9.62E-11 | | 3.04E-08 | | Reactome | 10.02 | |
| Immune System | | 1174 | | 113 (9.8%) | | 2.42E-10 | | 6.38E-08 | | Reactome | 9.62 | |
| **Cytokine Signaling in Immune system** | | 376 | | 50 (13.3%) | | 3.19E-09 | | 4.99E-07 | | Reactome | 8.5 | |
| Class A/1 (Rhodopsin-like receptors) | | 326 | | 44 (13.7%) | | 1.39E-08 | | 1.29E-06 | | Reactome | 7.86 | |
| Extracellular matrix organization | | 264 | | 38 (14.4%) | | 3.09E-08 | | 2.47E-06 | | Reactome | 7.51 | |
| Peptide ligand-binding receptors | | 199 | | 31 (15.9%) | | 6.55E-08 | | 4.93E-06 | | Reactome | 7.18 | |
| GPCR ligand binding | | 454 | | 51 (11.4%) | | 4.05E-07 | | 2.13E-05 | | Reactome | 6.39 | |
| Degradation of the extracellular matrix | | 84 | | 18 (21.4%) | | 4.58E-07 | | 2.33E-05 | | Reactome | 6.34 | |
| G alpha (i) signalling events | | 243 | | 33 (13.8%) | | 8.08E-07 | | 3.99E-05 | | Reactome | 6.09 | |
| HS-GAG biosynthesis | | 31 | | 10 (32.3%) | | 3.37E-06 | | 0.000137 | | Reactome | 5.47 | |
| Collagen degradation | | 33 | | 10 (30.3%) | | 6.38E-06 | | 0.000229 | | Reactome | 5.2 | |
| **Hemostasis** | | 493 | | 51 (10.4%) | | 6.51E-06 | | 0.000229 | | Reactome | 5.19 | |
| Non-integrin membrane-ECM interactions | | 42 | | 11 (26.2%) | | 1.05E-05 | | 0.000326 | | Reactome | 4.98 | |
| **Interferon Signaling** | | 68 | | 14 (20.6%) | | 1.41E-05 | | 0.000424 | | Reactome | 4.85 | |
| Signaling by Interleukins | | 270 | | 32 (11.9%) | | 2.57E-05 | | 0.000699 | | Reactome | 4.59 | |
| Innate Immune System | | 709 | | 64 (9.1%) | | 2.90E-05 | | 0.000765 | | Reactome | 4.54 | |
| Growth hormone receptor signaling | | 19 | | 7 (36.8%) | | 3.92E-05 | | 0.000954 | | Reactome | 4.41 | |
| HS-GAG degradation | | 21 | | 7 (33.3%) | | 8.23E-05 | | 0.00173 | | Reactome | 4.08 | |
| Cell surface interactions at the vascular wall | | 101 | | 16 (16.0%) | | 9.35E-05 | | 0.00194 | | Reactome | 4.03 | |
| Assembly of collagen fibrils and other multimeric structures | | 44 | | 10 (22.7%) | | 9.92E-05 | | 0.00204 | | Reactome | 4 | |
| Heparan sulfate/heparin (HS-GAG) metabolism | | 54 | | 11 (20.4%) | | 0.000129 | | 0.00256 | | Reactome | 3.89 | |
| Activation of Matrix Metalloproteinases | | 30 | | 8 (26.7%) | | 0.00015 | | 0.00286 | | Reactome | 3.82 | |
| Tryptophan catabolism | | 11 | | 5 (45.5%) | | 0.000166 | | 0.00306 | | Reactome | 3.78 | |
| ISG15 antiviral mechanism | | 31 | | 8 (25.8%) | | 0.000192 | | 0.00338 | | Reactome | 3.72 | |
| Antiviral mechanism by IFN-stimulated genes | | 31 | | 8 (25.8%) | | 0.000192 | | 0.00338 | | Reactome | 3.72 | |
| Gastrin-CREB signalling pathway via PKC and MAPK | | 382 | | 38 (10.0%) | | 0.000222 | | 0.00362 | | Reactome | 3.65 | |
| Collagen formation | | 87 | | 14 (16.1%) | | 0.000238 | | 0.00376 | | Reactome | 3.62 | |
| Syndecan interactions | | 20 | | 6 (30.0%) | | 0.000515 | | 0.00726 | | Reactome | 3.29 | |
| Astrocytic Glutamate-Glutamine Uptake And Metabolism | | 4 | | 3 (75.0%) | | 0.000619 | | 0.00843 | | Reactome | 3.21 | |
| Neurotransmitter uptake and Metabolism In Glial Cells | | 4 | | 3 (75.0%) | | 0.000619 | | 0.00843 | | Reactome | 3.21 | |
| Regulation of IFNG signaling | | 14 | | 5 (35.7%) | | 0.000629 | | 0.00849 | | Reactome | 3.2 | |
| Glycosaminoglycan metabolism | | 119 | | 16 (13.6%) | | 0.000651 | | 0.00872 | | Reactome | 3.19 | |
| SLC-mediated transmembrane transport | | 263 | | 27 (10.3%) | | 0.00108 | | 0.0133 | | Reactome | 2.97 | |
| Immunoregulatory interactions between a Lymphoid and a non-Lymphoid cell | | 146 | | 17 (12.5%) | | 0.00114 | | 0.0139 | | Reactome | 2.94 | |
| Laminin interactions | | 23 | | 6 (26.1%) | | 0.00117 | | 0.0141 | | Reactome | 2.93 | |
| Binding and Uptake of Ligands by Scavenger Receptors | | 40 | | 8 (20.0%) | | 0.00122 | | 0.0146 | | Reactome | 2.91 | |
| Dectin-2 family | | 10 | | 4 (40.0%) | | 0.00141 | | 0.0164 | | Reactome | 2.85 | |
| **Platelet activation, signaling and aggregation** | | 229 | | 24 (10.5%) | | 0.0016 | | 0.0181 | | Reactome | 2.8 | |
| Transport of inorganic cations/anions and amino acids/oligopeptides | | 95 | | 13 (13.7%) | | 0.00187 | | 0.021 | | Reactome | 2.73 | |
| Axon guidance | | 459 | | 40 (8.8%) | | 0.00202 | | 0.0219 | | Reactome | 2.69 | |
| Signaling by NOTCH3 | | 11 | | 4 (36.4%) | | 0.00212 | | 0.0228 | | Reactome | 2.67 | |
| Regulation of IFNA signaling | | 26 | | 6 (23.1%) | | 0.00231 | | 0.0244 | | Reactome | 2.64 | |
| A tetrasaccharide linker sequence is required for GAG synthesis | | 26 | | 6 (23.1%) | | 0.00231 | | 0.0244 | | Reactome | 2.64 | |
| VEGFR2 mediated cell proliferation | | 198 | | 21 (10.7%) | | 0.00248 | | 0.026 | | Reactome | 2.61 | |
| MAPK family signaling cascades | | 225 | | 23 (10.3%) | | 0.00258 | | 0.0266 | | Reactome | 2.59 | |
| Interferon alpha/beta signaling | | 27 | | 6 (22.2%) | | 0.00284 | | 0.0291 | | Reactome | 2.55 | |
| Scavenging by Class A Receptors | | 19 | | 5 (26.3%) | | 0.00291 | | 0.0295 | | Reactome | 2.54 | |
| Interferon gamma signaling | | 19 | | 5 (26.3%) | | 0.00291 | | 0.0295 | | Reactome | 2.54 | |
| MAPK1/MAPK3 signaling | | 191 | | 20 (10.5%) | | 0.0036 | | 0.0353 | | Reactome | 2.44 | |
| Signaling by Leptin | | 193 | | 20 (10.4%) | | 0.00405 | | 0.0389 | | Reactome | 2.39 | |
| **Dissolution of Fibrin Clot** | | 13 | | 4 (30.8%) | | 0.00422 | | 0.039 | | Reactome | 2.37 | |
| Negative regulators of RIG-I/MDA5 signaling | | 21 | | 5 (23.8%) | | 0.00465 | | 0.0422 | | Reactome | 2.33 | |
| Activation of C3 and C5 | | 7 | | 3 (42.9%) | | 0.00479 | | 0.0426 | | Reactome | 2.32 | |
| Anchoring fibril formation | | 7 | | 3 (42.9%) | | 0.00479 | | 0.0426 | | Reactome | 2.32 | |
| Signaling by SCF-KIT | | 264 | | 25 (9.5%) | | 0.0048 | | 0.0426 | | Reactome | 2.32 | |
| NCAM signaling for neurite out-growth | | 223 | | 22 (9.9%) | | 0.00485 | | 0.0428 | | Reactome | 2.31 | |
| Chondroitin sulfate/dermatan sulfate metabolism | | 51 | | 8 (16.0%) | | 0.00526 | | 0.0455 | | Reactome | 2.28 | |
| Interleukin-3, 5 and GM-CSF signaling | | 211 | | 21 (10.0%) | | 0.00527 | | 0.0455 | | Reactome | 2.28 | |
| RAF/MAP kinase cascade | | 185 | | 19 (10.3%) | | 0.00551 | | 0.0456 | | Reactome | 2.26 | |
| SHC1 events in EGFR signaling | | 185 | | 19 (10.3%) | | 0.00551 | | 0.0456 | | Reactome | 2.26 | |
| SOS-mediated signalling | | 185 | | 19 (10.3%) | | 0.00551 | | 0.0456 | | Reactome | 2.26 | |
| GRB2 events in EGFR signaling | | 185 | | 19 (10.3%) | | 0.00551 | | 0.0456 | | Reactome | 2.26 | |
| SHC1 events in ERBB2 signaling | | 185 | | 19 (10.3%) | | 0.00551 | | 0.0456 | | Reactome | 2.26 | |
| SHC1 events in ERBB4 signaling | | 185 | | 19 (10.3%) | | 0.00551 | | 0.0456 | | Reactome | 2.26 | |
| GRB2 events in ERBB2 signaling | | 185 | | 19 (10.3%) | | 0.00551 | | 0.0456 | | Reactome | 2.26 | |
| Receptor-ligand binding initiates the second proteolytic cleavage of Notch receptor | | 14 | | 4 (28.6%) | | 0.00565 | | 0.0463 | | Reactome | 2.25 | |
| FRS-mediated FGFR2 signaling | | 186 | | 19 (10.3%) | | 0.00584 | | 0.0469 | | Reactome | 2.23 | |
| FRS-mediated FGFR1 signaling | | 186 | | 19 (10.3%) | | 0.00584 | | 0.0469 | | Reactome | 2.23 | |
| FRS-mediated FGFR3 signaling | | 186 | | 19 (10.3%) | | 0.00584 | | 0.0469 | | Reactome | 2.23 | |
| FRS-mediated FGFR4 signaling | | 186 | | 19 (10.3%) | | 0.00584 | | 0.0469 | | Reactome | 2.23 | |
| Interleukin-2 signaling | | 202 | | 20 (10.0%) | | 0.00674 | | 0.0533 | | Reactome | 2.17 | |
| Signalling to p38 via RIT and RIN | | 189 | | 19 (10.1%) | | 0.00693 | | 0.0536 | | Reactome | 2.16 | |
| ARMS-mediated activation | | 189 | | 19 (10.1%) | | 0.00693 | | 0.0536 | | Reactome | 2.16 | |
| Adaptive Immune System | | 612 | | 47 (7.9%) | | 0.00702 | | 0.0538 | | Reactome | 2.15 | |
| RAF-independent MAPK1/3 activation | | 23 | | 5 (21.7%) | | 0.00703 | | 0.0538 | | Reactome | 2.15 | |
| Retinoid metabolism and transport | | 42 | | 7 (16.7%) | | 0.00705 | | 0.0538 | | Reactome | 2.15 | |
| Frs2-mediated activation | | 190 | | 19 (10.1%) | | 0.00732 | | 0.0552 | | Reactome | 2.14 | |
| **RIG-I/MDA5 mediated induction of IFN-alpha/beta pathways** | | 53 | | 8 (15.1%) | | 0.00753 | | 0.0556 | | Reactome | 2.12 | |
| Prolonged ERK activation events | | 192 | | 19 (9.9%) | | 0.00817 | | 0.06 | | Reactome | 2.09 | |
| GPVI-mediated activation cascade | | 54 | | 8 (14.8%) | | 0.00843 | | 0.0611 | | Reactome | 2.07 | |
| Interconversion of polyamines | | 3 | | 2 (66.7%) | | 0.00858 | | 0.0616 | | Reactome | 2.07 | |
| Hydroxycarboxylic acid-binding receptors | | 3 | | 2 (66.7%) | | 0.00858 | | 0.0616 | | Reactome | 2.07 | |
| Signalling to RAS | | 194 | | 19 (9.8%) | | 0.00909 | | 0.065 | | Reactome | 2.04 | |
| Integrin cell surface interactions | | 67 | | 9 (13.6%) | | 0.0092 | | 0.0655 | | Reactome | 2.04 | |
| Interleukin receptor SHC signaling | | 195 | | 19 (9.8%) | | 0.00958 | | 0.0679 | | Reactome | 2.02 | |
