# Supplementary material for: An integrative genomics approach identifies novel pathways that influence candidaemia susceptibility
Source: PLoS One. 2017 Jul 20;12(7):e0180824. doi: 10.1371/journal.pone.0180824 (PMC5519064; doi:10.1371/journal.pone.0180824)
Supplement: S5 Table — SNPs rs12491812 and rs1802141 were in strong linkage disequilibrium (LD) with synonymous variants located in CISH and SNPS1 genes respectively. (source: Haploreg http://archive.broadinstitute.org/mammals/haploreg/haploreg.php). (DOCX) [file pone.0180824.s009.docx]

Table S5. Candidaemia-associated SNPs and variants with r^2^>=0.8. SNPs rs12491812 and rs1802141 were in strong linkage disequilibrium (LD) with synonymous variants located in *CISH* and *SNPS1* genes respectively (source: Haploreg <http://archive.broadinstitute.org/mammals/haploreg/haploreg.php>).

| **Locus 1. Query SNP: rs6699706 and variants with r^2^ >= 0.8** | | | | | |  |  |  |  |
| --- | --- | --- | --- | --- | --- | --- | --- | --- | --- |
| **Chromosome** | **Position (hg38)** | **LD** | **LD** | **Variant** | **Reference** | **Altered** | **EUR** | **GENCODE** | **dbSNP** |
|  |  | **(r²)** | **(D')** |  |  |  | **Frequency** | **Genes** | **Functional annotation** |
| 1 | 18989152 | 0.9 | 0.95 | [rs57825564](http://www.broadinstitute.org/mammals/haploreg/detail_v4.1.php?query=&id=rs57825564) | A | G | 0.11 | 32kb 5' of IFFO2 | Intergenic |
| 1 | 18989323 | 0.9 | 0.95 | [rs59569385](http://www.broadinstitute.org/mammals/haploreg/detail_v4.1.php?query=&id=rs59569385) | A | G | 0.11 | 33kb 5' of IFFO2 | Intergenic |
| 1 | 18990505 | 0.91 | 0.96 | [rs59405037](http://www.broadinstitute.org/mammals/haploreg/detail_v4.1.php?query=&id=rs59405037) | T | C | 0.11 | 34kb 5' of IFFO2 | Intergenic |
| 1 | 18990540 | 0.91 | 0.96 | [rs58552530](http://www.broadinstitute.org/mammals/haploreg/detail_v4.1.php?query=&id=rs58552530) | G | T | 0.11 | 34kb 5' of IFFO2 | Intergenic |
| 1 | 18990607 | 0.9 | 0.96 | [rs57735998](http://www.broadinstitute.org/mammals/haploreg/detail_v4.1.php?query=&id=rs57735998) | A | G | 0.12 | 34kb 5' of IFFO2 | Intergenic |
| 1 | 18990844 | 0.91 | 0.96 | [rs28789190](http://www.broadinstitute.org/mammals/haploreg/detail_v4.1.php?query=&id=rs28789190) | G | A | 0.11 | 34kb 5' of IFFO2 | Intergenic |
| 1 | 18991035 | 0.91 | 0.96 | [rs6669987](http://www.broadinstitute.org/mammals/haploreg/detail_v4.1.php?query=&id=rs6669987) | G | A | 0.11 | 34kb 5' of IFFO2 | Intergenic |
| 1 | 18991493 | 0.91 | 0.96 | [rs7527267](http://www.broadinstitute.org/mammals/haploreg/detail_v4.1.php?query=&id=rs7527267) | G | T | 0.11 | 35kb 5' of IFFO2 | Intergenic |
| 1 | 18991593 | 0.91 | 0.96 | [rs7540747](http://www.broadinstitute.org/mammals/haploreg/detail_v4.1.php?query=&id=rs7540747) | A | G | 0.11 | 35kb 5' of IFFO2 | Intergenic |
| 1 | 18991819 | 0.89 | 0.96 | [rs6682824](http://www.broadinstitute.org/mammals/haploreg/detail_v4.1.php?query=&id=rs6682824) | C | G | 0.12 | 35kb 5' of IFFO2 | Intergenic |
| 1 | 18992603 | 0.92 | 0.96 | [rs7539506](http://www.broadinstitute.org/mammals/haploreg/detail_v4.1.php?query=&id=rs7539506) | C | A | 0.11 | 36kb 5' of IFFO2 | Intergenic |
| 1 | 18992627 | 0.92 | 0.96 | [rs7543956](http://www.broadinstitute.org/mammals/haploreg/detail_v4.1.php?query=&id=rs7543956) | A | T | 0.11 | 36kb 5' of IFFO2 | Intergenic |
| 1 | 18993125 | 0.92 | 0.96 | [rs6694309](http://www.broadinstitute.org/mammals/haploreg/detail_v4.1.php?query=&id=rs6694309) | T | C | 0.11 | 36kb 5' of IFFO2 | Intergenic |
| 1 | 18993715 | 0.92 | 0.96 | [rs6697292](http://www.broadinstitute.org/mammals/haploreg/detail_v4.1.php?query=&id=rs6697292) | T | G | 0.11 | 37kb 5' of IFFO2 | Intergenic |
| 1 | 18993825 | 0.92 | 0.96 | [rs6689626](http://www.broadinstitute.org/mammals/haploreg/detail_v4.1.php?query=&id=rs6689626) | C | A | 0.11 | 37kb 5' of IFFO2 | Intergenic |
| 1 | 18993937 | 0.92 | 0.96 | [rs6694529](http://www.broadinstitute.org/mammals/haploreg/detail_v4.1.php?query=&id=rs6694529) | A | G | 0.11 | 37kb 5' of IFFO2 | Intergenic |
| 1 | 18994050 | 0.92 | 0.96 | [rs6689820](http://www.broadinstitute.org/mammals/haploreg/detail_v4.1.php?query=&id=rs6689820) | C | A | 0.11 | 37kb 5' of IFFO2 | Intergenic |
| 1 | 18994344 | 0.92 | 0.96 | [rs28623160](http://www.broadinstitute.org/mammals/haploreg/detail_v4.1.php?query=&id=rs28623160) | T | C | 0.11 | 38kb 5' of IFFO2 | Intergenic |
| 1 | 18994421 | 0.92 | 0.96 | [rs28575256](http://www.broadinstitute.org/mammals/haploreg/detail_v4.1.php?query=&id=rs28575256) | C | T | 0.11 | 38kb 5' of IFFO2 | Intergenic |
| 1 | 18994571 | 0.92 | 0.96 | [rs28651004](http://www.broadinstitute.org/mammals/haploreg/detail_v4.1.php?query=&id=rs28651004) | A | G | 0.11 | 38kb 5' of IFFO2 | Intergenic |
| 1 | 18995310 | 0.95 | 0.97 | [rs28369012](http://www.broadinstitute.org/mammals/haploreg/detail_v4.1.php?query=&id=rs28369012) | A | C | 0.11 | 39kb 5' of IFFO2 | Intergenic |
| 1 | 18995575 | 0.95 | 0.97 | [rs56153612](http://www.broadinstitute.org/mammals/haploreg/detail_v4.1.php?query=&id=rs56153612) | C | T | 0.11 | 39kb 5' of IFFO2 | Intergenic |
| 1 | 18995992 | 0.95 | 0.97 | [rs4611046](http://www.broadinstitute.org/mammals/haploreg/detail_v4.1.php?query=&id=rs4611046) | A | G | 0.11 | 39kb 5' of IFFO2 | Intergenic |
| 1 | 18996055 | 0.92 | 0.97 | [rs1570861](http://www.broadinstitute.org/mammals/haploreg/detail_v4.1.php?query=&id=rs1570861) | G | A | 0.12 | 39kb 5' of IFFO2 | Intergenic |
| 1 | 18996157 | 0.94 | 0.97 | [rs1570860](http://www.broadinstitute.org/mammals/haploreg/detail_v4.1.php?query=&id=rs1570860) | T | C | 0.11 | 39kb 5' of IFFO2 | Intergenic |
| 1 | 18996556 | 0.94 | 0.97 | [rs6690097](http://www.broadinstitute.org/mammals/haploreg/detail_v4.1.php?query=&id=rs6690097) | G | C | 0.11 | 40kb 5' of IFFO2 | Intergenic |
| 1 | 18996839 | 0.94 | 0.97 | [rs6659084](http://www.broadinstitute.org/mammals/haploreg/detail_v4.1.php?query=&id=rs6659084) | T | C | 0.11 | 40kb 5' of IFFO2 | Intergenic |
| 1 | 18996887 | 0.94 | 0.97 | [rs6690430](http://www.broadinstitute.org/mammals/haploreg/detail_v4.1.php?query=&id=rs6690430) | G | T | 0.11 | 40kb 5' of IFFO2 | Intergenic |
| 1 | 18997108 | 0.9 | 0.95 | [rs1316768](http://www.broadinstitute.org/mammals/haploreg/detail_v4.1.php?query=&id=rs1316768) | G | A | 0.11 | 40kb 5' of IFFO2 | Intergenic |
| 1 | 18997598 | 0.9 | 0.96 | [rs6703129](http://www.broadinstitute.org/mammals/haploreg/detail_v4.1.php?query=&id=rs6703129) | C | T | 0.12 | 41kb 5' of IFFO2 | Intergenic |
| 1 | 18997744 | 0.94 | 0.97 | [rs28690197](http://www.broadinstitute.org/mammals/haploreg/detail_v4.1.php?query=&id=rs28690197) | C | G | 0.11 | 41kb 5' of IFFO2 | Intergenic |
| 1 | 18997899 | 0.94 | 0.97 | [rs28417827](http://www.broadinstitute.org/mammals/haploreg/detail_v4.1.php?query=&id=rs28417827) | G | A | 0.11 | 41kb 5' of IFFO2 | Intergenic |
| 1 | 18997917 | 0.94 | 0.97 | [rs28686865](http://www.broadinstitute.org/mammals/haploreg/detail_v4.1.php?query=&id=rs28686865) | G | A | 0.11 | 41kb 5' of IFFO2 | Intergenic |
| 1 | 18998168 | 0.94 | 0.97 | [rs28431045](http://www.broadinstitute.org/mammals/haploreg/detail_v4.1.php?query=&id=rs28431045) | T | C | 0.11 | 41kb 5' of IFFO2 | Intergenic |
| 1 | 18998191 | 0.94 | 0.97 | [rs28429352](http://www.broadinstitute.org/mammals/haploreg/detail_v4.1.php?query=&id=rs28429352) | C | A | 0.11 | 42kb 5' of IFFO2 | Intergenic |
| 1 | 18998227 | 0.91 | 0.96 | [rs28366691](http://www.broadinstitute.org/mammals/haploreg/detail_v4.1.php?query=&id=rs28366691) | G | A | 0.11 | 42kb 5' of IFFO2 | Intergenic |
| 1 | 18998229 | 0.94 | 0.97 | [rs28628464](http://www.broadinstitute.org/mammals/haploreg/detail_v4.1.php?query=&id=rs28628464) | C | T | 0.11 | 42kb 5' of IFFO2 | Intergenic |
| 1 | 18998590 | 0.94 | 0.97 | [rs28715564](http://www.broadinstitute.org/mammals/haploreg/detail_v4.1.php?query=&id=rs28715564) | A | G | 0.11 | 42kb 5' of IFFO2 | Intergenic |
| 1 | 18999277 | 0.97 | 1 | [rs6700076](http://www.broadinstitute.org/mammals/haploreg/detail_v4.1.php?query=&id=rs6700076) | G | C | 0.11 | 43kb 5' of IFFO2 | Intergenic |
| 1 | 18999422 | 0.97 | 0.99 | [rs6666106](http://www.broadinstitute.org/mammals/haploreg/detail_v4.1.php?query=&id=rs6666106) | A | G | 0.11 | 43kb 5' of IFFO2 | Intergenic |
| 1 | 18999740 | 0.95 | 0.99 | [rs6669421](http://www.broadinstitute.org/mammals/haploreg/detail_v4.1.php?query=&id=rs6669421) | T | A | 0.12 | 43kb 5' of IFFO2 | Intergenic |
| 1 | 18999819 | 0.97 | 0.99 | [rs6700619](http://www.broadinstitute.org/mammals/haploreg/detail_v4.1.php?query=&id=rs6700619) | G | C | 0.11 | 43kb 5' of IFFO2 | Intergenic |
| 1 | 19000277 | 1 | 1 | [rs28398771](http://www.broadinstitute.org/mammals/haploreg/detail_v4.1.php?query=&id=rs28398771) | A | G | 0.11 | 44kb 5' of IFFO2 | Intergenic |
| 1 | 19000319 | 1 | 1 | [rs6699706](http://www.broadinstitute.org/mammals/haploreg/detail_v4.1.php?query=&id=rs6699706) | A | C | 0.11 | 44kb 5' of IFFO2 | Intergenic |
| 1 | 19000888 | 1 | 1 | [rs6700354](http://www.broadinstitute.org/mammals/haploreg/detail_v4.1.php?query=&id=rs6700354) | A | G | 0.11 | 44kb 5' of IFFO2 | Intergenic |
| 1 | 19001112 | 0.81 | 1 | [rs6703643](http://www.broadinstitute.org/mammals/haploreg/detail_v4.1.php?query=&id=rs6703643) | T | G | 0.09 | 44kb 5' of IFFO2 | Intergenic |
| 1 | 19001271 | 1 | 1 | [rs72951502](http://www.broadinstitute.org/mammals/haploreg/detail_v4.1.php?query=&id=rs72951502) | C | T | 0.11 | 45kb 5' of IFFO2 | Intergenic |
| 1 | 19001288 | 1 | 1 | [rs28382321](http://www.broadinstitute.org/mammals/haploreg/detail_v4.1.php?query=&id=rs28382321) | G | A | 0.11 | 45kb 5' of IFFO2 | Intergenic |
| 1 | 19001345 | 1 | 1 | [rs28480631](http://www.broadinstitute.org/mammals/haploreg/detail_v4.1.php?query=&id=rs28480631) | T | C | 0.11 | 45kb 5' of IFFO2 | Intergenic |
| 1 | 19001583 | 1 | 1 | [rs28806527](http://www.broadinstitute.org/mammals/haploreg/detail_v4.1.php?query=&id=rs28806527) | T | C | 0.11 | 45kb 5' of IFFO2 | Intergenic |
| 1 | 19001748 | 1 | 1 | [rs28840430](http://www.broadinstitute.org/mammals/haploreg/detail_v4.1.php?query=&id=rs28840430) | T | C | 0.11 | 45kb 5' of IFFO2 | Intergenic |
| 1 | 19001964 | 1 | 1 | [rs28505299](http://www.broadinstitute.org/mammals/haploreg/detail_v4.1.php?query=&id=rs28505299) | G | T | 0.11 | 45kb 5' of IFFO2 | Intergenic |
| 1 | 19001966 | 1 | 1 | [rs28633203](http://www.broadinstitute.org/mammals/haploreg/detail_v4.1.php?query=&id=rs28633203) | A | G | 0.11 | 45kb 5' of IFFO2 | Intergenic |
| 1 | 19002512 | 1 | 1 | [rs6701963](http://www.broadinstitute.org/mammals/haploreg/detail_v4.1.php?query=&id=rs6701963) | C | G | 0.11 | 46kb 5' of IFFO2 | Intergenic |
| 1 | 19003051 | 1 | 1 | [rs6702530](http://www.broadinstitute.org/mammals/haploreg/detail_v4.1.php?query=&id=rs6702530) | C | T | 0.11 | 46kb 5' of IFFO2 | Intergenic |
| 1 | 19003218 | 1 | 1 | [rs28505711](http://www.broadinstitute.org/mammals/haploreg/detail_v4.1.php?query=&id=rs28505711) | G | A | 0.11 | 47kb 5' of IFFO2 | Intergenic |
| 1 | 19004562 | 0.97 | 1 | [rs2012808](http://www.broadinstitute.org/mammals/haploreg/detail_v4.1.php?query=&id=rs2012808) | A | G | 0.12 | 48kb 5' of IFFO2 | Intergenic |
| 1 | 19004650 | 1 | 1 | [rs6699361](http://www.broadinstitute.org/mammals/haploreg/detail_v4.1.php?query=&id=rs6699361) | G | T | 0.11 | 48kb 5' of IFFO2 | Intergenic |
| 1 | 19004914 | 1 | 1 | [rs1078895](http://www.broadinstitute.org/mammals/haploreg/detail_v4.1.php?query=&id=rs1078895) | T | C | 0.11 | 48kb 5' of IFFO2 | Intergenic |
| 1 | 19005103 | 1 | 1 | [rs1078893](http://www.broadinstitute.org/mammals/haploreg/detail_v4.1.php?query=&id=rs1078893) | T | A | 0.11 | 48kb 5' of IFFO2 | Intergenic |
| 1 | 19005397 | 1 | 1 | [rs6426622](http://www.broadinstitute.org/mammals/haploreg/detail_v4.1.php?query=&id=rs6426622) | T | C | 0.11 | 49kb 5' of IFFO2 | Intergenic |
| 1 | 19005582 | 0.99 | 1 | [rs7523455](http://www.broadinstitute.org/mammals/haploreg/detail_v4.1.php?query=&id=rs7523455) | A | G | 0.11 | 49kb 5' of IFFO2 | Intergenic |
| 1 | 19006097 | 0.96 | 0.99 | [rs16862451](http://www.broadinstitute.org/mammals/haploreg/detail_v4.1.php?query=&id=rs16862451) | C | T | 0.11 | 49kb 5' of IFFO2 | Intergenic |
| 1 | 19006231 | 0.96 | 0.99 | [rs28675749](http://www.broadinstitute.org/mammals/haploreg/detail_v4.1.php?query=&id=rs28675749) | C | T | 0.11 | 50kb 5' of IFFO2 | Intergenic |
| 1 | 19006280 | 0.96 | 0.99 | [rs16862452](http://www.broadinstitute.org/mammals/haploreg/detail_v4.1.php?query=&id=rs16862452) | T | C | 0.11 | 50kb 5' of IFFO2 | Intergenic |
| **Locus 2. Query SNP: rs11102637 and variants with r^2^ >= 0.8** | | | | | |  |  |  |  |
| 1 | 113405301 | 0.8 | 0.9 | [rs1418607](http://www.broadinstitute.org/mammals/haploreg/detail_v4.1.php?query=&id=rs1418607) | C | T | 0.18 | MAGI3 | Intronic |
| 1 | 113405986 | 0.8 | 0.9 | [rs2027537](http://www.broadinstitute.org/mammals/haploreg/detail_v4.1.php?query=&id=rs2027537) | C | G,T | 0.18 | MAGI3 | Intronic |
| 1 | 113406706 | 0.8 | 0.9 | [rs12021783](http://www.broadinstitute.org/mammals/haploreg/detail_v4.1.php?query=&id=rs12021783) | A | C | 0.18 | MAGI3 | Intronic |
| 1 | 113408485 | 0.8 | 0.9 | [rs12023641](http://www.broadinstitute.org/mammals/haploreg/detail_v4.1.php?query=&id=rs12023641) | A | C | 0.18 | MAGI3 | Intronic |
| 1 | 113410914 | 0.8 | 0.9 | [rs55737954](http://www.broadinstitute.org/mammals/haploreg/detail_v4.1.php?query=&id=rs55737954) | G | A | 0.18 | MAGI3 | Intronic |
| 1 | 113424319 | 0.82 | 0.91 | [rs10458458](http://www.broadinstitute.org/mammals/haploreg/detail_v4.1.php?query=&id=rs10458458) | G | C | 0.18 | MAGI3 | Intronic |
| 1 | 113424783 | 0.83 | 0.91 | [rs10458459](http://www.broadinstitute.org/mammals/haploreg/detail_v4.1.php?query=&id=rs10458459) | C | T | 0.18 | MAGI3 | Intronic |
| 1 | 113425052 | 0.81 | 0.91 | [rs12029305](http://www.broadinstitute.org/mammals/haploreg/detail_v4.1.php?query=&id=rs12029305) | A | G | 0.18 | MAGI3 | Intronic |
| 1 | 113428152 | 0.83 | 0.91 | [rs12032395](http://www.broadinstitute.org/mammals/haploreg/detail_v4.1.php?query=&id=rs12032395) | A | G | 0.18 | MAGI3 | Intronic |
| 1 | 113429925 | 0.82 | 0.91 | [rs72687924](http://www.broadinstitute.org/mammals/haploreg/detail_v4.1.php?query=&id=rs72687924) | G | T | 0.18 | MAGI3 | Intronic |
| 1 | 113431641 | 0.82 | 0.91 | [rs12030900](http://www.broadinstitute.org/mammals/haploreg/detail_v4.1.php?query=&id=rs12030900) | A | G | 0.18 | MAGI3 | Intronic |
| 1 | 113431937 | 0.82 | 0.91 | [rs12034178](http://www.broadinstitute.org/mammals/haploreg/detail_v4.1.php?query=&id=rs12034178) | G | T | 0.18 | MAGI3 | Intronic |
| 1 | 113436043 | 0.82 | 0.91 | [rs35818543](http://www.broadinstitute.org/mammals/haploreg/detail_v4.1.php?query=&id=rs35818543) | CAT | C | 0.18 | MAGI3 | Intronic |
| 1 | 113437599 | 0.82 | 0.91 | [rs5777159](http://www.broadinstitute.org/mammals/haploreg/detail_v4.1.php?query=&id=rs5777159) | TA | T | 0.18 | MAGI3 | Intronic |
| 1 | 113440005 | 1 | 1 | [rs12039669](http://www.broadinstitute.org/mammals/haploreg/detail_v4.1.php?query=&id=rs12039669) | T | G | 0.18 | MAGI3 | Intronic |
| 1 | 113441032 | 1 | 1 | [rs12022213](http://www.broadinstitute.org/mammals/haploreg/detail_v4.1.php?query=&id=rs12022213) | C | T | 0.18 | MAGI3 | Intronic |
| 1 | 113441121 | 1 | 1 | [rs12039944](http://www.broadinstitute.org/mammals/haploreg/detail_v4.1.php?query=&id=rs12039944) | A | G | 0.18 | MAGI3 | Intronic |
| 1 | 113441435 | 1 | 1 | [rs1936928](http://www.broadinstitute.org/mammals/haploreg/detail_v4.1.php?query=&id=rs1936928) | G | A | 0.18 | MAGI3 | Intronic |
| 1 | 113441608 | 1 | 1 | [rs1936927](http://www.broadinstitute.org/mammals/haploreg/detail_v4.1.php?query=&id=rs1936927) | A | G | 0.18 | MAGI3 | Intronic |
| 1 | 113442834 | 0.99 | 1 | [rs1573996](http://www.broadinstitute.org/mammals/haploreg/detail_v4.1.php?query=&id=rs1573996) | A | G | 0.18 | MAGI3 | Intronic |
| 1 | 113446628 | 1 | 1 | [rs11102636](http://www.broadinstitute.org/mammals/haploreg/detail_v4.1.php?query=&id=rs11102636) | A | G | 0.18 | MAGI3 | Intronic |
| 1 | 113447427 | 0.99 | 1 | [rs12043827](http://www.broadinstitute.org/mammals/haploreg/detail_v4.1.php?query=&id=rs12043827) | G | A | 0.18 | MAGI3 | Intronic |
| 1 | 113449170 | 1 | 1 | [rs11102637](http://www.broadinstitute.org/mammals/haploreg/detail_v4.1.php?query=&id=rs11102637) | G | A | 0.18 | MAGI3 | Intronic |
| 1 | 113449853 | 1 | 1 | [rs12025806](http://www.broadinstitute.org/mammals/haploreg/detail_v4.1.php?query=&id=rs12025806) | C | T | 0.18 | MAGI3 | Intronic |
| 1 | 113454244 | 0.99 | 1 | [rs12045559](http://www.broadinstitute.org/mammals/haploreg/detail_v4.1.php?query=&id=rs12045559) | G | T | 0.18 | MAGI3 | Intronic |
| 1 | 113454850 | 1 | 1 | [rs12046450](http://www.broadinstitute.org/mammals/haploreg/detail_v4.1.php?query=&id=rs12046450) | G | A | 0.18 | MAGI3 | Intronic |
| 1 | 113455064 | 1 | 1 | [rs11102638](http://www.broadinstitute.org/mammals/haploreg/detail_v4.1.php?query=&id=rs11102638) | T | C | 0.18 | MAGI3 | Intronic |
| 1 | 113464525 | 1 | 1 | [rs11102640](http://www.broadinstitute.org/mammals/haploreg/detail_v4.1.php?query=&id=rs11102640) | G | T | 0.18 | MAGI3 | Intronic |
| 1 | 113465662 | 1 | 1 | [rs11102641](http://www.broadinstitute.org/mammals/haploreg/detail_v4.1.php?query=&id=rs11102641) | A | G | 0.18 | MAGI3 | Intronic |
| 1 | 113468315 | 1 | 1 | [rs72687938](http://www.broadinstitute.org/mammals/haploreg/detail_v4.1.php?query=&id=rs72687938) | T | G | 0.18 | MAGI3 | Intronic |
| 1 | 113468684 | 1 | 1 | [rs72687939](http://www.broadinstitute.org/mammals/haploreg/detail_v4.1.php?query=&id=rs72687939) | G | A | 0.18 | MAGI3 | Intronic |
| 1 | 113473636 | 1 | 1 | [rs78995277](http://www.broadinstitute.org/mammals/haploreg/detail_v4.1.php?query=&id=rs78995277) | T | C | 0.18 | MAGI3 | Intronic |
|  |  | 0.96 | 0.98 | [rs201975600](http://www.broadinstitute.org/mammals/haploreg/detail_v4.1.php?query=&id=rs201975600) | TTC | T | 0.18 | MAGI3 | Intronic |
| 1 | 113476614 | 1 | 1 | [rs148488077](http://www.broadinstitute.org/mammals/haploreg/detail_v4.1.php?query=&id=rs148488077) | C | T | 0.18 | MAGI3 | Intronic |
| 1 | 113477369 | 1 | 1 | [rs7413018](http://www.broadinstitute.org/mammals/haploreg/detail_v4.1.php?query=&id=rs7413018) | T | G | 0.18 | MAGI3 | Intronic |
| 1 | 113480445 | 1 | 1 | [rs74465571](http://www.broadinstitute.org/mammals/haploreg/detail_v4.1.php?query=&id=rs74465571) | C | T | 0.18 | MAGI3 | Intronic |
| 1 | 113480717 | 1 | 1 | [rs72687944](http://www.broadinstitute.org/mammals/haploreg/detail_v4.1.php?query=&id=rs72687944) | C | T | 0.18 | MAGI3 | Intronic |
| 1 | 113481726 | 1 | 1 | [rs12038473](http://www.broadinstitute.org/mammals/haploreg/detail_v4.1.php?query=&id=rs12038473) | C | T | 0.18 | MAGI3 | Intronic |
| 1 | 113482047 | 1 | 1 | [rs11102644](http://www.broadinstitute.org/mammals/haploreg/detail_v4.1.php?query=&id=rs11102644) | A | G | 0.18 | MAGI3 | Intronic |
| 1 | 113484093 | 1 | 1 | [rs72687947](http://www.broadinstitute.org/mammals/haploreg/detail_v4.1.php?query=&id=rs72687947) | G | T | 0.18 | MAGI3 | Intronic |
| 1 | 113484144 | 1 | 1 | [rs72687948](http://www.broadinstitute.org/mammals/haploreg/detail_v4.1.php?query=&id=rs72687948) | A | C | 0.18 | MAGI3 | Intronic |
| 1 | 113484858 | 1 | 1 | [rs72687949](http://www.broadinstitute.org/mammals/haploreg/detail_v4.1.php?query=&id=rs72687949) | G | A | 0.18 | MAGI3 | Intronic |
| 1 | 113488304 | 1 | 1 | [rs12044485](http://www.broadinstitute.org/mammals/haploreg/detail_v4.1.php?query=&id=rs12044485) | C | T | 0.18 | MAGI3 | Intronic |
| 1 | 113489889 | 1 | 1 | [rs12039226](http://www.broadinstitute.org/mammals/haploreg/detail_v4.1.php?query=&id=rs12039226) | G | A | 0.18 | MAGI3 | Intronic |
| 1 | 113492466 | 1 | 1 | [rs12033666](http://www.broadinstitute.org/mammals/haploreg/detail_v4.1.php?query=&id=rs12033666) | A | G | 0.18 | MAGI3 | Intronic |
| 1 | 113493543 | 1 | 1 | [rs1936926](http://www.broadinstitute.org/mammals/haploreg/detail_v4.1.php?query=&id=rs1936926) | G | A | 0.18 | MAGI3 | Intronic |
| 1 | 113507469 | 0.95 | 0.97 | [rs12046451](http://www.broadinstitute.org/mammals/haploreg/detail_v4.1.php?query=&id=rs12046451) | G | A | 0.18 | MAGI3 | Intronic |
| 1 | 113513195 | 0.91 | 0.95 | [rs74988614](http://www.broadinstitute.org/mammals/haploreg/detail_v4.1.php?query=&id=rs74988614) | T | G | 0.18 | MAGI3 | Intronic |
| 1 | 113513394 | 0.82 | 0.95 | [rs12046434](http://www.broadinstitute.org/mammals/haploreg/detail_v4.1.php?query=&id=rs12046434) | G | A | 0.16 | MAGI3 | Intronic |
| 1 | 113515327 | 0.91 | 0.95 | [rs12045148](http://www.broadinstitute.org/mammals/haploreg/detail_v4.1.php?query=&id=rs12045148) | A | T | 0.18 | MAGI3 | Intronic |
| 1 | 113515658 | 0.89 | 0.95 | [rs4838988](http://www.broadinstitute.org/mammals/haploreg/detail_v4.1.php?query=&id=rs4838988) | C | T | 0.18 | MAGI3 | Intronic |
| 1 | 113519247 | 0.89 | 0.95 | [rs12024139](http://www.broadinstitute.org/mammals/haploreg/detail_v4.1.php?query=&id=rs12024139) | G | T | 0.18 | MAGI3 | Intronic |
| 1 | 113520568 | 0.87 | 0.95 | [rs11102645](http://www.broadinstitute.org/mammals/haploreg/detail_v4.1.php?query=&id=rs11102645) | T | A | 0.18 | MAGI3 | Intronic |
| 1 | 113520901 | 0.89 | 0.95 | [rs12022849](http://www.broadinstitute.org/mammals/haploreg/detail_v4.1.php?query=&id=rs12022849) | A | G | 0.18 | MAGI3 | Intronic |
| 1 | 113522168 | 0.89 | 0.95 | [rs2884705](http://www.broadinstitute.org/mammals/haploreg/detail_v4.1.php?query=&id=rs2884705) | C | T | 0.18 | MAGI3 | Intronic |
| 1 | 113522276 | 0.88 | 0.95 | [rs2359413](http://www.broadinstitute.org/mammals/haploreg/detail_v4.1.php?query=&id=rs2359413) | T | G | 0.18 | MAGI3 | Intronic |
| 1 | 113522673 | 0.89 | 0.95 | [rs72687960](http://www.broadinstitute.org/mammals/haploreg/detail_v4.1.php?query=&id=rs72687960) | T | A | 0.18 | MAGI3 | Intronic |
| 1 | 113522718 | 0.89 | 0.95 | [rs72687961](http://www.broadinstitute.org/mammals/haploreg/detail_v4.1.php?query=&id=rs72687961) | C | T | 0.18 | MAGI3 | Intronic |
| 1 | 113525957 | 0.89 | 0.95 | [rs2051083](http://www.broadinstitute.org/mammals/haploreg/detail_v4.1.php?query=&id=rs2051083) | G | T | 0.18 | MAGI3 | Intronic |
| 1 | 113526463 | 0.89 | 0.95 | [rs12026682](http://www.broadinstitute.org/mammals/haploreg/detail_v4.1.php?query=&id=rs12026682) | G | A | 0.18 | MAGI3 | Intronic |
| 1 | 113528995 | 0.85 | 0.94 | [rs4839324](http://www.broadinstitute.org/mammals/haploreg/detail_v4.1.php?query=&id=rs4839324) | G | A | 0.17 | MAGI3 | Intronic |
| **Locus 3. Query SNP: rs3766122 and variants with r^2^ >= 0.8** | | | | | |  |  |  |  |
| 1 | 169555933 | 0.86 | 0.97 | [rs9332581](http://www.broadinstitute.org/mammals/haploreg/detail_v4.1.php?query=&id=rs9332581) | G | T | 0.05 | F5 | Intronic |
| 1 | 169556011 | 0.86 | 0.97 | [rs9332580](http://www.broadinstitute.org/mammals/haploreg/detail_v4.1.php?query=&id=rs9332580) | A | G | 0.05 | F5 | Intronic |
| 1 | 169566680 | 0.91 | 0.98 | [rs12024736](http://www.broadinstitute.org/mammals/haploreg/detail_v4.1.php?query=&id=rs12024736) | A | G | 0.06 | F5 | Intronic |
| 1 | 169591019 | 0.95 | 1 | [rs3917843](http://www.broadinstitute.org/mammals/haploreg/detail_v4.1.php?query=&id=rs3917843) | C | T | 0.06 | SELP | Intronic |
| 1 | 169597589 | 1 | 1 | [rs3766122](http://www.broadinstitute.org/mammals/haploreg/detail_v4.1.php?query=&id=rs3766122) | T | C | 0.06 | SELP | Intronic |
| **Locus 4. Query SNP: rs296537 and variants with r^2^ >= 0.8** | | | | | |  |  |  |  |
| 1 | 200919286 | 1 | -1 | [rs78154166](http://www.broadinstitute.org/mammals/haploreg/detail_v4.1.php?query=&id=rs78154166) | G | A | 0.01 | 3.6kb 3' of C1orf106 | Intergenic |
| 1 | 200920121 | 1 | -1 | [rs139023910](http://www.broadinstitute.org/mammals/haploreg/detail_v4.1.php?query=&id=rs139023910) | A | T | 0.01 | 4.4kb 3' of C1orf106 | Intergenic |
| 1 | 200921348 | 1 | -1 | [rs59233488](http://www.broadinstitute.org/mammals/haploreg/detail_v4.1.php?query=&id=rs59233488) | C | G | 0.01 | 5.6kb 3' of C1orf106 | Intergenic |
| 1 | 200925926 | 1 | -1 | [rs113661670](http://www.broadinstitute.org/mammals/haploreg/detail_v4.1.php?query=&id=rs113661670) | A | G | 0.01 | 7.4kb 3' of U6 | Intergenic |
| 1 | 200931047 | 1 | -1 | [rs143611860](http://www.broadinstitute.org/mammals/haploreg/detail_v4.1.php?query=&id=rs143611860) | G | C | 0.01 | 2.3kb 3' of U6 | Intronic |
| 1 | 200935921 | 1 | -1 | [rs13374187](http://www.broadinstitute.org/mammals/haploreg/detail_v4.1.php?query=&id=rs13374187) | A | G | 0.01 | 2.5kb 5' of U6 | Intronic |
| 1 | 200936507 | 1 | -1 | [rs113689739](http://www.broadinstitute.org/mammals/haploreg/detail_v4.1.php?query=&id=rs113689739) | A | T | 0.01 | 3.1kb 5' of U6 | Intronic |
| 1 | 200936759 | 1 | -1 | [rs7525822](http://www.broadinstitute.org/mammals/haploreg/detail_v4.1.php?query=&id=rs7525822) | A | G | 0.01 | 3.3kb 5' of U6 | Intronic |
| 1 | 200937554 | 1 | -1 | [rs146936361](http://www.broadinstitute.org/mammals/haploreg/detail_v4.1.php?query=&id=rs146936361) | G | A | 0.01 | 4.1kb 5' of U6 | Intronic |
| 1 | 200942108 | 1 | -1 | [rs145480224](http://www.broadinstitute.org/mammals/haploreg/detail_v4.1.php?query=&id=rs145480224) | G | C | 0.01 | 8.7kb 5' of U6 | Intronic |
| 1 | 200942917 | 1 | -1 | [rs10920073](http://www.broadinstitute.org/mammals/haploreg/detail_v4.1.php?query=&id=rs10920073) | A | G | 0.01 | 9.5kb 5' of U6 | Intronic |
| 1 | 200944808 | 1 | -1 | [rs10920075](http://www.broadinstitute.org/mammals/haploreg/detail_v4.1.php?query=&id=rs10920075) | T | A | 0.01 | 11kb 5' of U6 | Intronic |
| 1 | 200948002 | 1 | 1 | [rs296537](http://www.broadinstitute.org/mammals/haploreg/detail_v4.1.php?query=&id=rs296537) | A | G | 0.99 | 15kb 5' of U6 | Intronic |
| 1 | 200948169 | 1 | -1 | [rs10920077](http://www.broadinstitute.org/mammals/haploreg/detail_v4.1.php?query=&id=rs10920077) | G | A | 0.01 | 15kb 5' of U6 | Intergenic |
| 1 | 200949719 | 1 | -1 | [rs10920078](http://www.broadinstitute.org/mammals/haploreg/detail_v4.1.php?query=&id=rs10920078) | C | T | 0.01 | 16kb 5' of U6 | Intronic |
| 1 | 200950832 | 1 | -1 | [rs80051606](http://www.broadinstitute.org/mammals/haploreg/detail_v4.1.php?query=&id=rs80051606) | A | C | 0.01 | 17kb 5' of U6 | Intronic |
| 1 | 200952049 | 1 | -1 | [rs111635100](http://www.broadinstitute.org/mammals/haploreg/detail_v4.1.php?query=&id=rs111635100) | C | T | 0.01 | 17kb 3' of KIF21B | Intronic |
| 1 | 200952267 | 1 | -1 | [rs28445895](http://www.broadinstitute.org/mammals/haploreg/detail_v4.1.php?query=&id=rs28445895) | C | A | 0.01 | 17kb 3' of KIF21B | Intronic |
| 1 | 200952668 | 1 | -1 | [rs12079204](http://www.broadinstitute.org/mammals/haploreg/detail_v4.1.php?query=&id=rs12079204) | A | G | 0.01 | 17kb 3' of KIF21B | Intronic |
| 1 | 200952817 | 1 | -1 | [rs12074541](http://www.broadinstitute.org/mammals/haploreg/detail_v4.1.php?query=&id=rs12074541) | T | C | 0.01 | 17kb 3' of KIF21B | Intronic |
| 1 | 200958620 | 0.83 | -1 | [rs16847413](http://www.broadinstitute.org/mammals/haploreg/detail_v4.1.php?query=&id=rs16847413) | G | A | 0.01 | 11kb 3' of KIF21B | Intronic |
| 1 | 200959658 | 0.83 | -1 | [rs7554653](http://www.broadinstitute.org/mammals/haploreg/detail_v4.1.php?query=&id=rs7554653) | T | C | 0.01 | 9.7kb 3' of KIF21B | Intronic |
| 1 | 201022257 | 1 | -1 | [rs139357431](http://www.broadinstitute.org/mammals/haploreg/detail_v4.1.php?query=&id=rs139357431) | G | A | 0.01 | KIF21B | Intronic |
|  |  |  |  |  |  |  |  |  |  |
| **Locus 5. Query SNP: rs6748999 and variants with r^2^ >= 0.8** | | | | | |  |  |  |  |
| 2 | 127447412 | 0.81 | -0.9 | [rs6745666](http://www.broadinstitute.org/mammals/haploreg/detail_v4.1.php?query=&id=rs6745666) | C | G | 0.96 | IWS1 | Intergenic |
| 2 | 127447787 | 0.81 | -0.9 | [rs6716856](http://www.broadinstitute.org/mammals/haploreg/detail_v4.1.php?query=&id=rs6716856) | G | T | 0.96 | IWS1 | Intergenic |
| 2 | 127455012 | 0.81 | 0.9 | [rs777567](http://www.broadinstitute.org/mammals/haploreg/detail_v4.1.php?query=&id=rs777567) | G | T | 0.04 | IWS1 | Intergenic |
| 2 | 127455583 | 0.81 | 0.9 | [rs77511630](http://www.broadinstitute.org/mammals/haploreg/detail_v4.1.php?query=&id=rs77511630) | C | G | 0.04 | IWS1 | Intergenic |
| 2 | 127469132 | 1 | 1 | [rs6748999](http://www.broadinstitute.org/mammals/haploreg/detail_v4.1.php?query=&id=rs6748999) | A | G | 0.04 | IWS1 | Intergenic |
| 2 | 127506177 | 0.85 | 0.93 | [rs140923032](http://www.broadinstitute.org/mammals/haploreg/detail_v4.1.php?query=&id=rs140923032) | G | A | 0.04 | IWS1 | Intronic |
| 2 | 127507153 | 0.85 | 0.93 | [rs114358254](http://www.broadinstitute.org/mammals/haploreg/detail_v4.1.php?query=&id=rs114358254) | T | C | 0.04 | IWS1 | Intronic |
| 2 | 127512132 | 0.82 | 0.93 | [rs75704656](http://www.broadinstitute.org/mammals/haploreg/detail_v4.1.php?query=&id=rs75704656) | G | A | 0.04 | IWS1 | Intronic |
| **Locus 6. Query SNP: rs12491812 and variants with r^2^ >= 0.8** | | | | | |  |  |  |  |
| 3 | 50497204 | 0.82 | 1 | [rs9814874](http://www.broadinstitute.org/mammals/haploreg/detail_v4.1.php?query=&id=rs9814874) | G | A | 0.01 | CACNA2D2 | Intronic |
| 3 | 50501788 | 0.82 | 1 | [rs1107312](http://www.broadinstitute.org/mammals/haploreg/detail_v4.1.php?query=&id=rs1107312) | A | G | 0.01 | CACNA2D2 | Intronic |
| 3 | 50505295 | 0.82 | 1 | [rs3806706](http://www.broadinstitute.org/mammals/haploreg/detail_v4.1.php?query=&id=rs3806706) | G | C | 0.01 | 1.1kb 5' of CACNA2D2 | Intergenic |
| 3 | 50518502 | 1 | 1 | [rs17050991](http://www.broadinstitute.org/mammals/haploreg/detail_v4.1.php?query=&id=rs17050991) | A | C | 0.01 | 14kb 5' of CACNA2D2 | Intergenic |
| 3 | 50519150 | 1 | 1 | [rs12491812](http://www.broadinstitute.org/mammals/haploreg/detail_v4.1.php?query=&id=rs12491812) | C | T | 0.01 | 15kb 5' of CACNA2D2 | intergenic |
| 3 | 50521386 | 1 | 1 | [rs61574029](http://www.broadinstitute.org/mammals/haploreg/detail_v4.1.php?query=&id=rs61574029) | T | G | 0.01 | 17kb 5' of CACNA2D2 | intergenic |
| 3 | 50522770 | 1 | 1 | [rs61613532](http://www.broadinstitute.org/mammals/haploreg/detail_v4.1.php?query=&id=rs61613532) | T | C | 0.01 | 19kb 5' of CACNA2D2 | intergenic |
| 3 | 50523067 | 1 | 1 | [rs61193866](http://www.broadinstitute.org/mammals/haploreg/detail_v4.1.php?query=&id=rs61193866) | G | A | 0.01 | 19kb 5' of CACNA2D2 | intergenic |
| 3 | 50523127 | 0.82 | 1 | [rs72938758](http://www.broadinstitute.org/mammals/haploreg/detail_v4.1.php?query=&id=rs72938758) | A | G | 0.01 | 19kb 5' of CACNA2D2 | intergenic |
| 3 | 50525135 | 1 | 1 | [rs12487817](http://www.broadinstitute.org/mammals/haploreg/detail_v4.1.php?query=&id=rs12487817) | T | C | 0.01 | 21kb 5' of CACNA2D2 | intergenic |
| 3 | 50526739 | 1 | 1 | [rs12486143](http://www.broadinstitute.org/mammals/haploreg/detail_v4.1.php?query=&id=rs12486143) | G | T | 0.01 | 22kb 5' of CACNA2D2 | intergenic |
| 3 | 50529066 | 1 | 1 | [rs4688713](http://www.broadinstitute.org/mammals/haploreg/detail_v4.1.php?query=&id=rs4688713) | A | G | 0.01 | 25kb 5' of CACNA2D2 | intergenic |
| 3 | 50529104 | 1 | -1 | [rs6766638](http://www.broadinstitute.org/mammals/haploreg/detail_v4.1.php?query=&id=rs6766638) | G | T | 0.99 | 25kb 5' of CACNA2D2 | intergenic |
| 3 | 50530757 | 1 | 1 | [rs73835531](http://www.broadinstitute.org/mammals/haploreg/detail_v4.1.php?query=&id=rs73835531) | T | C | 0.01 | 27kb 5' of CACNA2D2 | intergenic |
| 3 | 50532344 | 1 | 1 | [rs17050995](http://www.broadinstitute.org/mammals/haploreg/detail_v4.1.php?query=&id=rs17050995) | A | G | 0.01 | 26kb 3' of C3orf18 | intergenic |
| 3 | 50540850 | 0.89 | 1 | [rs17051000](http://www.broadinstitute.org/mammals/haploreg/detail_v4.1.php?query=&id=rs17051000) | A | T | 0.01 | 17kb 3' of C3orf18 | intergenic |
| 3 | 50544526 | 0.89 | 1 | [rs12494094](http://www.broadinstitute.org/mammals/haploreg/detail_v4.1.php?query=&id=rs12494094) | A | C | 0.01 | 14kb 3' of C3orf18 | intergenic |
| 3 | 50550745 | 0.9 | 1 | [rs56170012](http://www.broadinstitute.org/mammals/haploreg/detail_v4.1.php?query=&id=rs56170012) | A | G | 0.01 | 7.3kb 3' of C3orf18 | intergenic |
| 3 | 50551935 | 0.89 | 1 | [rs60552073](http://www.broadinstitute.org/mammals/haploreg/detail_v4.1.php?query=&id=rs60552073) | C | T | 0.01 | 6.1kb 3' of C3orf18 | intergenic |
| 3 | 50553197 | 0.89 | 1 | [rs4688710](http://www.broadinstitute.org/mammals/haploreg/detail_v4.1.php?query=&id=rs4688710) | G | A | 0.01 | 4.8kb 3' of C3orf18 | intergenic |
| 3 | 50571017 | 0.89 | 1 | [rs2227293](http://www.broadinstitute.org/mammals/haploreg/detail_v4.1.php?query=&id=rs2227293) | G | A | 0.01 | C3orf18 | 5'-UTR |
| 3 | 50582612 | 0.89 | 1 | [rs2285090](http://www.broadinstitute.org/mammals/haploreg/detail_v4.1.php?query=&id=rs2285090) | T | C | 0.01 | HEMK1 | 3'-UTR |
| 3 | 50584763 | 0.89 | 1 | [rs72341372](http://www.broadinstitute.org/mammals/haploreg/detail_v4.1.php?query=&id=rs72341372) | CAT | C | 0.01 | HEMK1 | 3'-UTR |
| 3 | 50586144 | 0.89 | 1 | [rs3749265](http://www.broadinstitute.org/mammals/haploreg/detail_v4.1.php?query=&id=rs3749265) | T | A | 0.01 | 1.2kb 3' of HEMK1 | intergenic |
| 3 | 50586245 | 0.89 | 1 | [rs1894459](http://www.broadinstitute.org/mammals/haploreg/detail_v4.1.php?query=&id=rs1894459) | A | G | 0.01 | 1.3kb 3' of HEMK1 | intergenic |
| 3 | 50589619 | 0.89 | 1 | [rs79192940](http://www.broadinstitute.org/mammals/haploreg/detail_v4.1.php?query=&id=rs79192940) | G | A | 0.01 | 4.7kb 3' of HEMK1 | intergenic |
| 3 | 50592228 | 0.89 | 1 | [rs12494326](http://www.broadinstitute.org/mammals/haploreg/detail_v4.1.php?query=&id=rs12494326) | C | G | 0.01 | 7.3kb 3' of HEMK1 | intergenic |
| 3 | 50596404 | 0.89 | 1 | [rs61316441](http://www.broadinstitute.org/mammals/haploreg/detail_v4.1.php?query=&id=rs61316441) | C | T | 0.01 | 10kb 3' of CISH | intergenic |
| 3 | 50596595 | 0.89 | 1 | [rs4688707](http://www.broadinstitute.org/mammals/haploreg/detail_v4.1.php?query=&id=rs4688707) | C | T | 0.01 | 9.9kb 3' of CISH | intergenic |
| 3 | 50607727 | 0.89 | 1 | [rs2239753](http://www.broadinstitute.org/mammals/haploreg/detail_v4.1.php?query=&id=rs2239753) | A | G | 0.01 | **CISH** | **synonymous** |
| 3 | 50607982 | 0.89 | 1 | [rs2239752](http://www.broadinstitute.org/mammals/haploreg/detail_v4.1.php?query=&id=rs2239752) | G | A | 0.01 | **CISH** | **synonymous** |
| **Locus 7. Query SNP: rs16891982 and variants with r^2^ >= 0.8** | | | | | |  |  |  |  |
| 5 | 33951588 | 1 | 1 | [rs16891982](http://www.broadinstitute.org/mammals/haploreg/detail_v4.1.php?query=&id=rs16891982) | C | G | 0.97 | SLC45A2 | intergenic |
| **Locus 8. Query SNP: rs11760176 and variants with r^2^ >= 0.8** | | | | | |  |  |  |  |
| 6 | 29980813 | 0.84 | 0.97 | [rs139093747](http://www.broadinstitute.org/mammals/haploreg/detail_v4.1.php?query=&id=rs139093747) | GA | G | 0.04 | 2.4kb 3' of HCG9 | intergenic |
| 6 | 29982544 | 0.84 | 0.97 | [rs3734830](http://www.broadinstitute.org/mammals/haploreg/detail_v4.1.php?query=&id=rs3734830) | G | A | 0.04 | 4.1kb 3' of HCG9 | intergenic |
| 6 | 29982640 | 0.84 | 0.97 | [rs3734831](http://www.broadinstitute.org/mammals/haploreg/detail_v4.1.php?query=&id=rs3734831) | T | C | 0.04 | 4.2kb 3' of HCG9 | intergenic |
| 6 | 29982800 | 0.84 | 0.97 | [rs3734833](http://www.broadinstitute.org/mammals/haploreg/detail_v4.1.php?query=&id=rs3734833) | A | G | 0.04 | 4.4kb 3' of HCG9 | intergenic |
| 6 | 29982804 | 0.86 | 0.96 | [rs3734834](http://www.broadinstitute.org/mammals/haploreg/detail_v4.1.php?query=&id=rs3734834) | A | G | 0.04 | 4.4kb 3' of HCG9 | intergenic |
| 6 | 29983062 | 0.84 | 0.97 | [rs73725904](http://www.broadinstitute.org/mammals/haploreg/detail_v4.1.php?query=&id=rs73725904) | G | C | 0.04 | 4.7kb 3' of HCG9 | intergenic |
| 6 | 29983202 | 0.84 | 0.97 | [rs73428121](http://www.broadinstitute.org/mammals/haploreg/detail_v4.1.php?query=&id=rs73428121) | A | C | 0.04 | 4.8kb 3' of HCG9 | intergenic |
| 6 | 29983272 | 0.84 | 0.97 | [rs73428122](http://www.broadinstitute.org/mammals/haploreg/detail_v4.1.php?query=&id=rs73428122) | T | G | 0.04 | 4.9kb 3' of HCG9 | intergenic |
| 6 | 29983284 | 0.81 | 0.93 | [rs114832360](http://www.broadinstitute.org/mammals/haploreg/detail_v4.1.php?query=&id=rs114832360) | C | T | 0.04 | 4.9kb 3' of HCG9 | intergenic |
| 6 | 29983307 | 0.81 | 0.93 | [rs73428125](http://www.broadinstitute.org/mammals/haploreg/detail_v4.1.php?query=&id=rs73428125) | T | A | 0.04 | 4.9kb 3' of HCG9 | intergenic |
| 6 | 29983427 | 0.84 | 0.97 | [rs57035982](http://www.broadinstitute.org/mammals/haploreg/detail_v4.1.php?query=&id=rs57035982) | G | A | 0.04 | 5kb 3' of HCG9 | intergenic |
| 6 | 29983496 | 0.84 | 0.97 | [rs56015532](http://www.broadinstitute.org/mammals/haploreg/detail_v4.1.php?query=&id=rs56015532) | A | G | 0.04 | 5.1kb 3' of HCG9 | intergenic |
| 6 | 29983506 | 0.84 | 0.97 | [rs57080532](http://www.broadinstitute.org/mammals/haploreg/detail_v4.1.php?query=&id=rs57080532) | A | C | 0.04 | 5.1kb 3' of HCG9 | intergenic |
| 6 | 29983517 | 0.84 | 0.97 | [rs57680173](http://www.broadinstitute.org/mammals/haploreg/detail_v4.1.php?query=&id=rs57680173) | G | T | 0.04 | 5.1kb 3' of HCG9 | intergenic |
| 6 | 29983834 | 0.81 | 0.93 | [rs55760574](http://www.broadinstitute.org/mammals/haploreg/detail_v4.1.php?query=&id=rs55760574) | A | T | 0.04 | 5.4kb 3' of HCG9 | intergenic |
| 6 | 29983842 | 0.81 | 0.93 | [rs60587228](http://www.broadinstitute.org/mammals/haploreg/detail_v4.1.php?query=&id=rs60587228) | G | A | 0.04 | 5.4kb 3' of HCG9 | intergenic |
| 6 | 29983902 | 0.81 | 0.93 | [rs56385845](http://www.broadinstitute.org/mammals/haploreg/detail_v4.1.php?query=&id=rs56385845) | G | A | 0.04 | 5.5kb 3' of HCG9 | intergenic |
| 6 | 29984114 | 0.84 | 0.97 | [rs58161185](http://www.broadinstitute.org/mammals/haploreg/detail_v4.1.php?query=&id=rs58161185) | A | G | 0.04 | 5.7kb 3' of HCG9 | intergenic |
| 6 | 29984287 | 0.87 | 0.97 | [rs201313489](http://www.broadinstitute.org/mammals/haploreg/detail_v4.1.php?query=&id=rs201313489) | CT | C | 0.04 | 5.9kb 3' of HCG9 | intergenic |
| 6 | 29984292 | 0.87 | 0.97 | [rs35361490](http://www.broadinstitute.org/mammals/haploreg/detail_v4.1.php?query=&id=rs35361490) | G | A | 0.04 | 5.9kb 3' of HCG9 | intergenic |
| 6 | 29984325 | 0.87 | 0.97 | [rs34513540](http://www.broadinstitute.org/mammals/haploreg/detail_v4.1.php?query=&id=rs34513540) | G | T | 0.04 | 5.9kb 3' of HCG9 | intergenic |
| 6 | 29984329 | 0.84 | 0.93 | [rs114426132](http://www.broadinstitute.org/mammals/haploreg/detail_v4.1.php?query=&id=rs114426132) | C | T | 0.04 | 5.9kb 3' of HCG9 | intergenic |
| 6 | 29984330 | 0.84 | 0.93 | [rs115313680](http://www.broadinstitute.org/mammals/haploreg/detail_v4.1.php?query=&id=rs115313680) | C | G | 0.04 | 5.9kb 3' of HCG9 | intergenic |
| 6 | 29984370 | 0.84 | 0.97 | [rs11759891](http://www.broadinstitute.org/mammals/haploreg/detail_v4.1.php?query=&id=rs11759891) | A | T | 0.04 | 6kb 3' of HCG9 | intergenic |
| 6 | 29984385 | 0.84 | 0.97 | [rs11757750](http://www.broadinstitute.org/mammals/haploreg/detail_v4.1.php?query=&id=rs11757750) | T | C | 0.04 | 6kb 3' of HCG9 | intergenic |
| 6 | 29984538 | 0.81 | 0.93 | [rs11755337](http://www.broadinstitute.org/mammals/haploreg/detail_v4.1.php?query=&id=rs11755337) | C | G | 0.04 | 6.1kb 3' of HCG9 | intergenic |
| 6 | 29984548 | 0.81 | 0.93 | [rs11757792](http://www.broadinstitute.org/mammals/haploreg/detail_v4.1.php?query=&id=rs11757792) | T | C | 0.04 | 6.1kb 3' of HCG9 | intergenic |
| 6 | 29984582 | 0.84 | 0.97 | [rs11755374](http://www.broadinstitute.org/mammals/haploreg/detail_v4.1.php?query=&id=rs11755374) | G | A | 0.04 | 6.2kb 3' of HCG9 | intergenic |
| 6 | 29984642 | 0.84 | 0.97 | [rs61704409](http://www.broadinstitute.org/mammals/haploreg/detail_v4.1.php?query=&id=rs61704409) | C | T | 0.04 | 6.2kb 3' of HCG9 | intergenic |
| 6 | 29984705 | 0.84 | 0.97 | [rs35144883](http://www.broadinstitute.org/mammals/haploreg/detail_v4.1.php?query=&id=rs35144883) | C | T | 0.04 | 6.3kb 3' of HCG9 | intergenic |
| 6 | 29984776 | 0.84 | 0.97 | [rs58867136](http://www.broadinstitute.org/mammals/haploreg/detail_v4.1.php?query=&id=rs58867136) | A | G | 0.04 | 6.4kb 3' of HCG9 | intergenic |
| 6 | 29984927 | 0.84 | 0.97 | [rs17186930](http://www.broadinstitute.org/mammals/haploreg/detail_v4.1.php?query=&id=rs17186930) | C | T | 0.04 | 6.5kb 3' of HCG9 | intergenic |
| 6 | 29985037 | 0.84 | 0.97 | [rs17186937](http://www.broadinstitute.org/mammals/haploreg/detail_v4.1.php?query=&id=rs17186937) | T | C | 0.04 | 6.6kb 3' of HCG9 | intergenic |
| 6 | 29985143 | 0.84 | 0.97 | [rs17186944](http://www.broadinstitute.org/mammals/haploreg/detail_v4.1.php?query=&id=rs17186944) | C | T | 0.04 | 6.7kb 3' of HCG9 | intergenic |
| 6 | 29985154 | 0.84 | 0.97 | [rs11755966](http://www.broadinstitute.org/mammals/haploreg/detail_v4.1.php?query=&id=rs11755966) | G | A | 0.04 | 6.7kb 3' of HCG9 | intergenic |
| 6 | 29985258 | 0.84 | 0.97 | [rs11755984](http://www.broadinstitute.org/mammals/haploreg/detail_v4.1.php?query=&id=rs11755984) | G | A | 0.04 | 6.9kb 3' of HCG9 | intergenic |
| 6 | 29985322 | 0.84 | 0.97 | [rs11755961](http://www.broadinstitute.org/mammals/haploreg/detail_v4.1.php?query=&id=rs11755961) | C | T | 0.04 | 6.9kb 3' of HCG9 | intergenic |
| 6 | 29985493 | 0.84 | 0.97 | [rs11755992](http://www.broadinstitute.org/mammals/haploreg/detail_v4.1.php?query=&id=rs11755992) | C | G | 0.04 | 7.1kb 3' of HCG9 | intergenic |
| 6 | 29985534 | 0.84 | 0.97 | [rs11751331](http://www.broadinstitute.org/mammals/haploreg/detail_v4.1.php?query=&id=rs11751331) | A | T | 0.04 | 7.1kb 3' of HCG9 | intergenic |
| 6 | 29985651 | 0.84 | 0.97 | [rs11758493](http://www.broadinstitute.org/mammals/haploreg/detail_v4.1.php?query=&id=rs11758493) | T | G | 0.04 | 7.2kb 3' of HCG9 | intergenic |
| 6 | 29985849 | 0.84 | 0.97 | [rs73725909](http://www.broadinstitute.org/mammals/haploreg/detail_v4.1.php?query=&id=rs73725909) | C | T | 0.04 | 7.4kb 3' of HCG9 | intergenic |
| 6 | 29985851 | 0.81 | 0.93 | [rs73725910](http://www.broadinstitute.org/mammals/haploreg/detail_v4.1.php?query=&id=rs73725910) | G | A | 0.04 | 7.4kb 3' of HCG9 | intergenic |
| 6 | 29985941 | 0.81 | 0.93 | [rs73725912](http://www.broadinstitute.org/mammals/haploreg/detail_v4.1.php?query=&id=rs73725912) | G | A | 0.04 | 7.5kb 3' of HCG9 | intergenic |
| 6 | 29986026 | 0.84 | 0.97 | [rs73428152](http://www.broadinstitute.org/mammals/haploreg/detail_v4.1.php?query=&id=rs73428152) | C | A | 0.04 | 7.6kb 3' of HCG9 | intergenic |
| 6 | 29986208 | 0.84 | 0.97 | [rs5875226](http://www.broadinstitute.org/mammals/haploreg/detail_v4.1.php?query=&id=rs5875226) | C | CA | 0.04 | 7.8kb 3' of HCG9 | intergenic |
| 6 | 29986211 | 0.81 | 0.93 | [rs35685401](http://www.broadinstitute.org/mammals/haploreg/detail_v4.1.php?query=&id=rs35685401) | C | T | 0.04 | 7.8kb 3' of HCG9 | intergenic |
| 6 | 29986334 | 0.84 | 0.97 | [rs12662947](http://www.broadinstitute.org/mammals/haploreg/detail_v4.1.php?query=&id=rs12662947) | T | C | 0.04 | 7.9kb 3' of HCG9 | intergenic |
| 6 | 29986422 | 0.84 | 0.97 | [rs12661411](http://www.broadinstitute.org/mammals/haploreg/detail_v4.1.php?query=&id=rs12661411) | C | T | 0.04 | 8kb 3' of HCG9 | intergenic |
| 6 | 29986503 | 0.84 | 0.97 | [rs12662967](http://www.broadinstitute.org/mammals/haploreg/detail_v4.1.php?query=&id=rs12662967) | T | C | 0.04 | 8.1kb 3' of HCG9 | intergenic |
| 6 | 29986679 | 0.81 | 0.93 | [rs73410503](http://www.broadinstitute.org/mammals/haploreg/detail_v4.1.php?query=&id=rs73410503) | G | A | 0.04 | 8.3kb 3' of HCG9 | intergenic |
| 6 | 29986840 | 0.84 | 0.97 | [rs34894339](http://www.broadinstitute.org/mammals/haploreg/detail_v4.1.php?query=&id=rs34894339) | A | G | 0.04 | 8.4kb 3' of HCG9 | intergenic |
| 6 | 29986847 | 0.84 | 0.97 | [rs73725917](http://www.broadinstitute.org/mammals/haploreg/detail_v4.1.php?query=&id=rs73725917) | A | C,T | 0.04 | 8.4kb 3' of HCG9 | intergenic |
| 6 | 29987074 | 0.84 | 0.97 | [rs17186979](http://www.broadinstitute.org/mammals/haploreg/detail_v4.1.php?query=&id=rs17186979) | A | G | 0.04 | 8.7kb 3' of HCG9 | intergenic |
| 6 | 29987280 | 0.84 | 0.97 | [rs17187021](http://www.broadinstitute.org/mammals/haploreg/detail_v4.1.php?query=&id=rs17187021) | T | C | 0.04 | 8.9kb 3' of HCG9 | intergenic |
| 6 | 29987310 | 0.81 | 0.93 | [rs17187035](http://www.broadinstitute.org/mammals/haploreg/detail_v4.1.php?query=&id=rs17187035) | T | C | 0.04 | 8.9kb 3' of HCG9 | intergenic |
| 6 | 29987445 | 0.84 | 0.97 | [rs17187077](http://www.broadinstitute.org/mammals/haploreg/detail_v4.1.php?query=&id=rs17187077) | A | C | 0.04 | 9kb 3' of HCG9 | intergenic |
| 6 | 29987611 | 0.81 | 0.93 | [rs17187141](http://www.broadinstitute.org/mammals/haploreg/detail_v4.1.php?query=&id=rs17187141) | T | C | 0.04 | 9.2kb 3' of HCG9 | intergenic |
| 6 | 29987762 | 0.84 | 0.97 | [rs17180794](http://www.broadinstitute.org/mammals/haploreg/detail_v4.1.php?query=&id=rs17180794) | T | C | 0.04 | 9.4kb 3' of HCG9 | intergenic |
| 6 | 29987833 | 0.8 | 0.9 | [rs73410515](http://www.broadinstitute.org/mammals/haploreg/detail_v4.1.php?query=&id=rs73410515) | G | A | 0.04 | 9.4kb 3' of HCG9 | intergenic |
| 6 | 29987997 | 0.84 | 0.97 | [rs73410518](http://www.broadinstitute.org/mammals/haploreg/detail_v4.1.php?query=&id=rs73410518) | T | C,G | 0.04 | 9.6kb 3' of HCG9 | intergenic |
| 6 | 29988243 | 0.84 | 0.97 | [rs76517404](http://www.broadinstitute.org/mammals/haploreg/detail_v4.1.php?query=&id=rs76517404) | T | C | 0.04 | 9.8kb 3' of HCG9 | intergenic |
| 6 | 29988248 | 0.81 | 0.93 | [rs201767378](http://www.broadinstitute.org/mammals/haploreg/detail_v4.1.php?query=&id=rs201767378) | AG | A | 0.04 | 9.8kb 3' of HCG9 | intergenic |
| 6 | 29988272 | 0.84 | 0.97 | [rs73410524](http://www.broadinstitute.org/mammals/haploreg/detail_v4.1.php?query=&id=rs73410524) | G | A | 0.04 | 9.9kb 3' of HCG9 | intergenic |
| 6 | 29988401 | 0.84 | 0.97 | [rs73410525](http://www.broadinstitute.org/mammals/haploreg/detail_v4.1.php?query=&id=rs73410525) | C | A | 0.04 | 10kb 3' of HCG9 | intergenic |
| 6 | 29988516 | 0.84 | 0.97 | [rs112793716](http://www.broadinstitute.org/mammals/haploreg/detail_v4.1.php?query=&id=rs112793716) | C | T | 0.04 | 10kb 3' of HCG9 | intergenic |
| 6 | 29988552 | 0.84 | 0.97 | [rs73410528](http://www.broadinstitute.org/mammals/haploreg/detail_v4.1.php?query=&id=rs73410528) | G | A | 0.04 | 10kb 3' of HCG9 | intergenic |
| 6 | 29988698 | 0.81 | 0.93 | [rs147065659](http://www.broadinstitute.org/mammals/haploreg/detail_v4.1.php?query=&id=rs147065659) | TGAG | T | 0.04 | 10kb 3' of HCG9 | intergenic |
| 6 | 29988943 | 0.84 | 0.97 | [rs12662336](http://www.broadinstitute.org/mammals/haploreg/detail_v4.1.php?query=&id=rs12662336) | G | A | 0.04 | 11kb 3' of HCG9 | intergenic |
| 6 | 29989300 | 0.84 | 0.97 | [rs12662611](http://www.broadinstitute.org/mammals/haploreg/detail_v4.1.php?query=&id=rs12662611) | C | T | 0.04 | 11kb 3' of HCG9 | intergenic |
| 6 | 29989508 | 0.84 | 0.97 | [rs12664166](http://www.broadinstitute.org/mammals/haploreg/detail_v4.1.php?query=&id=rs12664166) | T | G | 0.04 | 11kb 3' of HCG9 | intergenic |
| 6 | 29993802 | 0.84 | 0.97 | [rs17187224](http://www.broadinstitute.org/mammals/haploreg/detail_v4.1.php?query=&id=rs17187224) | C | T | 0.04 | 7.2kb 3' of ZNRD1-AS1 | intergenic |
| 6 | 29994016 | 0.84 | 0.97 | [rs17180864](http://www.broadinstitute.org/mammals/haploreg/detail_v4.1.php?query=&id=rs17180864) | C | T | 0.04 | 7kb 3' of ZNRD1-AS1 | intergenic |
| 6 | 29994066 | 0.84 | 0.97 | [rs17187252](http://www.broadinstitute.org/mammals/haploreg/detail_v4.1.php?query=&id=rs17187252) | G | A | 0.04 | 6.9kb 3' of ZNRD1-AS1 | intergenic |
| 6 | 29995744 | 0.84 | 0.97 | [rs6911583](http://www.broadinstitute.org/mammals/haploreg/detail_v4.1.php?query=&id=rs6911583) | T | C | 0.04 | 5.3kb 3' of ZNRD1-AS1 | intergenic |
| 6 | 29995947 | 0.84 | 0.97 | [rs58858573](http://www.broadinstitute.org/mammals/haploreg/detail_v4.1.php?query=&id=rs58858573) | C | A | 0.04 | 5.1kb 3' of ZNRD1-AS1 | intergenic |
| 6 | 29996620 | 0.84 | 0.97 | [rs10947040](http://www.broadinstitute.org/mammals/haploreg/detail_v4.1.php?query=&id=rs10947040) | G | A | 0.04 | 4.4kb 3' of ZNRD1-AS1 | intergenic |
| 6 | 29997052 | 0.84 | 0.97 | [rs12110437](http://www.broadinstitute.org/mammals/haploreg/detail_v4.1.php?query=&id=rs12110437) | A | T | 0.04 | 4kb 3' of ZNRD1-AS1 | intergenic |
| 6 | 29998038 | 0.84 | 0.97 | [rs72502507](http://www.broadinstitute.org/mammals/haploreg/detail_v4.1.php?query=&id=rs72502507) | A | C | 0.04 | 3kb 3' of ZNRD1-AS1 | intergenic |
| 6 | 29998247 | 0.81 | 0.93 | [rs12111210](http://www.broadinstitute.org/mammals/haploreg/detail_v4.1.php?query=&id=rs12111210) | G | A | 0.04 | 2.8kb 3' of ZNRD1-AS1 | intergenic |
| 6 | 29998893 | 0.81 | 0.93 | [rs6917877](http://www.broadinstitute.org/mammals/haploreg/detail_v4.1.php?query=&id=rs6917877) | A | G | 0.04 | 2.1kb 3' of ZNRD1-AS1 | intergenic |
| 6 | 29998897 | 0.84 | 0.97 | [rs6940956](http://www.broadinstitute.org/mammals/haploreg/detail_v4.1.php?query=&id=rs6940956) | T | A | 0.04 | 2.1kb 3' of ZNRD1-AS1 | intergenic |
| 6 | 29999555 | 0.81 | 0.93 | [rs17187349](http://www.broadinstitute.org/mammals/haploreg/detail_v4.1.php?query=&id=rs17187349) | T | C | 0.04 | 1.5kb 3' of ZNRD1-AS1 | intergenic |
| 6 | 30000403 | 0.81 | 0.93 | [rs12664967](http://www.broadinstitute.org/mammals/haploreg/detail_v4.1.php?query=&id=rs12664967) | C | G | 0.04 | 607bp 3' of ZNRD1-AS1 | intergenic |
| 6 | 30000678 | 0.87 | 0.97 | [rs10947042](http://www.broadinstitute.org/mammals/haploreg/detail_v4.1.php?query=&id=rs10947042) | C | T | 0.04 | 332bp 3' of ZNRD1-AS1 | intergenic |
| 6 | 30000900 | 0.87 | 0.97 | [rs28698859](http://www.broadinstitute.org/mammals/haploreg/detail_v4.1.php?query=&id=rs28698859) | G | A | 0.04 | 110bp 3' of ZNRD1-AS1 | intergenic |
|  |  | 0.91 | 1 | [rs59854844](http://www.broadinstitute.org/mammals/haploreg/detail_v4.1.php?query=&id=rs59854844) | G | A | 0.04 | ZNRD1-AS1 | intergenic |
| 6 | 30001413 | 0.91 | 1 | [rs72502509](http://www.broadinstitute.org/mammals/haploreg/detail_v4.1.php?query=&id=rs72502509) | C | T | 0.04 | ZNRD1-AS1 | intergenic |
| 6 | 30001656 | 0.91 | 1 | [rs12665444](http://www.broadinstitute.org/mammals/haploreg/detail_v4.1.php?query=&id=rs12665444) | G | C | 0.04 | ZNRD1-AS1 | intergenic |
| 6 | 30002475 | 0.91 | 1 | [rs10947043](http://www.broadinstitute.org/mammals/haploreg/detail_v4.1.php?query=&id=rs10947043) | T | C | 0.04 | ZNRD1-AS1 | intergenic |
| 6 | 30002561 | 0.91 | 1 | [rs11755549](http://www.broadinstitute.org/mammals/haploreg/detail_v4.1.php?query=&id=rs11755549) | A | G | 0.04 | ZNRD1-AS1 | intergenic |
| 6 | 30002650 | 1 | 1 | [rs11760176](http://www.broadinstitute.org/mammals/haploreg/detail_v4.1.php?query=&id=rs11760176) | C | T | 0.04 | ZNRD1-AS1 | intergenic |
| 6 | 30003151 | 0.91 | 1 | [rs7742799](http://www.broadinstitute.org/mammals/haploreg/detail_v4.1.php?query=&id=rs7742799) | G | A | 0.04 | ZNRD1-AS1 | intronic |
| 6 | 30003257 | 0.91 | 1 | [rs7762851](http://www.broadinstitute.org/mammals/haploreg/detail_v4.1.php?query=&id=rs7762851) | T | G | 0.04 | ZNRD1-AS1 | intronic |
| 6 | 30003300 | 0.91 | 1 | [rs7742712](http://www.broadinstitute.org/mammals/haploreg/detail_v4.1.php?query=&id=rs7742712) | C | G | 0.04 | ZNRD1-AS1 | intronic |
| 6 | 30003514 | 0.87 | 0.97 | [rs11964113](http://www.broadinstitute.org/mammals/haploreg/detail_v4.1.php?query=&id=rs11964113) | T | C | 0.04 | ZNRD1-AS1 | intronic |
| 6 | 30004196 | 0.91 | 1 | [rs11754605](http://www.broadinstitute.org/mammals/haploreg/detail_v4.1.php?query=&id=rs11754605) | T | C | 0.04 | ZNRD1-AS1 | intronic |
| 6 | 30004615 | 0.91 | 1 | [rs6933897](http://www.broadinstitute.org/mammals/haploreg/detail_v4.1.php?query=&id=rs6933897) | T | A | 0.04 | ZNRD1-AS1 | intronic |
| 6 | 30004997 | 0.91 | 1 | [rs6912001](http://www.broadinstitute.org/mammals/haploreg/detail_v4.1.php?query=&id=rs6912001) | G | C | 0.04 | ZNRD1-AS1 | intronic |
| 6 | 30005193 | 0.91 | 1 | [rs11965458](http://www.broadinstitute.org/mammals/haploreg/detail_v4.1.php?query=&id=rs11965458) | T | C | 0.04 | ZNRD1-AS1 | intronic |
| 6 | 30005410 | 0.91 | 1 | [rs11965524](http://www.broadinstitute.org/mammals/haploreg/detail_v4.1.php?query=&id=rs11965524) | T | A | 0.04 | ZNRD1-AS1 | intronic |
| 6 | 30006212 | 0.87 | 0.97 | [rs73725950](http://www.broadinstitute.org/mammals/haploreg/detail_v4.1.php?query=&id=rs73725950) | G | C | 0.04 | ZNRD1-AS1 | intronic |
| 6 | 30006306 | 0.87 | 0.97 | [rs146541051](http://www.broadinstitute.org/mammals/haploreg/detail_v4.1.php?query=&id=rs146541051) | CG | C | 0.04 | ZNRD1-AS1 | intronic |
| 6 | 30006569 | 0.91 | 1 | [rs73414461](http://www.broadinstitute.org/mammals/haploreg/detail_v4.1.php?query=&id=rs73414461) | G | C | 0.04 | ZNRD1-AS1 | intronic |
| 6 | 30007942 | 0.91 | 1 | [rs73414465](http://www.broadinstitute.org/mammals/haploreg/detail_v4.1.php?query=&id=rs73414465) | T | C | 0.04 | ZNRD1-AS1 | intronic |
| 6 | 30007989 | 0.91 | 1 | [rs3765608](http://www.broadinstitute.org/mammals/haploreg/detail_v4.1.php?query=&id=rs3765608) | G | C | 0.04 | ZNRD1-AS1 | intronic |
| 6 | 30007998 | 0.91 | 1 | [rs73725953](http://www.broadinstitute.org/mammals/haploreg/detail_v4.1.php?query=&id=rs73725953) | A | C | 0.04 | ZNRD1-AS1 | intronic |
| 6 | 30008964 | 0.91 | 1 | [rs12663252](http://www.broadinstitute.org/mammals/haploreg/detail_v4.1.php?query=&id=rs12663252) | A | G | 0.04 | ZNRD1-AS1 | intronic |
| 6 | 30010250 | 0.91 | 1 | [rs12661053](http://www.broadinstitute.org/mammals/haploreg/detail_v4.1.php?query=&id=rs12661053) | C | T | 0.04 | ZNRD1-AS1 | intronic |
| 6 | 30011866 | 0.91 | 1 | [rs11753431](http://www.broadinstitute.org/mammals/haploreg/detail_v4.1.php?query=&id=rs11753431) | G | A | 0.04 | ZNRD1-AS1 | intronic |
| 6 | 30012381 | 0.84 | 0.93 | [rs78453967](http://www.broadinstitute.org/mammals/haploreg/detail_v4.1.php?query=&id=rs78453967) | C | T | 0.04 | ZNRD1-AS1 | intronic |
| 6 | 30012413 | 0.84 | 0.93 | [rs11753957](http://www.broadinstitute.org/mammals/haploreg/detail_v4.1.php?query=&id=rs11753957) | C | T | 0.04 | ZNRD1-AS1 | intronic |
| 6 | 30012463 | 0.91 | 1 | [rs11753960](http://www.broadinstitute.org/mammals/haploreg/detail_v4.1.php?query=&id=rs11753960) | C | T | 0.04 | ZNRD1-AS1 | intronic |
| 6 | 30013222 | 0.84 | 0.93 | [rs11754620](http://www.broadinstitute.org/mammals/haploreg/detail_v4.1.php?query=&id=rs11754620) | G | A | 0.04 | ZNRD1-AS1 | intronic |
| 6 | 30013591 | 0.91 | 1 | [rs9261134](http://www.broadinstitute.org/mammals/haploreg/detail_v4.1.php?query=&id=rs9261134) | G | C,T | 0.04 | ZNRD1-AS1 | intronic |
| 6 | 30013956 | 0.91 | 1 | [rs11759388](http://www.broadinstitute.org/mammals/haploreg/detail_v4.1.php?query=&id=rs11759388) | A | G | 0.04 | ZNRD1-AS1 | intronic |
| 6 | 30014652 | 0.84 | 0.97 | [rs11965585](http://www.broadinstitute.org/mammals/haploreg/detail_v4.1.php?query=&id=rs11965585) | G | A | 0.04 | ZNRD1-AS1 | intronic |
| 6 | 30014986 | 0.84 | 0.97 | [rs11965659](http://www.broadinstitute.org/mammals/haploreg/detail_v4.1.php?query=&id=rs11965659) | G | C | 0.04 | ZNRD1-AS1 | intronic |
| 6 | 30015638 | 0.84 | 0.97 | [rs6902901](http://www.broadinstitute.org/mammals/haploreg/detail_v4.1.php?query=&id=rs6902901) | C | T | 0.04 | ZNRD1-AS1 | intronic |
| 6 | 30018365 | 0.84 | 0.97 | [rs6931672](http://www.broadinstitute.org/mammals/haploreg/detail_v4.1.php?query=&id=rs6931672) | G | A | 0.04 | ZNRD1-AS1 | intronic |
| 6 | 30018724 | 0.84 | 0.97 | [rs6932091](http://www.broadinstitute.org/mammals/haploreg/detail_v4.1.php?query=&id=rs6932091) | C | T | 0.04 | ZNRD1-AS1 | intronic |
| 6 | 30019414 | 0.84 | 0.97 | [rs17187488](http://www.broadinstitute.org/mammals/haploreg/detail_v4.1.php?query=&id=rs17187488) | G | A | 0.04 | ZNRD1-AS1 | intronic |
| 6 | 30021187 | 0.84 | 0.97 | [rs6928966](http://www.broadinstitute.org/mammals/haploreg/detail_v4.1.php?query=&id=rs6928966) | T | C | 0.04 | ZNRD1-AS1 | intronic |
| 6 | 30021411 | 0.84 | 0.97 | [rs17193851](http://www.broadinstitute.org/mammals/haploreg/detail_v4.1.php?query=&id=rs17193851) | T | C | 0.04 | ZNRD1-AS1 | intronic |
| 6 | 30021912 | 0.84 | 0.97 | [rs10947045](http://www.broadinstitute.org/mammals/haploreg/detail_v4.1.php?query=&id=rs10947045) | C | T | 0.04 | ZNRD1-AS1 | intronic |
| 6 | 30023157 | 0.84 | 0.97 | [rs10807060](http://www.broadinstitute.org/mammals/haploreg/detail_v4.1.php?query=&id=rs10807060) | G | A | 0.04 | ZNRD1-AS1 | intronic |
| 6 | 30023760 | 0.84 | 0.97 | [rs73725970](http://www.broadinstitute.org/mammals/haploreg/detail_v4.1.php?query=&id=rs73725970) | C | T | 0.04 | ZNRD1-AS1 | intronic |
| 6 | 30023796 | 0.84 | 0.97 | [rs73416498](http://www.broadinstitute.org/mammals/haploreg/detail_v4.1.php?query=&id=rs73416498) | G | A | 0.04 | ZNRD1-AS1 | intronic |
| 6 | 30024837 | 0.84 | 0.97 | [rs73416501](http://www.broadinstitute.org/mammals/haploreg/detail_v4.1.php?query=&id=rs73416501) | G | A | 0.04 | ZNRD1-AS1 | intronic |
| 6 | 30025737 | 0.84 | 0.97 | [rs10550781](http://www.broadinstitute.org/mammals/haploreg/detail_v4.1.php?query=&id=rs10550781) | ACACAC | A | 0.04 | ZNRD1-AS1 | intronic |
| 6 | 30026394 | 0.84 | 0.97 | [rs35763150](http://www.broadinstitute.org/mammals/haploreg/detail_v4.1.php?query=&id=rs35763150) | A | G | 0.04 | ZNRD1-AS1 | intronic |
| 6 | 30027247 | 0.84 | 0.97 | [rs76290849](http://www.broadinstitute.org/mammals/haploreg/detail_v4.1.php?query=&id=rs76290849) | T | C | 0.04 | ZNRD1-AS1 | intronic |
| 6 | 30027961 | 0.84 | 0.97 | [rs11967317](http://www.broadinstitute.org/mammals/haploreg/detail_v4.1.php?query=&id=rs11967317) | G | T | 0.04 | ZNRD1-AS1 | intronic |
| 6 | 30030209 | 0.84 | 0.97 | [rs7764237](http://www.broadinstitute.org/mammals/haploreg/detail_v4.1.php?query=&id=rs7764237) | A | G | 0.04 | ZNRD1-AS1 | intronic |
| 6 | 30030247 | 0.84 | 0.97 | [rs7746555](http://www.broadinstitute.org/mammals/haploreg/detail_v4.1.php?query=&id=rs7746555) | T | C | 0.04 | ZNRD1-AS1 | intronic |
| 6 | 30031232 | 0.84 | 0.97 | [rs61230487](http://www.broadinstitute.org/mammals/haploreg/detail_v4.1.php?query=&id=rs61230487) | C | T | 0.04 | ZNRD1-AS1 | intronic |
| 6 | 30031451 | 0.84 | 0.97 | [rs58555592](http://www.broadinstitute.org/mammals/haploreg/detail_v4.1.php?query=&id=rs58555592) | A | G | 0.04 | ZNRD1-AS1 | intronic |
| 6 | 30032165 | 0.84 | 0.97 | [rs2894006](http://www.broadinstitute.org/mammals/haploreg/detail_v4.1.php?query=&id=rs2894006) | G | A | 0.04 | ZNRD1-AS1 | intronic |
| 6 | 30032384 | 0.84 | 0.97 | [rs112400819](http://www.broadinstitute.org/mammals/haploreg/detail_v4.1.php?query=&id=rs112400819) | G | A | 0.04 | ZNRD1-AS1 | intronic |
| 6 | 30032420 | 0.84 | 0.97 | [rs113739823](http://www.broadinstitute.org/mammals/haploreg/detail_v4.1.php?query=&id=rs113739823) | A | T | 0.04 | ZNRD1-AS1 | intronic |
| 6 | 30032946 | 0.84 | 0.97 | [rs17187523](http://www.broadinstitute.org/mammals/haploreg/detail_v4.1.php?query=&id=rs17187523) | C | T | 0.04 | ZNRD1-AS1 | intronic |
| 6 | 30033196 | 0.84 | 0.97 | [rs10947046](http://www.broadinstitute.org/mammals/haploreg/detail_v4.1.php?query=&id=rs10947046) | T | G | 0.04 | ZNRD1-AS1 | intronic |
| 6 | 30035041 | 0.84 | 0.97 | [rs73420421](http://www.broadinstitute.org/mammals/haploreg/detail_v4.1.php?query=&id=rs73420421) | T | C | 0.04 | ZNRD1-AS1 | intergenic |
| 6 | 30036751 | 0.84 | 0.97 | [rs58980848](http://www.broadinstitute.org/mammals/haploreg/detail_v4.1.php?query=&id=rs58980848) | C | T | 0.04 | ZNRD1-AS1 | intronic |
| 6 | 30036837 | 0.84 | 0.97 | [rs60265332](http://www.broadinstitute.org/mammals/haploreg/detail_v4.1.php?query=&id=rs60265332) | A | T | 0.04 | ZNRD1-AS1 | intronic |
| 6 | 30036921 | 0.84 | 0.97 | [rs58314476](http://www.broadinstitute.org/mammals/haploreg/detail_v4.1.php?query=&id=rs58314476) | A | G | 0.04 | ZNRD1-AS1 | intronic |
| 6 | 30038011 | 0.84 | 0.97 | [rs73420434](http://www.broadinstitute.org/mammals/haploreg/detail_v4.1.php?query=&id=rs73420434) | A | T | 0.04 | ZNRD1-AS1 | intronic |
| 6 | 30038735 | 0.84 | 0.97 | [rs73725984](http://www.broadinstitute.org/mammals/haploreg/detail_v4.1.php?query=&id=rs73725984) | T | G | 0.04 | ZNRD1-AS1 | intronic |
| 6 | 30039339 | 0.84 | 0.97 | [rs73420439](http://www.broadinstitute.org/mammals/haploreg/detail_v4.1.php?query=&id=rs73420439) | T | C | 0.04 | ZNRD1-AS1 | intronic |
| 6 | 30039934 | 0.84 | 0.97 | [rs10947047](http://www.broadinstitute.org/mammals/haploreg/detail_v4.1.php?query=&id=rs10947047) | G | A | 0.04 | ZNRD1-AS1 | intronic |
| 6 | 30040207 | 0.84 | 0.97 | [rs11962771](http://www.broadinstitute.org/mammals/haploreg/detail_v4.1.php?query=&id=rs11962771) | G | A | 0.04 | ZNRD1-AS1 | intronic |
| 6 | 30041484 | 0.84 | 0.97 | [rs12661609](http://www.broadinstitute.org/mammals/haploreg/detail_v4.1.php?query=&id=rs12661609) | G | A | 0.04 | ZNRD1-AS1 | intronic |
| 6 | 30041887 | 0.84 | 0.97 | [rs12664794](http://www.broadinstitute.org/mammals/haploreg/detail_v4.1.php?query=&id=rs12664794) | A | G | 0.04 | ZNRD1-AS1 | intronic |
| 6 | 30043113 | 0.84 | 0.97 | [rs60477028](http://www.broadinstitute.org/mammals/haploreg/detail_v4.1.php?query=&id=rs60477028) | T | C | 0.04 | ZNRD1-AS1 | intronic |
| 6 | 30044563 | 0.84 | 0.97 | [rs6940552](http://www.broadinstitute.org/mammals/haploreg/detail_v4.1.php?query=&id=rs6940552) | G | A | 0.04 | ZNRD1-AS1 | intronic |
| 6 | 30048520 | 0.84 | 0.97 | [rs6457138](http://www.broadinstitute.org/mammals/haploreg/detail_v4.1.php?query=&id=rs6457138) | A | C | 0.04 | ZNRD1-AS1 | intronic |
| **Locus 9. Query SNP: rs1490387 and variants with r^2^ >= 0.8** | | | | | |  |  |  |  |
| 6 | 126337897 | 0.86 | 0.93 | [rs6919397](http://www.broadinstitute.org/mammals/haploreg/detail_v4.1.php?query=&id=rs6919397) | T | G | 0.45 | RP11-73O6.4 | intergenic |
| 6 | 126339209 | 0.86 | 0.93 | [rs79950493](http://www.broadinstitute.org/mammals/haploreg/detail_v4.1.php?query=&id=rs79950493) | T | TAGG | 0.45 | RP11-73O6.4 | intergenic |
| 6 | 126340008 | 0.86 | 0.93 | [rs9388486](http://www.broadinstitute.org/mammals/haploreg/detail_v4.1.php?query=&id=rs9388486) | T | C | 0.45 | RP11-73O6.4 | intergenic |
| 6 | 126340712 | 0.85 | 0.93 | [rs9375435](http://www.broadinstitute.org/mammals/haploreg/detail_v4.1.php?query=&id=rs9375435) | C | T | 0.46 | CENPW | intronic |
| 6 | 126343187 | 0.87 | 0.94 | [rs17754780](http://www.broadinstitute.org/mammals/haploreg/detail_v4.1.php?query=&id=rs17754780) | C | T | 0.45 | CENPW | intronic |
| 6 | 126345270 | 0.86 | 0.94 | [rs6907898](http://www.broadinstitute.org/mammals/haploreg/detail_v4.1.php?query=&id=rs6907898) | C | T | 0.45 | CENPW | intronic |
| 6 | 126345677 | 0.86 | 0.94 | [rs200987420](http://www.broadinstitute.org/mammals/haploreg/detail_v4.1.php?query=&id=rs200987420) | A | AAAT | 0.45 | CENPW | intronic |
| 6 | 126345678 | 0.86 | 0.94 | [rs201988238](http://www.broadinstitute.org/mammals/haploreg/detail_v4.1.php?query=&id=rs201988238) | A | AATAAC | 0.45 | CENPW | intronic |
| 6 | 126348333 | 0.86 | 0.94 | [rs9401876](http://www.broadinstitute.org/mammals/haploreg/detail_v4.1.php?query=&id=rs9401876) | C | A | 0.45 | CENPW | intronic |
| 6 | 126353208 | 0.87 | 0.94 | [rs2045258](http://www.broadinstitute.org/mammals/haploreg/detail_v4.1.php?query=&id=rs2045258) | A | G | 0.45 | RP11-73O6.4 | intergenic |
| 6 | 126357122 | 0.82 | 0.93 | [rs9388487](http://www.broadinstitute.org/mammals/haploreg/detail_v4.1.php?query=&id=rs9388487) | G | T | 0.47 | RP11-73O6.4 | intergenic |
| 6 | 126357960 | 0.87 | 0.94 | [rs9491624](http://www.broadinstitute.org/mammals/haploreg/detail_v4.1.php?query=&id=rs9491624) | A | T | 0.45 | RP11-73O6.4 | intergenic |
| 6 | 126359343 | 0.87 | 0.94 | [rs2326387](http://www.broadinstitute.org/mammals/haploreg/detail_v4.1.php?query=&id=rs2326387) | G | A | 0.45 | RP11-73O6.4 | intergenic |
| 6 | 126360922 | 0.86 | 0.94 | [rs35634111](http://www.broadinstitute.org/mammals/haploreg/detail_v4.1.php?query=&id=rs35634111) | AT | A | 0.45 | RP11-73O6.4 | intergenic |
| 6 | 126362448 | 0.82 | 0.93 | [rs9398803](http://www.broadinstitute.org/mammals/haploreg/detail_v4.1.php?query=&id=rs9398803) | A | G | 0.47 | RP11-73O6.4 | intergenic |
| 6 | 126364681 | 0.85 | 0.93 | [rs2027299](http://www.broadinstitute.org/mammals/haploreg/detail_v4.1.php?query=&id=rs2027299) | G | C | 0.45 | RP11-73O6.4 | intergenic |
| 6 | 126365367 | 0.87 | 0.94 | [rs4895807](http://www.broadinstitute.org/mammals/haploreg/detail_v4.1.php?query=&id=rs4895807) | C | G | 0.45 | RP11-73O6.4 | intergenic |
| 6 | 126377573 | 0.89 | 0.95 | [rs9388489](http://www.broadinstitute.org/mammals/haploreg/detail_v4.1.php?query=&id=rs9388489) | A | G | 0.45 | RP11-73O6.4 | intergenic |
| 6 | 126377904 | 0.89 | 0.95 | [rs67126256](http://www.broadinstitute.org/mammals/haploreg/detail_v4.1.php?query=&id=rs67126256) | TG | T | 0.45 | RP11-73O6.4 | intergenic |
| 6 | 126380821 | 0.87 | 0.94 | [rs1361262](http://www.broadinstitute.org/mammals/haploreg/detail_v4.1.php?query=&id=rs1361262) | T | C | 0.45 | RP11-73O6.4 | intergenic |
| 6 | 126382244 | 0.86 | 0.94 | [rs9398804](http://www.broadinstitute.org/mammals/haploreg/detail_v4.1.php?query=&id=rs9398804) | T | A | 0.45 | RP11-73O6.4 | intergenic |
| 6 | 126383649 | 0.85 | 0.95 | [rs9388490](http://www.broadinstitute.org/mammals/haploreg/detail_v4.1.php?query=&id=rs9388490) | C | T | 0.44 | RP11-73O6.4 | intergenic |
| 6 | 126386699 | 0.84 | 0.92 | [rs11307281](http://www.broadinstitute.org/mammals/haploreg/detail_v4.1.php?query=&id=rs11307281) | CT | C | 0.46 | RP11-73O6.4 | intergenic |
| 6 | 126391101 | 0.9 | 0.95 | [rs1578060](http://www.broadinstitute.org/mammals/haploreg/detail_v4.1.php?query=&id=rs1578060) | G | C | 0.45 | RP11-73O6.4 | intergenic |
| 6 | 126405149 | 0.9 | 0.95 | [rs9398805](http://www.broadinstitute.org/mammals/haploreg/detail_v4.1.php?query=&id=rs9398805) | C | T | 0.46 | RP11-73O6.4 | intergenic |
| 6 | 126406266 | 0.83 | 0.93 | [rs199553791](http://www.broadinstitute.org/mammals/haploreg/detail_v4.1.php?query=&id=rs199553791) | T | TA | 0.44 | RP11-73O6.4 | intergenic |
| 6 | 126406784 | 0.85 | 0.96 | [rs4897179](http://www.broadinstitute.org/mammals/haploreg/detail_v4.1.php?query=&id=rs4897179) | G | A | 0.44 | RP11-73O6.4 | intergenic |
| 6 | 126406804 | 0.88 | 0.95 | [rs4897180](http://www.broadinstitute.org/mammals/haploreg/detail_v4.1.php?query=&id=rs4897180) | A | T | 0.45 | RP11-73O6.4 | intergenic |
| 6 | 126409397 | 0.89 | 0.94 | [rs576049](http://www.broadinstitute.org/mammals/haploreg/detail_v4.1.php?query=&id=rs576049) | T | G | 0.45 | RP11-73O6.4 | intergenic |
| 6 | 126411948 | 0.8 | 0.92 | [rs59698523](http://www.broadinstitute.org/mammals/haploreg/detail_v4.1.php?query=&id=rs59698523) | T | C | 0.44 | RP11-73O6.4 | intergenic |
| 6 | 126411994 | 0.82 | 0.9 | [rs112166936](http://www.broadinstitute.org/mammals/haploreg/detail_v4.1.php?query=&id=rs112166936) | A | C | 0.45 | RP11-73O6.4 | intergenic |
| 6 | 126419677 | 0.87 | 0.93 | [rs34279746](http://www.broadinstitute.org/mammals/haploreg/detail_v4.1.php?query=&id=rs34279746) | TTA | T | 0.45 | RP11-73O6.4 | intergenic |
| 6 | 126422941 | 0.9 | 0.95 | [rs9321065](http://www.broadinstitute.org/mammals/haploreg/detail_v4.1.php?query=&id=rs9321065) | G | A | 0.45 | RP11-73O6.4 | intergenic |
| 6 | 126423847 | 0.87 | 0.95 | [rs145608233](http://www.broadinstitute.org/mammals/haploreg/detail_v4.1.php?query=&id=rs145608233) | AATT | A | 0.44 | RP11-73O6.4 | intergenic |
| 6 | 126423930 | 0.86 | 0.94 | [rs2039735](http://www.broadinstitute.org/mammals/haploreg/detail_v4.1.php?query=&id=rs2039735) | T | C | 0.45 | RP11-73O6.4 | intergenic |
| 6 | 126427347 | 0.91 | 0.96 | [rs1538172](http://www.broadinstitute.org/mammals/haploreg/detail_v4.1.php?query=&id=rs1538172) | A | G | 0.46 | RP11-73O6.4 | intergenic |
| 6 | 126430775 | 0.92 | 0.96 | [rs9401881](http://www.broadinstitute.org/mammals/haploreg/detail_v4.1.php?query=&id=rs9401881) | C | T | 0.46 | RP11-73O6.4 | intergenic |
| 6 | 126431652 | 0.92 | 0.96 | [rs4565329](http://www.broadinstitute.org/mammals/haploreg/detail_v4.1.php?query=&id=rs4565329) | C | T | 0.46 | RP11-73O6.4 | intergenic |
| 6 | 126431728 | 0.92 | 0.96 | [rs1538170](http://www.broadinstitute.org/mammals/haploreg/detail_v4.1.php?query=&id=rs1538170) | C | T | 0.46 | RP11-73O6.4 | intergenic |
| 6 | 126431738 | 0.92 | 0.96 | [rs1538171](http://www.broadinstitute.org/mammals/haploreg/detail_v4.1.php?query=&id=rs1538171) | C | G | 0.46 | RP11-73O6.4 | intergenic |
| 6 | 126432418 | 0.92 | 0.96 | [rs4897181](http://www.broadinstitute.org/mammals/haploreg/detail_v4.1.php?query=&id=rs4897181) | C | T | 0.46 | RP11-73O6.4 | intergenic |
| 6 | 126433909 | 0.92 | 0.96 | [rs9401882](http://www.broadinstitute.org/mammals/haploreg/detail_v4.1.php?query=&id=rs9401882) | A | G | 0.46 | RP11-73O6.4 | intergenic |
| 6 | 126434496 | 0.92 | 0.96 | [rs4897182](http://www.broadinstitute.org/mammals/haploreg/detail_v4.1.php?query=&id=rs4897182) | T | G | 0.46 | RP11-73O6.4 | intergenic |
| 6 | 126437394 | 0.92 | 0.96 | [rs9375439](http://www.broadinstitute.org/mammals/haploreg/detail_v4.1.php?query=&id=rs9375439) | C | G | 0.46 | RP11-73O6.4 | intergenic |
| 6 | 126437645 | 0.92 | 0.96 | [rs9398808](http://www.broadinstitute.org/mammals/haploreg/detail_v4.1.php?query=&id=rs9398808) | G | T | 0.46 | RP11-73O6.4 | intergenic |
| 6 | 126437887 | 0.92 | 0.96 | [rs9385399](http://www.broadinstitute.org/mammals/haploreg/detail_v4.1.php?query=&id=rs9385399) | G | T | 0.46 | RP11-73O6.4 | intergenic |
| 6 | 126438441 | 0.92 | 0.96 | [rs1415671](http://www.broadinstitute.org/mammals/haploreg/detail_v4.1.php?query=&id=rs1415671) | T | G | 0.46 | RP11-73O6.4 | intergenic |
| 6 | 126438494 | 0.86 | 0.96 | [rs149232167](http://www.broadinstitute.org/mammals/haploreg/detail_v4.1.php?query=&id=rs149232167) | T | TG,TT | 0.44 | RP11-73O6.4 | intergenic |
| 6 | 126439848 | 0.91 | 0.96 | [rs2184968](http://www.broadinstitute.org/mammals/haploreg/detail_v4.1.php?query=&id=rs2184968) | T | C | 0.46 | RP11-73O6.4 | intergenic |
| 6 | 126440082 | 0.92 | 0.96 | [rs2152876](http://www.broadinstitute.org/mammals/haploreg/detail_v4.1.php?query=&id=rs2152876) | G | A | 0.46 | RP11-73O6.4 | intergenic |
| 6 | 126443044 | 0.92 | 0.96 | [rs9385400](http://www.broadinstitute.org/mammals/haploreg/detail_v4.1.php?query=&id=rs9385400) | T | G | 0.46 | RP11-73O6.4 | intergenic |
| 6 | 126446365 | 0.91 | 0.96 | [rs1361107](http://www.broadinstitute.org/mammals/haploreg/detail_v4.1.php?query=&id=rs1361107) | A | C | 0.46 | RP11-73O6.4 | intergenic |
| 6 | 126446454 | 0.92 | 0.96 | [rs1361108](http://www.broadinstitute.org/mammals/haploreg/detail_v4.1.php?query=&id=rs1361108) | C | T | 0.46 | RP11-73O6.4 | intergenic |
| 6 | 126449878 | 0.92 | 0.96 | [rs1337735](http://www.broadinstitute.org/mammals/haploreg/detail_v4.1.php?query=&id=rs1337735) | T | A | 0.46 | RP11-73O6.4 | intergenic |
| 6 | 126449997 | 0.92 | 0.96 | [rs1361109](http://www.broadinstitute.org/mammals/haploreg/detail_v4.1.php?query=&id=rs1361109) | C | T | 0.46 | RP11-73O6.4 | intergenic |
| 6 | 126450541 | 0.93 | 0.96 | [rs1572569](http://www.broadinstitute.org/mammals/haploreg/detail_v4.1.php?query=&id=rs1572569) | G | A | 0.45 | RP11-73O6.4 | intergenic |
| 6 | 126452341 | 0.93 | 0.96 | [rs2184967](http://www.broadinstitute.org/mammals/haploreg/detail_v4.1.php?query=&id=rs2184967) | C | T | 0.45 | RP11-73O6.4 | intergenic |
| 6 | 126452434 | 0.93 | 0.96 | [rs4559102](http://www.broadinstitute.org/mammals/haploreg/detail_v4.1.php?query=&id=rs4559102) | G | A | 0.45 | RP11-73O6.4 | intergenic |
| 6 | 126454948 | 0.92 | 0.96 | [rs9388494](http://www.broadinstitute.org/mammals/haploreg/detail_v4.1.php?query=&id=rs9388494) | G | T | 0.46 | RP11-73O6.4 | intergenic |
| 6 | 126457454 | 0.93 | 0.96 | [rs9375441](http://www.broadinstitute.org/mammals/haploreg/detail_v4.1.php?query=&id=rs9375441) | G | A | 0.45 | RP11-73O6.4 | intergenic |
| 6 | 126460288 | 0.93 | 0.96 | [rs4895808](http://www.broadinstitute.org/mammals/haploreg/detail_v4.1.php?query=&id=rs4895808) | C | T | 0.45 | RP11-73O6.4 | intergenic |
| 6 | 126461311 | 0.92 | 0.96 | [rs9388495](http://www.broadinstitute.org/mammals/haploreg/detail_v4.1.php?query=&id=rs9388495) | G | A | 0.46 | RP11-73O6.4 | intergenic |
| 6 | 126463916 | 0.98 | 0.99 | [rs9388496](http://www.broadinstitute.org/mammals/haploreg/detail_v4.1.php?query=&id=rs9388496) | A | G | 0.45 | RP11-73O6.4 | intergenic |
| 6 | 126466961 | 0.97 | 0.99 | [rs1844594](http://www.broadinstitute.org/mammals/haploreg/detail_v4.1.php?query=&id=rs1844594) | G | A | 0.46 | RP11-73O6.4 | intergenic |
| 6 | 126467998 | 0.98 | 0.99 | [rs9398809](http://www.broadinstitute.org/mammals/haploreg/detail_v4.1.php?query=&id=rs9398809) | C | T | 0.45 | RP11-73O6.4 | intergenic |
| 6 | 126468326 | 0.98 | 0.99 | [rs9385401](http://www.broadinstitute.org/mammals/haploreg/detail_v4.1.php?query=&id=rs9385401) | C | T | 0.45 | RP11-73O6.4 | intergenic |
| 6 | 126475965 | 0.98 | 0.99 | [rs9401883](http://www.broadinstitute.org/mammals/haploreg/detail_v4.1.php?query=&id=rs9401883) | A | G | 0.45 | RP11-73O6.4 | intergenic |
| 6 | 126479998 | 0.99 | 0.99 | [rs1159619](http://www.broadinstitute.org/mammals/haploreg/detail_v4.1.php?query=&id=rs1159619) | C | A | 0.45 | RP11-73O6.4 | intergenic |
| 6 | 126481991 | 0.98 | 0.99 | [rs9375442](http://www.broadinstitute.org/mammals/haploreg/detail_v4.1.php?query=&id=rs9375442) | A | C | 0.45 | RP11-73O6.4 | intergenic |
| 6 | 126484109 | 0.99 | 0.99 | [rs2050644](http://www.broadinstitute.org/mammals/haploreg/detail_v4.1.php?query=&id=rs2050644) | A | G | 0.45 | RP11-73O6.4 | intergenic |
| 6 | 126485530 | 0.99 | 0.99 | [rs4422634](http://www.broadinstitute.org/mammals/haploreg/detail_v4.1.php?query=&id=rs4422634) | T | C | 0.45 | RP11-73O6.4 | intergenic |
| 6 | 126487491 | 0.97 | 0.99 | [rs1120786](http://www.broadinstitute.org/mammals/haploreg/detail_v4.1.php?query=&id=rs1120786) | T | G | 0.45 | RP11-73O6.4 | intergenic |
| 6 | 126488724 | 0.98 | 0.99 | [rs9372839](http://www.broadinstitute.org/mammals/haploreg/detail_v4.1.php?query=&id=rs9372839) | G | A | 0.45 | RP11-73O6.4 | intergenic |
| 6 | 126490782 | 0.98 | 0.99 | [rs2326451](http://www.broadinstitute.org/mammals/haploreg/detail_v4.1.php?query=&id=rs2326451) | A | T | 0.46 | RP11-73O6.4 | intergenic |
| 6 | 126494458 | 0.99 | 0.99 | [rs9398810](http://www.broadinstitute.org/mammals/haploreg/detail_v4.1.php?query=&id=rs9398810) | C | A | 0.45 | RP11-73O6.4 | intergenic |
| 6 | 126498553 | 0.99 | 0.99 | [rs4418209](http://www.broadinstitute.org/mammals/haploreg/detail_v4.1.php?query=&id=rs4418209) | T | G | 0.45 | RP11-73O6.4 | intergenic |
| 6 | 126501489 | 0.99 | 1 | [rs9372840](http://www.broadinstitute.org/mammals/haploreg/detail_v4.1.php?query=&id=rs9372840) | A | C | 0.45 | RP11-73O6.4 | intergenic |
| 6 | 126504691 | 1 | 1 | [rs9401885](http://www.broadinstitute.org/mammals/haploreg/detail_v4.1.php?query=&id=rs9401885) | C | G | 0.45 | RP11-73O6.4 | intergenic |
| 6 | 126504739 | 0.96 | 1 | [rs72422730](http://www.broadinstitute.org/mammals/haploreg/detail_v4.1.php?query=&id=rs72422730) | 8-mer | A | 0.44 | RP11-73O6.4 | intergenic |
| 6 | 126505673 | 0.99 | 1 | [rs2130603](http://www.broadinstitute.org/mammals/haploreg/detail_v4.1.php?query=&id=rs2130603) | A | C | 0.45 | RP11-73O6.4 | intergenic |
| 6 | 126508835 | 0.99 | 1 | [rs7738135](http://www.broadinstitute.org/mammals/haploreg/detail_v4.1.php?query=&id=rs7738135) | G | A | 0.45 | RP11-73O6.4 | intergenic |
| 6 | 126509366 | 0.99 | 1 | [rs4053271](http://www.broadinstitute.org/mammals/haploreg/detail_v4.1.php?query=&id=rs4053271) | T | C | 0.45 | RP11-73O6.4 | intergenic |
| 6 | 126512937 | 1 | 1 | [rs1490387](http://www.broadinstitute.org/mammals/haploreg/detail_v4.1.php?query=&id=rs1490387) | C | A | 0.45 | RP11-73O6.4 | intergenic |
| 6 | 126513869 | 1 | 1 | [rs9375446](http://www.broadinstitute.org/mammals/haploreg/detail_v4.1.php?query=&id=rs9375446) | G | A | 0.45 | RP11-73O6.4 | intergenic |
| 6 | 126514509 | 1 | 1 | [rs1490388](http://www.broadinstitute.org/mammals/haploreg/detail_v4.1.php?query=&id=rs1490388) | C | T | 0.45 | RP11-73O6.4 | intergenic |
| 6 | 126516517 | 1 | 1 | [rs1907067](http://www.broadinstitute.org/mammals/haploreg/detail_v4.1.php?query=&id=rs1907067) | A | C | 0.45 | RP11-73O6.4 | intergenic |
| 6 | 126517459 | 0.99 | 1 | [rs1602278](http://www.broadinstitute.org/mammals/haploreg/detail_v4.1.php?query=&id=rs1602278) | A | C,G | 0.46 | RP11-73O6.4 | intergenic |
| 6 | 126530014 | 0.85 | 0.99 | [rs1490384](http://www.broadinstitute.org/mammals/haploreg/detail_v4.1.php?query=&id=rs1490384) | C | T | 0.49 | RP11-73O6.4 | intergenic |
| **Locus 10. Query SNP: rs7022618 and variants with r^2^ >= 0.8** | | | | | |  |  |  |  |
| 9 | 114813645 | 0.89 | 0.96 | [rs10817677](http://www.broadinstitute.org/mammals/haploreg/detail_v4.1.php?query=&id=rs10817677) | C | T | 0.14 | 7.5kb 5' of TNFSF15 | intergenic |
| 9 | 114814199 | 0.89 | 0.96 | [rs7865494](http://www.broadinstitute.org/mammals/haploreg/detail_v4.1.php?query=&id=rs7865494) | C | T | 0.14 | 8.1kb 5' of TNFSF15 | intergenic |
| 9 | 114814687 | 0.85 | 0.96 | [rs7866221](http://www.broadinstitute.org/mammals/haploreg/detail_v4.1.php?query=&id=rs7866221) | G | T | 0.15 | 8.6kb 5' of TNFSF15 | intergenic |
| 9 | 114814957 | 0.85 | 0.96 | [rs7866379](http://www.broadinstitute.org/mammals/haploreg/detail_v4.1.php?query=&id=rs7866379) | C | T | 0.15 | 8.8kb 5' of TNFSF15 | intergenic |
| 9 | 114816160 | 0.88 | 0.96 | [rs10117997](http://www.broadinstitute.org/mammals/haploreg/detail_v4.1.php?query=&id=rs10117997) | A | T | 0.15 | 10kb 5' of TNFSF15 | intergenic |
| 9 | 114816811 | 0.8 | 0.96 | [rs55897861](http://www.broadinstitute.org/mammals/haploreg/detail_v4.1.php?query=&id=rs55897861) | A | 10-mer | 0.16 | 11kb 5' of TNFSF15 | intergenic |
| 9 | 114818898 | 0.87 | 0.96 | [rs12340243](http://www.broadinstitute.org/mammals/haploreg/detail_v4.1.php?query=&id=rs12340243) | T | G | 0.15 | 13kb 5' of TNFSF15 | intergenic |
| 9 | 114819380 | 0.86 | 0.96 | [rs10982415](http://www.broadinstitute.org/mammals/haploreg/detail_v4.1.php?query=&id=rs10982415) | G | A | 0.15 | 13kb 5' of TNFSF15 | intergenic |
| 9 | 114819660 | 0.87 | 0.97 | [rs2145929](http://www.broadinstitute.org/mammals/haploreg/detail_v4.1.php?query=&id=rs2145929) | T | C | 0.15 | 14kb 5' of TNFSF15 | intergenic |
| 9 | 114819807 | 0.87 | 0.97 | [rs7873916](http://www.broadinstitute.org/mammals/haploreg/detail_v4.1.php?query=&id=rs7873916) | A | G | 0.15 | 14kb 5' of TNFSF15 | intergenic |
| 9 | 114823090 | 0.81 | 0.97 | [rs7029554](http://www.broadinstitute.org/mammals/haploreg/detail_v4.1.php?query=&id=rs7029554) | A | G | 0.16 | 17kb 5' of TNFSF15 | intergenic |
| 9 | 114823185 | 0.82 | 0.97 | [rs7032906](http://www.broadinstitute.org/mammals/haploreg/detail_v4.1.php?query=&id=rs7032906) | C | T | 0.16 | 17kb 5' of TNFSF15 | intergenic |
| 9 | 114824129 | 0.82 | 0.97 | [rs1407306](http://www.broadinstitute.org/mammals/haploreg/detail_v4.1.php?query=&id=rs1407306) | G | T | 0.16 | 18kb 5' of TNFSF15 | intergenic |
| 9 | 114824742 | 0.86 | 0.97 | [rs1419134](http://www.broadinstitute.org/mammals/haploreg/detail_v4.1.php?query=&id=rs1419134) | A | G | 0.15 | 19kb 5' of TNFSF15 | intergenic |
| 9 | 114825133 | 0.88 | 0.96 | [rs12337233](http://www.broadinstitute.org/mammals/haploreg/detail_v4.1.php?query=&id=rs12337233) | C | T | 0.15 | 19kb 5' of TNFSF15 | intergenic |
| 9 | 114827294 | 0.81 | 0.96 | [rs4262377](http://www.broadinstitute.org/mammals/haploreg/detail_v4.1.php?query=&id=rs4262377) | G | T | 0.12 | 21kb 5' of TNFSF15 | intergenic |
| 9 | 114827333 | 0.8 | 0.96 | [rs10982417](http://www.broadinstitute.org/mammals/haploreg/detail_v4.1.php?query=&id=rs10982417) | C | T | 0.12 | 21kb 5' of TNFSF15 | intergenic |
| 9 | 114829725 | 0.8 | 0.96 | [rs7468800](http://www.broadinstitute.org/mammals/haploreg/detail_v4.1.php?query=&id=rs7468800) | C | A | 0.12 | 24kb 5' of TNFSF15 | intergenic |
| 9 | 114831737 | 0.82 | 0.97 | [rs7872350](http://www.broadinstitute.org/mammals/haploreg/detail_v4.1.php?query=&id=rs7872350) | C | T | 0.12 | 26kb 5' of TNFSF15 | intergenic |
| 9 | 114833900 | 0.83 | 0.98 | [rs6478111](http://www.broadinstitute.org/mammals/haploreg/detail_v4.1.php?query=&id=rs6478111) | C | T | 0.12 | 28kb 5' of TNFSF15 | intergenic |
| 9 | 114838803 | 0.81 | 0.95 | [rs10982420](http://www.broadinstitute.org/mammals/haploreg/detail_v4.1.php?query=&id=rs10982420) | A | T | 0.13 | 33kb 5' of TNFSF15 | intergenic |
| 9 | 114840016 | 0.8 | 0.94 | [rs56235203](http://www.broadinstitute.org/mammals/haploreg/detail_v4.1.php?query=&id=rs56235203) | A | G | 0.13 | 34kb 5' of TNFSF15 | intergenic |
| 9 | 114840879 | 0.81 | 0.95 | [rs10982421](http://www.broadinstitute.org/mammals/haploreg/detail_v4.1.php?query=&id=rs10982421) | C | A | 0.13 | 35kb 5' of TNFSF15 | intergenic |
| 9 | 114841503 | 0.81 | 0.95 | [rs10982422](http://www.broadinstitute.org/mammals/haploreg/detail_v4.1.php?query=&id=rs10982422) | T | C | 0.13 | 35kb 5' of TNFSF15 | intergenic |
| 9 | 114841966 | 0.8 | 0.95 | [rs1075074](http://www.broadinstitute.org/mammals/haploreg/detail_v4.1.php?query=&id=rs1075074) | A | G | 0.13 | 36kb 5' of TNFSF15 | intergenic |
| 9 | 114842234 | 0.8 | 0.95 | [rs11560576](http://www.broadinstitute.org/mammals/haploreg/detail_v4.1.php?query=&id=rs11560576) | A | T | 0.13 | 36kb 5' of TNFSF15 | intergenic |
| 9 | 114842790 | 0.8 | 0.94 | [rs11554257](http://www.broadinstitute.org/mammals/haploreg/detail_v4.1.php?query=&id=rs11554257) | T | C | 0.13 | 37kb 5' of TNFSF15 | intergenic |
| 9 | 114842969 | 0.81 | 0.95 | [rs2418318](http://www.broadinstitute.org/mammals/haploreg/detail_v4.1.php?query=&id=rs2418318) | T | C | 0.13 | 37kb 5' of TNFSF15 | intergenic |
| 9 | 114843582 | 0.81 | 0.95 | [rs10982423](http://www.broadinstitute.org/mammals/haploreg/detail_v4.1.php?query=&id=rs10982423) | A | C | 0.13 | 37kb 5' of TNFSF15 | intergenic |
| 9 | 114843864 | 0.82 | 0.96 | [rs10982424](http://www.broadinstitute.org/mammals/haploreg/detail_v4.1.php?query=&id=rs10982424) | G | A | 0.13 | 38kb 5' of TNFSF15 | intergenic |
| 9 | 114843925 | 0.82 | 0.96 | [rs10982425](http://www.broadinstitute.org/mammals/haploreg/detail_v4.1.php?query=&id=rs10982425) | C | T | 0.13 | 38kb 5' of TNFSF15 | intergenic |
| 9 | 114845338 | 0.82 | 0.97 | [rs12237931](http://www.broadinstitute.org/mammals/haploreg/detail_v4.1.php?query=&id=rs12237931) | G | A | 0.12 | 39kb 5' of TNFSF15 | intergenic |
| 9 | 114845728 | 0.97 | 1 | [rs1885385](http://www.broadinstitute.org/mammals/haploreg/detail_v4.1.php?query=&id=rs1885385) | T | A | 0.14 | 40kb 5' of TNFSF15 | intergenic |
| 9 | 114847443 | 0.82 | 0.97 | [rs10491581](http://www.broadinstitute.org/mammals/haploreg/detail_v4.1.php?query=&id=rs10491581) | T | A | 0.12 | 41kb 5' of TNFSF15 | intergenic |
| 9 | 114847639 | 0.81 | 0.96 | [rs16931895](http://www.broadinstitute.org/mammals/haploreg/detail_v4.1.php?query=&id=rs16931895) | A | C | 0.12 | 42kb 5' of TNFSF15 | intergenic |
| 9 | 114849633 | 0.83 | 0.99 | [rs2093403](http://www.broadinstitute.org/mammals/haploreg/detail_v4.1.php?query=&id=rs2093403) | T | C | 0.12 | 44kb 5' of TNFSF15 | intergenic |
| 9 | 114850752 | 0.98 | 1 | [rs10114224](http://www.broadinstitute.org/mammals/haploreg/detail_v4.1.php?query=&id=rs10114224) | T | C | 0.14 | 43kb 3' of TNFSF8 | intergenic |
| 9 | 114851416 | 0.98 | 1 | [rs10982427](http://www.broadinstitute.org/mammals/haploreg/detail_v4.1.php?query=&id=rs10982427) | T | C | 0.14 | 42kb 3' of TNFSF8 | intergenic |
| 9 | 114852782 | 0.99 | 1 | [rs4348576](http://www.broadinstitute.org/mammals/haploreg/detail_v4.1.php?query=&id=rs4348576) | A | G | 0.14 | 41kb 3' of TNFSF8 | intergenic |
| 9 | 114854276 | 1 | 1 | [rs7022618](http://www.broadinstitute.org/mammals/haploreg/detail_v4.1.php?query=&id=rs7022618) | T | C | 0.14 | 39kb 3' of TNFSF8 | intergenic |
| 9 | 114855220 | 0.99 | 1 | [rs10115186](http://www.broadinstitute.org/mammals/haploreg/detail_v4.1.php?query=&id=rs10115186) | G | C | 0.14 | 39kb 3' of TNFSF8 | intergenic |
| 9 | 114855286 | 0.83 | 0.99 | [rs10982431](http://www.broadinstitute.org/mammals/haploreg/detail_v4.1.php?query=&id=rs10982431) | G | A | 0.12 | 38kb 3' of TNFSF8 | intergenic |
| 9 | 114855993 | 0.83 | 0.99 | [rs79021241](http://www.broadinstitute.org/mammals/haploreg/detail_v4.1.php?query=&id=rs79021241) | C | T | 0.12 | 38kb 3' of TNFSF8 | intergenic |
| 9 | 114856377 | 0.99 | 1 | [rs10119266](http://www.broadinstitute.org/mammals/haploreg/detail_v4.1.php?query=&id=rs10119266) | T | A | 0.14 | 37kb 3' of TNFSF8 | intergenic |
| 9 | 114856390 | 0.99 | 1 | [rs10122171](http://www.broadinstitute.org/mammals/haploreg/detail_v4.1.php?query=&id=rs10122171) | A | T | 0.14 | 37kb 3' of TNFSF8 | intergenic |
| 9 | 114856435 | 0.99 | 1 | [rs10116032](http://www.broadinstitute.org/mammals/haploreg/detail_v4.1.php?query=&id=rs10116032) | C | T | 0.14 | 37kb 3' of TNFSF8 | intergenic |
| 9 | 114856907 | 0.98 | 1 | [rs16931913](http://www.broadinstitute.org/mammals/haploreg/detail_v4.1.php?query=&id=rs16931913) | C | A | 0.14 | 37kb 3' of TNFSF8 | intergenic |
| 9 | 114857329 | 0.99 | 1 | [rs7043587](http://www.broadinstitute.org/mammals/haploreg/detail_v4.1.php?query=&id=rs7043587) | T | C | 0.14 | 36kb 3' of TNFSF8 | intergenic |
| 9 | 114857408 | 0.98 | 1 | [rs7027268](http://www.broadinstitute.org/mammals/haploreg/detail_v4.1.php?query=&id=rs7027268) | A | G | 0.14 | 36kb 3' of TNFSF8 | intergenic |
| 9 | 114857479 | 0.99 | 1 | [rs2418319](http://www.broadinstitute.org/mammals/haploreg/detail_v4.1.php?query=&id=rs2418319) | T | C | 0.14 | 36kb 3' of TNFSF8 | intergenic |
| 9 | 114857644 | 0.99 | 1 | [rs2418320](http://www.broadinstitute.org/mammals/haploreg/detail_v4.1.php?query=&id=rs2418320) | T | C | 0.14 | 36kb 3' of TNFSF8 | intergenic |
| 9 | 114857767 | 0.83 | 0.99 | [rs2418321](http://www.broadinstitute.org/mammals/haploreg/detail_v4.1.php?query=&id=rs2418321) | C | T | 0.12 | 36kb 3' of TNFSF8 | intergenic |
| 9 | 114858124 | 0.82 | 0.97 | [rs10982433](http://www.broadinstitute.org/mammals/haploreg/detail_v4.1.php?query=&id=rs10982433) | T | C | 0.12 | 36kb 3' of TNFSF8 | intergenic |
| 9 | 114858435 | 0.82 | 0.97 | [rs2145931](http://www.broadinstitute.org/mammals/haploreg/detail_v4.1.php?query=&id=rs2145931) | T | C | 0.12 | 35kb 3' of TNFSF8 | intergenic |
| 9 | 114858683 | 0.83 | 0.99 | [rs149084342](http://www.broadinstitute.org/mammals/haploreg/detail_v4.1.php?query=&id=rs149084342) | TC | T | 0.12 | 35kb 3' of TNFSF8 | intergenic |
| 9 | 114859473 | 1 | 1 | [rs10982434](http://www.broadinstitute.org/mammals/haploreg/detail_v4.1.php?query=&id=rs10982434) | C | T | 0.14 | 34kb 3' of TNFSF8 | intergenic |
| 9 | 114862450 | 0.83 | 0.99 | [rs10982436](http://www.broadinstitute.org/mammals/haploreg/detail_v4.1.php?query=&id=rs10982436) | G | C | 0.12 | 31kb 3' of TNFSF8 | intergenic |
| 9 | 114863588 | 0.99 | 1 | [rs7866387](http://www.broadinstitute.org/mammals/haploreg/detail_v4.1.php?query=&id=rs7866387) | T | C | 0.14 | 30kb 3' of TNFSF8 | intergenic |
| 9 | 114863619 | 0.83 | 0.99 | [rs10982438](http://www.broadinstitute.org/mammals/haploreg/detail_v4.1.php?query=&id=rs10982438) | G | A | 0.12 | 30kb 3' of TNFSF8 | intergenic |
| 9 | 114864530 | 0.98 | 1 | [rs7027093](http://www.broadinstitute.org/mammals/haploreg/detail_v4.1.php?query=&id=rs7027093) | A | G | 0.14 | 29kb 3' of TNFSF8 | intergenic |
| 9 | 114864911 | 0.85 | 0.99 | [rs202132262](http://www.broadinstitute.org/mammals/haploreg/detail_v4.1.php?query=&id=rs202132262) | CA | C | 0.12 | 29kb 3' of TNFSF8 | intergenic |
| 9 | 114864914 | 0.97 | 0.99 | [rs56136096](http://www.broadinstitute.org/mammals/haploreg/detail_v4.1.php?query=&id=rs56136096) | T | A | 0.14 | 29kb 3' of TNFSF8 | intergenic |
| **Locus 11. Query SNP: rs72758135 and variants with r^2^ >= 0.8** | | | | | |  |  |  |  |
| 9 | 120862871 | 1 | 1 | [rs72758135](http://www.broadinstitute.org/mammals/haploreg/detail_v4.1.php?query=&id=rs72758135) | T | C | 0.13 | PHF19 | intronic |
| 9 | 120864033 | 0.87 | 1 | [rs10985064](http://www.broadinstitute.org/mammals/haploreg/detail_v4.1.php?query=&id=rs10985064) | C | A | 0.12 | PHF19 | intronic |
| 9 | 120864255 | 0.86 | 0.99 | [rs12377227](http://www.broadinstitute.org/mammals/haploreg/detail_v4.1.php?query=&id=rs12377227) | A | G | 0.12 | PHF19 | intronic |
|  |  | 0.87 | 1 | [rs148772466](http://www.broadinstitute.org/mammals/haploreg/detail_v4.1.php?query=&id=rs148772466) | 8-mer | T | 0.12 | PHF19 | intronic |
| 9 | 120864792 | 0.87 | 1 | [rs72758138](http://www.broadinstitute.org/mammals/haploreg/detail_v4.1.php?query=&id=rs72758138) | G | C | 0.12 | PHF19 | intronic |
| 9 | 120865282 | 0.85 | 0.97 | [rs62581711](http://www.broadinstitute.org/mammals/haploreg/detail_v4.1.php?query=&id=rs62581711) | G | A | 0.12 | PHF19 | intronic |
| 9 | 120866549 | 0.87 | 1 | [rs10985066](http://www.broadinstitute.org/mammals/haploreg/detail_v4.1.php?query=&id=rs10985066) | G | C | 0.12 | PHF19 | intronic |
| 9 | 120867372 | 0.87 | 1 | [rs10985067](http://www.broadinstitute.org/mammals/haploreg/detail_v4.1.php?query=&id=rs10985067) | G | A | 0.12 | PHF19 | intronic |
| 9 | 120867446 | 0.87 | 1 | [rs10985068](http://www.broadinstitute.org/mammals/haploreg/detail_v4.1.php?query=&id=rs10985068) | G | C | 0.12 | PHF19 | intronic |
| **Locus 12. Query SNP: rs1360119 and variants with r^2^ >= 0.8** | | | | | |  |  |  |  |
| 10 | 30401778 | 0.82 | 1 | [rs75201195](http://www.broadinstitute.org/mammals/haploreg/detail_v4.1.php?query=&id=rs75201195) | G | T | 0.02 | 27kb 5' of MTPAP | intergenic |
| 10 | 30415753 | 0.88 | 1 | [rs116688297](http://www.broadinstitute.org/mammals/haploreg/detail_v4.1.php?query=&id=rs116688297) | A | G | 0.02 | 18kb 5' of MAP3K8 | intergenic |
| 10 | 30421514 | 0.82 | 0.94 | [rs139462166](http://www.broadinstitute.org/mammals/haploreg/detail_v4.1.php?query=&id=rs139462166) | G | T | 0.02 | 12kb 5' of MAP3K8 | intergenic |
| 10 | 30422726 | 0.88 | 1 | [rs143851974](http://www.broadinstitute.org/mammals/haploreg/detail_v4.1.php?query=&id=rs143851974) | G | C | 0.02 | 11kb 5' of MAP3K8 | intergenic |
| 10 | 30428353 | 0.82 | 0.94 | [rs114738416](http://www.broadinstitute.org/mammals/haploreg/detail_v4.1.php?query=&id=rs114738416) | G | A | 0.02 | 5.6kb 5' of MAP3K8 | intergenic |
| 10 | 30434097 | 1 | 1 | [rs8176952](http://www.broadinstitute.org/mammals/haploreg/detail_v4.1.php?query=&id=rs8176952) | G | A | 0.02 | MAP3K8 | 5'-UTR |
| 10 | 30434777 | 1 | 1 | [rs1360119](http://www.broadinstitute.org/mammals/haploreg/detail_v4.1.php?query=&id=rs1360119) | C | A | 0.02 | MAP3K8 | intronic |
| 10 | 30435543 | 0.88 | 1 | [rs76069125](http://www.broadinstitute.org/mammals/haploreg/detail_v4.1.php?query=&id=rs76069125) | T | C | 0.02 | MAP3K8 | intronic |
| 10 | 30436848 | 0.88 | 1 | [rs79392497](http://www.broadinstitute.org/mammals/haploreg/detail_v4.1.php?query=&id=rs79392497) | C | T | 0.02 | MAP3K8 | intronic |
| 10 | 30440775 | 0.88 | 1 | [rs141857700](http://www.broadinstitute.org/mammals/haploreg/detail_v4.1.php?query=&id=rs141857700) | G | A | 0.02 | MAP3K8 | intronic |
| **Locus 13. Query SNP: rs7092540 and variants with r^2^ >= 0.8** | | | | | |  |  |  |  |
| 10 | 53092145 | 0.95 | 1 | [rs951289](http://www.broadinstitute.org/mammals/haploreg/detail_v4.1.php?query=&id=rs951289) | C | T | 0.03 | 116kb 5' of RP11-319F12.2 | intergenic |
| 10 | 53092368 | 1 | 1 | [rs951287](http://www.broadinstitute.org/mammals/haploreg/detail_v4.1.php?query=&id=rs951287) | C | T | 0.03 | 117kb 5' of RP11-319F12.2 | intergenic |
| 10 | 53092470 | 0.9 | 1 | [rs951286](http://www.broadinstitute.org/mammals/haploreg/detail_v4.1.php?query=&id=rs951286) | C | T | 0.02 | 117kb 5' of RP11-319F12.2 | intergenic |
| 10 | 53093470 | 0.95 | 1 | [rs73350293](http://www.broadinstitute.org/mammals/haploreg/detail_v4.1.php?query=&id=rs73350293) | T | A | 0.03 | 118kb 5' of RP11-319F12.2 | intergenic |
| 10 | 53094196 | 1 | 1 | [rs7092540](http://www.broadinstitute.org/mammals/haploreg/detail_v4.1.php?query=&id=rs7092540) | G | A | 0.03 | 118kb 5' of RP11-319F12.2 | intergenic |
| 10 | 53095751 | 0.9 | 1 | [rs117932090](http://www.broadinstitute.org/mammals/haploreg/detail_v4.1.php?query=&id=rs117932090) | G | A | 0.02 | 120kb 5' of RP11-319F12.2 | intergenic |
| 10 | 53095970 | 0.9 | 1 | [rs77554008](http://www.broadinstitute.org/mammals/haploreg/detail_v4.1.php?query=&id=rs77554008) | T | C | 0.02 | 120kb 5' of RP11-319F12.2 | intergenic |
| 10 | 53096806 | 0.9 | 1 | [rs143715967](http://www.broadinstitute.org/mammals/haploreg/detail_v4.1.php?query=&id=rs143715967) | 13-mer | T | 0.02 | 121kb 5' of RP11-319F12.2 | intergenic |
| 10 | 53097291 | 0.9 | 1 | [rs117797312](http://www.broadinstitute.org/mammals/haploreg/detail_v4.1.php?query=&id=rs117797312) | C | T | 0.02 | 121kb 5' of RP11-319F12.2 | intergenic |
| 10 | 53097965 | 0.9 | 1 | [rs116906593](http://www.broadinstitute.org/mammals/haploreg/detail_v4.1.php?query=&id=rs116906593) | C | T | 0.02 | 122kb 5' of RP11-319F12.2 | intergenic |
| 10 | 53098299 | 0.9 | 1 | [rs77174046](http://www.broadinstitute.org/mammals/haploreg/detail_v4.1.php?query=&id=rs77174046) | A | G | 0.02 | 122kb 5' of RP11-319F12.2 | intergenic |
| 10 | 53098634 | 0.9 | 1 | [rs76456878](http://www.broadinstitute.org/mammals/haploreg/detail_v4.1.php?query=&id=rs76456878) | C | T | 0.02 | 123kb 5' of RP11-319F12.2 | intergenic |
| 10 | 53098965 | 0.9 | 1 | [rs76515300](http://www.broadinstitute.org/mammals/haploreg/detail_v4.1.php?query=&id=rs76515300) | T | C | 0.02 | 123kb 5' of RP11-319F12.2 | intergenic |
| 10 | 53099452 | 0.9 | 1 | [rs76657066](http://www.broadinstitute.org/mammals/haploreg/detail_v4.1.php?query=&id=rs76657066) | G | A | 0.02 | 124kb 5' of RP11-319F12.2 | intergenic |
| 10 | 53100607 | 0.9 | 1 | [rs146597526](http://www.broadinstitute.org/mammals/haploreg/detail_v4.1.php?query=&id=rs146597526) | G | A | 0.02 | 125kb 5' of RP11-319F12.2 | intergenic |
| 10 | 53102644 | 0.9 | 1 | [rs77627116](http://www.broadinstitute.org/mammals/haploreg/detail_v4.1.php?query=&id=rs77627116) | G | A | 0.02 | 127kb 5' of RP11-319F12.2 | intergenic |
| 10 | 53105027 | 0.9 | 1 | [rs117659648](http://www.broadinstitute.org/mammals/haploreg/detail_v4.1.php?query=&id=rs117659648) | C | T | 0.02 | 129kb 5' of RP11-319F12.2 | intergenic |
| 10 | 53105878 | 0.9 | 1 | [rs78263838](http://www.broadinstitute.org/mammals/haploreg/detail_v4.1.php?query=&id=rs78263838) | T | C | 0.02 | 130kb 5' of RP11-319F12.2 | intergenic |
| **Locus 14. Query SNP: rs59665078 and variants with r^2^ >= 0.8** | | | | | |  |  |  |  |
| 12 | 128790563 | 1 | 1 | [rs59665078](http://www.broadinstitute.org/mammals/haploreg/detail_v4.1.php?query=&id=rs59665078) | T | C | 0.07 | 2.6kb 3' of SLC15A4 | intergenic |
| **Locus 15. Query SNP: rs7149309 and variants with r^2^ >= 0.8** | | | | | |  |  |  |  |
| 14 | 94467053 | 1 | 1 | [rs7149309](http://www.broadinstitute.org/mammals/haploreg/detail_v4.1.php?query=&id=rs7149309) | C | T | 0.01 | SERPINA9 | intronic |
| **Locus 16. Query SNP: rs1802141 and variants with r^2^ >= 0.8** | | | | | |  |  |  |  |
| 16 | 28956102 | 0.82 | 1 | [rs73533880](http://www.broadinstitute.org/mammals/haploreg/detail_v4.1.php?query=&id=rs73533880) | C | T | 0.01 | NFATC2IP | intronic |
| 16 | 28956367 | 0.82 | 1 | [rs8047616](http://www.broadinstitute.org/mammals/haploreg/detail_v4.1.php?query=&id=rs8047616) | C | T | 0.01 | NFATC2IP | intronic |
| 16 | 28957978 | 0.82 | 1 | [rs111925530](http://www.broadinstitute.org/mammals/haploreg/detail_v4.1.php?query=&id=rs111925530) | C | T | 0.01 | RP11-264B17.2 | intronic |
| 16 | 28958196 | 0.82 | 1 | [rs60603404](http://www.broadinstitute.org/mammals/haploreg/detail_v4.1.php?query=&id=rs60603404) | T | A | 0.01 | RP11-264B17.2 | intronic |
| 16 | 28961777 | 0.82 | 1 | [rs113677814](http://www.broadinstitute.org/mammals/haploreg/detail_v4.1.php?query=&id=rs113677814) | A | G | 0.01 | RP11-264B17.2 | intronic |
| 16 | 28963337 | 0.82 | 1 | [rs8044999](http://www.broadinstitute.org/mammals/haploreg/detail_v4.1.php?query=&id=rs8044999) | A | G | 0.01 | RP11-264B17.2 | intronic |
| 16 | 28970906 | 0.82 | 1 | [rs8060015](http://www.broadinstitute.org/mammals/haploreg/detail_v4.1.php?query=&id=rs8060015) | A | G | 0.01 | 3.3kb 5' of SPNS1 | intergenic |
| 16 | 28971984 | 0.82 | 1 | [rs148131850](http://www.broadinstitute.org/mammals/haploreg/detail_v4.1.php?query=&id=rs148131850) | CT | C | 0.01 | 2.2kb 5' of SPNS1 | intergenic |
| 16 | 28973283 | 0.82 | 1 | [rs11863981](http://www.broadinstitute.org/mammals/haploreg/detail_v4.1.php?query=&id=rs11863981) | G | C | 0.01 | 937bp 5' of SPNS1 | intergenic |
| 16 | 28974410 | 0.82 | 1 | [rs58714362](http://www.broadinstitute.org/mammals/haploreg/detail_v4.1.php?query=&id=rs58714362) | T | A | 0.01 | SPNS1 | intergenic |
| 16 | 28975676 | 0.82 | 1 | [rs7184156](http://www.broadinstitute.org/mammals/haploreg/detail_v4.1.php?query=&id=rs7184156) | G | A | 0.01 | SPNS1 | intronic |
| 16 | 28976332 | 0.82 | 1 | [rs7189583](http://www.broadinstitute.org/mammals/haploreg/detail_v4.1.php?query=&id=rs7189583) | A | C | 0.01 | SPNS1 | intronic |
| 16 | 28977491 | 0.82 | 1 | [rs8044724](http://www.broadinstitute.org/mammals/haploreg/detail_v4.1.php?query=&id=rs8044724) | G | A | 0.01 | SPNS1 | intronic |
| 16 | 28979639 | 0.82 | 1 | [rs9925291](http://www.broadinstitute.org/mammals/haploreg/detail_v4.1.php?query=&id=rs9925291) | C | T | 0.01 | SPNS1 | intronic |
| 16 | 28982048 | 0.82 | 1 | [rs61747536](http://www.broadinstitute.org/mammals/haploreg/detail_v4.1.php?query=&id=rs61747536) | C | T | 0.01 | **SPNS1** | **synonymous** |
| 16 | 28983736 | 0.82 | 1 | [rs11859822](http://www.broadinstitute.org/mammals/haploreg/detail_v4.1.php?query=&id=rs11859822) | T | C | 0.01 | SPNS1 | intronic |
| 16 | 28985949 | 0.82 | 1 | [rs41280846](http://www.broadinstitute.org/mammals/haploreg/detail_v4.1.php?query=&id=rs41280846) | T | C | 0.01 | RP11-264B17.3 | intronic |
| 16 | 28987287 | 0.82 | 1 | [rs73535859](http://www.broadinstitute.org/mammals/haploreg/detail_v4.1.php?query=&id=rs73535859) | C | T | 0.01 | RP11-264B17.3 | intronic |
| 16 | 28987628 | 0.82 | 1 | [rs7202093](http://www.broadinstitute.org/mammals/haploreg/detail_v4.1.php?query=&id=rs7202093) | C | T | 0.01 | RP11-264B17.3 | intronic |
| 16 | 28990688 | 1 | 1 | [rs1802141](http://www.broadinstitute.org/mammals/haploreg/detail_v4.1.php?query=&id=rs1802141) | A | G | 0.01 | RP11-264B17.5 | 3'-UTR |
| 16 | 28991788 | 1 | 1 | [rs139964413](http://www.broadinstitute.org/mammals/haploreg/detail_v4.1.php?query=&id=rs139964413) | AC | A | 0.01 | 1kb 3' of LAT | intergenic |
| 16 | 28993042 | 1 | 1 | [rs112600506](http://www.broadinstitute.org/mammals/haploreg/detail_v4.1.php?query=&id=rs112600506) | A | G | 0.01 | 2.3kb 3' of LAT | intergenic |
| 16 | 28993307 | 1 | 1 | [rs111708809](http://www.broadinstitute.org/mammals/haploreg/detail_v4.1.php?query=&id=rs111708809) | A | G | 0.01 | 2.5kb 3' of LAT | intergenic |
| 16 | 28994574 | 1 | 1 | [rs7193414](http://www.broadinstitute.org/mammals/haploreg/detail_v4.1.php?query=&id=rs7193414) | T | C | 0.01 | 3.8kb 3' of LAT | intergenic |
| **Locus 17. Query SNP: rs3848405 and variants with r^2^ >= 0.8** | | | | | |  |  |  |  |
| 17 | 79119130 | 1 | 1 | [rs3848405](http://www.broadinstitute.org/mammals/haploreg/detail_v4.1.php?query=&id=rs3848405) | T | C | 0.01 | RBFOX3 | intronic |
| 17 | 79119823 | 1 | 1 | [rs139172998](http://www.broadinstitute.org/mammals/haploreg/detail_v4.1.php?query=&id=rs139172998) | C | T | 0.01 | RBFOX3 | intronic |
| 17 | 79124876 | 0.86 | 1 | [rs73400195](http://www.broadinstitute.org/mammals/haploreg/detail_v4.1.php?query=&id=rs73400195) | C | T | 0.01 | RBFOX3 | intronic |
| **Locus 18. Query SNP: rs769450 and variants with r^2^ >= 0.8** | | | | | |  |  |  |  |
| 19 | 44895560 | 0.95 | 0.98 | [rs2238681](http://www.broadinstitute.org/mammals/haploreg/detail_v4.1.php?query=&id=rs2238681) | C | T | 0.42 | TOMM40 | intronic |
| 19 | 44898409 | 0.95 | 0.98 | [rs8106922](http://www.broadinstitute.org/mammals/haploreg/detail_v4.1.php?query=&id=rs8106922) | A | G | 0.42 | TOMM40 | Intronic |
| 19 | 44899220 | 0.96 | 0.98 | [rs34878901](http://www.broadinstitute.org/mammals/haploreg/detail_v4.1.php?query=&id=rs34878901) | C | T | 0.42 | TOMM40 | Intronic |
| 19 | 44900155 | 0.85 | 0.99 | [rs1160985](http://www.broadinstitute.org/mammals/haploreg/detail_v4.1.php?query=&id=rs1160985) | C | T | 0.46 | TOMM40 | Intronic |
| 19 | 44900601 | 0.85 | 0.99 | [rs760136](http://www.broadinstitute.org/mammals/haploreg/detail_v4.1.php?query=&id=rs760136) | A | G | 0.46 | TOMM40 | Intronic |
| 19 | 44901174 | 0.81 | 0.97 | [rs741780](http://www.broadinstitute.org/mammals/haploreg/detail_v4.1.php?query=&id=rs741780) | T | C | 0.45 | TOMM40 | Intronic |
| 19 | 44901715 | 0.84 | 0.98 | [rs1038025](http://www.broadinstitute.org/mammals/haploreg/detail_v4.1.php?query=&id=rs1038025) | T | C | 0.46 | TOMM40 | Intronic |
| 19 | 44901805 | 0.85 | 0.99 | [rs1038026](http://www.broadinstitute.org/mammals/haploreg/detail_v4.1.php?query=&id=rs1038026) | A | G | 0.46 | TOMM40 | Intronic |
| 19 | 44902264 | 0.95 | 0.98 | [rs1305062](http://www.broadinstitute.org/mammals/haploreg/detail_v4.1.php?query=&id=rs1305062) | G | C | 0.42 | TOMM40 | Intronic |
| 19 | 44904531 | 0.85 | 0.99 | [rs7259620](http://www.broadinstitute.org/mammals/haploreg/detail_v4.1.php?query=&id=rs7259620) | G | A | 0.46 | 852bp 3' of TOMM40 | Intergenic |
| 19 | 44907187 | 1 | 1 | [rs769450](http://www.broadinstitute.org/mammals/haploreg/detail_v4.1.php?query=&id=rs769450) | G | A | 0.42 | APOE | Intronic |

Abbreviations: LD, linkage disequilibrium; Eur, European
