# Supplementary material for: An integrative genomics approach identifies novel pathways that influence candidaemia susceptibility
Source: PLoS One. 2017 Jul 20;12(7):e0180824. doi: 10.1371/journal.pone.0180824 (PMC5519064; doi:10.1371/journal.pone.0180824)
Supplement: S6 Table — Bolded genes show a log2 fold change > 1.5. (DOCX) [file pone.0180824.s010.docx]

Table S6. Differential expression of genes that are located within a 500 kilobase (kb) window around the candidaemia-associated SNPs upon *Candida* stimulation at 4 hours. Bolded genes show a log2 fold change > 1.5.

| Candidaemia SNP | Gene | P adjusted | Log2FoldChange |
| --- | --- | --- | --- |
| rs769450 | BCL3 | 4.44E-36 | 1.11 |
| rs72758135 | **TRAF1** | 6.74E-58 | 1.73 |
|  | STOM | 9.70E-10 | 1.25 |
| rs7149309 | **IFI27** | 1.04E-09 | 2.37 |
|  | SERPINA1 | 2.80E-25 | 1.12 |
| rs7022618 | **TNFSF15** | 7.72E-72 | 3.89 |
|  | TNFSF8 | 9.95E-21 | 1.01 |
|  | **TNC** | 5.04E-08 | 2.28 |
| rs6748999 | PROC | 8.03E-03 | -1.22 |
| rs3848405 | **C1QTNF1** | 4.20E-09 | 2.59 |
|  | LGALS3BP | 1.44E-19 | 1.49 |
| rs3766122 | BLZF1 | 5.71E-17 | 1.33 |
| rs296537 | **LAD1** | 2.02E-11 | 2.32 |
|  | IGFN1 | 1.01E-06 | 1.19 |
|  | **TNNT2** | 2.51E-04 | 1.65 |
| rs1802141 | **IL27** | 1.11E-11 | 2.21 |
| rs1360119 | **MAP3K8** | 1.67E-23 | 1.68 |
| rs12491812 | **CISH** | 7.92E-14 | 1.62 |
