# Supplementary material for: An integrative genomics approach identifies novel pathways that influence candidaemia susceptibility
Source: PLoS One. 2017 Jul 20;12(7):e0180824. doi: 10.1371/journal.pone.0180824 (PMC5519064; doi:10.1371/journal.pone.0180824)
Supplement: S7 Table — Bolded genes show a log2 fold change > 1.5. (DOCX) [file pone.0180824.s011.docx]

Table S7. Differential expression of genes that are located within a 500 kilobase (kb) window around the candidaemia-associated SNPs upon *Candida* stimulation at 24 hours. Bolded genes show a log2 fold change > 1.5.

| Candidaemia SNP | Gene | P adjusted | Log2FoldChange |
| --- | --- | --- | --- |
| rs6699706 | AKR7A3 | 1.33E-02 | -1.36 |
| rs3766122 | ATP1B1 | 1.50E-06 | 1.42 |
| rs296537 | **IGFN1** | 1.75E-20 | 5.14 |
|  | LAD1 | 6.45E-15 | 1.19 |
| rs6748999 | PROC | 1.16E-05 | -1.76 |
|  | MYO7B | 9.41E-05 | 1.28 |
| rs12491812 | **SEMA3F** | 1.49E-07 | 2.70 |
|  | **CISH** | 2.51E-26 | 2.45 |
|  | CACNA2D2 | 4.58E-16 | -1.14 |
|  | RASSF1 | 6.67E-33 | -1.09 |
| rs11760176 | **UBD** | 5.31E-03 | 2.18 |
| rs7022618 | **TNFSF15** | 9.00E-34 | 3.34 |
| rs72758135 | **STOM** | 1.25E-29 | 1.55 |
| rs1360119 | **ZNF438** | 3.90E-40 | 1.91 |
|  | MAP3K8 | 4.14E-45 | 1.39 |
| rs59665078 | GLT1D1 | 7.24E-13 | 1.44 |
| rs7149309 | **IFI27** | 1.31E-08 | 2.94 |
|  | **SERPINA1** | 4.87E-18 | 2.59 |
|  | **SERPINA6** | 3.30E-02 | 1.72 |
| rs1802141 | **IL27** | 3.54E-10 | 3.60 |
|  | **NUPR1** | 3.05E-03 | 2.19 |
| rs3848405 | **C1QTNF1** | 1.07E-35 | 6.37 |
|  | LGALS3BP | 1.05E-06 | 1.50 |
|  | RBFOX3 | 4.78E-03 | -1.41 |
| rs769450 | **PVRL2** | 3.06E-17 | 2.51 |
|  | BCL3 | 3.32E-18 | 1.42 |
|  | APOE | 1.15E-02 | -1.27 |
|  | RELB | 9.48E-20 | 1.20 |
