# Supplementary material for: An integrative genomics approach identifies novel pathways that influence candidaemia susceptibility
Source: PLoS One. 2017 Jul 20;12(7):e0180824. doi: 10.1371/journal.pone.0180824 (PMC5519064; doi:10.1371/journal.pone.0180824)
Supplement: S8 Table — (DOCX) [file pone.0180824.s012.docx]

Table S8. All thirty-one prioritized susceptibility genes for candidaemia showed a strong enrichment for complement and coagulation pathways along with cytokine- and immune-related pathways based on KEGG and Reactome sources.

| p-value | q-value | Pathway | Source | Members/input/overlap |
| --- | --- | --- | --- | --- |
| 0.0000001 | 0.0000064 | Complement and coagulation cascades - Homo sapiens (human) | KEGG | SERPINA1; PROC; F5; C5; MBL2 |
| 0.0001401 | 0.0030831 | Staphylococcus aureus infection - Homo sapiens (human) | KEGG | SELP; C5; MBL2 |
| 0.0002155 | 0.0031604 | Hemostasis | Reactome | SELL; SERPINA1; F5; PROC; LAT; SELP |
| 0.0004579 | 0.0039998 | Platelet degranulation | Reactome | SERPINA1; SELP; F5 |
| 0.0005448 | 0.0039998 | Response to elevated platelet cytosolic Ca2+ | Reactome | SERPINA1; SELP; F5 |
| 0.0007567 | 0.0039998 | Common Pathway of Fibrin Clot Formation | Reactome | PROC; F5 |
| 0.0007760 | 0.0039998 | Platelet activation, signaling and aggregation | Reactome | LAT; SERPINA1; SELP; F5 |
| 0.0007938 | 0.0039998 | Immune System | Reactome | SELL; MAP3K8; TNFSF15; CISH; LAT; MBL2; C5; CD19 |
| 0.0008181 | 0.0039998 | Cell surface interactions at the vascular wall | Reactome | SELL; PROC; SELP |
| 0.0023813 | 0.0104777 | Formation of Fibrin Clot (Clotting Cascade) | Reactome | PROC; F5 |
| 0.0047155 | 0.0188622 | Cytokine Signaling in Immune system | Reactome | LAT; MAP3K8; CISH; TNFSF15 |
| 0.0085296 | 0.0312754 | Innate Immune System | Reactome | LAT; MAP3K8; CD19; C5; MBL2 |
