# Supplementary material for: An integrative genomics approach identifies novel pathways that influence candidaemia susceptibility
Source: PLoS One. 2017 Jul 20;12(7):e0180824. doi: 10.1371/journal.pone.0180824 (PMC5519064; doi:10.1371/journal.pone.0180824)
Supplement: S9 Table — Cytokine levels were log transformed and statistical significance was tested with Kruskal Wallis test. (DOCX) [file pone.0180824.s013.docx]

Table S9. Five additional candidaemia SNPs showed a moderate association with circulating cytokine levels as measured in serum from candidaemia patients. Cytokine levels were log transformed and statistical significance was tested with Kruskal Wallis test.

| SNP | P value (IFNγ) | P value (IL6) | P value (IL8) |
| --- | --- | --- | --- |
| rs296537 | 0.21 | 0.06 | 0.04 |
| rs7092540 | 0.01 | 0.20 | 0.24 |
| rs7149309 | 0.01 | 0.21 | 0.06 |
| rs1802141 | 0.00 | 0.07 | 0.04 |
| rs3848405 | 0.13 | 0.03 | 0.04 |
